# Isocyanides as Acceptor Groups in MHAT Reactions with Unactivated Alkenes

**DOI:** 10.1021/acs.orglett.3c02358

**Published:** 2023-08-30

**Authors:** Jordi Puig, Josep Bonjoch, Ben Bradshaw

**Affiliations:** Laboratori de Química Orgànica, Facultat de Farmàcia, Universitat de Barcelona, Av. Joan XXIII 27-31, 08028 Barcelona, Spain

## Abstract

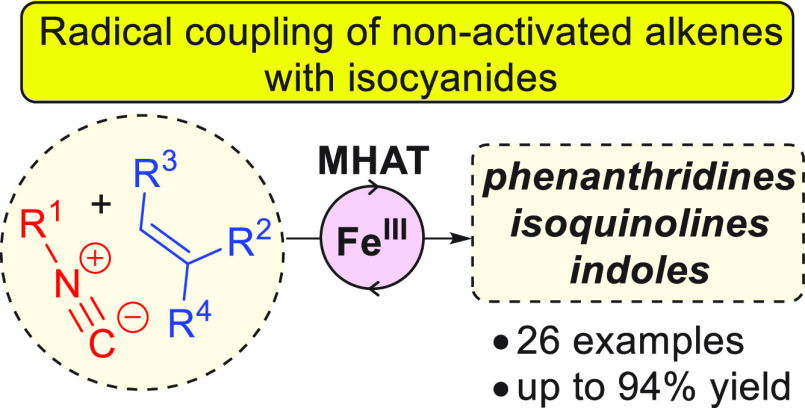

The use of isocyanides as acceptor groups in metal–hydride hydrogen atom transfer (MHAT) coupling reactions with nonactivated alkenes to form heterocycles is described. Monosubstituted alkenes couple and cyclize directly, whereas more substituted alkenes proceed via a two-step, one-pot procedure involving MHAT reductive cyclization followed by a MHAT Minisci coupling upon the addition of acid. To highlight the utility of the methodology, a diverse variety of substituted heterocycles such as phenanthridines, indoles, and isoquinolines were prepared.

The metal hydride hydrogen atom transfer (MHAT) reaction of alkenes^1^ is a potent strategy to develop new C–C bond-forming reactions via radical pathways.^2^ Key advantages of using alkenes as proradicals include their general ubiquity as chemical feedstocks, their stability in synthetic sequences, and their specific reaction profiles. Additionally, the novel disconnection possibilities arising from the use of alkenes as radical precursors has led to MHAT reactions making significant inroads into the field of total synthesis,^3^ a trend likely to increase as more acceptor groups become available in this burgeoning research area. Acceptor groups currently used in MHAT C–C coupling reactions include electron-deficient alkenes,^4^ sulfonylhydrazones derived from formaldehyde,^5^ nitriles,^6^ pyridine salts,^7^ imines,^8^*N*-sulfinylimines,^9^ acylsilanes,^10^ alkynyl bromides,^11^ β-nitroalkenes,^12^ and difluoroalkenes.^13^ Within our own research group, we have developed novel MHAT reactions employing ketones,^14^ aldehydes,^15^ Cbz hydrazones,^16^ and tosyl hydrazones^17^ as viable acceptor groups (Figure 1).

**Figure 1:**
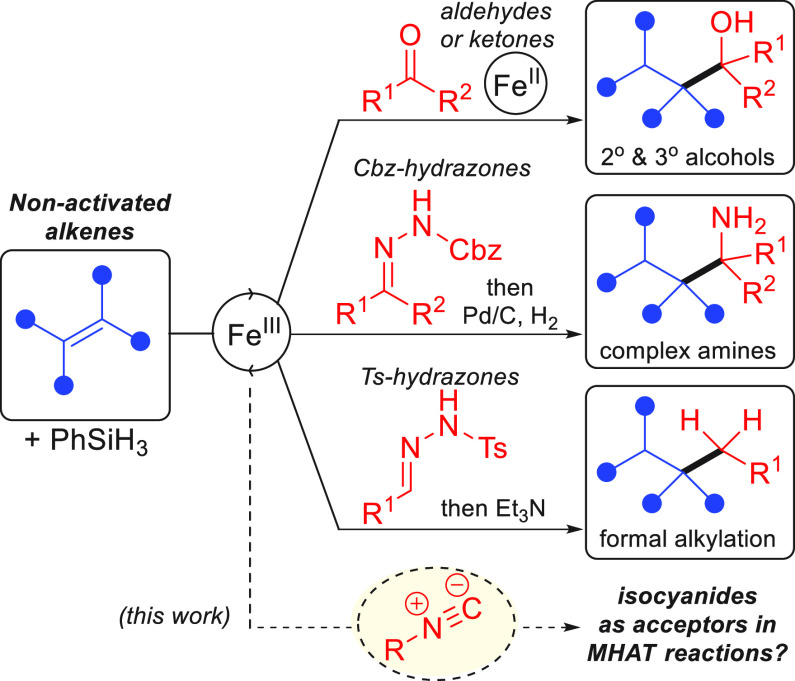
Acceptor groups used in MHAT reactions developed by our research group.

Looking to expand the pool of available acceptor groups, we focused on isocyanides, which have been widely exploited in radical reactions,^18^ especially for the synthesis of various nitrogen-containing compounds,^19^ but have yet to be studied in the context of MHAT alkene coupling reactions. The results presented herein demonstrate that isocyanides can now be added to the growing list of acceptors in MHAT C–C coupling reactions.

At the outset of this work, we envisaged that the key challenge would be to achieve a chemoselective reaction of the metal hydride species with the donor alkene as opposed to the isocyanide group.^20^ Indeed, our initial concerns that a competitive direct reductive cyclization of the isocyanide group would outcompete the reductive coupling of the alkene proved justified. When the isocyanides **1**–**5** prepared for this study were treated under MHAT conditions *without the presence of any alkene*, the corresponding heterocycles were formed in high yield (Scheme 1). Treatment of isocyanide **1** in the presence of *tert*-butyl hydroperoxide (TBHP) as an oxidant resulted in the formation of phenanthridine **6** in a 74% yield. Under identical conditions, the isocyanide precursor **2** readily gave isoquinoline **7** in an excellent 86% yield. Next, we examined several indole precursors. Substrates **3** and **4**, bearing electron-poor alkenes, gave good yields of indoles **8** and **9**, respectively, while the electron-neutral alkene **5** afforded a complex mixture of products that was difficult to identify. Notably, for the synthesis of indoles, using a mixture of THF and MeOH as the solvent in combination with heating gave better results. Furthermore, no oxidant was required, as the Fe^III^ species can be regenerated by the reduction of the formed α-radical in an analogous manner to Baran’s MHAT coupling reaction of electron-deficient alkenes.^4c^

**Scheme 1:**
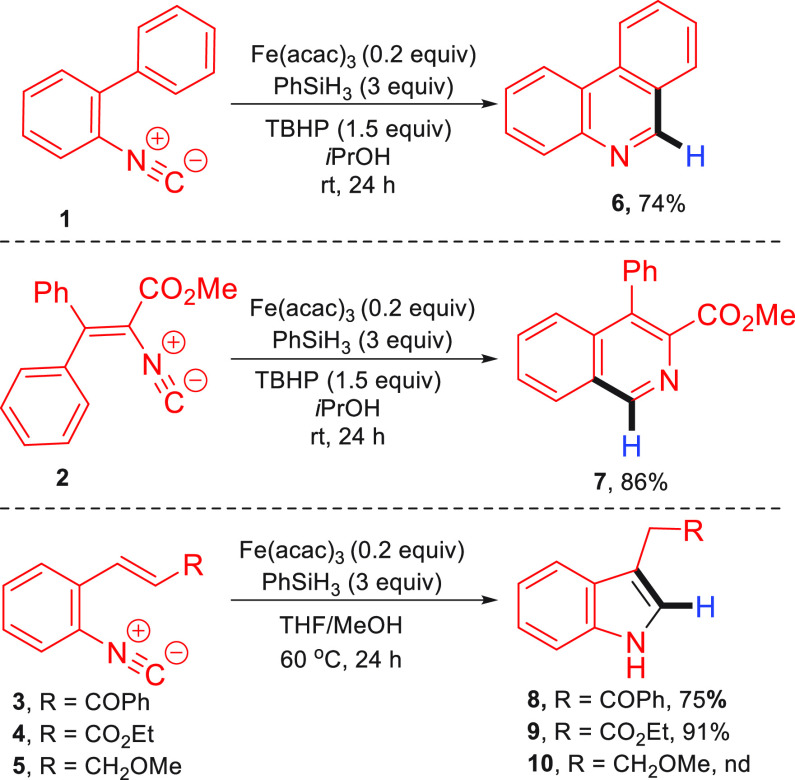
Synthesis of Core Heterocycles via MHAT

We then turned to the MHAT coupling reaction of alkenes, beginning by studying the addition of but-3-en-1-ol as the donor alkene. The initial reaction with isocyanide **1** gave only trace amounts of the desired compound **6a**, the predominant species formed being the competing reductive cyclization side product **6**. However, after extensive optimization (see the Supporting Information for full details), we were able to obtain the coupled product in a good yield of 75% (Table 1, entry 1).

**Table 1:**
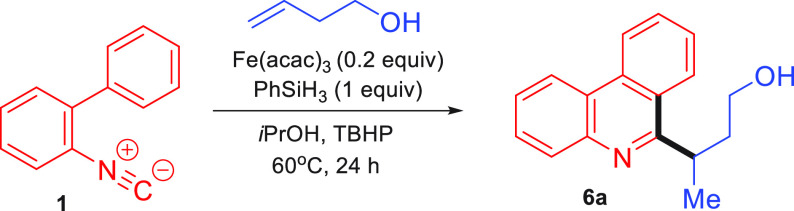
Optimization of the Reaction Conditions for the MHAT Coupling to Form Phenanthridines

entry	deviation from optimum conditions	yield
1	no variation^a^	75%
2	without heating	63%
3	3 equiv of oxidant	17%
4	THF/MeOH instead of *i*PrOH	23%
5	0.2 M *i*PrOH instead of 0.4 M^b^,^c^	30%
6	4 h instead of 24 h^c^	32%
7	2.5 equiv of PhSiH_3_^b^,^c^	30%
8	2 equiv of alkene	40%^d^
9	2 equiv of isocyanide	40%^d^
10	Fe(dibm)_3_ instead of Fe(acac)_3_^c^	40%

a Reaction conditions are as follows: isocyanide/alkene (1:1), *i*PrOH (0.4 M), and TBHP (70% in H_2_O) (1.5 equiv).

b 0.4 equiv of Fe(acac)_3_ was used.

c The reaction was performed at room temperature.

d Yield calculated with respect to the limiting reagent.

As can be observed, temperature (entry 2) made little difference to the reaction. In contrast, the quantity of oxidant (entry 3), choice of the reaction solvent (entry 4), concentration (entry 5), reaction time (entry 6), and amount of PhSiH_3_ (entry 7) proved essential for good reactivity. Somewhat surprisingly, neither increasing the quantity of alkene (entry 8) nor adding more isocyanide (entry 9) improved the reaction yield. Finally, using other forms of iron bearing a larger ligand was detrimental to the reaction’s outcome (entry 10).

With the optimized conditions in hand, the reaction scopes of the isocyanide and alkene were investigated (Schemes 2 and 3). We began by modifying the 2-isocyanobiphenyl component, where we observed that adding substituents on the aromatic rings gave phenanthridines **6b**–**6e** in yields similar to **6a**. Next, modification of the alkene component was investigated by varying the functional group and chain length of the alkenes to give compounds **6f**–**6i** in yields similar to those of the previous examples. However, we found that more substituted alkenes were less effective donors, resulting in low yields of the corresponding coupled products **6j**–**6l**. As the donor radical’s stability and steric hindrance increases, the reductive cyclization reaction is more likely to outcompete the desired alkene coupling. To overcome this setback, we proposed combining the MHAT reductive cyclization reaction outlined in Scheme 1 with a MHAT-mediated Minisci reaction.^21^

**Scheme 2:**
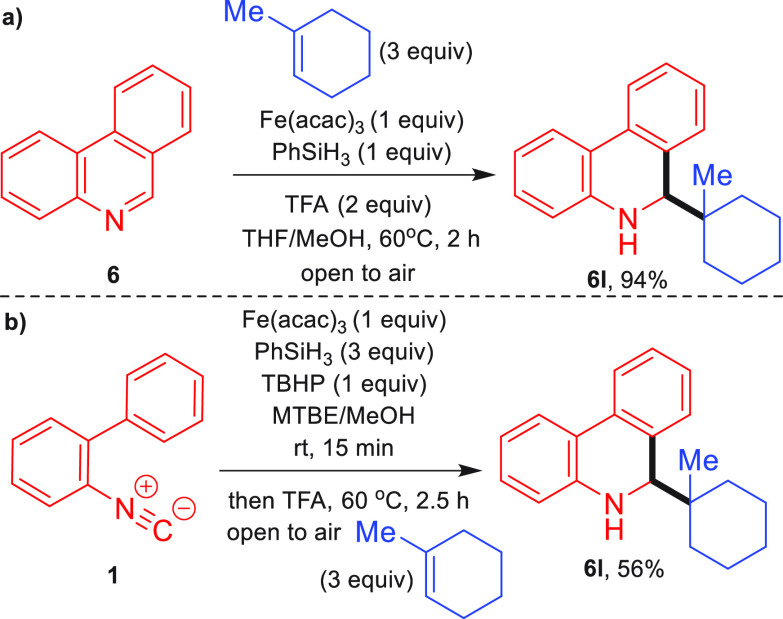
(a) MHAT Minisci Coupling Reaction and (b) Combined MHAT Reductive Cyclization and MHAT Coupling Reaction

**Scheme 3:**
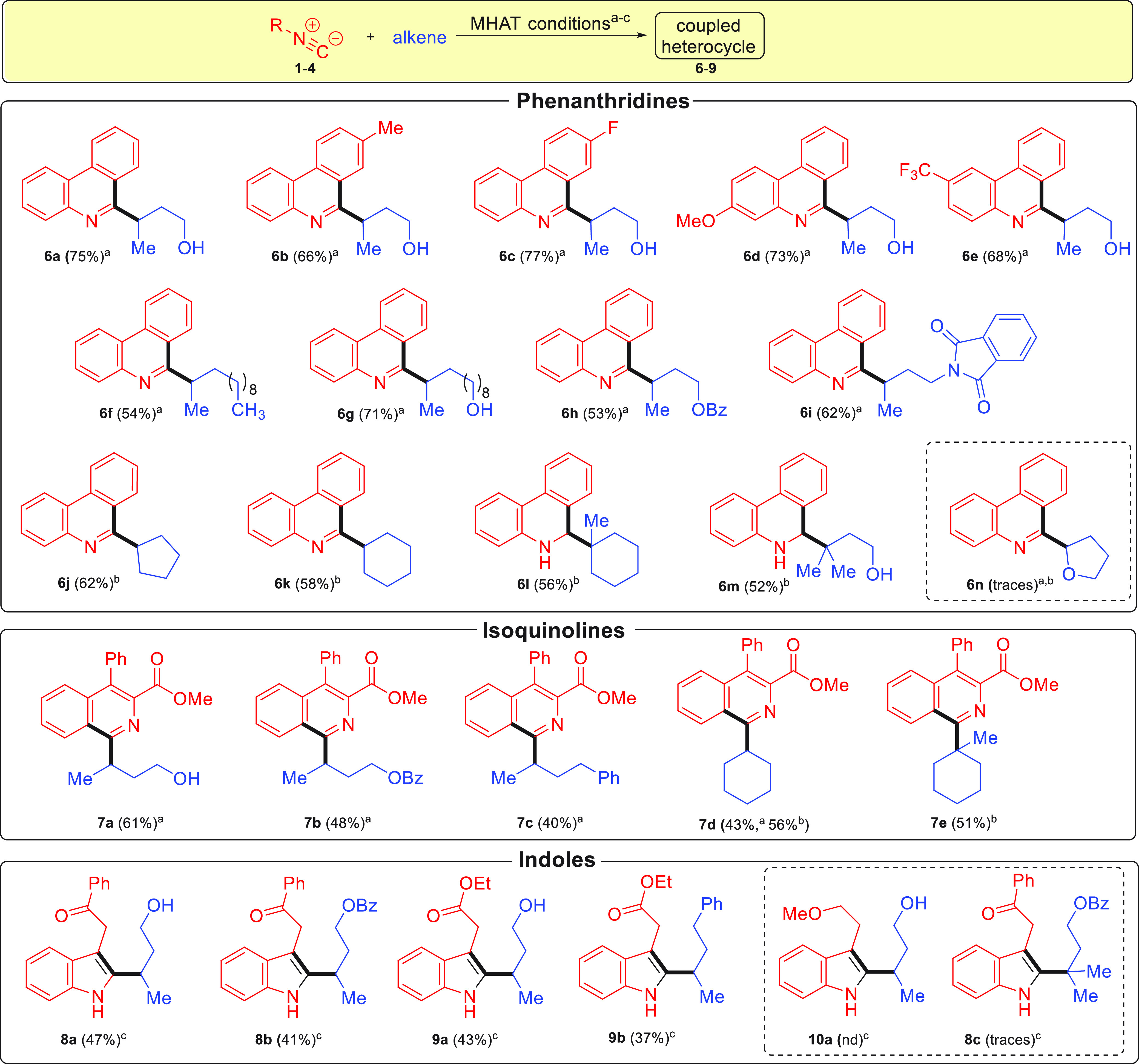
Scope of the MHAT Coupling-Cyclization Reaction Reaction conditions are as follows: isocyanide/alkene (1:1), Fe(acac)_3_ (0.2 equiv), PhSiH_3_ (1 equiv), *i*PrOH [0.4 M], and TBHP (1.5 equiv) at 60 °C for 24 h. Reaction conditions are as follows: Fe(acac)_3_ (1 equiv), PhSiH_3_ (3 equiv), TBHP (1 equiv), and MTBE/MeOH (0.2 M) at rt for 15 min, then TFA [2 equiv) and alkene (3 equiv) at 60 °C for 2.5 h open to air. Reaction conditions are as follows: isocyanide/alkene (1:1), Fe(acac)_3_ (0.2 equiv), PhSiH_3_ (1 equiv), and *i*PrOH (0.04 M] at rt for 24 h.

After considerable optimization (Supporting Information), it was found that phenanthridine **6** could be coupled with 1-methyl-1-cyclohexene to give **6l** in the presence of TFA in an excellent 94% yield. (Scheme 2a). Interestingly, the product obtained was the reduced compound, and no reoxidation of the heterocyclic ring was observed. We were then able to develop a one-pot synthesis, starting with the reductive cyclization of isocyanide **1** to **6** (determined by TLC), followed by the addition of TFA and alkene to the reaction mixture to effect the Minisci coupling reaction, which gave **6l** in a 56% yield for the overall process (Scheme 2b).

With this modified protocol now in hand, the disubstituted alkenes cyclopentane and cyclohexene could be readily coupled to give phenanthridines **6j** and **6k**. Notably, the principal products in both cases were the oxidized heterocycles.^22^ In contrast, trisubstituted alkenes **6l** and **6m** were obtained exclusively in their reduced form. Unfortunately, attempts to couple 2,3-dihydrofuran to evaluate the introduction of heteroatoms into the ring-coupled products gave only traces of the corresponding coupled product **6n**. We then investigated the use of isocyanide **2** to form coupled isoquinolines, showing that representative monosubstituted (**7a**–**7c**), disubstituted (**7d**), and trisubstituted (**7e**) alkenes could all be used as donor groups. In contrast to the phenanthridine series, only fully oxidized heterocycles were observed in all cases. The coupling of indole precursors **3**–**5** proved significantly more challenging, as these isocyanides were more susceptible to the competing reduction than the other candidate substrates. Eventually, after extensive screening of conditions, good to moderate yields of coupled products **8a**, **8b**, **9a**, and **9b** were obtained. Coupling of isocyanide **5** without an electron-withdrawing group to obtain indole **10a** yielded only a complex product mixture. Finally, the use of trisubstituted alkenes was unsuccessful, giving only traces of **8c** under direct coupling conditions. In this case, the modified one-pot reaction Minisci reaction conditions could not be employed due to the electron-rich nature of indoles.

The proposed mechanism for the reaction is outlined in Scheme 4a. Formation of the iron hydride species and addition to the alkene generate a carbon-centered radical **A** that upon addition to the isocyanide furnished the corresponding imidoyl radical **B**. A subsequent 6-*endo*-trig cyclization generates a cyclohexadienyl radical **C**, which is deprotonated by a hydroxyl anion (formed by the reaction of TBHP with Fe^II^) to give the radical anion **D**, which reduces *t*BuOOH by SET to provide the phenanthridine **6a**.^23^ Alternatively, it is possible that **C** undergoes one-electron oxidation via an Fe^III^ species or TBHP, resulting in rearomatization. Finally, oxidation of the Fe^II^ species by TBHP completes the catalytic cycle. If HAT from the iron hydride species to **1** occurs instead (i.e., in the absence of an alkene), then the noncoupled product **6** will be formed through a sequence analogous to that previously outlined. Addition of TFA at this point activates the heterocycle to give **E**, allowing it to couple via a Minisci reaction with more impeded alkenes to give **F** (Scheme 4b).

**Scheme 4:**
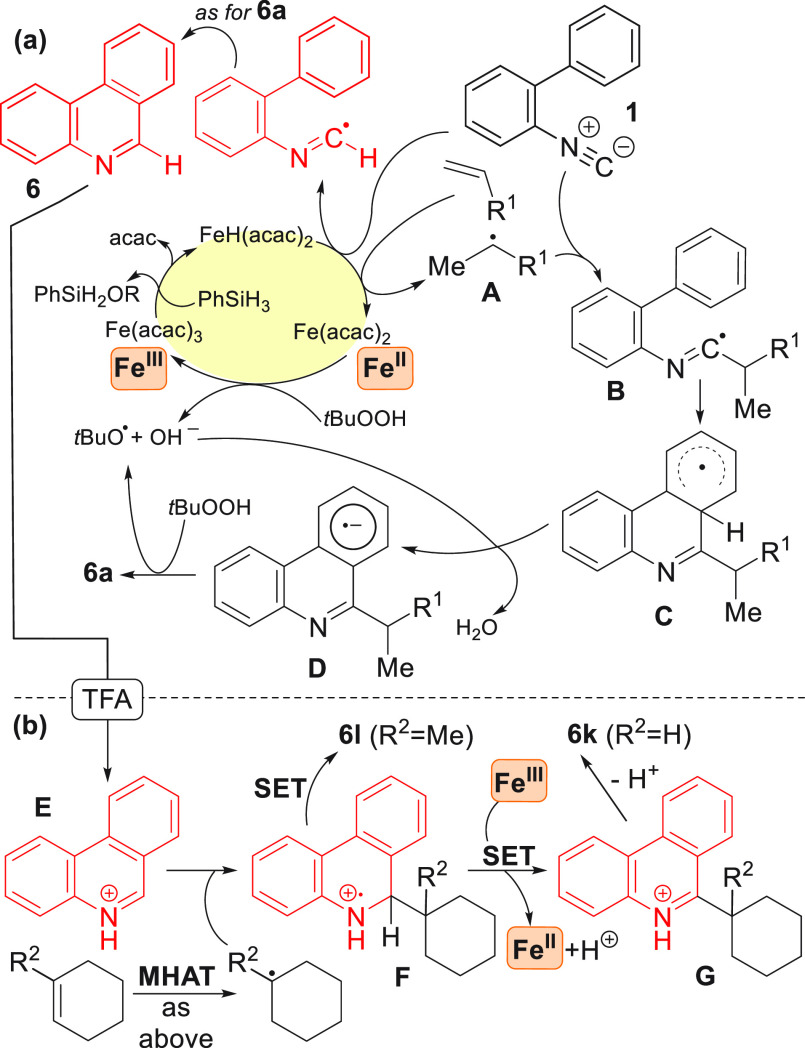
Proposed Mechanism for the MHAT Couplings with Isocyanides (a) Direct coupling of the alkene or MHAT reductive cyclization in the absence of alkene. (b) Switching to MHAT-Minisci coupling mode via the addition of TFA and the alkene.

A SET process from the Fe^III^ species results in reoxidation of the heterocyclic ring of **F** to **G** to give the coupled product **6k** (R^2^ = H) upon workup. In the case of trisubstituted alkenes (R^2^ = Me), the additional steric impediment inhibits this process, and instead the SET process occurs directly to the nitrogen to give the reduced heterocycle **6l**. It should be noted that without the addition of TFA the Minisci reaction does not take place (Supporting Information table S4, entry 37), ruling out the possibility that the heterocycle and not the isocyanide is the coupling partner, for example, under the optimum conditions of Table 1.

In summary, we have demonstrated for the first time that isocyanides can be successfully used in MHAT couplings with unactivated alkenes, allowing the synthesis of phenanthridine, isoquinoline, and indole ring systems. By combining MHAT with Minisci conditions, different mechanistic cycles can be simultaneously exploited to generate one-pot reactions. This approach opens the way for the development of other types of novel combinations in MHAT and tandem reactions to generate considerable molecular complexity in a single operation. Work in this direction is now in progress.

## Data Availability

The data underlying this study are available in the published article and its Supporting Information.

## Supporting Information Available

Experimental procedures, characterization data, and NMR spectra (PDF)
FAIR data, including the primary NMR FID files, for compounds **1–9b** (ZIP)
