# Supplementary material for: Isocyanides as Acceptor Groups in MHAT Reactions with Unactivated Alkenes
Source: Org Lett. 2023 Aug 30;25(35):6539–43. doi: 10.1021/acs.orglett.3c02358 (PMC10496133; doi:10.1021/acs.orglett.3c02358)
Supplement: Supplementary file 1 — ol3c02358_si_001.pdf [file ol3c02358_si_001.pdf]

Supporting information for:

Isocyanides as acceptor groups in MHAT reactions with unactivated alkenes

Jordi Puig, Josep Bonjoch,*
and Ben Bradshaw*

Laboratori de Química Orgànica, Facultat de Farmàcia, IBUB,
Universitat de Barcelona, Av. Joan XXIII 27-31, 08028-Barcelona, Spain
*Emails: josep.bonjoch@ub.edu, benbradshaw@ub.edu

Contents

General Information SI-2

Experimental procedures SI-3

Preparation of starting materials SI-3

Screening table of conditions for reductive couplings SI-8

Screening table of conditions for alkene MHAT couplings SI-9

Synthesis of core heterocycles via MHAT SI-12

Synthesis of coupled products via MHAT SI-13

NMR Assignment Tables SI-26

Copies of ^{1}H, ^{13}C and ^{19}F NMR spectra SI-28

References SI-65

General Information

All reactions were carried out under an Argon atmosphere with dry, freshly distilled solvents under anhydrous conditions. Analytical thin-layer chromatography was performed on SiO_2 (Merck silica gel 60 F_{254}), and the spots were located with 1% aqueous $KMnO_4$ or 2% ethanolic anisaldehyde. Chromatography refers to flash chromatography and was carried out on SiO_2 (SDS silica gel 60 ACC, 35-75 µm, 230-240 mesh ASTM) or aluminum oxide (neutral) pH 6.5-7.5 (63-200 µm). Drying of organic extracts during workup of reactions was performed over anhydrous Na_2SO_4. Evaporation of solvent was accomplished with a rotatory evaporator. NMR spectra were recorded in $CDCl_3$ except were stated otherwise and the chemical shifts of ^1H and ^{13}C NMR spectra are reported in ppm downfield (δ) from Me_4Si or $CDCl_3$. All NMR data assignments are supported by gCOSY and gHSQC experiments. High resolution mass spectra (HMRS) were performed using an electrospray (ESI) ionization source and a TOF analyzer (Agilent Technologies).

Experimental procedures
Preparation of starting materials

Biphenylamines **SI-1b**, **SI-1c**, **SI-1d,** and **SI-1e** were prepared according to the reported procedure.[1] O-Alkenylarylamines **SI-4**,[2] **SI-6**,[3] and **SI-8**[4] were prepared according to the reported procedures. Alkenes are commercially available, except for but-3-en-1-yl benzoate[5] and 2-(but-3-en-1-yl)isoindoline-1,3-dione.[6]

2-Isocyano-1,1'-biphenyl (1a)

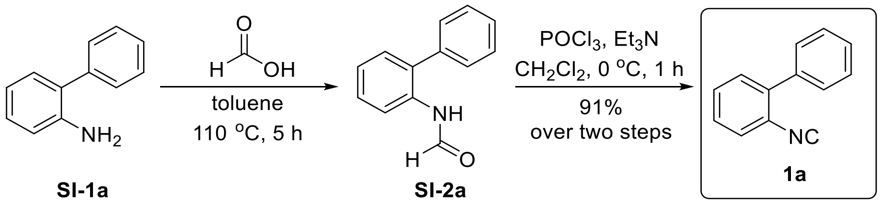

General procedure for the preparation of isocyanides: A solution of **SI-1a** (3 g, 17.7 mmol) and formic acid (98%, 3.1 mL, 79.8 mmol) in toluene (1 M, 18 mL) was refluxed using a Dean-Stark apparatus for 5 h. The reaction was quenched with a saturated aq. Na_2CO_3 solution (10 mL) and extracted with CH_2Cl_2 (3 × 10 mL). The combined organic extracts were washed with brine, dried, and concentrated. The formamide **SI-2a** was shown to be an unstable compound, so the two steps were conducted consecutively. Using material from a separate experiment a small sample was purified by chromatography (hexane → hexane/EtOAc 50:50) to give a mixture of rotamers; ^{1}H NMR (400 MHz, $CDCl_3$) δ 8.46–8.43 (m, 0.5H), 8.27 (dd, *J* = 8.2 Hz, 1H), 8.12–8.10 (m, 0.5H), 7.53 (br s, 1H), 7.41 (t, *J* = 7.2 Hz, 2H), 7.36–7.27 (m, 4H), 7.24–7.19 (m, 1H), 7.15 (t, *J* = 7.2 Hz, 1H). To a solution of the crude formamide **SI-2a** (3.5 g, 17.7 mmol) and Et_3N (20 mL, 0.14 mol) in anhydrous CH_2Cl_2 (36 mL) at 0 °C, was added $POCl_3$ (3.3 mL, 35.5 mmol) dropwise and the mixture was stirred for 1 h at 0 °C. The reaction was quenched by slowly adding a saturated aq. Na_2CO_3 solution (10 mL) and the mixture was allowed to stir for a further 1 h. The mixture was extracted with CH_2Cl_2 (3 × 15 mL), and combined organic extracts were washed with water and brine, dried and concentrated. Purification by chromatography (hexane → hexane/EtOAc 95:5) gave **1a** (2.90 g, 91% over two steps) as a green oil; ^{1}H NMR (400 MHz, $CDCl_3$) δ 7.53–7.43 (m, 8H, Ph), 7.40–7.36 (m, 1H, Ph); ^{13}C NMR (101 MHz, $CDCl_3$) δ 166.8 (NC), 138.9 (C_{ipso}), 137.1 (C_{ipso}), 130.7 (Ph), 129.6 (Ph), 129.1 (Ph), 128.7 (Ph), 128.5 (Ph), 128.2 (Ph), 127.9 (Ph). Spectral data were identical to those previously reported.[7]

2-isocyano-4'-methyl-1,1'-biphenyl (1b)

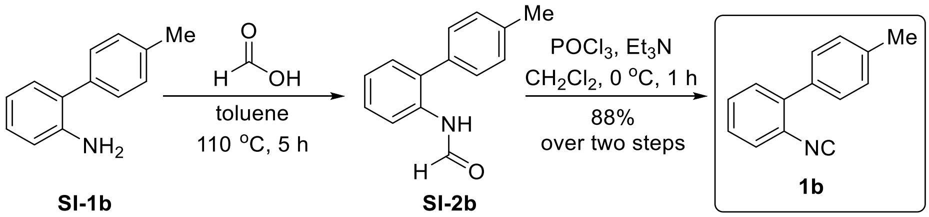

According to the general procedure for the preparation of isocyanides **SI-1b** (500 mg, 2.73 mmol) and formic acid (98%, 0.46 mL, 12.3 mmol) in toluene (1 M, 3 mL) gave

formamide **SI-2b** as a white solid; ^{1}H NMR (400 MHz, $CDCl_3$) δ 8.69 (d, *J* = 11.2 Hz, 0.5 H), 8.38 (d, *J* = 6.8 Hz, 0.5 H), 8.29 (d, *J* = 2 Hz, 0.5 H), 7.39–7.14 (m, 8H), 2.49 (s, 1.5 H), 2.41 (s, 1.5 H). The crude formamide **SI-2b** (576 mg, 2.73 mmol), Et_3N (3 mL, 21.8 mmol), anhydrous THF (5.5 mL) and $POCl_3$ (0.51 mL, 5.46 mmol) gave after purification by chromatography (hexane → hexane/EtOAc 97.5:2.5) **1b** (465 mg, 88% over two steps) as a green oil; ^{1}H NMR (400 MHz, $CDCl_3$) δ 7.50–7.44 (m, 5H, Ph), 7.38–7.32 (m, 3H, Ph), 2.46 (s, 3H, Me); ^{13}C NMR (101 MHz, $CDCl_3$) δ 166.5 (NC), 138.8 (C_{ipso}), 138.2 (C_{ipso}), 134.1 (C_{ipso}), 130.5 (Ph), 129.5 (Ph), 129.3 (Ph), 128.8 (Ph), 127.9 (Ph), 127.8 (Ph), 21.2 (Me). Spectral data were identical to those previously reported.[1]

4'-fluoro-2-isocyano-1,1'-biphenyl (1c)

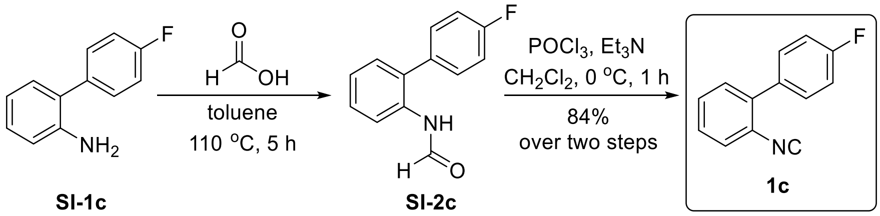

According to the general procedure for the preparation of isocyanides **SI-1c** (800 mg, 4.27 mmol) and formic acid (98%, 0.72 mL, 19.2 mmol) in toluene (1 M, 4.30 mL) gave formamide **SI-2c** as a brownish solid; ^{1}H NMR (400 MHz, $CDCl_3$) δ 8.67 (d, *J* = 11.2 Hz, 0.5 H), 8.34 (d, *J* = 9.2 Hz, 0.5 H), 8.30 (d, *J* = 1.6 Hz, 0.5 H), 7.41–7.28 (m, 4H), 7.24–7.14 (m, 4H). The crude formamide **SI-2c** (920 mg, 4.27 mmol), Et_3N (4.76 mL, 34.2 mmol), anhydrous THF (8.5 mL), and $POCl_3$ (0.80 mL, 8.55 mmol) gave after purification by chromatography (hexane → hexane/EtOAc 97.5:2.5) **1c** (710 mg, 84% over two steps) as a green oil; ^{1}H NMR (400 MHz, $CDCl_3$) δ 7.51–7.44 (m, 4H, Ph), 7.41–7.36 (m, 2H, Ph), 7.17 (t, *J* = 8.8 Hz, 2H); ^{13}C NMR (101 MHz, $CDCl_3$) δ 166.9 (NC), 162.9 (d, *J* = 249.1 Hz, C-F), 137.9 (C_{ipso}), 133.1 (d, *J* = 3.6 Hz, C_{ipso}), 130.9 (d, *J* = 8.3 Hz, Ph), 130.6 (Ph), 129.7 (Ph), 128.4 (Ph), 127.9 (Ph), 115.7 (d, *J* = 21.5 Hz, Ph); ^{19}F NMR (376 MHz, $CDCl_3$) δ −113.38 (dd, *J* = 13.9, 8.6, 5.6 Hz). Spectral data were identical to those previously reported.[1]

2-isocyano-4-methoxy-1,1'-biphenyl (1d)

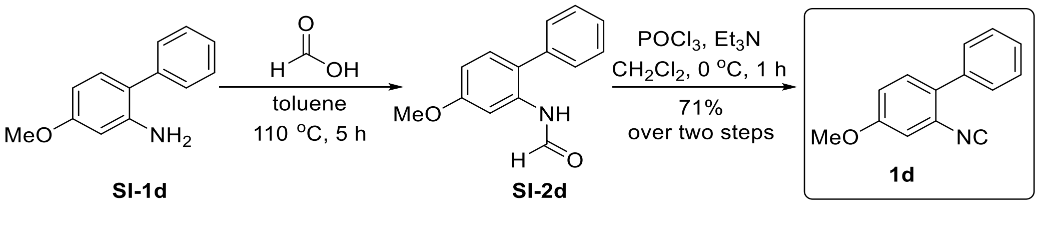

According to the general procedure for the preparation of isocyanides **SI-1d** (600 mg, 3.01 mmol) and formic acid (98%, 0.51 mL, 13.5 mmol) in toluene (1 M, 3 mL) gave formamide **SI-2d** as a yellowish oil; ^{1}H NMR (400 MHz, $CDCl_3$) δ 8.67 (d, *J* = 11.6 Hz, 0.5 H), 8.28 (d, *J* = 2 Hz, 0.5 H), 8.08 (d, *J* = 2.8 Hz, 0.5 H), 7.49–7.22 (m, 5H), 7.14 (d, *J* = 8.4 Hz, 1 H), 6.83–6.74 (m, 2H), 3.86 (s, 3H). To a solution of the crude formamide **SI-2d** (684 mg, 3.01 mmol), Et_3N (3.35 mL, 24.1 mmol), anhydrous THF (6 mL) and $POCl_3$ (0.56 mL, 6.02 mmol) gave after purification by chromatography (hexane →

hexane/EtOAc 95:5) **1d** (630 mg, 71% over two steps) as a pale green solid; ^{1}H NMR (400 MHz, $CDCl_3$) δ 7.52–7.46 (m, 4H, Ph), 7.43–7.39 (m, 1H, Ph), 7.36–7.33 (m, 1H, Ph), 7.04–7.01 (m, 2H, Ph), 3.86 (s, 3H, Me); ^{13}C NMR (101 MHz, $CDCl_3$) δ 166.4 (NC), 159.1 (C_{ipso}), 136.9 (C_{ipso}), 131.5 (Ph), 131.4 (C_{ipso}), 129.0 (Ph), 128.6 (Ph), 127.9 (Ph), 116.2 (Ph), 112.8 (Ph), 55.8 (Me). Spectral data were identical to those previously reported.[1]

2-isocyano-5-(trifluoromethyl)-1,1'-biphenyl (1e)

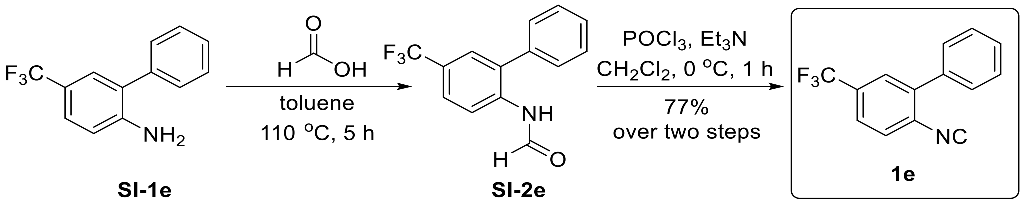

According to the general procedure for the preparation of isocyanides **SI-1e** (850 mg, 3.58 mmol) and formic acid (98%, 0.61 mL, 16.1 mmol) in toluene (1 M, 3.60 mL) gave formamide **SI-2e** as a white solid; ^{1}H NMR (400 MHz, $CDCl_3$) δ 8.81 (d, *J* = 11.2 Hz, 0.5 H), 8.60 (d, *J* = 8.8 Hz, 0.5 H), 8.34 (d, *J* = 2 Hz, 0.5 H), 7.63 (d, *J* = 8.4 Hz, 1H), 7.57–7.47 (m, 3H), 7.43–7.33 (m, 2H), 7.28–7.24 (m, 1H), 7.19–7.14 (m, 1H). To a solution of the crude formamide **SI-2e** (950 mg, 3.58 mmol), Et_3N (4 mL, 28.7 mmol), anhydrous THF (7.2 mL) and $POCl_3$ (0.67 mL, 7.17 mmol) gave after purification by chromatography (hexane → hexane/EtOAc 97.5:2.5) **1e** (684 mg, 77% over two steps) as a brownish oil; ^{1}H NMR (400 MHz, $CDCl_3$) δ 7.72 (s, 1H, Ph), 7.67–7.61 (m, 2H, Ph), 7.54–7.48 (m, 5H, Ph); ^{13}C NMR (101 MHz, $CDCl_3$) δ 169.6 (NC), 139.8 (C_{ipso}), 135.7 (C_{ipso}), 131.6 (q, *J* = 33.2 Hz, C-F_3), 129.2 (Ph), 129.0 (Ph), 128.9 (Ph), 128.5 (Ph), 127.9 (q, *J* = 4 Hz, Ph), 125.2 (q, *J* = 3.6 Hz, Ph), 124.7 (Ph), 122.0 (Ph); ^{19}F NMR (376 MHz, $CDCl_3$) δ −62.91 (s) ppm. Spectral data were identical to those previously reported.[1]

Methyl 2-isocyano-3,3-diphenylacrylate (2)

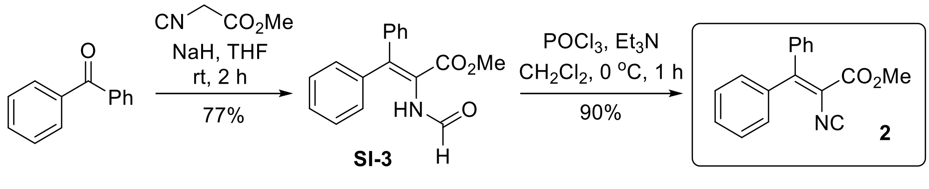

To a solution of NaH (90%, 88 mg, 3.29 mmol) in THF (2.7 mL), a mixture of benzophenone (500 mg, 2.74 mmol) and methyl isocyanoacetate (272 mg, 2.74 mmol) in THF (2.7 mL) at room temperature was added and stirred for 2 h. The reaction was quenched by adding a 10% AcOH aq. solution at 0 °C until there is no hydrogen release. The solvent was removed under reduced pressure and extracted with CH_2Cl_2 (3 × 10 mL). The organic layer was washed with water and brine, dried, concentrated, and recrystallized with MeOH to give **SI-3** (594 mg, 77%) as a white solid. ^{1}H NMR (400 MHz, CDCl3) δ 7.45–7.35 (m, 8H, Ph), 7.17–7.15 (m, 2H, Ph), 3.68 (s, 3H, Me); ^{13}C NMR (101 MHz, $CDCl_3$) δ 169.9 (C-1), 162.3 (NC), 154.5 (C-3), 137.8 (C_{ipso}), 137.4 (C_{ipso}), 130.3 (Ph), 129.9 (Ph), 129.6 (C-2), 129.1 (Ph), 128.5 (Ph), 128.3 (Ph), 52.9 (Me). The

formamide **SI-3** (300 mg, 1.07 mmol) was dissolved in anhydrous CH_2Cl_2 (2.2 mL) with Et_3N (1.2 mL, 8.53 mmol) and cooled to 0 °C. Then, $POCl_3$ (200 μL, 2.13 mmol) was added dropwise, and the mixture was stirred for 1 h at 0 °C. The mixture was quenched by slowly adding a saturated aq. Na_2CO_3 solution (3 mL) and the mixture was stirred for 1 h. The mixture was extracted with CH_2Cl_2, washed with water and brine, and dried and concentrated. Purification by chromatography (hexane → hexane/EtOAc 90:10) gave **2** (254 g, 90%) as a brownish solid; ^{1}H NMR (400 MHz, $CDCl_3$) δ 7.45–7.35 (m, 8H, Ph), 7.17–7.15 (m, 2H, Ph), 3.68 (s, 3H, Me); ^{13}C NMR (101 MHz, $CDCl_3$) δ 169.9 (C-1), 162.3 (NC), 154.5 (C-3), 137.8 (C_{ipso}), 137.4 (C_{ipso}), 130.3 (Ph), 129.9 (Ph), 129.6 (C-2), 129.1 (Ph), 128.5 (Ph), 128.3 (Ph), 52.9 (Me). Spectral data were identical to those previously reported.[8]

3-(2-Isocyanophenyl)-1-phenylprop-2-en-1-one (3)

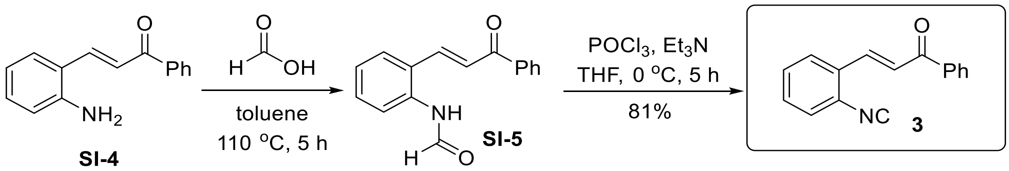

According to the general procedure for the preparation of isocyanides, **SI-4** (1.15 g, 5.15 mmol) and formic acid (98%, 0.9 mL, 23.2 mmol) in toluene (1 M, 5.2 mL) gave **SI-5** as yellow solid as a mixture of rotamers; ^{1}H NMR (400 MHz, $CDCl_3$) δ 8.54–8.48 (m, 1H), 8.09–7.98 (m, 3H), 7.72 (dd, J = 18.4, 7.6 Hz, 1H), 7.57–7.42 (m, 5H), 7.34–7.21 (m, 1H), 5.29 (s, 1H). The crude formamide **SI-5** (1.16 g, 4.64 mmol), Et_3N (2.6 mL, 18.6 mmol), anhydrous THF (9.3 mL), and $POCl_3$ (651 μL, 6.96 mmol) gave after purification by chromatography (hexane → hexane/EtOAc 75:25) **3** (873 mg, 81%) as a brownish solid; ^{1}H NMR (400 MHz, $CDCl_3$) δ 8.05–8.02 (m, 2H, Ph), 8.03 (d, J = 16 Hz, 1H, H-3), 7.79–7.76 (m, 1H, Ph), 7.63–7.59 (m, 1H, Ph), 7.62 (d, J = 16 Hz, 1H, H-2), 7.54 (s, 1H, Ph), 7.52 (s, 1H, Ph), 7.47 (t, J = 2.4 Hz, 1H, Ph), 7.45 (m, 2H, Ph); ^{13}C NMR (101 MHz, $CDCl_3$) δ 189.9 (C-1), 169.1 (NC), 137.9 (C-3), 137.6 (C_{ipso}), 133.1 (Ph), 131.3 (C_{ipso}), 130.7 (Ph), 129.6 (Ph), 128.7 (Ph), 128.6 (Ph), 127.9 (Ph), 127.5 (Ph), 126.1 (C-2). Spectral data were identical to those previously reported.[2]

Ethyl 3-(2-isocyanophenyl)acrylate (4)

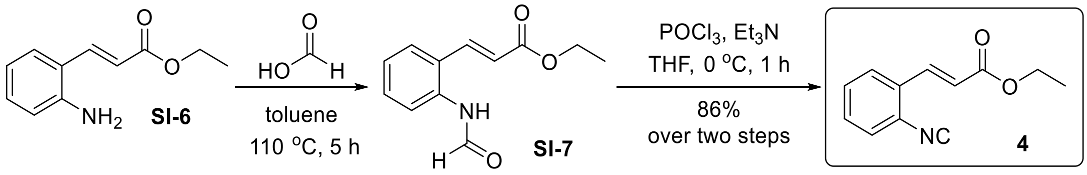

According to the general procedure for the preparation of isocyanides, **SI-6** (3.1 g, 16.2 mmol) and formic acid (98%, 2.8 mL, 72.9 mmol) in toluene (1 M, 16 mL) gave **SI-7** as yellow solid as a mixture of rotamers; ^{1}H NMR (400 MHz, $CDCl_3$) δ 8.94 (d, J = 10.4 Hz, 0.5H), 8.48–8.43 (m, 1H), 7.93–7.87 (m, 1H), 7.55 (dd, J = 25.2, 8 Hz, 1H), 7.39–7.31 (m, 1H), 7.24 (t, J = 7.6 Hz, 0.5 Hz), 7.19–7.12 (m, 1H), 6.37 (dd, J = 16, 10.8 Hz, 1H), 4.19 (q, J = 14.4, 7.2 Hz, 2H), 1.26 (q, J = 14.8, 7.2 Hz, 3H). The crude formamide **SI-7** (3.55 g, 16.2 mmol), Et_3N (18 mL, 0.13 mol), anhydrous THF (33 mL) and $POCl_3$ (3.03 mL, 32.4 mmol) gave after purification by chromatography (hexane → hexane/EtOAc 95:5) **4** (2.79 g, 86%) as a brownish solid; ^{1}H NMR (400 MHz, $CDCl_3$) δ 7.96 (d, J = 16.4

Hz, 1H, H-3), 7.67–7.65 (m, 1H, Ph), 7.45–7.40 (m, 3H, Ph), 6.53 (d, J = 16 Hz, 1H, H-2), 4.29 (q, J = 14.4, 7.2 Hz, 2H, $OC\underline{H}_2CH_3$), 1.35 (t, J = 7.2 Hz, 3H, $OCH_2C\underline{H}_3$); ^{13}C NMR (101 MHz, $CDCl_3$) δ 168.8 (C-1), 165.9 (NC), 137.6 (C-3), 130.8 (C_{ipso}), 130.6 (Ph), 129.6 (Ph), 127.7 (Ph), 126.9 (Ph), 122.5 (C-2), 60.9 ($O\underline{C}H_2CH_3$), 14.2 ($OCH_2\underline{C}H_3$). Spectral data were identical to those previously reported.[3]

1-Isocyano-2-(3-methoxyprop-1-en-1-yl)benzene (5)

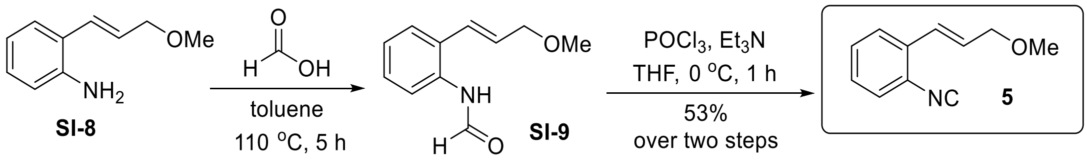

According to the general procedure for the preparation of isocyanides, **SI-8** (107 mg, 0.66 mmol) and formic acid (98%, 113 μL, 2.95 mmol) in toluene (1 M, 0.7 mL) gave **SI-9** as a yellow solid. The crude formamide **SI-9** (125 mg, 0.65 mmol), Et_3N (730 μL, 5.23 mmol), anhydrous THF (1.3 mL) and $POCl_3$ (122 μL, 1.31 mmol) gave after purification by chromatography (hexane → hexane/EtOAc 90:10) **5** (60 mg, 53%) as a brownish oil; ^{1}H NMR (400 MHz, $CDCl_3$) δ 7.60–7.58 (m, 1H, Ph), 7.36 (t, J = 6.8 Hz, 2H, Ph), 7.28–7.24 (m, 1H, Ph), 6.93 (d, J = 16 Hz, 1H, H-1), 6.40 (dt, J = 16, 6 Hz, 1H, H-2), 4.15 (dd, J = 6, 1.6 Hz, 2H, H-3), 3.42 (s, 3H, Me); ^{13}C NMR (101 MHz, $CDCl_3$) δ 166.8 (NC), 160.6 (C_{ipso}), 133.1 (C_{ipso}), 130.7 (C-2), 129.4 (Ph), 128.2 (Ph), 127.1 (Ph), 126.0 (Ph), 125.9 (C-1), 72.7 (C-3), 58.3 (Me). Spectral data were identical to those previously reported.[4]

Screening table of conditions for reductive couplings

Table S1. Phenanthridines reductive coupling

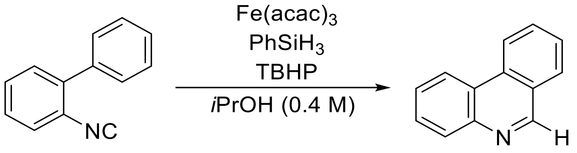

1a 6

Entry	$Fe(acac)_3$	$PhSiH_3$	TBHP	T°	time	6
**1**	0.2	1	1.5 [a]	50 °C	2 h	43%
**2**	0.2	2.5	1.5 [b]	50 °C	2 h	40%
**3**	0.2 [c]	1	1.5 [b]	50 °C	2 h	55%
**4[d]**	0.2	1	1.5 [b]	rt	2 h	23%
**5**	0.2	1	1.5[a]	rt	24 h	51%
**6**	0.2	3	1.5[a]	rt	24 h	74%
**7**	0.2	3	1.5[a]	50 °C	24 h	62%
**8**	1	3	-	rt	2 h	-
**9**	0.2	3	1[a]	rt	2 h	50%
**10**	1	3	1[a]	rt	2 h	44%
**11**	0.2	3	3[a]	rt	2 h	40%

[a]TBHP 70% in water. [b]TBHP 5.5 M in decane. [c]$Fe(acac)_2$ instead of $Fe(acac)_3$. [d]*i*PrOH 0.04 M.

Table S2. Isoquinolines reductive coupling

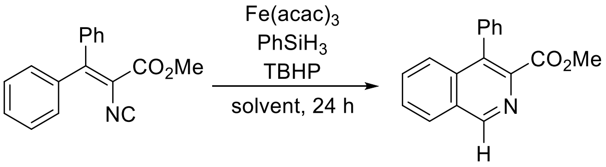

2 7

Entry	9	$Fe(acac)_3$	$PhSiH_3$	TBHP	solvent	T°	7
**1**	1	0.2	1	1.5[a]	*i*PrOH 0.4 M	50 °C	9%
**2**	1	0.2	1	1.5[a]	THF 0.4 M[b]	50 °C	9%
**3**	1	0.2	1	1.5[a]	*i*PrOH 0.4 M	rt	34%
**4**	1	0.2[c]	1	1.5[d]	*i*PrOH 0.4 M	rt	51%
**5**	1	0.2	1	1.5[d]	*i*PrOH 0.4 M	rt	83%
**6**	1	0.2	3	1.5[d]	*i*PrOH 0.4 M	rt	86%

[a]TBHP 5.5 M in decane. [b]MeOH (10 equiv) were added. [c]$Fe(acac)_2$ was used instead of $Fe(acac)_3$. [d]TBHP 70% in water.

Table S3. Indoles reductive coupling

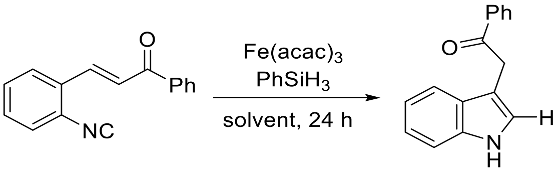

3 8

Entry	3	$Fe(acac)_3$	$PhSiH_3$	solvent	T°	8
**1**	1	0.2[a]	1	*i*PrOH 0.4 M	rt	-
**2**	1	0.2	1	*i*PrOH 0.04 M	rt	47%
**3**	1	0.05	1	EtOH 0.04 M	60 °C	11%
**4**	1	0.05/0.15[b]	1	*i*PrOH 0.04 M	rt	37%
**5**	1	0.2	3	THF 0.04 M[c]	60 °C	75%

[a]$Fe(acac)_2$ was used instead of $Fe(acac)_3$. [b]A combination of $Fe(acac)_3$ and $Fe(acac)_2$ was used. [c]MeOH (10 equiv) were added.

Screening table of conditions for alkene MHAT couplings

Table S4. Optimization table for the formation of phenanthridine 6a

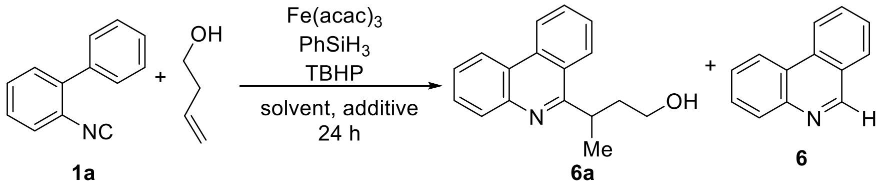

1a **6a** **6**

Entry	1:a	Fe	$PhSiH_3$	TBHP	base	solvent	T°	6a	6
**1**	1:1	1.0	2.5	-	-	EtOH 0.04 M	rt	9%	-
**2**	1:1	0.2	2.5	-	-	EtOH 0.04 M	rt	20%	-
**3**	1:1	0.2	2.5	-	-	THF 0.04 M[a]	rt	19%	
**4**	1:1	2.0	2.5	-	-	THF 0.04 M[a]	rt	20%	-
**5**	1:1	2.0	2.5	-	-	*t*-BuOH 0.04M	rt	6%	-
**6**	1:1	2.0	2.5	-	-	MeCN 0.04 M	rt	5%	-
**7**	1:1	0.4	2.5	-	-	MeCN 0.04 M	90 °C	29%	-
**8**	1:1	0.2	1	1.5	-	MeCN 0.4 M	60 °C	18%	-
**9**	1:1	0.4	2.5	1.5	-	*i*PrOH 0.4 M	rt	30%	30%
**10**	1:1	0.4	1	1.5	-	*i*PrOH 0.4 M	rt	40%	30%
**11**	1:1	0.2	1	1.5	-	*i*PrOH 0.4 M	rt	63%	25%
**12**	1:1[b]	0.2	1	1.5	-	*i*PrOH 0.4 M	rt	53%	3%
**13**	1:1	0.05	1	1.5	-	*i*PrOH 0.4 M	rt	41%	41%
**14**	1:1	0.2	0.5	1.5	-	*i*PrOH 0.4 M	rt	42%	22%
**15**	1:1	0.2	1[c]	1.5	-	*i*PrOH 0.4 M	rt	50%	17%
**16**[d]	1:1	0.2	1	1.5	-	*i*PrOH 0.4 M	rt	33%	11%
**17**	1:1	0.2[e]	1	1.5	-	*i*PrOH 0.4 M	rt	40%	50%
**18**	1:1	0.2	1	1.5	-	*i*PrOH 0.4 M	60 °C	75%	13%
**19**	1:1	0.2[f]	1	1.5	-	*i*PrOH 0.4 M	60 °C	23%	72%
**20**	1:1	0.2	1	1.5	-	*i*PrOH 0.4 M	100 °C	53%	40%
**21**	2:1	0.2	1	1.5	-	*i*PrOH 0.4 M	60 °C	40%	35%
**22**	1:2	0.2	1	1.5	-	*i*PrOH 0.4 M	60 °C	40%	46%
**23**	1:1	0.2	1	3	-	*i*PrOH 0.4 M	60 °C	17%	22%
**24**	1:1	0.2	1	0.5	-	*i*PrOH 0.4 M	60 °C	39%	50%
**25**	1:1	0.2	1	1.5[g]	-	*i*PrOH 0.4 M	60 °C	42%	10%
**26**	1:1	0.2	1	1.5	Na_2HPO_4 (1 equiv)	*i*PrOH 0.4 M	60 °C	31%	29%
**27**	1:1	0.2	1	1.5	DBU (0.5 equiv)	*i*PrOH 0.4 M	60 °C	-	-
**28**	1:1	0.2	1	1.5	Na_2CO_3 (1.5 equiv)	*i*PrOH 0.4 M	60 °C	18%	32%
**29**	1:1	0.2	1	1.5	$NaHCO_3$ (1 equiv)	*i*PrOH 0.4 M	60 °C	33%	45%
**30**	1:1	0.2	1	1.5	-	*t*-BuOH 0.4 M	60 °C	43%	29%
**31**	1:1	0.05	1	1.5	-	*t*-BuOH 0.4 M	60 °C	54%	36%
**32**	1:1	0.2	5.5[h]	1.5	-	*t*-BuOH 0.4 M	60 °C	26%	26%
**33**	1:1	0.4	1	-	-	*i*PrOH 0.4 M	rt	38%	20%
**34**	1:1	0.2	1	1.5	-	THF[a]	60 °C	23%	52%
**35**[i]	1:1	0.2	1	1.5	-	*i*PrOH 0.4 M	rt	32%	30%
**36**	1:1	0.4	1	1.5	-	*i*PrOH 0.2 M	rt	30%	40%
**37**	1[j]:1	0.2	1	1.5	-	*i*PrOH 0.4 M	60 °C	-	-

[a]MeOH (10 equiv) was added. [b]Addition of isocyanide by syringe pump. [c]Addition of $PhSiH_3$ by syringe pump. [d]Without argon purge. [e]$Fe(dibm)_3$ instead of $Fe(acac)_3$ was used. [f]$Fe(acac)_2$ instead of $Fe(acac)_3$ was used. [g]DTBP was used as oxidant instead of TBHP. [h]PMHS (5.5 equiv) used instead of $PhSiH_3$. [i]4 h instead of 24 h. [j]**6** was used instead of **1** as starting material.

Table S5. Optimization table for the formation of phenanthridine 6f via Minisci coupling

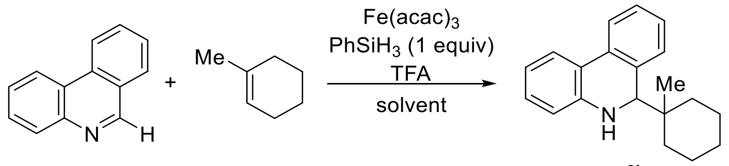

Entry	1:alkene	Fe(acac)$_3$	TBHP	Acid	solvent	T°	time	6l
**1**	1:3	1	-	2[a]	THF/MeOH 0.2 M	60 °C	2 h	40%
**2**	1:3	1	-	2	THF/MeOH 0.2 M	60 °C	2 h	86%
**3**	1:3	0.2	1.5	2[a]	THF/MeOH 0.2 M	60 °C	2 h	12%
**4**	1:3	0.2	1.5	2	THF/MeOH 0.2 M	rt	2 h	10%
**5**	1:3	0.4	1	2	*i*PrOH 0.2 M	rt	2 h	31%
**6**	1:3	0.4	1	2	CH_2Cl_2 0.2 M	40 °C	7 h	25%
**7**	1:3	0.4	1.5	2	Toluene/MeOH 0.2M	60 °C	24 h	42%
**8**	1:3	0.4	1.5	2	MeCN/MeOH 0.2 M	60 °C	24 h	26%
**9**	1:3	0.4	1.5	2	*t*-BuOH/MeOH 0.2M	60 °C	24 h	35%
**10**	1:3	0.4	1.5	2	DMF/MeOH 0.2 M	60 °C	24 h	17%
**11**	1:3	0.4	1.5	2	DCE/MeOH 0.2 M	60 °C	24 h	37%
**12**	1:3	1	1.5	2	DCE/MeOH 0.2 M	60 °C	24 h	29%
**13[b]**	1:3	0.4	1.5	2	DCE/MeOH 0.2 M	60 °C	24 h	38%
**14**	1:3	0.2	1.5	2	EtOH 0.2 M	60 °C	24 h	63%
**15**	1:3	0.2	1.5	2	DCE 0.2 M	60 °C	24 h	34%
**16**	1:3	0.2	1.5	2	DCE/MeOH 0.2 M	60 °C	24 h	35%
**17**	1:3	1	-	2	*i*PrOH 0.2 M	60 °C	3 h	30%
**18**	1:3	0.2[c]	1.5	2	THF/MeOH 0.2 M	rt	2 h	11%
**19[d]**	1:3	0.2[c]	1.5	2	THF/MeOH 0.2 M	rt	2 h	-
**20[d]**	1:3	0.2:0.2[c]	1.5	2	THF/MeOH 0.2 M	rt	4 h	-
**21[d]**	1:3	0.2[c]	1.5	2	THF/MeOH 0.2 M	rt	4 h	-
**22[e]**	1:3	1	-	2	THF/MeOH 0.2 M	60 °C	2 h	-
**23**	1:3	1	1.5[f]	2	THF/MeOH 0.2 M	60 °C	4 h	45%
**24[g]**	1:3	0.2	-	2	THF/MeOH 0.2 M	60 °C	24 h	31%
**25[h]**	1:3	1	-	2	THF/MeOH 0.2 M	60 °C	2 h	-
**26**	1:3	0.2	-	2	THF/MeOH 0.2 M	60 °C	2 h	25%
**27**	1:3	1	-	2	THF/MeOH 0.2 M	60 °C	24 h	44%
**28**	1:3	1	-	-	THF/MeOH 0.2 M	60 °C	24 h	5%
**29[g]**	1:3	1	-	2	THF/MeOH 0.2 M	60 °C	2 h	94%

[a]$BF_3 \cdot Et_2O$ added instead [b]2.5 equiv of $PhSiH_3$ was added. [c] $Fe(acac)_2$ was used instead. [d]No $PhSiH_3$ was added. [e]After 2 h chloranil (2 equiv) was added. [f]TBHP was added after 2 h of reaction. [g]Without argon purge. [h]MnO_2 added after the reaction is completed.

Table S6. Optimization table for the formation of phenanthridine 6f via sequential MHAT cyclization-Minisci coupling from isocyanide 1

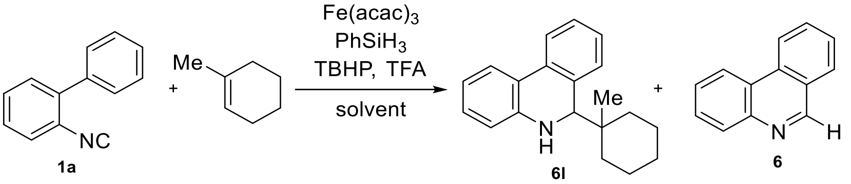

Entry	1:alk	Fe(acac)$_3$	PhSiH$_3$	TBHP	TFA	solvent	T°	time	6l	1
**1**	1:3	1	1	-	2[a]	THF/MeOH 0.2 M	60 °C	2 h	-	-
**2[b]**	1:3	1	1	-	2[a]	THF/MeOH 0.2 M	60 °C	2 h	30%	-
**3[c]**	1:3	2	2.5	-	2	*i*PrOH 0.2 M	60 °C	2 h	20%	-
**4[d]**	1:3	1	3	1	2	MTBE/MeOH 0.2 M	rt to 60 °C	2.5 h	56%	-

[a]$BF_3 \cdot Et_2O$ added instead. [b]Acid was added 1 hour later. [c]Acid and alkene were added 10 min after the addition of $PhSiH_3$. [d]See Method 3- Sequential MHAT-Minisci Coupling from the corresponding isocyanide (SI-14).

Table S7. Optimization table for the formation of isoquinoline 7a

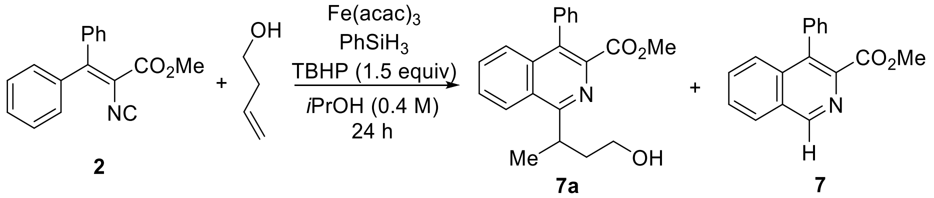

2 **7a** **7**

Entry	9:a	$Fe(acac)_3$	$PhSiH_3$	T°	7a	7
**1**[a]	1:1	0.2	1	60 °C	23%	29%
**2**[a,b]	1:1	0.2	1	60 °C	17%	-
**3**[a]	1:1	0.2	3	60 °C	21%	40%
**4**[a,b]	1:1	0.2	3	rt	46%	38%
**5**[a]	1:1	0.2	1	rt	38%	40%
**6**[a]	1:1	0.4	3	rt	33%	57%
**7**[a]	1:1	0.1	3	rt	25%	54%
**8**[c]	1:1	0.2	3	rt	53%	22%
**9**[a]	1:1	0.2	3	rt	61%	27%

[a]TBHP 70% in water. [b]*i*PrOH (0.04 M). [c]TBHP 5.5 M in decane.

Table S8. Optimization table for the formation of indole 8a

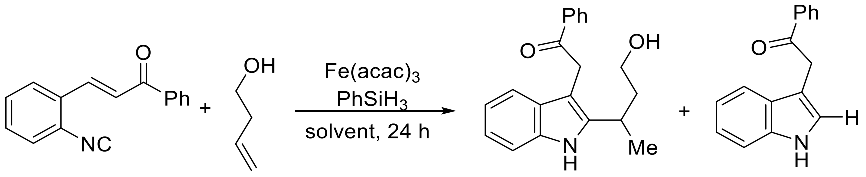

3 **8a** **8**

Entry	3:a	$Fe(acac)_3$	$PhSiH_3$	solvent	T°	8a	8
**1**	1:1	0.2	1	*i*PrOH 0.4 M	rt	-	22%
**2**	1:1	0.4	1	*i*PrOH 0.04 M	rt	15%	29%
**3**	1:1	1	1	THF 0.04 M[a]	rt	23%	33%
**4**	1:1	0.05	5	*i*PrOH 0.04 M	rt	5%	40%
**5**	1:1	1	3	THF 0.04 M[a]	rt	9%	47%
**6**	1:1	1	3	DCE 0.04 M[a]	rt	19%	58%
**7**	1:1	0.2	3	THF 0.04 M[a]	60 °C	-	80%
**8**	1:1[b]	0.4[c]	1	THF 0.04 M[a]	rt	8%	37%
**9**	1:1	0.4	1	EtOH/EG (5:1) 0.2 M	rt	16%	28%
**10**	1:3	0.1	5.5[d]	ChCl/EG (1:2) 0.2 M	60 °C	8%	11%
**11**	1:3	0.4[e]	1	*i*PrOH/DCE (1:1) 0.2 M	60 °C	11%	73%
**12**	1:1	0.2	3	EtOH 0.04 M	rt	10%	55%
**13**	1:3	0.4	3	*i*PrOH 0.04 M	rt	15%	41%
**14**	1:1	0.1	1	THF 0.04 M[a]	rt	11%	43%
**15**	1:1	0.05[c]	1	THF 0.04 M[a]	rt	19%	59%
**16**	2:1[f]	1	1	THF 0.04 M[a]	rt	18%	57%
**17**	1:1	2	1	THF 0.04 M[a]	rt	14%	38%
**18**	1:1	1	1	THF 0.04 M[g]	rt	9%	58%
**19**	1:1	1	1	THF 0.004 M[a]	rt	31%	40%
**20**	1:1	1	1	THF 0.04 M[a]	0 °C	21%	38%
**21**	1:3	1	1	THF 0.02 M[h]	0 °C	9%	33%
**22**	1:1	1	1	*i*PrOH 0.02 M	rt	14%	35%
**23**	1:1	0.2[i]	1	*i*PrOH 0.04 M	rt	-	44%
**24**	1:1	0.2[j]	1	*i*PrOH 0.04 M	rt	-	-
**25**	1:1	0.2	1	$MeCN/H_2O$ (1:1) (0.0125 M)	rt	-	21%
**26**	1:1	0.2	1	*i*PrOH 0.04 M	rt	47%	16%

[a]MeOH (10 equiv) were added. [b]4-phenylbutene used as alkene. [c]$Fe(acac)_2$ (0.4 equiv) were added. [d]PMHS was used instead of $PhSiH_3$. [e]$Fe(dpm)_3$ was used instead of $Fe(acac)_3$. [f]Second equivalent of isocyanide was added 2 h later. [g]*i*PrOH (10 equiv) were added. [h]*i*PrOH (50 equiv) were added. [i]Co(salen) was used instead of $Fe(acac)_3$. [j]$Mn(dpm)_3$ was used instead of $Fe(acac)_3$.

Synthesis of core heterocycles via MHAT

Phenanthridine (6)

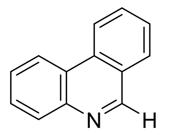

6

To a solution of isocyanide **1** (315 mg, 1.76 mmol, 1 equiv) and Fe(acac)$_3$ (124 mg, 0.35 mmol, 0.2 equiv) in *i*PrOH (0.4 M, 4.4 mL) was added TBHP (70%, 377 μL, 2.64 mmol, 1.5 equiv) and the mixture was degassed and bubbled with argon for 5 min. PhSiH$_3$ (570 mg, 5.27 mmol, 3 equiv) was added (<u>Caution</u>: continuous argon purge with outlet was maintained to avoid over-pressurization of the reaction flask). The reaction mixture was stirred at room temperature for 24 h. The mixture was concentrated and purified by chromatography (hexane → hexane/EtOAc 90:10) to give **6** (233 mg, 74%) as brownish solid; ^{1}H NMR (400 MHz, CDCl$_3$) δ 9.30 (s, 1H, H-6), 8.63 (d, *J* = 7.6 Hz, 1H, H-1), 8.59 (dd, *J* = 8.4, 1.6 Hz, 1H, H-10), 8.20 (dd, *J* = 8, 2 Hz, 1H, H-4), 8.06 (d, *J* = 8 Hz, 1H, H-7), 7.88 (ddd, *J* = 8.4, 7.2, 1.6 Hz, 1H, H-9), 7.78–7.68 (m, 3H, H-2, H-3 and H-8); ^{13}C NMR (101 MHz, CDCl$_3$) δ 153.5 (C-6), 144.3 (C-4a), 132.5 (C-10a), 131.0 (C-9), 130.0 (C-4), 128.8 (C-7), 128.7 (C-3), 127.5 (C-8), 127.1 (C-2), 126.3 (C-6a), 124.1 (C-10b), 122.2 (C-1), 121.8 (C-10). HRMS (ESI) *m/z*: $[M+H]^+$ calcd for $[C_{13}H_{10}N]^+$ 180.0813, found 180.0819. Spectral data were identical to those previously reported.[9]

3-Methoxycarbonyl-4-phenylisoquinoline (7)

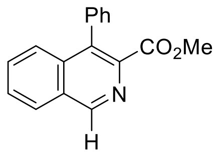

7

To a solution of isocyanide **2** (100 mg, 0.38 mmol,1 equiv) and Fe(acac)$_3$ (27 mg, 0.076 mmol, 0.2 equiv) in *i*PrOH (0.4 M, 1 mL), was added TBHP (70%, 81 μL, 0.57 mmol, 1.5 equiv) and the mixture was degassed and bubbled with argon for 5 min. PhSiH$_3$ (51 mg, 0.38 mmol, 1 equiv) was added, and the reaction mixture was stirred at room temperature for 24 h. The reaction mixture was concentrated and purified by chromatography (hexane → hexane/EtOAc 75:25) to give **7** (86 mg, 86%) as a brownish oil; ^{1}H NMR (400 MHz, CDCl$_3$) δ 9.32 (s, 1H, H-1), 8.07 (d, *J* = 8 Hz, 1H, H-8), 7.72–7.61 (m, 3H, H-5, H-6, H-7), 7.53–7.47 (m, 3H, Ph), 7.34–7.32 (m, 2H, Ph), 3.74 (s, 3H, Me); ^{13}C NMR (101 MHz, CDCl$_3$) δ 167.0 (C=O), 151.7 (C-1), 140.9 (C-3), 135.9 (C_{ipso}), 135.7 (C-4), 134.9 (C-4a), 131.1 (C-6), 129.5 (Ph), 129.0 (C-8a), 128.8 (C-7), 128.2 (Ph), 127.9 (Ph), 127.6 (C-8), 126.5 (C-5), 52.4 (Me). HRMS (ESI) *m/z*: $[M+H]^+$ calcd for $[C_{17}H_{14}NO_2]^+$ 264.1024, found 264.1025. Spectral data were identical to those previously reported.[10]

3-(2-Oxo-2-phenylethyl)indole (8)

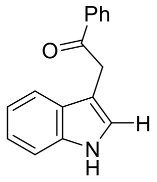

8

Isocyanide **3** (100 mg, 0.43 mmol, 1 equiv) and Fe(acac)$_3$ (30 mg, 0.086 mmol, 0.2 equiv) were dissolved in THF (0.04 M, 11 mL) and MeOH (174 μL, 4.29 mmol, 10 equiv) degassed and bubbled with argon for 5 min. PhSiH$_3$ (139 mg, 1.29 mmol, 3 equiv) was added, and the reaction mixture was stirred at 60 °C using a heating block for 24 h. The reaction mixture was concentrated and purified by chromatography (hexane → hexane/EtOAc 75:25) to give **8** (76 mg, 75%) as brownish oil ; ^{1}H NMR (400 MHz, CDCl$_3$) δ 8.11 (br s, 1H, NH), 8.05 (d, *J* = 7.2 Hz, 2H, Ph), 7.61 (d, *J* = 8 Hz, 1H, H-4), 7.54 (t, *J* = 7.2 Hz, 1H, Ph), 7.44 (t, *J* = 8 Hz, 2H, Ph), 7.33 (d, *J* = 8 Hz, 1H, H-7), 7.19 (t, *J* = 7.2 Hz, 1H, H-6), 7.13 (t, *J* = 8 Hz, 1H, H-5), 7.10 (s, 1H, H-2), 4.41 (s,

2H, CH_2); ^{13}C NMR (101 MHz, $CDCl_3$) δ 197.9 (C=O), 136.7 (C-7a), 136.1 (C_{ipso}), 132.9 (Ph), 128.6 (Ph), 128.5 (Ph), 127.3 (C-3a), 123.2 (C-2), 122.1 (C-6), 119.6 (C-5), 118.7 (C-4), 111.2 (C-7), 108.8 (C-3), 35.5 (CH_2). HRMS (ESI) *m/z*: $[M+H]^+$ calcd for $[C_{16}H_{14}NO]^+$ 236.1075, found 236.1076. Spectral data were identical to those previously reported.[11]

Ethyl 3-Indoleacetate (9)

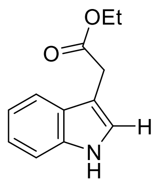

9

Isocyanide **4** (100 mg, 0.50 mmol,1 equiv), $Fe(acac)_3$ (35 mg, 0.10 mmol, 0.2 equiv) were dissolved in THF (0.04 M, 12.4 mL), and MeOH (201 μL, 4.97 mmol, 10 equiv), degassed and bubbled with argon for 5 min. $PhSiH_3$ (161 mg, 1.49 mmol, 3 equiv) was added and the reaction mixture was stirred at 60 °C using a heating block for 24 h The reaction mixture was concentrated and purified by chromatography (hexane → hexane/EtOAc 75:25) to give **9** (92 mg, 91%) as a brownish oil; ^{1}H NMR (400 MHz, $CDCl_3$) δ 8.17 (br s, 1H, NH), 7.64 (d, *J* = 8.4 Hz, 1H, H-4), 7.32 (dt, *J* = 8, 1.2 Hz, 1H, H-7), 7.21 (ddd, *J* = 8.4, 7.2, 1.2 Hz, 1H, H-6), 7.15 (ddd, *J* = 8, 7.2, 1.6 Hz, 1H, H-5), 7.10 (d, *J* = 2.4 Hz, 1H, H-2), 4.19 (q, *J* = 7.2 Hz, 2H, $C\underline{H}_2CH_3$), 3.79 (s, 2H, CH_2), 1.28 (t, *J* = 6.8 Hz, 3H, $CH_2C\underline{H}_3$); ^{13}C NMR (101 MHz, $CDCl_3$) δ 172.3 (C=O), 136.2 (C-7a), 127.3 (C-3a), 123.2 (C-2), 122.2 (C-6), 119.7 (C-5), 118.9 (C-4), 111.3 (C-7), 108.5 (C-3), 60.9 ($\underline{C}H_2CH_3$), 31.5 (CH_2), 14.3 ($CH_2\underline{C}H_3$). HRMS (ESI) *m/z*: $[M+H]^+$ calcd for $[C_{12}H_{14}NO_2]^+$ 204.1024, found 204.1022. Spectral data were identical to those previously reported.[12]

Synthesis of coupled products via MHAT.
General Methods

Method 1a – MHAT Coupling from the corresponding isocyanide.
To a solution of isocyanide (1 equiv), alkene (1 equiv), and $Fe(acac)_3$ (0.2 equiv) in *i*PrOH (0.4 M) was added TBHP (70% in water, 1.5 equiv) and the mixture was degassed and bubbled with argon for 5 minutes. The mixture was adjusted to the indicated temperature using a heating block, and $PhSiH_3$ (1 equiv) was then added via syringe. After 24 h at this temperature, the reaction mixture was concentrated and purified by column chromatography.

Method 1b – MHAT Coupling from the corresponding isocyanide.
A solution of isocyanide (1 equiv), alkene (1 equiv), and $Fe(acac)_3$ (0.2 equiv) in *i*PrOH (0.04 M) was degassed and bubbled with argon for 5 minutes. The mixture was adjusted to the indicated temperature using a heating block, and $PhSiH_3$ (1 equiv) was then added via syringe. After 24 h at this temperature, the reaction mixture was concentrated and purified by column chromatography.

Method 2 – MHAT-Minisci Coupling from the corresponding heterocycle.
To a solution of the heterocycle (1 equiv), alkene (3 equiv), and $Fe(acac)_3$ (1 equiv) in 4:1 THF/MeOH (0.2 M) was added TFA (2 equiv). The mixture was adjusted to the indicated temperature using a heating block, and $PhSiH_3$ (1 equiv) was added via syringe and stirred for 3 h open to the air. The reaction was quenched by the addition of saturated aq. $NaHCO_3$ solution, extracted three times with EtOAc, and the combined organic

extracts were washed with brine, dried, concentrated, and purified by column chromatography.

Method 3 – Sequential MHAT-Minisci Coupling from the corresponding isocyanide.

To a solution of isocyanide (1 equiv) and $Fe(acac)_3$ (0.2 equiv) in 4:1 MTBE/MeOH (0.4 M) was added TBHP (70% in water, 1 equiv) and the mixture was degassed and bubbled with argon for 5 minutes. $PhSiH_3$ (3 equiv) was added via syringe, and the reaction mixture was stirred at room temperature for 15 min. Then, the reaction was opened to air, and MTBE (to a 0.2 M solution) was added, followed by $Fe(acac)_3$ (0.8 equiv), TFA (2 equiv), and alkene (3 equiv), and the reaction was heated to 60 °C using a heating block and stirred for 3 h. The reaction was quenched by the addition of saturated aq. $NaHCO_3$ solution, extracted three times with EtOAc, and the combined organic extracts were washed with brine, dried, concentrated, and purified by column chromatography.

Scope of MHAT coupling-cyclization reaction

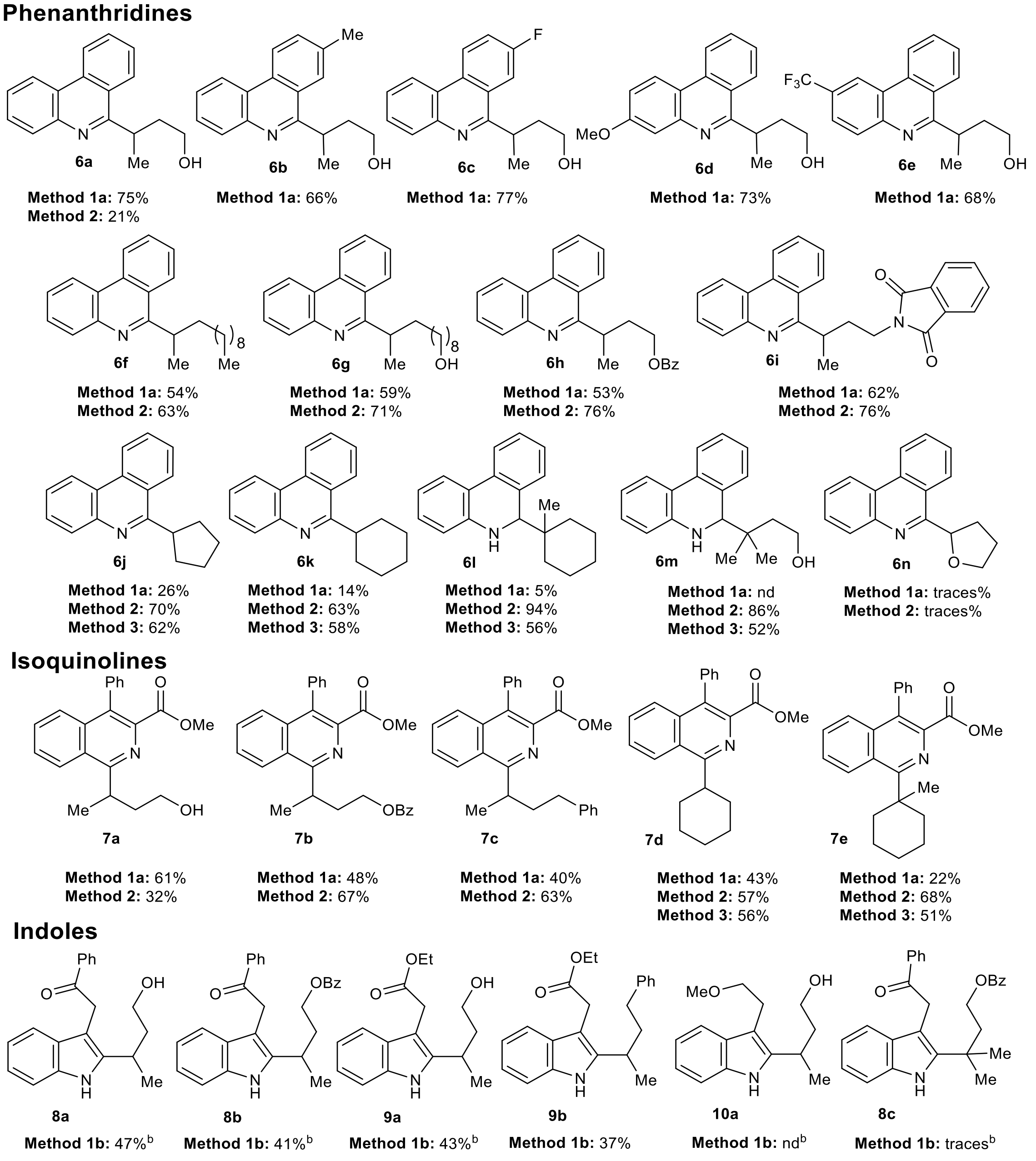

6-(4-Hydroxybutan-2-yl)phenanthridine (6a)

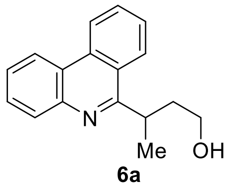

6a

Method 1a: Isocyanide **1a** (100 mg, 0.56 mmol), but-3-en-1-ol (40 mg, 0.56 mmol), $Fe(acac)_3$ (40 mg, 0.11 mmol), TBHP (70%, 120 μL, 0.84 mmol) and $PhSiH_3$ (60 mg, 0.56 mmol) in *i*PrOH (0.4 M, 1.4 mL) at 60 °C. Purification by chromatography (hexane → hexane/EtOAc 50:50) gave **6a** (105 mg, 75%) as a yellow oil.
Method 1a (1 mmol scale): Isocyanide **1a** (179 mg, 1 mmol), but-3-en-1-ol (72 mg, 1 mmol), $Fe(acac)_3$ (70 mg, 0.2 mmol), TBHP (70%, 215 μL, 1.50 mmol) and $PhSiH_3$ (108 mg, 1 mmol) in *i*PrOH (0.4 M, 2.5 mL) at 60 °C. Purification by chromatography (hexane → hexane/EtOAc 50:50) gave **6a** (176 mg, 70%) as a yellow oil.
Method 2: Phenanthridine **6** (50 mg, 0.28 mmol), but-3-en-1-ol (60 mg, 0.84 mmol), $Fe(acac)_3$ (98 mg, 0.28 mmol), TFA (43 μL, 0.56 mmol) and $PhSiH_3$ (30 mg, 0.28 mmol) in 4:1 THF/MeOH (0.2 M, 1.4 mL) at 60 °C. Purification by chromatography (hexane → hexane/EtOAc 50:50) gave **6a** (15 mg, 21%) as a yellow oil.
^{1}H NMR (400 MHz, $CDCl_3$) δ 8.66 (d, *J* = 8.4 Hz, 1H, H-1), 8.54 (d, *J* = 8 Hz, 1H, H-10), 8.36 (d, *J* = 8.4 Hz, 1H, H-7), 8.10 (d, *J* = 8.4 Hz, 1H, H-4), 7.85 (t, *J* = 8.4 Hz, 1H, H-2), 7.71 (t, *J* = 7.6 Hz, 2H, H-3 and H-8), 7.63 (t, *J* = 7.2 Hz, 1H, H-9), 4.97 (br s, 1H, OH), 4.28–4.20 (m, 1H, H-2'), 3.94–3.88 (m, 1H, H-4'), 3.84–3.79 (m, 1H, H-4'), 2.40–2.32 (m, 1H, H-3'), 2.28–2.20 (m, 1H, H-3'), 1.51 (d, *J* = 6.8 Hz, 3H, Me); ^{13}C NMR (101 MHz, $CDCl_3$) δ 165.4 (C-6), 142.8 (C-4a), 133.3 (C-10a), 130.7 (C-2), 129.2 (C-4), 128.9 (C-3), 127.6 (C-8), 126.8 (C-9), 126.1 (C-7), 124.9 (C-6a), 123.6 (C-10b), 122.8 (C-1), 122.0 (C-10), 59.9 (C-4'), 36.7 (C-3'), 35.3 (C-2'), 20.1 (Me); HRMS (ESI) *m/z*: $[M+H]^+$ calcd for $[C_{17}H_{18}NO]^+$ 252.1388, found 252.1387.

6-(4-Hydroxybutan-2-yl)-8-methylphenanthridine (6b)

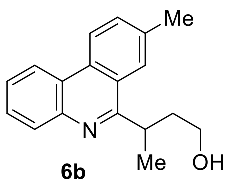

6b

Method 1a: Isocyanide **1b** (100 mg, 0.52 mmol), but-3-en-1-ol (37 mg, 0.52 mmol), $Fe(acac)_3$ (36 mg, 0.10 mmol), TBHP (70%, 111 μL, 0.78 mmol) and $PhSiH_3$ (56 mg, 0.52 mmol) in *i*PrOH (0.4 M, 1.3 mL) at 60 °C. Purification by chromatography (hexane → hexane/EtOAc 50:50) gave **6b** (91 mg, 66%) as a yellow oil.
^{1}H NMR (400 MHz, $CDCl_3$) δ 8.56 (d, *J* = 8.4 Hz, 1H, H-1), 8.51 (dd, *J* = 8, 1.6 Hz, 1H, H-2), 8.13 (br s, 1H, H-7), 8.08 (dd, *J* = 8, 1.6 Hz, 1H, H-9), 7.68 (ddd, *J* = 8.4, 7.2, 1.6 Hz, 2H, H-3 and H-4), 7.61 (ddd, *J* = 8.4, 7.2, 1.6 Hz, 1H, H-10), 4.28–4.20 (m, 1H, H-2'), 3.96–3.90 (m, 1H, H-4'), 3.84–3.79 (m, 1H, H-4'), 2.63 (s, 3H, Me), 2.39–2.31 (m, 1H, H-3'), 2.29–2.21 (m, 1H, H-3'), 1.51 (d, *J* = 7.2 Hz, 3H, Me); ^{13}C NMR (101 MHz, $CDCl_3$) δ 165.1 (C-6), 142.4 (C-4a), 137.5 (C-8 and C-10a), 132.5 (C-3), 129.1 (C-9), 128.4 (C-4), 126.8 (C-10), 125.6 (C-7), 125.1 (C-6a), 123.8 (C-10b), 122.8 (C-1), 121.9 (C-2), 59.9 (C-4'), 36.5 (C-3'), 35.4 (C-2'), 21.1 (Me), 20.1 (Me). HRMS (ESI) *m/z*: $[M+H]^+$ calcd for $[C_{18}H_{20}NO]^+$ 266.1545, found 266.1547.

6-(4-Hydroxybutan-2-yl)-8-fluorophenanthridine (6c)

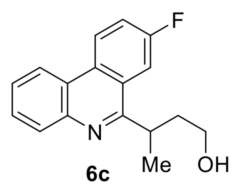

6c

Method 1a: Isocyanide **1c** (100 mg, 0.51 mmol), but-3-en-1-ol (36 mg, 0.51 mmol), Fe(acac)$_3$ (36 mg, 0.10 mmol), TBHP (70%, 110 μL, 0.76 mmol) and $PhSiH_3$ (55 mg, 0.51 mmol) in *i*PrOH (0.4 M, 1.3 mL) at 60 °C. Purification by chromatography (hexane → hexane/EtOAc 50:50) gave **6c** (105 mg, 77%) as a white solid. ^{1}H NMR (400 MHz, $CDCl_3$) δ 8.66 (dd, *J* = 9.2, 5.6 Hz, 1H, H-10), 8.49 (dd, *J* = 8.4, 1.2 Hz, 1H, H-1), 8.10 (dd, *J* = 8.8, 1.6 Hz, 1H, H-4), 7.98 (dd, *J* = 10.4, 2.8 Hz, 1H, H-7), 7.71 (ddd, *J* = 8.4, 7.2, 1.2 Hz, 1H, H-3), 7.64 (ddd, *J* = 8.4, 7,2, 1.2 Hz, 1H, H-2), 7.60 (ddd, *J* = 10.8, 8, 2.8 Hz, 1H, H-9), 4.13–4.04 (m, 1H, H-2'), 3.92–3.86 (m, 1H, H-4'), 3.84–3.78 (m, 1H, H-4'), 2.41–2.34 (m, 1H, H-3'), 2.25–2.17 (m, 1H, H-3'), 1.50 (d, *J* = 6.8 Hz, 3H, Me); ^{13}C NMR (101 MHz, $CDCl_3$) δ 164.5 (C-6), 161.7 (d, *J* = 248.7 Hz, C-8), 142.6 (C-4a), 130.0 (C-10a), 129.5 (C-4), 128.7 (C-3), 127.2 (C-2), 126.3 (d, *J* = 7.2 Hz, C-6a), 125.4 (d, *J* = 8.4 Hz, C-10), 123.2 (C-10b), 121.8 (C-1), 119.8 (d, *J* = 23.6 Hz, C-9), 110.8 (d, *J* = 21.9 Hz, C-7), 60.1 (C-4'), 36.8 (C-3'), 35.3 (C-2'), 20.0 (Me); ^{19}F NMR (376 MHz, $CDCl_3$)δ −111.87 (s). HRMS (ESI) *m/z*: $[M+H]^+$ calcd for $[C_{17}H_{17}FNO]^+$ 270.1294, found 270.1291.

6-(4-Hydroxybutan-2-yl)-3-methoxyphenanthridine (6d)

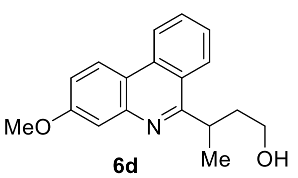

6d

Method 1a: Isocyanide **1d** (100 mg, 0.48 mmol), but-3-en-1-ol (34 mg, 0.48 mmol), Fe(acac)$_3$ (34 mg, 0.10 mmol), TBHP (70%, 103 μL, 0.72 mmol) and $PhSiH_3$ (52 mg, 0.48 mmol) in *i*PrOH (0.4 M, 1.2 mL) at 60 °C. Purification by chromatography (hexane → hexane/EtOAc 50:50) gave **6d** (98 mg, 73%) as an orange solid.

^{1}H NMR (400 MHz, $CDCl_3$) δ 8.55 (d, *J* = 8.4 Hz, 1H, H-10), 8.42 (d, *J* = 9.2 Hz, 1H, H-1), 8.32 (d, *J* = 8.4 Hz, 1H, H-7), 7.81 (ddd, *J* = 8.4, 6.8, 1.2 Hz, 1H, H-9), 7.63 (ddd, *J* = 8, 6.8, 1.2 Hz, 1H, H-8), 7.47 (d, *J* = 2.8 Hz, 1H, H-4), 7.26 (dd, *J* = 9.2, 2.8 Hz, 1H, H-2), 4.29–4.21 (m, 1H, H-2'), 3.98 (s, 3H, OMe), 3.95–3.89 (m, 1H, H-4'), 3.84–3.79 (m, 1H, H-4'), 2.38–2.31 (m, 1H, H-3'), 2.28–2.20 (m, 1H, H-3'), 1.51 (d, *J* = 6.8 Hz, 3H, Me); ^{13}C NMR (101 MHz, $CDCl_3$) δ 165.9 (C-6), 160.4 (C-3), 144.5 (C-4a), 133.6 (C-10a), 130.8 (C-9), 126.5 (C-8), 126.1 (C-7), 124.0 (C-6a), 123.3 (C-1), 122.4 (C-10), 118.0 (C-2), 117.7 (C-10b), 108.8 (C-4), 59.9 (C-4'), 55.8 (OMe), 36.6 (C-3'), 35.4 (C-2'), 20.1 (Me); HRMS (ESI) *m/z*: $[M+H]^+$ calcd for $[C_{18}H_{20}NO_2]^+$ 282.1494, found 282.1496.

6-(4-Hydroxybutan-2-yl)-2-(trifluoromethyl)phenanthridine (6e)

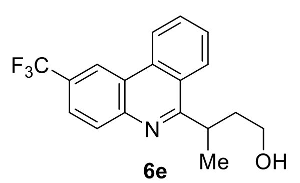

6e

Method 1a: Isocyanide **1e** (100 mg, 0.40 mmol), but-3-en-1-ol (29 mg, 0.40 mmol), Fe(acac)$_3$ (29 mg, 0.08 mmol), TBHP (70%, 87 μL, 0.61 mmol) and $PhSiH_3$ (44 mg, 0.40 mmol) in *i*PrOH (0.4 M, 1.0 mL) at 60 °C. Purification by chromatography (hexane → hexane/EtOAc 75:25) gave **6e** (88 mg, 68%) as a pale yellow solid.

^{1}H NMR (400 MHz, $CDCl_3$) δ 8.83 (s, 1H, H-1), 8.69 (d, *J* = 8 Hz, 1H, H-10), 8.42 (d, *J* = 8 Hz, 1H, H-7), 8.20 (d, *J* = 8.8 Hz, 1H, H-4), 7.94–7.89 (m, 2H, H-3 and H-9), 7.79 (ddd, *J* = 8.4 Hz, 7.2, 1.2 Hz, H-8), 4.18 (m, 1H, H-2'), 3.89–3.78 (m, 2H, H-4'), 2.45–2.37 (m, 1H, H-3'), 2.23–2.15 (m, 1H, H-3'), 1.52 (d, *J* = 6.8 Hz, 3H, Me); ^{13}C NMR (101 MHz, $CDCl_3$) δ 167.9 (C-6), 144.6 (C-4a), 132.9 (C-10a), 131.3 (C-9), 130.3 (C-4), 128.5 (C-

8), 127.2 (q, J = 272.3 Hz, CF_3), 126.3 (C-7), 125.3 (C-6a), 124.8 (q, J = 3.3 Hz, C-3), 123.2 (C-10b), 122.9 (C-10), 119.9 (q, J = 4.2 Hz, C-1), 60.3 (C-4'), 37.1 (C-3'), 34.9 (C-2'), 20.3 (Me); ^{19}F NMR (376 MHz, $CDCl_3$)δ −61.75 (s). HRMS (ESI) *m/z*: $[M+H]^+$ calcd for $[C_{18}H_{17}F_3NO]^+$ 320.1262, found 320.1262.

6-(Dodecan-2-yl)phenanthridine (**6f**)

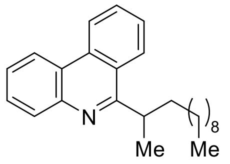

6f

Method 1a: Isocyanide **1a** (50 mg, 0.28 mmol), 1-dodecene (47 mg, 0.28 mmol), $Fe(acac)_3$ (20 mg, 0.056 mmol), TBHP (70%, 60 μL, 0.42 mmol) and $PhSiH_3$ (30 mg, 0.28 mmol) in *i*PrOH (0.4 M, 0.7 mL) at 60 °C. Purification by chromatography (hexane → hexane/EtOAc 99:1) gave **6f** (53 mg, 54%) as a yellow oil.

Method 2: Phenanthridine **6** (50 mg, 0.28 mmol), 1-dodecene (140 mg, 0.84 mmol), $Fe(acac)_3$ (98 mg, 0.28 mmol), TFA (43 μL, 0.56 mmol) and $PhSiH_3$ (30 mg, 0.28 mmol) in 4:1 THF/MeOH (0.2 M, 1.4 mL) at 60 °C. Purification by chromatography (hexane → hexane/EtOAc 99:1) gave **6f** (61 mg, 63%) as a yellow oil.

^{1}H NMR (400 MHz, $CDCl_3$) δ 8.67 (d, J = 8 Hz, 1H, H-1), 8.55 (dd, J = 8, 1.2 Hz, 1H, H-10), 8.33 (d, J = 8.4 Hz, 1H, H-7), 8.15 (d, J = 8 Hz, 1H, H-4), 7.82 (ddd, J = 8, 6.8, 1.2 Hz, 1H, H-2), 7.70 (dddd, J = 9.6, 7.2, 6, 1.6 Hz, 2H, H-3 and H-8), 7.61 (ddd, J = 8.4, 7.2, 1.6 Hz, 1H, H-9), 3.84 (sext, J = 6.8 Hz, 1H, H-2'), 2.18–2.09 (m, 1H, H-3'), 1.81–1.72 (m, 1H, H-3'), 1.48 (d, J = 6.8 Hz, 3H, Me), 1.23 (br s, 16H, H-4'–H-11'), 0.87 (t, J = 6.8 Hz, 3H, Me); ^{13}C NMR (101 MHz, $CDCl_3$) δ 165.8 (C-6), 144.0 (C-4a), 133.2 (C-10a), 130.1 (C-4), 130.0 (C-2), 128.5 (C-3), 127.2 (C-8), 126.2 (C-9), 125.7 (C-7), 125.3 (C-6a), 123.4 (C-10b), 122.7 (C-1), 121.9 (C-10), 36.8 (C-2'), 36.4 (C-3'), 32.1 (CH_2), 30.0 (CH_2), 29.8 (CH_2), 29.8 (CH_2), 29.7 (CH_2), 29.5 (CH_2), 28.1 (CH_2), 22.8 (CH_2), 20.3 (Me), 14.3 (Me); HRMS (ESI) *m/z*: $[M+H]^+$ calcd for $[C_{25}H_{34}N]^+$ 348.5540, found 348.4562.

6-(11-Hydroxyundecan-2-yl)phenanthridine (6g)

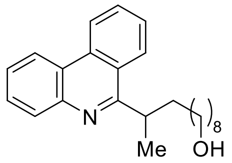

6g

Method 1a: Isocyanide **1a** (100 mg, 0.56 mmol), 10-undecen-1-ol (95 mg, 0.56 mmol), $Fe(acac)_3$ (40 mg, 0.11 mmol), TBHP (70%, 120 μL, 0.84 mmol) and $PhSiH_3$ (60 mg, 0.56 mmol) in *i*PrOH (0.4 M, 1.4 mL) at 60 °C. Purification by chromatography (hexane → hexane/EtOAc 90:10) gave **6g** (111 mg, 59%) as a yellow oil.

Method 2: Phenanthridine **6** (100 mg, 0.56 mmol), 10-undecen-1-ol (285 mg, 1.67 mmol), $Fe(acac)_3$ (197 mg, 0.56 mmol), TFA (85 μL, 1.12 mmol) and $PhSiH_3$ (60 mg, 0.56 mmol) in 4:1 THF/MeOH (0.2 M, 2.9 mL) at 60 °C. Purification by chromatography (hexane → hexane/EtOAc 90:10) gave **6g** (133 mg, 71%) as a yellow oil.

^{1}H NMR (400 MHz, $CDCl_3$) δ 8.65 (d, J = 8.4 Hz, 1H, H-1), 8.54 (d, J = 8.4 Hz, 1H, H-10), 8.32 (d, J = 8.4 Hz, 1H, H-7), 8.15 (d, J = 8 Hz, 1H, H-4), 7.82 (ddd, J = 8.4, 7.2, 1.6 Hz, 1H, H-2), 7.73–7.67 (m, 2H, H-3 and H8), 7.61 (ddd, J = 8.4, 6.8, 1.6 Hz, 1H, H-9), 3.83 (sext, J = 6.8 Hz, 1H, H-2'), 3.61 (t, J = 6.8 Hz, 2H, H-11'), 2.19–2.10 (m, 1H, CH_2), 1.81–1.72 (m, 1H, CH_2), 1.56–1.51 (m, 2H, CH_2), 1.49 (d, J = 6.8 Hz, 3H, Me), 1.36–1.26 (m, 12H, CH_2); ^{13}C NMR (101 MHz, $CDCl_3$) δ 165.8 (C-6), 143.9 (C-4a), 133.1 (C-10a), 130.1 (C-2), 129.9 (C-4), 128.5 (C-3), 127.2 (C-8), 126.3 (C-9), 125.7 (C-7), 125.3 (C-6a), 123.4 (C-10b), 122.7 (C-1), 121.9 (C-10), 63.1 (C-11'), 36.7 (C-2'), 36.4 (CH_2), 32.9

(CH2), 29.9 (CH2), 29.6 (CH2), 29.5 (CH2), 29.4 (CH2), 28.0 (CH2), 25.8 (CH2), 20.3 (Me); HRMS (ESI) *m/z*: [M+H]$^+$ calcd for $[C_{24}H_{30}NO]^+$ 348.2327, found 348.2330.

6-(4-(Benzoyloxy)butan-2-yl)phenanthridine (6h)

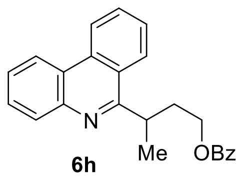

6h

Method 1a: Isocyanide **1a** (100 mg, 0.56 mmol), but-3-en-1-yl benzoate (98 mg, 0.56 mmol), $Fe(acac)_3$ (40 mg, 0.11 mmol), TBHP (70%, 120 μL, 0.84 mmol) and $PhSiH_3$ (60 mg, 0.56 mmol) in *i*PrOH (0.4 M, 1.4 mL) at 60 °C. Purification by chromatography (hexane → hexane/EtOAc 97.5:2.5) gave **6h** (105 mg, 53%) as a pale-yellow oil.

Method 2: Phenanthridine **6** (100 mg, 0.56 mmol), but-3-en-1-yl benzoate (295 mg, 1.67 mmol), $Fe(acac)_3$ (197 mg, 0.56 mmol), TFA (85 μL, 1.12 mmol) and $PhSiH_3$ (60 mg, 0.56 mmol) in 4:1 THF/MeOH (0.2 M, 2.8 mL) at 60 °C. Purification by chromatography (hexane → hexane/EtOAc 97.5:2.5) gave **6h** (151 mg, 76%) as a pale-yellow oil.

^{1}H NMR (400 MHz, $CDCl_3$) δ 8.65 (d, *J* = 8.4 Hz, 1H, H-10), 8.54 (d, *J* = 8 Hz, 1H, H-1), 8.33 (d, *J* = 8.4 Hz, 1H, H-7), 8.15 (dd, *J* = 8, 1.2 Hz, 1H, H-3), 7.95–7.93 (m, 2H, Ph), 7.81 (ddd, *J* = 8.8, 6.8, 1.2 Hz, 1H, H-9), 7.72 (ddd, *J* = 8.4, 7.2, 1.6 Hz, 1H, H-2), 7.65 (ddd, *J* = 8.4, 6.8, 1.2 Hz, 1H, H-8), 7.62 (ddd, *J* = 8.4, 7.2, 1.6 Hz, 1H, H-4), 7.53 (tt, *J* = 6.8, 1.2 Hz, 1H, Ph), 7.38 (t, *J* = 8.4 Hz, 2H, Ph), 4.55–4.49 (m, 1H, H-4'), 4.41–4.35 (m, 1H, H-4'), 4.12 (sext, *J* = 14.4, 6.8 Hz, 1H, H-2'), 2.78 (dq, *J* = 14, 6.8 Hz, 1H, H-3'), 2.28 (dq, *J* = 12, 6.8 Hz, 1H, H-3'), 1.55 (d, *J* = 6.8 Hz, 3H, Me); ^{13}C NMR (101 MHz, $CDCl_3$) δ 166.7 (C=O), 164.1 (C-6), 143.9 (C-4a), 133.2 (C-10a), 132.9 (Ph), 130.5 (C_{ipso}), 130.2 (C-3), 130.1 (C-9), 129.6 (Ph), 128.6 (C-2), 128.4 (Ph), 127.3 (C-8), 126.5 (C-4), 125.4 (C-7), 125.1 (C-6a), 123.5 (C-10b), 122.8 (C-10), 121.9 (C-1), 63.9 (C-4'), 34.7 (C-3'), 33.6 (C-2'), 20.9 (Me); HRMS (ESI) *m/z*: [M+H]$^+$ calcd for $[C_{24}H_{22}NO_2]^+$ 356.1650, found 356.1647.

6-(4-(1,3-Dioxoisoindolin-2-yl)butan-2-yl)phenanthridine (6i)

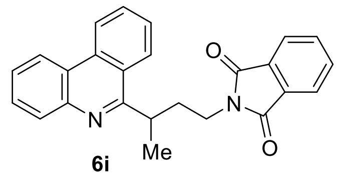

6i

Method 1a: Isocyanide **1a** (100 mg, 0.56 mmol), 2-(but-3-en-1-yl)isoindoline-1,3-dione (112 mg, 0.56 mmol), $Fe(acac)_3$ (40 mg, 0.11 mmol), TBHP (70%, 120 μL, 0.84 mmol) and $PhSiH_3$ (60 mg, 0.56 mmol) in *i*PrOH (0.4 M, 1.4 mL) at 60 °C. Purification by chromatography (hexane → hexane/EtOAc 75:25) gave **6i** (132 mg, 62%) as a pale-yellow solid.

Method 2: Phenanthridine **6** (100 mg, 0.56 mmol), 2-(but-3-en-1-yl)isoindoline-1,3-dione (336 mg, 1.67 mmol), $Fe(acac)_3$ (197 mg, 0.56 mmol), TFA (85 μL, 1.12 mmol) and $PhSiH_3$ (60 mg, 0.56 mmol) in 4:1 THF/MeOH (0.2 M, 2.8 mL) at 60 °C. Purification by chromatography (hexane → hexane/EtOAc 75:25) gave **6i** (161 mg, 76%) as a pale-yellow solid.

^{1}H NMR (400 MHz, $CDCl_3$) δ 8.57 (d, *J* = 9.6 Hz, 1H, H-10), 8.43 (d, *J* = 8.4 Hz, 1H, H-1), 8.24 (d, *J* = 8 Hz, 1H, H-7), 8.08 (d, *J* = 8.4 Hz, 1H, H-4), 7.79 (ddd, *J* = 8.4, 7.2, 1.2 Hz, 1H, H-9), 7.69–7.63 (m, 2H, H-3 and H-8), 7.61–7.53 (m, 5H, H-2 and Ar), 3.98–3.86 (m, 2H, H-2' and H-4'), 3.83–3.76 (m, 1H, H-4'), 2.88 (dq, *J* = 15.6, 7.6 Hz, 1H, H-3'), 2.12 (dq, *J* = 13.2, 5.6 Hz, 1H, H-3'), 1.48 (d, *J* = 7.2 Hz, 3H, Me); ^{13}C NMR (101 MHz, $CDCl_3$) δ 168.4 (C=O), 163.9 (C-6), 143.7 (C-4a), 133.7 (Ar), 133.2 (C-10a), 132.1 (C_{ipso}), 130.1 (C-4 and C-9), 128.5 (C-3), 127.3 (C-8), 126.3 (C-2), 125.6 (C-7),

124.9 (C-6a), 123.4 (C-10b), 122.9 (Ar), 122.6 (C-10), 121.8 (C-1), 37.1 (C-4'), 35.1 (C-2'), 33.6 (C-3'), 21.5 (Me). HRMS (ESI) *m/z*: $[M+H]^+$ calcd for $[C_{25}H_{21}N_2O_2]^+$ 381.1603, found 381.1605.

6-Cyclopentylphenanthridine (6j)

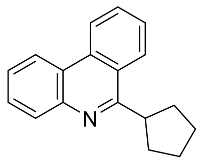

6j

Method 1a: Isocyanide **1a** (100 mg, 0.56 mmol), cyclopentene (38 mg, 0.56 mmol), $Fe(acac)_3$ (40 mg, 0.11 mmol), TBHP (70%, 120 μL, 0.84 mmol) and $PhSiH_3$ (60 mg, 0.56 mmol) in *i*PrOH (0.4 M, 1.4 mL) at 60 °C. Purification by chromatography (hexane → hexane/EtOAc 97.5:2.5) gave **6j** (36 mg, 26%) as a yellowish oil.
Method 2: Phenanthridine **6** (100 mg, 0.56 mmol), cyclopentene (114 mg, 1.67 mmol), $Fe(acac)_3$ (197 mg, 0.56 mmol), TFA (85 μL, 1.12 mmol) and $PhSiH_3$ (60 mg, 0.56 mmol) in 4:1 THF/MeOH (0.2 M, 2.9 mL) at 60 °C. Purification by chromatography (hexane → hexane/EtOAc 97.5:2.5) gave **6j** (97 mg, 70%) as a yellowish oil.
Method 3: Isocyanide **1a** (100 mg, 0.56 mmol), $Fe(acac)_3$ (40 mg, 0.11 mmol), TBHP (70% in water, 80 μL, 0.56 mmol), and $PhSiH_3$ (181 mg, 1.67 mmol) in 4:1 MTBE/MeOH (0.4 M, 1.4 mL) at room temperature. After 15 min, was added MTBE (to a 0.2 M solution, 1.4 mL), $Fe(acac)_3$ (157 mg, 0.45 mmol), TFA (85 μL, 1.12 mmol) and cyclopentene (153 μL, 1.67 mmol) at 60 °C, open to air. Purification by chromatography (hexane → hexane/EtOAc 97.5:2.5) gave **6j** (82 mg, 62%) as a yellowish oil.
^{1}H NMR (400 MHz, $CDCl_3$) δ 8.62 (d, *J* = 9.6 Hz, 1H, H-1), 8.53 (d, *J* = 8 Hz, 1H, H-10), 8.34 (d, *J* = 8,4 Hz, 1H, H-7), 8.18 (d, *J* = 8 Hz, 1H, H-4), 7.80 (ddd, *J* = 8.4, 7.2, 1.2 Hz, 1H, H-2), 7.73 (ddd, *J* = 8.4, 7.2, 1.6 Hz, 1H, H-3), 7.68 (ddd, *J* = 8.4, 7.2, 1.2 Hz, 1H, H-8), 7.61 (ddd, *J* = 8.4, 7.2, 1.6 Hz, H-9), 4.09 (q, *J* = 8 Hz, 1H, H-1'), 2.36–2.18 (m, 4H, CH_2), 2.04–1.94 (m, 2H, CH_2), 1.89–1.80 (m, 2H, CH_2); ^{13}C NMR (101 MHz, $CDCl_3$) δ 164.2 (C-6), 143.8 (C-4a), 132.9 (C-10a), 130.0 (C-2), 129.9 (C-4), 128.4 (C-3), 127.1 (C-8), 126.2 (C-9), 126.1 (C-7), 125.7 (C-6a), 123.5 (C-10b), 122.4 (C-1), 121.9 (C-10), 43.6 (C-1'), 32.2 (CH_2), 26.1 (CH_2); HRMS (ESI) *m/z*: $[M+H]^+$ calcd for $[C_{18}H_{18}N]^+$ 248.1439, found 248.1442. Spectral data were identical to those previously reported.[13]

6-Cyclohexylphenanthridine (6k)

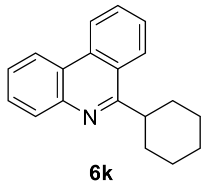

6k

Method 1a: Isocyanide **1a** (50 mg, 0.28 mmol), cyclohexene (23 mg, 028 mmol), $Fe(acac)_3$ (20 mg, 0.056 mmol), TBHP (70%, 60 μL, 0.42 mmol) and $PhSiH_3$ (30 mg, 0.28 mmol) in *i*PrOH (0.4 M, 700 μL) at 60 °C. Purification by chromatography (hexane → hexane/EtOAc 95:5) gave **6k** (10 mg, 14%) as a yellow oil.
Method 2: Phenanthridine **6** (100 mg, 0.56 mmol), cyclohexene (137 mg, 1.67 mmol), $Fe(acac)_3$ (197 mg, 0.56 mmol), TFA (85 μL, 1.12 mmol) and $PhSiH_3$ (60 mg, 0.56 mmol) in 4:1 THF/MeOH (0.2 M, 2.9 mL) at 60 °C. Purification by chromatography (hexane → hexane/EtOAc 95:5) gave **6k** (92 mg, 63%) as a yellow oil.
Method 3: Isocyanide **1a** (100 mg, 0.56 mmol), $Fe(acac)_3$ (40 mg, 0.11 mmol), TBHP (70% in water, 80 μL, 0.56 mmol), and $PhSiH_3$ (181 mg, 1.67 mmol) in 4:1 MTBE/MeOH (0.4 M, 1.4 mL) at room temperature. After 15 min, was added MTBE (to a 0.2 M solution, 1.4 mL), $Fe(acac)_3$ (157 mg, 0.45 mmol), TFA (85 μL, 1.12 mmol), and 1-methyl-1-cyclohexene (170 μL, 1.67 mmol) at 60 °C, open to air. Purification by chromatography (hexane → hexane/EtOAc 95:5) gave **6k** (84 mg, 58%) as a yellow oil.

^{1}H NMR (400 MHz, $CDCl_3$) δ 8.65 (d, *J* = 9.6 Hz, 1H, H-1), 8.54 (dd, *J* = 8, 1.2 Hz, 1H, H-10), 8.32 (d, *J* = 8.4 Hz, 1H, H-7), 8.13 (dd, *J* = 8, 1.6 Hz, 1H, H-4), 7.81 (ddd, *J* = 8.4, 6.8, 1.2 Hz, 1H, H-2), 7.72–7.67 (m, 2H, H-3 and H-8), 7.60 (ddd, *J* = 8.4, 7.2, 1.6 Hz, 1H, H-9), 3.62 (tt, *J* = 11.2, 3.2 Hz, 1H, H-1'), 2.09–2.07 (m, 2H, CH_2), 1.99–1.93 (m, 4H, CH_2), 1.88–1.82 (m, 1H, CH_2), 1.63–1.55 (m, 2H, CH_2), 1.45 (tt, *J* = 12.8, 3.6 Hz, 1H, CH_2); ^{13}C NMR (101 MHz, $CDCl_3$) δ 165.4 (C-6), 144.0 (C-4a), 133.2 (C-10a), 130.1 (C-4), 130.0 (C-2), 128.5 (C-3), 127.2 (C-8), 126.3 (C-9), 125.7 (C-7), 124.9 (C-6a), 123.5 (C-10b), 122.7 (C-1), 121.9 (C-10), 42.1 (C-1'), 32.4 (CH_2), 27.0 (CH_2), 26.5 (CH_2); HRMS (ESI) *m/z*: $[M+H]^+$ calcd for $[C_{19}H_{20}N]^+$ 262.1595, found 262.1596. Spectral data were identical to those previously reported.[11]

6-(1-Methylcyclohexyl)-5,6-dihydrophenanthridine (6l)

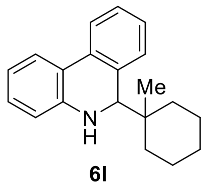

6l

Method 1a: Isocyanide **1a** (100 mg, 0.56 mmol), 1-methyl-1-cyclohexene (54 mg, 0.56 mmol), $Fe(acac)_3$ (40 mg, 0.11 mmol), TBHP (70%, 120 μL, 0.84 mmol) and $PhSiH_3$ (60 mg, 0.56 mmol) in *i*PrOH (0.4 M, 1.4 mL) at 60 °C. Purification by chromatography (hexane → hexane/EtOAc 97:2.5) gave **6l** (8 mg, 5%) as a colorless oil.

Method 2: Phenanthridine **6** (100 mg, 0.56 mmol), 1-methyl-1-cyclohexene (161 mg, 1.67 mmol), $Fe(acac)_3$ (197 mg, 0.56 mmol), TFA (85 μL, 1.12 mmol) and $PhSiH_3$ (60 mg, 0.56 mmol) in 4:1 THF/MeOH (0.2 M, 2.9 mL) at 60 °C. Purification by chromatography (hexane → hexane/EtOAc 97.5:2.5) gave **6l** (145 mg, 94%) as a colorless oil.

Method 3: Isocyanide **1a** (100 mg, 0.56 mmol), $Fe(acac)_3$ (40 mg, 0.11 mmol), TBHP (70% in water, 80 μL, 0.56 mmol) and $PhSiH_3$ (181 mg, 1.67 mmol) in 4:1 MTBE/MeOH (0.4 M, 1.4 mL) at room temperature. After 15 min, was added MTBE (to a 0.2 M solution, 1.4 mL), $Fe(acac)_3$ (157 mg, 0.45 mmol), TFA (85 μL, 1.12 mmol), and 1-methyl-1-cyclohexene (199 μL, 1.67 mmol) at 60 °C, open to air. Purification by chromatography (hexane → hexane/EtOAc 97.5:2.5) gave **6l** (86 mg, 56%) as a colorless oil.

^{1}H NMR (400 MHz, CD_3OD) δ 7.72 (d, *J* = 8 Hz, 1H, H-10), 7.60 (d, *J* = 7.6 Hz, 1H, H-1), 7.29 (tt, *J* = 7.2, 1.6 Hz, 1H, H-9), 7.17 (tt, *J* = 7.6, 2 Hz, 1H, H-8), 7.08 (d, *J* = 7.6 Hz, 1H, H-7), 7.00 (ddd, *J* = 8.4, 6.8, 1.2 Hz, 1H, H-3), 6.67 (d, *J* = 7.6 Hz, 1H, H-4), 6.62 (ddd, *J* = 7.2, 1.2 Hz, 1H, H-2), 4.03 (d, *J* = 2.4 Hz, 1H, H-6), 1.53–1.10 (m, 10H, CH_2) 0.67 (s, 3H, Me); ^{13}C NMR (101 MHz, CD_3OD) δ 146.9 (C-4a), 134.5 (C-10a), 132.9 (C-6a), 130.4 (C-7), 129.9 (C-3), 128.3 (C-9), 126.7 (C-8), 123.6 (C-1), 122.9 (C-10), 122.7 (C-10b), 118.0 (C-4), 114.8 (C-2), 65.2 (C-6), 43.0 (CH_2), 35.0 (CH_2), 34.9 (CH_2), 27.4 (CH_2), 22.9 (CH_2), 22.7 (Me), 19.4 (C-2'); HRMS (ESI) *m/z*: $[M+H]^+$ calcd for $[C_{20}H_{24}N]^+$ 278.1908, found 278.1903.

6-(4-Hydroxy-2-methylbutan-2-yl)-5,6-dihydrophenanthridine (6m)

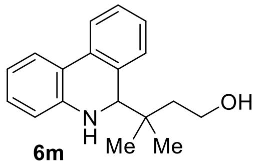

6m

Method 2: Phenanthridine **6** (200 mg, 1.12 mmol), 3-methylbut-3-en-1-ol (288 mg, 3.35 mmol), $Fe(acac)_3$ (394 mg, 1.12 mmol), TFA (171 μL, 2.232 mmol) and $PhSiH_3$ (121 mg, 1.12 mmol) in 4:1 THF/MeOH (0.2 M, 5.6 mL) at 60 °C. Purification by chromatography (hexane → hexane/EtOAc 90:10) gave **6m** (257 mg, 86%) as a white solid.

Method 3: Isocyanide **1a** (200 mg, 1.116 mmol), $Fe(acac)_3$ (79 mg, 0.223 mmol), TBHP (70% in water, 160 μL, 1.116 mmol), and $PhSiH_3$ (362 mg, 3.35 mmol) in 4:1 MTBE/MeOH (0.4 M, 2.8 mL) at room temperature. After 15 min, was added MTBE (to a 0.2 M solution, 2.8 mL), $Fe(acac)_3$ (315 mg, 0.89 mmol), TFA (171 μL, 2.23 mmol), and 3-methylbut-3-en-1-ol (338 μL, 3.35 mmol) at 60 °C, open to air. Purification by chromatography (hexane → hexane/EtOAc 90:10) gave **6m** (155 mg, 52%) as a white solid.

^{1}H NMR (400 MHz, CD_3CN) δ 7.70 (dd, *J* = 8, 1.6 Hz, 1H, H-10), 7.65 (dd, *J* = 7.6, 1.6 Hz, 1H, H-1), 7.35 (d, *J* = 7.6 Hz, 1H, H-7), 7.29 (t, *J* = 7.2 Hz, 1H, H-9), 7.21 (ddd, *J* = 8.8 7.6, 1.2 Hz, 1H, H-8), 7.12 (ddd, *J* = 8.8, 7.2, 1.6 Hz, 1H, H-3), 6.65 (ddd, *J* = 8.8, 7.6, 1.2 Hz, 1H, H-2), 6.51 (d, *J* = 8.4 Hz, 1H, H-4), 4.44 (s, 1H, H-6), 3.64 (t, *J* = 6.8 Hz, 2H, H-4'), 1.80 (t, *J* = 3.6 Hz, 2H, H-3'), 1.40 (s, 3H, Me), 0.93 (s, 3H, Me); ^{13}C NMR (101 MHz, CD_3CN) δ 146.9 (C-4a), 134.2 (C-10a), 133.3 (C-6a), 130.4 (C-3), 128.4 (C-9), 127.8 (C-8), 126.5 (C-7), 123.7 (C-1), 123.1 (C-10), 120.9 (C-10b), 117.4 (C-2), 112.5 (C-4), 68.4 (C-6), 68.3 (C-4'), 42.6 (C-2'), 39.9 (C-3'), 26.9 (Me), 21.7 (Me); HRMS (ESI) *m/z*: $[M+H]^+$ calcd for $[C_{18}H_{22}NO]^+$ 268.1701, found 268.1698.

1-(4-Hydroxybutan-2-yl)-3-methoxycarbonyl-4-phenylisoquinoline (7a)

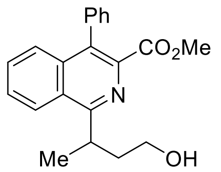

7a

Method 1a: Isocyanide **2** (100 mg, 0.38 mmol), but-3-en-1-ol (27 mg, 0.38 mmol), $Fe(acac)_3$ (27 mg, 0.076 mmol), TBHP (70%, 103 μL, 0.57 mmol) and $PhSiH_3$ (60 mg, 0.56 mmol) in *i*PrOH (0.4 M, 1 mL) at room temperature. Purification by chromatography (hexane → hexane/EtOAc 75:25) gave **7a** (78 mg, 61%) as a yellow oil.

Method 2: Isoquinoline **7** (100 mg, 0.38 mmol), but-3-en-1-ol (82 mg, 1.14 mmol), $Fe(acac)_3$ (134 mg, 0.38 mmol), TFA (58 μL, 0.76 mmol) and $PhSiH_3$ (123 mg, 1.14 mmol) in 4:1 THF/MeOH (0.2 M, 1.9 mL) at room temperature. Purification by chromatography (hexane → hexane/EtOAc 75:25) gave **7a** (41 mg, 32%) as a yellow oil.

^{1}H NMR (400 MHz, $CDCl_3$) δ 8.35 (d, *J* = 8.4 Hz, 1H, H-8), 7.71–7.67 (m, 1H, H-7), 7.64 (br s, 1H, H-6), 7.63 (br s, 1H, H-5), 7.52–7.46 (m, 3H, Ph), 7.35–7.29 (m, 2H, Ph), 4.25–4.19 (m, 1H, H-2'), 3.92–3.86 (m, 1H, H-4'), 3.80–3.74 (m, 1H, H-4'), 3.71 (s, 3H, Me), 2.38–2.30 (m, 1H, H-3'), 2.25–2.17 (m, 1H, H-3'), 1.50 (d, *J* = 6.8 Hz, 3H, Me); ^{13}C NMR (101 MHz, $CDCl_3$) δ 164.8 (C=O), 158.8 (C-1), 136.3 (C-3), 136.2 (Ph), 130.5 (C-6), 129.7 (Ph), 129.6 (Ph), 129.0 (C-4), 128.9 (C-8a), 128.6 (C-7), 128.3 (Ph), 128.2 (Ph), 128.1 (Ph), 127.9 (C-4a), 127.6 (C-5), 124.9 (C-8), 59.9 (C-4'), 52.3 (Me), 36.7 (C-3'), 34.9 (C-2'), 20.4 (Me); HRMS (ESI) *m/z*: $[M+H]^+$ calcd for $[C_{21}H_{22}NO_3]^+$ 336.1599, found 336.1597.

Methyl 1-(4-(Benzoyloxy)butan-2-yl)-4-phenylisoquinoline-3-carboxylate (7b)

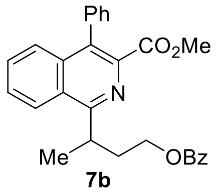

7b

Method 1a: Isocyanide **2** (50 mg, 0.19 mmol), but-3-en-1-yl benzoate (34 mg, 0.19 mmol), $Fe(acac)_3$ (14 mg, 0.038 mmol), TBHP (70%, 41 μL, 0.29 mmol) and $PhSiH_3$ (41 mg, 0.19 mmol) in *i*PrOH (0.4 M, 0.5 mL) at room temperature. Purification by chromatography (hexane → hexane/EtOAc 95:5) gave **7b** (40 mg, 48%) as a white solid.

Method 2: Isoquinoline **7** (100 mg, 0.38 mmol), but-3-en-1-yl benzoate (200 mg, 1.14 mmol), $Fe(acac)_3$ (134 mg, 0.38 mmol), TFA (58 μL, 0.76 mmol) and $PhSiH_3$ (41 mg,

0.38 mmol) in 4:1 THF/MeOH (0.2 M, 1.9 mL) at room temperature. Purification by chromatography (hexane → hexane/EtOAc 75:25) gave **7b** (112 mg, 67%) as a white solid.

^{1}H NMR (400 MHz, $CDCl_3$) δ 8.33–8.29 (m, 1H, H-8), 8.13–8.11 (m, 2H, Ph), 7.99–7.96 (m, 2H, Ph), 7.66–7.59 (m, 4H, H-5, H-6, H-7 and Ph), 7.55 (ddd, *J* = 8.8, 6.8, 1.6 Hz, 1H, Ph), 7.50–7.45 (m, 2H, Ph), 7.42 (t, *J* = 8 Hz, 1H, Ph), 7.35–7.28 (m, 1H, Ph), 4.52–4.46 (m, 1H, H-4'), 4.36–4.30 (m, 1H, H-4'), 4.11 (sext., *J* = 13.2, 6.8, 1H, H-2'), 3.65 (s, 3H, Me), 2.70 (dq, *J* = 14.4, 7.6 Hz, 1H, H-3'), 2.28 (dq, *J* = 13.2, 6 Hz, 1H, H-3'), 1.54 (d, *J* = 6.8 Hz, 3H, Me); ^{13}C NMR (101 MHz, $CDCl_3$) δ 170.9 (C=O), 168.4 (C=O), 164.0 (C-1), 141.6 (C-3), 136.4 (C-4), 136.1 (C_{ipso}), 133.9 (Ph), 132.9 (Ph), 131.6 (C-4a), 130.5 (C_{ipso}), 130.4 (Ph), 130.3 (Ph), 130.1 (C-6), 130.0 (Ph), 129.6 (Ph), 129.3 (C-8a), 128.6 (Ph), 128.5 (Ph), 128.3 (Ph), 128.2 (Ph), 127.9 (C-7), 127.4 (C-5), 127.1 (Ph), 124,5 (C-8), 63.9 (C-4'), 52.3 (Me), 34.9 (C-3'), 33.5 (C-2'), 21.1 (Me); HRMS (ESI) *m/z*: $[M+H]^+$ calcd for $[C_{28}H_{26}NO_4]^+$ 440.1862, found 440.1866.

Methyl 4-Phenyl-1-(4-phenylbutan-2-yl)isoquinoline-3-carboxylate (7c)

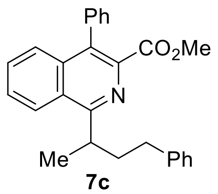

7c

Method 1a: Isocyanide **2** (70 mg, 0.27 mmol), 4-phenyl-1-butene (35 mg, 0.27 mmol), $Fe(acac)_3$ (19 mg, 0.053 mmol), TBHP (70%, 57 μL, 0.40 mmol) and $PhSiH_3$ (29 mg, 0.27 mmol) in *i*PrOH (0.4 M, 0.7 mL) at room temperature. Purification by chromatography (hexane → hexane/EtOAc 95:5) gave **7c** (42 mg, 40%) as a pale-yellow solid.

Method 2: Isoquinoline **7** (70 mg, 0.27 mmol), 4-phenyl-1-butene (105 mg, 0.798 mmol), $Fe(acac)_3$ (94 mg, 0.27 mmol), TFA (41 μL, 0.53 mmol) and $PhSiH_3$ (29 mg, 0.27 mmol) in 4:1 THF/MeOH (0.2 M, 1.4 mL) at room temperature. Purification by chromatography (hexane → hexane/EtOAc 95:5) gave **7a** (66 mg, 63%) as a pale-yellow solid.

^{1}H NMR (400 MHz, $CDCl_3$) δ 8.16–8.11 (m, 1H, H-8), 7.67–7.63 (m, 1H, H-5), 7.63–7.59 (m, 2H, H-6 and H-7), 7.52–7.45 (m, 3H, Ph), 7.38–7.35 (m, 2H, Ph), 7.29–7.25 (m, 2H, Ph), 7.20–7.16 (m, 3H, Ph), 3.84 (sext., *J* = 13.6, 6.8 Hz, 1H, H-2'), 3.68 (s, 3H, Me), 2.70 (t, *J* = 8 Hz, 2H, H-4'), 2.55–2.46 (m, 1H, H-3'), 2.16–2.05 (m, 1H, H-3'), 1.51 (d, *J* = 6.8 Hz, 3H, Me); ^{13}C NMR (101 MHz, $CDCl_3$) δ 168.5 (C=O), 164.9 (C-1), 142.7 (C-3), 141.6 (C_{ipso}), 136.5 (C-4), 135.9 (C_{ipso}), 131.3 (C-4a), 130.1 (C-6), 130.0 (Ph), 128.4 (Ph), 128.3 (Ph), 128.0 (Ph), 127.9 (C-7), 127.3 (C-5), 127.1 (C-8a), 125.8 (Ph), 124.7 (C-8), 52.3 (Me), 37.9 (C-3'), 35.9 (C-2'), 34.2 (C-4'), 20.7 (Me); HRMS (ESI) *m/z*: $[M+H]^+$ calcd for $[C_{27}H_{26}NO_2]^+$ 396.1963, found 396.1963.

1-Cyclohexyl-3-methoxycarbonyl-4-phenylisoquinoline (7d)

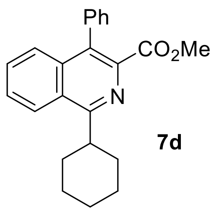

7d

Method 1a: Isocyanide **2** (30 mg, 0.11 mmol), cyclohexene (9 mg, 0.11 mmol), $Fe(acac)_3$ (8 mg, 0.023 mmol), TBHP (70%, 24 μL, 0.17 mmol) and $PhSiH_3$ (37 mg, 0.34 mmol) in *i*PrOH (0.4 M, 285 μL) at room temperature. Purification by chromatography (hexane → hexane/EtOAc 97.5:2.5) gave **7d** (17 mg, 43%) as a yellow oil.

Method 2: Isoquinoline **7** (100 mg, 0.38 mmol), cyclohexene (94 mg, 1.14 mmol), $Fe(acac)_3$ (134 mg, 0.38 mmol), TFA (58 μL, 0.76 mmol) and $PhSiH_3$ (123 mg, 1.14 mmol) in 4:1 THF/MeOH (0.2 M, 1.9 mL) at room temperature. Purification by chromatography (hexane → hexane/EtOAc 97.5:2.5) gave **7d** (75 mg, 57%) as a yellow oil.

Method 3: Isocyanide **2** (86 mg, 0.33 mmol), $Fe(acac)_3$ (23 mg, 0.065 mmol), TBHP (70% in water, 47 μL, 0.33 mmol), and $PhSiH_3$ (106 mg, 0.98 mmol) in 4:1 MTBE/MeOH (0.4 M, 820 μL) at room temperature. After 15 min, was added MTBE (to a 0.2 M solution, 820 μL), $Fe(acac)_3$ (92 mg, 0.26 mmol), TFA (47 μL, 0.65 mmol), and cyclohexene (99 μL, 0.98 mmol) at 60 °C, open to air. Purification by chromatography (hexane → hexane/EtOAc 97.5:2.5) gave **7d** (63 mg, 56%) as a yellow oil. ^{1}H NMR (500 MHz, CD_3OD) δ 8.40 (d, J = 8.5 Hz, 1H, H-8), 7.73–7.70 (m, 1H, H-7), 7.68–7.65 (m, 1H, H-6), 7.62–7.60 (m, 1H, H-5), 7.51–7.45 (m, 3H, Ph), 7.31–7.29 (m, 2H, Ph), 3.74–3.67 (m, 1H, H-1'), 3.59 (s, 3H, CH_3), 1.99–1.83 (m, 6H, CH_2), 1.65–1.58 (m, 2H, CH_2), 1.45–1.38 (m, 1H, CH_2), 1.32–1.25 (m, 1H, CH_2); ^{13}C NMR (126 MHz, CD_3OD) δ 170.2 (C=O), 166.6 (C-1), 143.0 (C-3), 137.4 (C_{ipso}), 136.9 (C-4), 131.9 (C-4a), 131.6 (C-6), 131.1 (Ph), 129.4 (Ph), 129.3 (Ph), 129.1 (C-5), 127.8 (C-7), 127.7 (C-8a), 125.9 (C-8), 52.6 (CH_3), 42.7 (C-1'), 33.4 (CH_2), 27.7 (CH_2), 27.3 (CH_2); HRMS (ESI) *m/z*: $[M+H]^+$ calcd for $[C_{23}H_{24}NO_2]^+$ 346.1807, found 346.1810. Spectral data were identical to those previously reported.[14]

1-(1-Methylcyclohexyl)-3-methoxycarbonyl-4-phenylisoquinoline (7e)

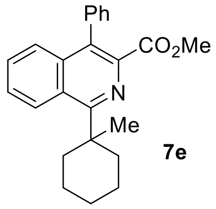

Method 1a: Isocyanide **2** (100 mg, 0.38 mmol), 1-methyl-1-cyclohexene (36 mg, 0.38 mmol), $Fe(acac)_3$ (27 mg, 0.076 mmol), TBHP (70%, 82 μL, 0.57 mmol) and $PhSiH_3$ (123 mg, 1.14 mmol) in *i*PrOH (0.4 M, 0.95 mL) at room temperature. Purification by chromatography (hexane → hexane/EtOAc 99:1) gave **7e** (30 mg, 22%) as a yellow oil.

Method 2: Isoquinoline **7** (100 mg, 0.38 mmol), 1-methyl-1-cyclohexene (109 mg, 1.14 mmol), $Fe(acac)_3$ (134 mg, 0.38 mmol), TFA (58 μL, 0.76 mmol) and $PhSiH_3$ (123 mg, 1.14 mmol) in 4:1 THF/MeOH (0.2 M, 1.9 mL) at room temperature. Purification by chromatography (hexane → hexane/EtOAc 99:1) gave **7e** (93 mg, 68%) as a yellow oil.

Method 3: Isocyanide **2** (86 mg, 0.33 mmol), $Fe(acac)_3$ (23 mg, 0.065 mmol), TBHP (70% in water, 47 μL, 0.33 mmol), and $PhSiH_3$ (106 mg, 0.98 mmol) in 4:1 MTBE/MeOH (0.4 M, 820 μL) at room temperature. After 15 min, was added MTBE (to a 0.2 M solution, 820 μL), $Fe(acac)_3$ (92 mg, 0.26 mmol), TFA (47 μL, 0.65 mmol), and 1-methyl-1-cyclohexene (116 μL, 0.98 mmol) at 60 °C, open to air. Purification by chromatography (hexane → hexane/EtOAc 99:1) gave **7e** (60 mg, 51%) as a yellow oil.

^{1}H NMR (400 MHz, CD_2Cl_2) δ 8.68–8.65 (m, 1H, H-8), 7.68–7.65 (m, 1H, H-7), 7.63–7.55 (m, 2H, H-5 and H-6), 7.52–7.46 (m, 3H, Ph), 7.35–7.33 (m, 2H, Ph), 3.63 (s, 3H, Me), 2.54–2.47 (m, 2H, CH_2), 1.96–1.89 (m, 2H, CH_2), 1.69 (s, 3H, Me), 1.67–1.62 (m, 4H, CH_2), 1.58–1.43 (m, 2H, CH_2); ^{13}C NMR (101 MHz, CD_2Cl_2) δ 168.6 (C=O), 166.8 (C-1), 140.6 (C-3), 137.4 (C-4), 136.9 (C_{ipso}), 131.6 (C-4a), 130.4 (Ph), 129.6 (C-6), 128.5 (Ph), 128.1 (C-7), 127.4 (C-8), 127.1 (C-8a), 126.9 (C-5), 52.2 (Me), 43.8 (CH_2), 39.3 (CH_2), 27.3 (C-1'), 26.9 (Me), 23.3 (CH_2). HRMS (ESI) *m/z*: $[M+H]^+$ calcd for $[C_{24}H_{26}NO_2]^+$ 360.1963, found 360.1959.

2-(4-Hydroxybutan-2-yl)-3-(2-oxo-2-phenylethyl)indole (8a)

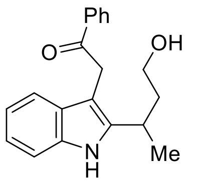

8a

Method 1b: Isocyanide **3** (100 mg, 0.43 mmol), but-3-en-1-ol (31 mg, 0.43 mmol), and $Fe(acac)_3$ (30 mg, 0.086 mmol), and $PhSiH_3$ (46 mg, 0.43 mmol) in *i*PrOH (0.04 M, 10.7 mL) at room temperature. Purification by chromatography (hexane → hexane/EtOAc 75:25) gave **8a** (62 mg, 47%) as a brownish oil.

^{1}H NMR (400 MHz, $CDCl_3$) δ 8.10 (d, *J* = 8.4 Hz, 2H, Ph), 8.03 (s, 1H, NH), 7.59 (t, *J* = 7.6 Hz, 1H, Ph), 7.49 (d, *J* = 8 Hz, 2H, Ph), 7.38 (d, *J* = 7.6 Hz, 1H, H-4), 7.30 (d, *J* = 8 Hz, 1H, H-7), 7.12 (t, *J* = 7.2 Hz, 1H, H-6), 7.05 (t, *J* = 7.6 Hz, 1H, H-5), 4.59 and 4.22 (2d, *J* = 16.8 Hz, 1H each, CH_2), 3.64–3.58 (m, 1H) and 3.56–3.49 (m, 1H, H-4'), 3.38–3.29 (m, 1H, H-2'), 2.56 (br s, 1H, OH), 2.01–1.93 (m, 1H) and 1.87–1.79 (m, 1H, H-3'), 1.32 (d, *J* = 6.8 Hz, 3H, Me); ^{13}C NMR (101 MHz, $CDCl_3$) δ 199.1 (C=O), 140.7 (C-2), 136.9 (C-7a), 135.4 (C_{ipso}), 133.2 (Ph), 128.6 (Ph), 128.5 (Ph), 128.3 (C-3a), 121.4 (C-6), 119.5 (C-5), 117.9 (C-4), 110.6 (C-7), 104.3 (C-3), 60.4 (C-4'), 39.5 (C-3'), 34.1 (CH_2), 27.9 (C-2'), 21.4 (Me). HRMS (ESI) *m/z*: $[M+H]^+$ calcd for $[C_{20}H_{22}NO_2]^+$ 308.1650, found 308.1652.

2-(4-(Benzoyloxy)butan-2-yl)-3-(2-oxo-2-phenylethyl)-1H-indole (8b)

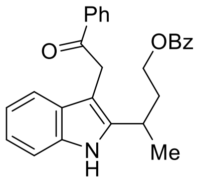

8b

Method 1b: Isocyanide **3** (100 mg, 0.43 mmol), but-3-en-1-yl benzoate (76 mg, 0.43 mmol), and $Fe(acac)_3$ (30 mg, 0.086 mmol) and $PhSiH_3$ (46 mg, 0.43 mmol) in *i*PrOH (0.04 M, 10.7 mL) at room temperature. Purification by chromatography (hexane → hexane/EtOAc 95:5) gave **8b** (72 mg, 41%) as a yellow solid.

^{1}H NMR (400 MHz, $CDCl_3$) δ 7.99–7.97 (m, 2H, Ph), 7.91–7.88 (m, 2H, Ph), 7.53–7.48 (m, 2H, Ph), 7.44 (d, *J* = 7.6 Hz, 1H, H-4), 7.38 (t, *J* = 7.6 Hz, 2H, Ph), 7.33–7.29 (m, 3H, H-7 and Ph), 7.13 (ddd, *J* = 8, 6.8, 1.2 Hz, 1H, H-6), 7.08 (ddd, *J* = 8, 7.2, 1.2 Hz, 1H, H-5), 4.36 (s, 2H, CH_2), 4.34–4.28 (m, 1H, H-4'), 4.21–4.15 (m, 1H, H-4'), 3.39–3.30 (m, 1H, H-2'), 2.17–2.02 (m, 2H, H-3'), 1.37 (d, *J* = 7.2 Hz, 3H, Me); ^{13}C NMR (101 MHz, $CDCl_3$) δ 197.7 (C=O), 166.7 (C=O), 139.7 (C-2), 136.9 (C-7a), 135.7 (C_{ipso}), 133.0 (Ph), 130.2 (C_{ipso}), 129.6 (Ph), 128.7 (Ph), 128.6 (C-3a), 128.5 (Ph), 121.7 (C-6), 119.8 (C-5), 118.4 (C-4), 110.8 (C-7), 104.8 (C-3), 63.3 (C-4'), 35.9 (C-3'), 34.8 (CH_2), 28.4 (C-2'), 21.0 (Me); HRMS (ESI) *m/z*: $[M+H]^+$ calcd for $[C_{27}H_{26}NO_3]^+$ 412.1912, found 412.1909.

Ethyl 2-(4-Hydroxybutan-2-yl)-3-indoleacetate (9a)

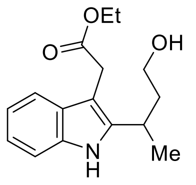

9a

Method 1b: Isocyanide **4** (100 mg, 0.50 mmol), but-3-en-1-ol (36 mg, 0.50 mmol), $Fe(acac)_3$ (35 mg, 0.099 mmol), and $PhSiH_3$ (54 mg, 0.50 mmol) in *i*PrOH (0.04 M, 12.4 mL) at room temperature. Purification by chromatography (hexane → hexane/EtOAc 50:50) gave **9a** (59 mg, 43%) as a yellow oil.

^{1}H NMR (400 MHz, $CDCl_3$) δ 8.15 (s, 1H, NH), 7.56 (d, *J* = 7.2 Hz, 1H, H-4), 7.28 (d, *J* = 8 Hz, 1H, H-7), 7.12 (m, 2H, H-5, H-6), 4.14 (q, *J* = 7.2 Hz, 2H, $OC\underline{H}_2CH_3$), 3.77 (d, *J* = 14.8 Hz, 1H, H-1'), 3.68 (d, *J* = 15.2 Hz, 1H, H-1'), 3.60–3.55 (m, 1H, H-4''), 3.45–3.37 (m, 2H, H-2'', H-4''), 2.03–1.96 (m, 1H, H-3''), 1.82–1.74 (m, 1H, H-3''), 1.33 (d, *J* = 7.2 Hz, 3H, H-1''), 1.26 (t, *J* = 7.2 Hz, 3H, $OCH_2C\underline{H}_3$); ^{13}C NMR (101 MHz, $CDCl_3$) δ 173.3 (C=O), 140.2 (C-2), 135.4 (C-7a), 128.1 (C-3a), 121.5 (C-6), 119.5 (C-5), 118.3 (C-4), 110.5 (C-7), 104.3 (C-3), 61.2 ($O\underline{C}H_2CH_3$),

59.8 (C-4''), 39.6 (C-3''), 30.4 (C-1''), 27.2 (C-2''), 21.5 (C-1''), 14.2 ($OCH_2\underline{C}H_3$). HRMS (ESI) *m/z*: $[M+H]^+$ calcd for $[C_{16}H_{22}NO_3]^+$ 276.1599, found 276.1599.

3-(Ethoxycarbonylmethyl)-2-(4-phenylbutan-2-yl)-1H-indole (9b)

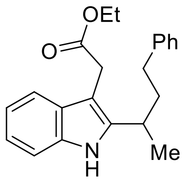

9b

Method 1b: Isocyanide **4** (100 mg, 0.50 mmol), 4-phenyl-1-butene (66 mg, 0.50 mmol), and $Fe(acac)_3$ (35 mg, 0.099 mmol), and $PhSiH_3$ (54 mg, 0.50 mmol) in *i*PrOH (0.04 M, 12.4 mL) at room temperature. Purification by chromatography (hexane → hexane/EtOAc 90:10) gave **9b** (62 mg, 37%) as a yellow oil.

^1H NMR (400 MHz, $CDCl_3$) δ 7.85 (br s, 1H, NH), 7.59 (d, *J* = 8.4 Hz, 1H, H-4), 7.31 (d, *J*= 6.8 Hz, 1H, H-7), 7.28–7.24 (m, 3H, Ph), 7.19–7.15 (m, 1H, Ph), 7.14–7.09 (m, 3H, H-5, H-6 and Ph), 4.09 (q, *J* = 7.2 Hz, 2H, $C\underline{H_2}CH_3$), 3.69 and 3.61 (2d, *J* = 15.2 Hz, 1H each, CH_2), 3.15 (sext., *J* = 14, 6.8 Hz, 1H, H-2'), 2.62–2.50 (m, 2H, H-4'), 2.06–1.95 (m, 2H, H-3'), 1.35 (s, *J* = 7.2 Hz, 3H, Me), 1.20 (t, *J* = 7.2 Hz, 3H, $CH_2C\underline{H_3}$); ^{13}C NMR (101 MHz, $CDCl_3$) δ 172.1 (C=O), 142.0 (C_{ipso}), 140.6 (C-2), 135.4 (C-7a), 128.6 (C-3a), 128.5 (Ph), 128.4 (Ph), 126.0 (Ph), 121.6 (C-6), 119.7 (C-5), 118.7 (C-4), 110.6 (C-7), 104.5 (C-3), 60.8 ($\underline{C}H_2CH_3$), 38.8 (C-3'), 33.9 (C-4'), 30.8 (C-2'), 30.7 (CH_2), 21.3 (Me), 14.4 ($CH_2\underline{C}H_3$); HRMS (ESI) *m/z*: $[M+H]^+$ calcd for $[C_{22}H_{26}NO_2]^+$ 336.1963, found 336.1962.

NMR Assignment Tables

Table S9. Phenanthridines

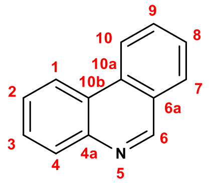

	Theory	6	6a	6b	6c	6d	6e	6f
**H-1**	8.53	8.63	8.66	8.56	8.49	8.42	8.83	8.66
**H-2**	7.64	7.78-7.68	7.85	8.51	7.64	7.26	-	7.82
**H-3**	7.72	7.78-7.68	7.71	7.68	7.71	-	7.94-7.89	7.70
**H-4**	8.19	8.20	8.10	7.68	8.10	7.47	8.20	8.14
**H-6**	9.26	9.30	-	-	-	-	-	-
**H-7**	7.99	8.06	8.36	8.13	7.98	8.32	8.42	8.32
**H-8**	7.65	7.78-7.68	7.71	-	-	7.63	7.79	7.70
**H-9**	7.88	7.88	7.63	8.08	7.60	7.81	7.94-7.89	7.61
**H-10**	8.52	8.59	8.54	7.61	8.66	8.55	8.69	8.55
**C-1**	122.2	122.2	122.7	122.8	121.8	123.3	119.9	122.7
**C-2**	127.0	127.1	130.5	121.9	127.2	118.0	-	130.0
**C-3**	128.6	128.7	128.7	132.5	128.7	160.4	124.8	128.5
**C-4**	130.1	130.0	129.0	128.4	129.5	108.8	130.3	130.1
**C-4a**	144.4	144.3	142.7	142.4	142.6	144.5	144.6	144.0
**C-6**	153.5	153.5	165.2	165.1	164.5	165.9	167.9	165.8
**C-6a**	126.3	126.3	124.8	125.1	126.3	124.0	125.3	125.3
**C-7**	128.7	128.8	125.9	125.6	110.8	126.1	126.3	125.7
**C-8**	127.4	127.5	127.4	137.5	161.7	126.5	128.5	127.2
**C-9**	130.9	131.0	126.6	129.1	119.8	130.8	131.3	126.2
**C-10**	121.8	121.8	121.9	126.8	125.4	122.4	122.9	121.9
**C-10a**	132.4	132.5	133.2	123.8	130.0	133.6	132.9	133.2
**C-10b**	124.0	124.1	123.5	137.5	123.2	117.7	123.3	123.4

	Theory	6g	6h	6i	6j	6k	6l	6m
**H-1**	8.53	8.65	8.54	8.43	8.62	8.65	7.60	7.65
**H-2**	7.64	7.82	7.72	7.61-7.53	7.80	7.81	6.62	6.65
**H-3**	7.72	7.73-7.67	8.15	7.69-7.63	7.73	7.72-7.67	7.00	7.12
**H-4**	8.19	8.15	7.62	8.08	8.18	8.13	6.67	6.51
**H-6**	9.26	-	-	-	-	-	4.03	4.44
**H-7**	7.99	8.32	8.33	8.24	8.34	8.32	7.08	7.35
**H-8**	7.65	7.73-7.67	7.65	7.69-7.63	7.68	7.72-7.67	7.17	7.21
**H-9**	7.88	7.61	7.81	7.79	7.61	7.60	7.29	7.29
**H-10**	8.52	8.54	8.65	8.57	8.53	8.54	7.72	7.70
**C-1**	122.2	122.7	121.9	121.8	122.4	122.7	123.6	123.7
**C-2**	127.0	130.1	128.6	126.3	130.0	130.0	114.8	117.4
**C-3**	128.6	127.2	130.2	128.5	128.4	128.5	129.9	130.4
**C-4**	130.1	129.9	126.5	130.1	129.9	130.1	118.0	112.5
**C-4a**	144.4	143.9	143.9	143.7	143.8	144.0	146.9	146.9
**C-6**	153.5	165.8	164.1	163.9	164.2	165.4	65.2	68.4
**C-6a**	126.3	125.3	125.1	124.9	125.7	124.9	132.9	133.3
**C-7**	128.7	125.7	125.4	125.6	126.1	125.7	130.4	126.5
**C-8**	127.4	127.2	127.3	127.3	127.1	127.2	126.7	127.8
**C-9**	130.9	126.3	130.1	130.1	126.2	126.3	128.3	128.4
**C-10**	121.8	121.9	122.8	122.6	121.9	121.9	122.9	123.1
**C-10a**	132.4	133.1	133.2	133.2	132.9	133.2	134.5	134.2
**C-10b**	124.0	123.4	123.5	123.4	123.5	123.5	122.7	120.9

Table S10. Isoquinolines

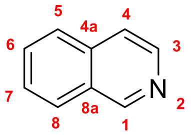

	Theory	7	7a	7b	7c	7d	7e
**H-1**	9.22	9.32	-	-	-	-	-
**H-3**	8.50	-	-	-	-	-	-
**H-4**	7.55	-	-	-	-	-	-
**H-5**	7.72	7.72–7.47	7.63	7.66-7.59	7.67-7.63	7.62-7.60	7.63-7.55
**H-6**	7.59	7.72–7.47	7.64	7.66-7.59	7.63-7.59	7.68-7.65	7.63-7.55
**H-7**	7.51	7.72–7.47	7.71-7.67	7.66-7.59	7.63-7.59	7.73-7.70	7.68-7.65
**H-8**	7.86	8.07	8.35	8.33-8.29	8.16-8.11	8.40	8.68-8.65
**C-1**	152.4	151.7	158.8	164.0	164.9	166.6	166.8
**C-3**	142.9	140.9	136.3	141.6	142.7	143.0	140.6
**C-4**	120.4	135.7	129.0	136.4	136.5	136.9	137.4
**C-4a**	135.7	134.9	127.9	131.6	131.3	131.9	131.6
**C-5**	126.4	126.5	127.6	127.4	127.1	129.1	126.9
**C-6**	130.2	131.1	130.5	130.1	130.1	131.6	129.6
**C-7**	127.1	127.6	128.6	127.9	127.9	127.8	128.1
**C-8**	127.5	128.8	124.9	124.5	124.7	125.9	127.4
**C-8a**	128.6	129.0	128.9	129.3	127.1	127.8	127.1

Table S11. Indoles

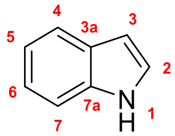

	Theory	8	8a	8b	9	9a	9b
**H-2**	7.05	7.10	-	-	7.10	-	-
**H-3**	6.52	-	-	-	-	-	-
**H-4**	7.64	7.61	7.38	7.44	7.64	7.56	7.59
**H-5**	7.12	7.13	7.05	7.08	7.15	7.12	7.14-7.09
**H-6**	7.18	7.19	7.12	7.13	7.21	7.12	7.14-7.09
**H-7**	7.27	7.33	7.30	7.33-7.29	7.32	7.28	7.31
**C-2**	124.3	123.2	140.7	139.7	123.2	140.2	140.6
**C-3**	102.2	108.8	104.3	104.8	108.5	104.3	104.5
**C-3a**	127.7	127.3	128.3	128.6	127.3	128.1	128.6
**C-4**	120.6	118.7	117.9	118.4	118.9	118.3	118.7
**C-5**	121.8	119.6	119.5	119.8	119.7	119.5	119.7
**C-6**	119.7	122.1	121.4	121.7	122.2	121.5	121.6
**C-7**	111.1	111.2	110.6	110.8	111.3	110.5	110.6
**C-7a**	135.7	136.7	136.9	136.9	136.2	135.4	135.4

Copies of ^{1}H and ^{13}C NMR spectra

1H NMR (400 MHz, CDCl3)

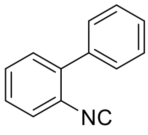

1a

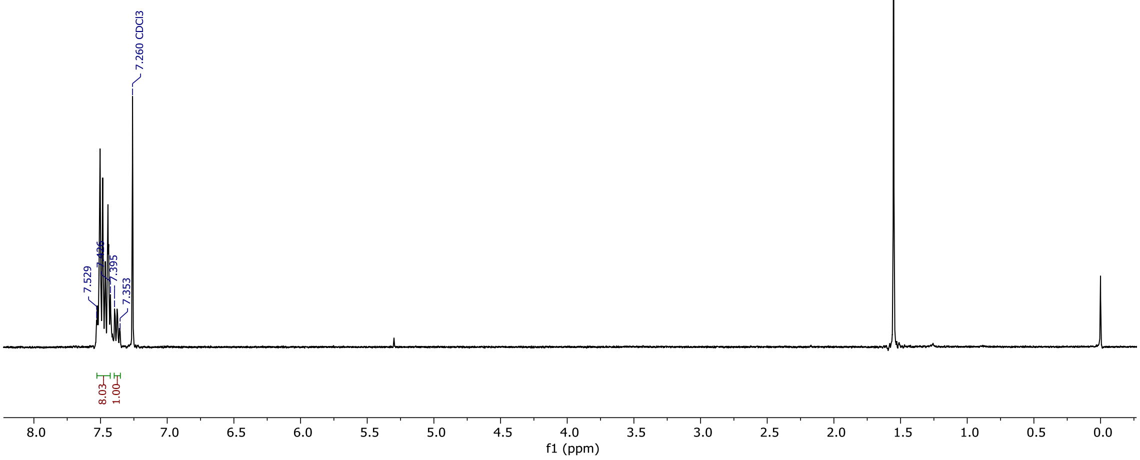

13C NMR (101 MHz, CDCl3)

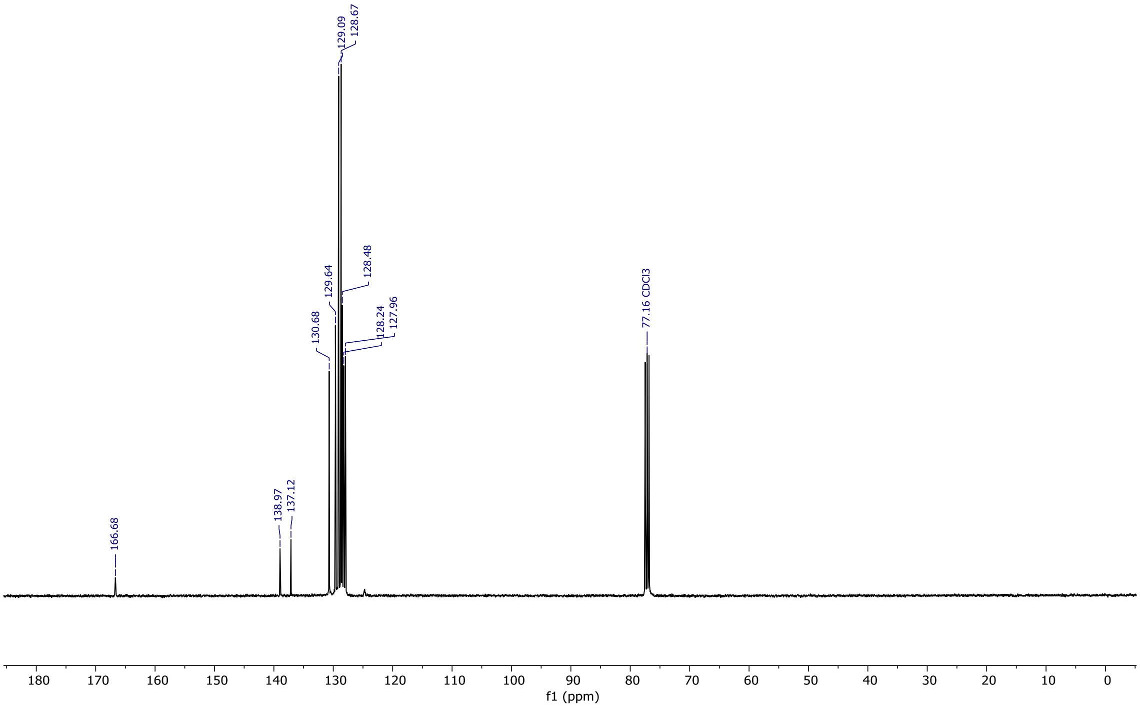

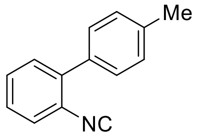

1b

1H NMR (400 MHz, CDCl3)

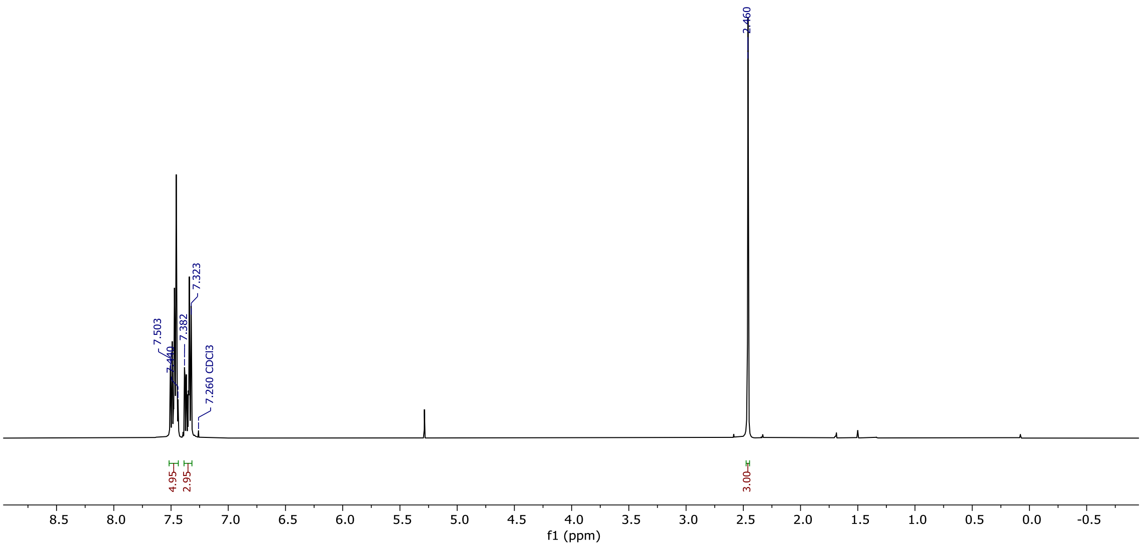

13C NMR (101 MHz, CDCl3)

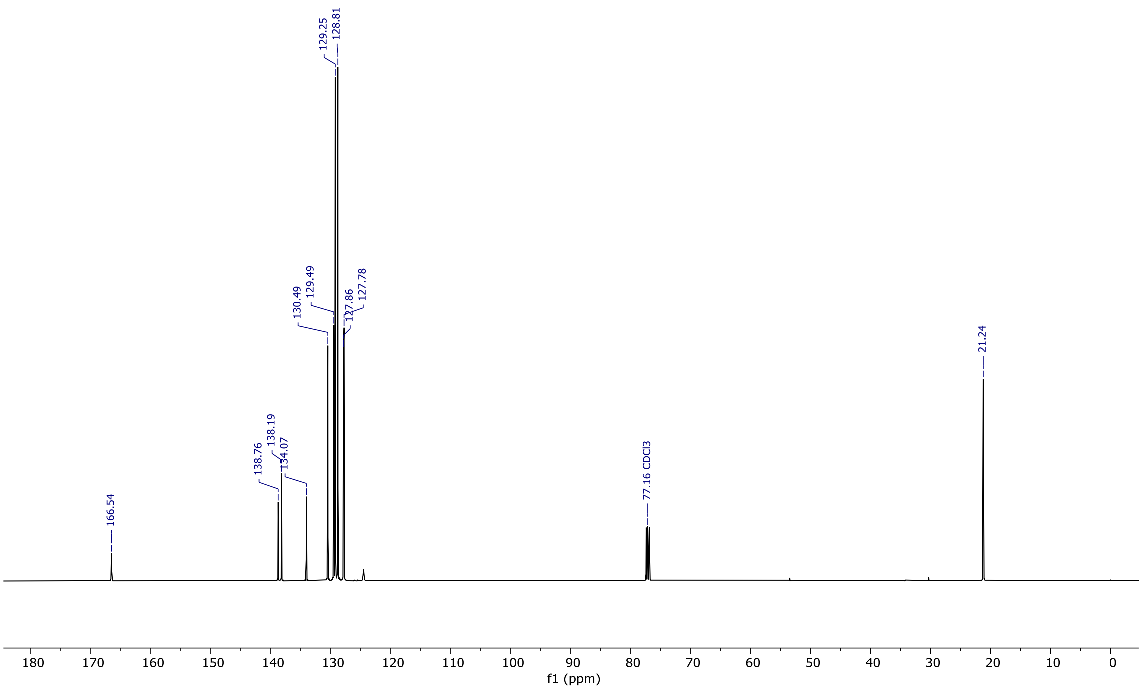

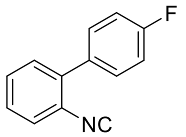
F
NC

1c

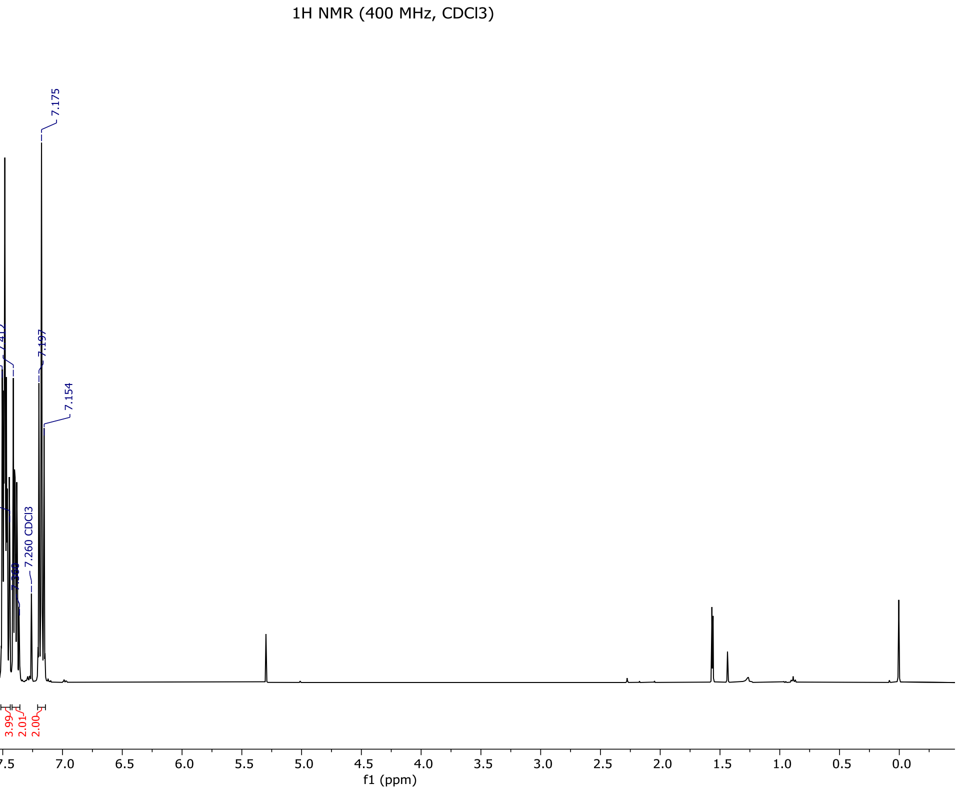
1H NMR (400 MHz, CDCl3)
7.505
7.441
7.412
7.260 CDCl3
7.197
7.175
7.154
3.99
2.01
2.00
f1 (ppm)

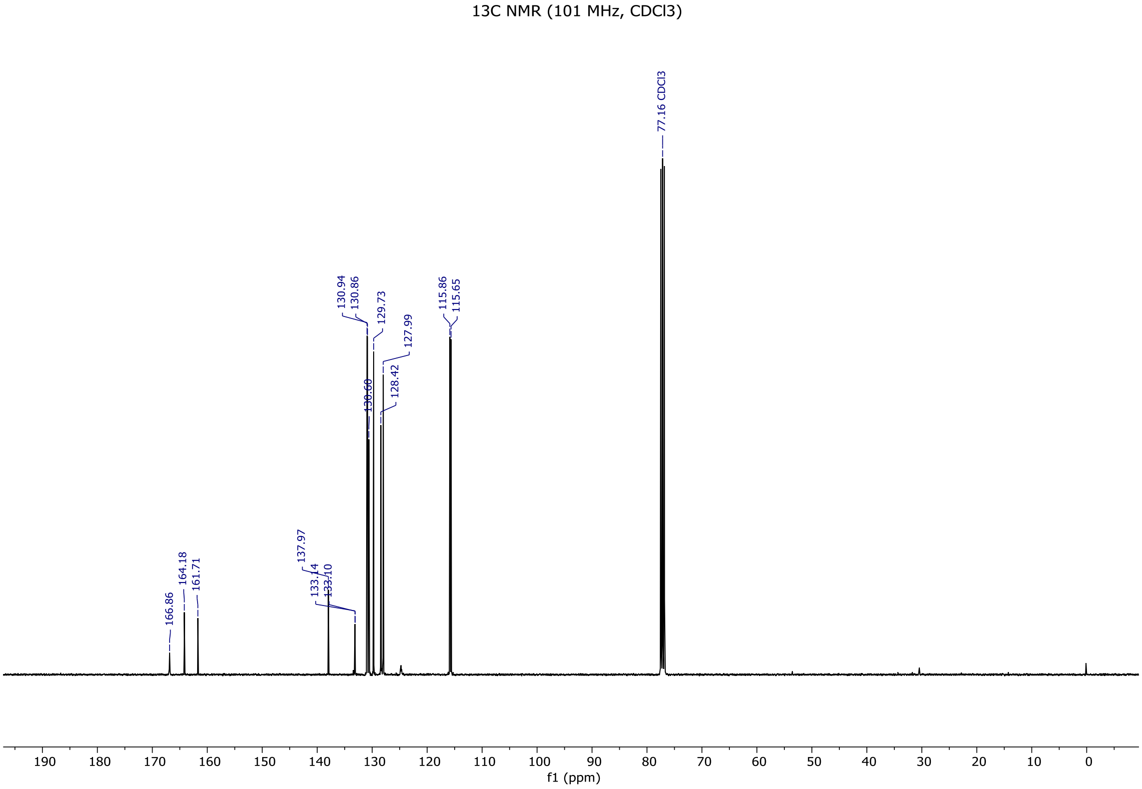
13C NMR (101 MHz, CDCl3)
166.86
164.18
161.71
137.97
133.14
133.10
130.94
130.86
130.60
129.73
128.42
127.99
115.86
115.65
77.16 CDCl3
f1 (ppm)

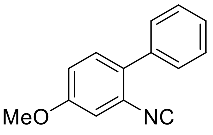

1d

1H NMR (400 MHz, CDCl3)

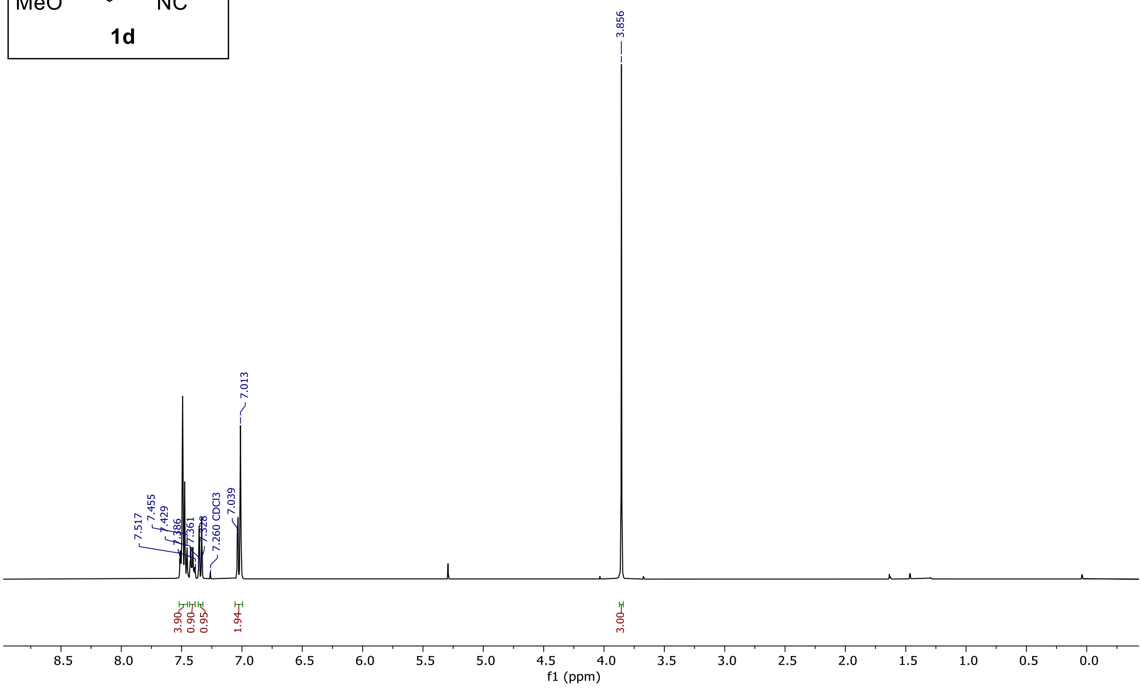

13C NMR (101 MHz, CDCl3)

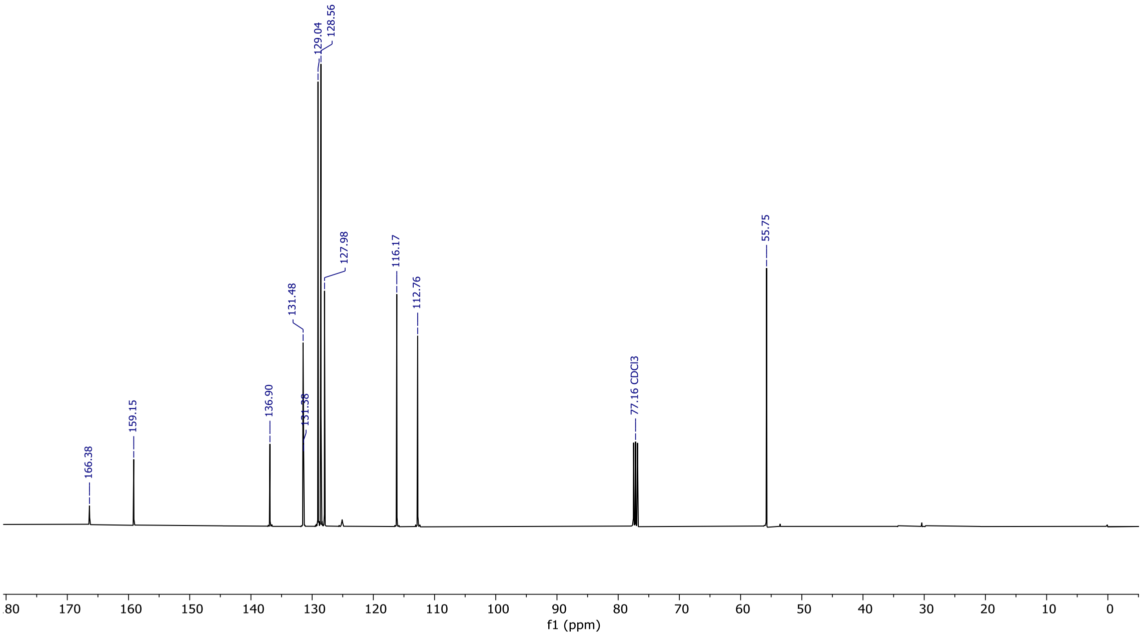

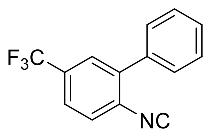

1e

1H NMR (400 MHz, CDCl3)

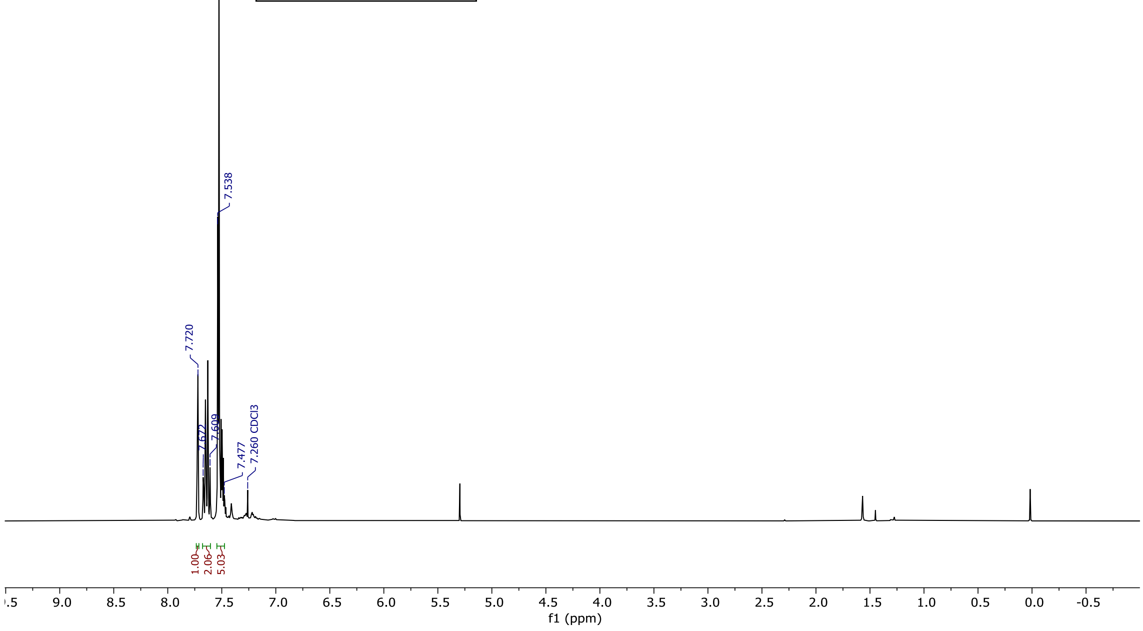

13C NMR (101 MHz, CDCl3)

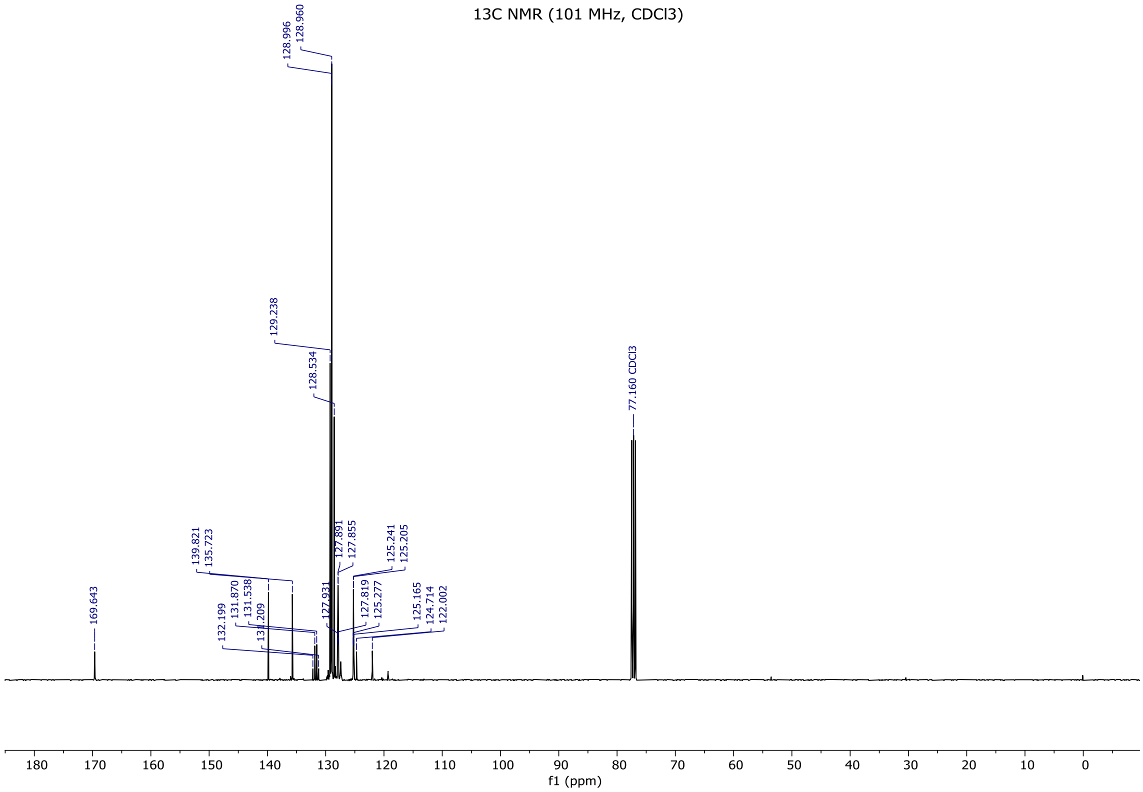

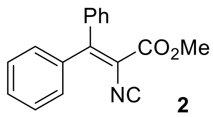

2

1H NMR (400 MHz, CDCl3)

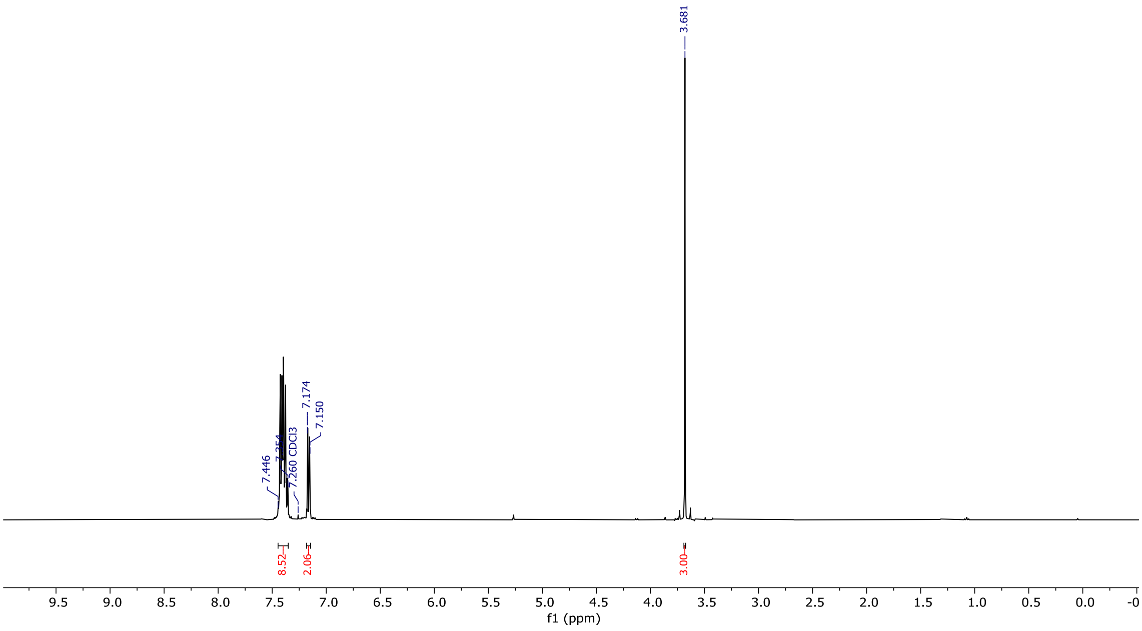

13C NMR (101 MHz, CDCl3)

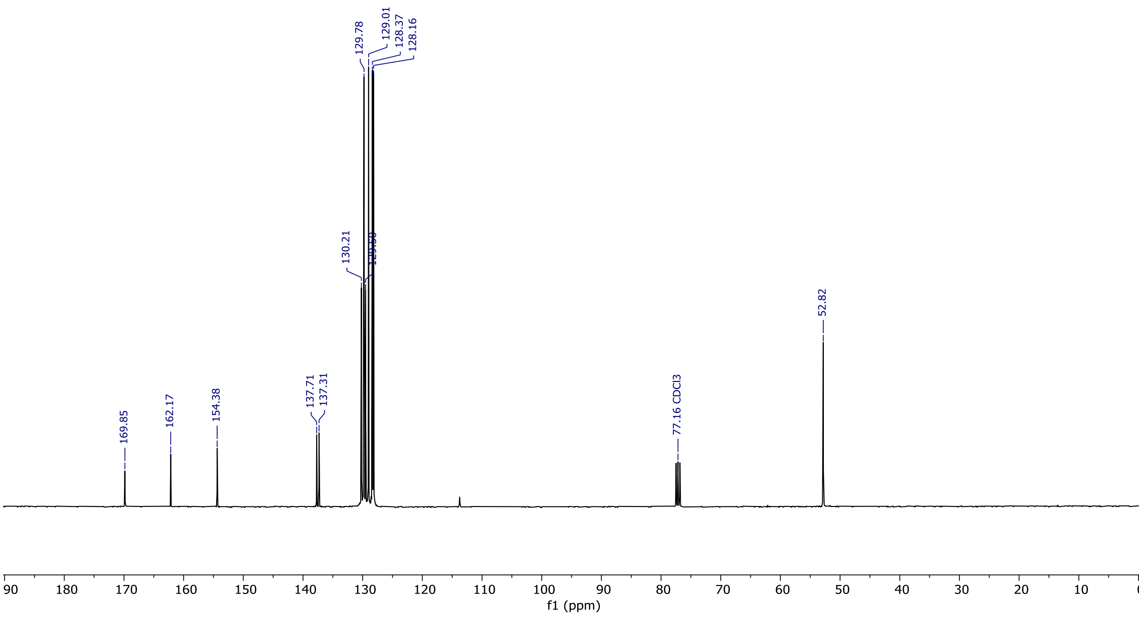

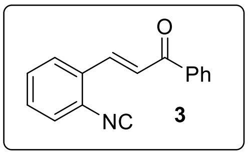

1H NMR (400 MHz, CDCl3)

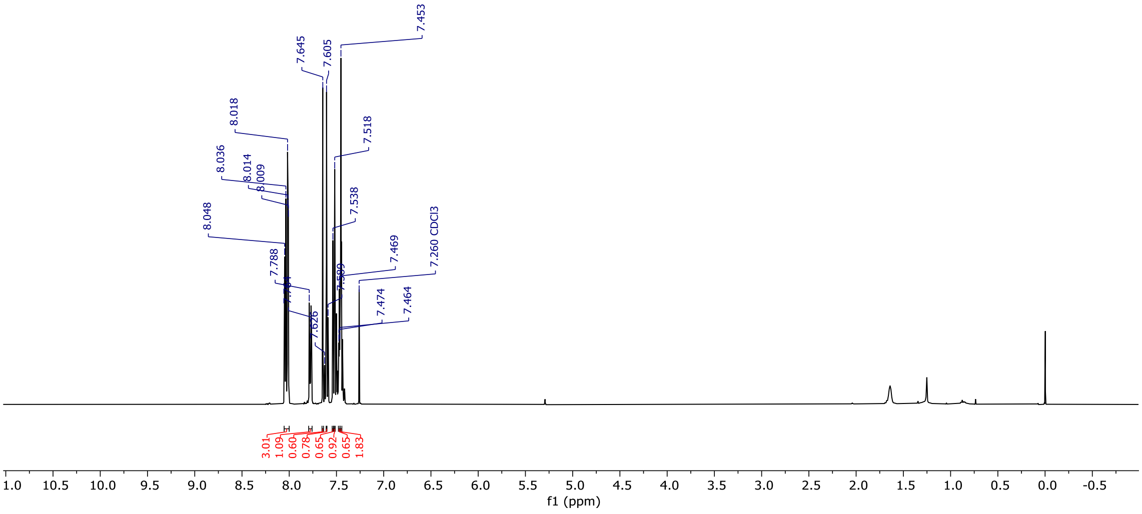

13C NMR (101 MHz, CDCl3)

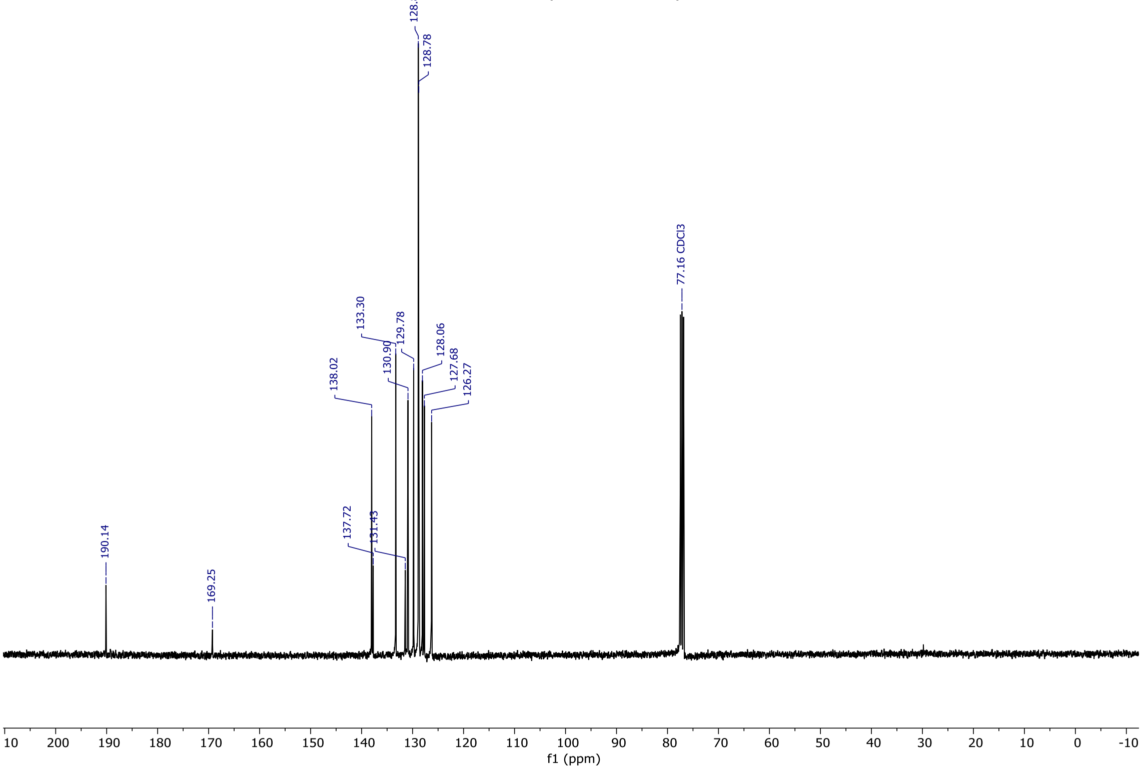

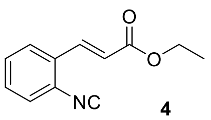

4

1H NMR (400 MHz, CDCl3)

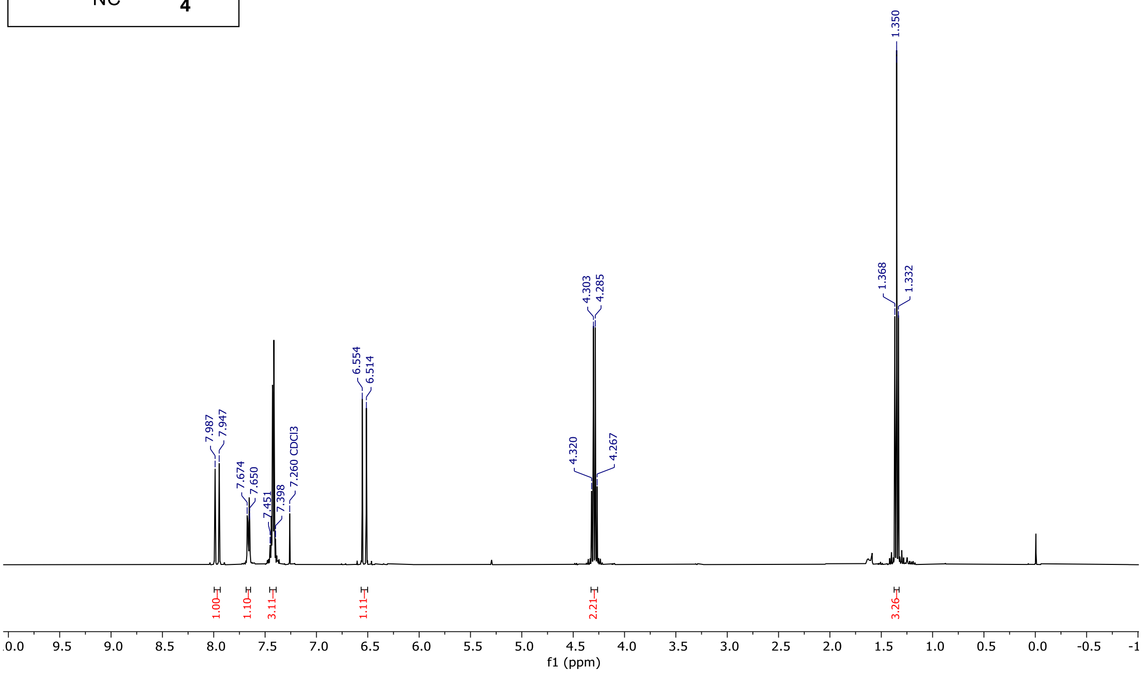

13C NMR (101 MHz, CDCl3)

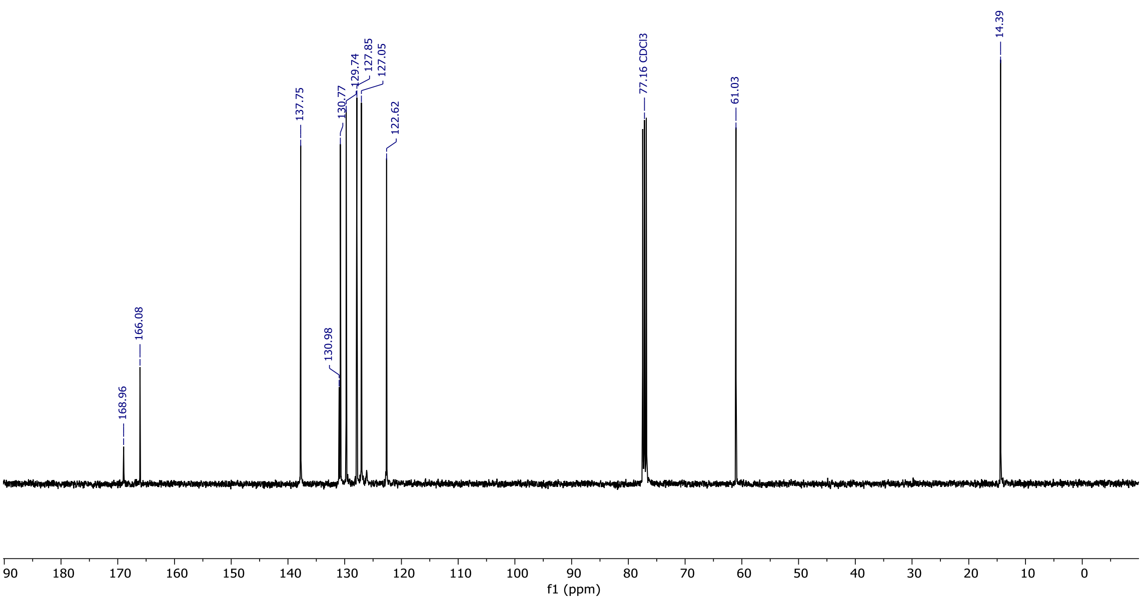

1H NMR (400 MHz, CDCl3)

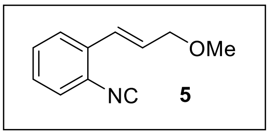

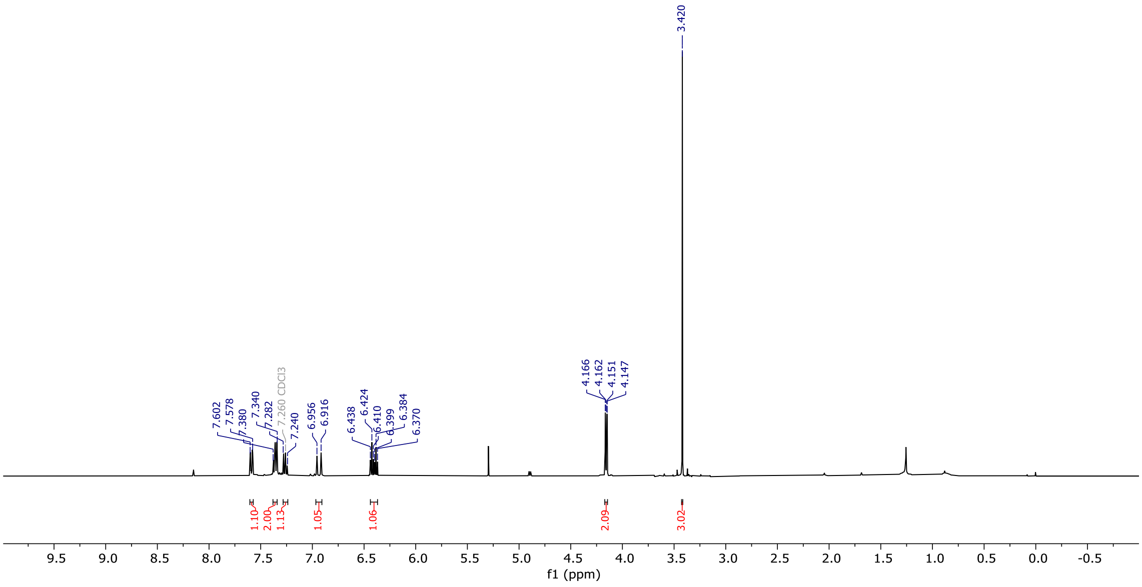

13C NMR (101 MHz, CDCl3)

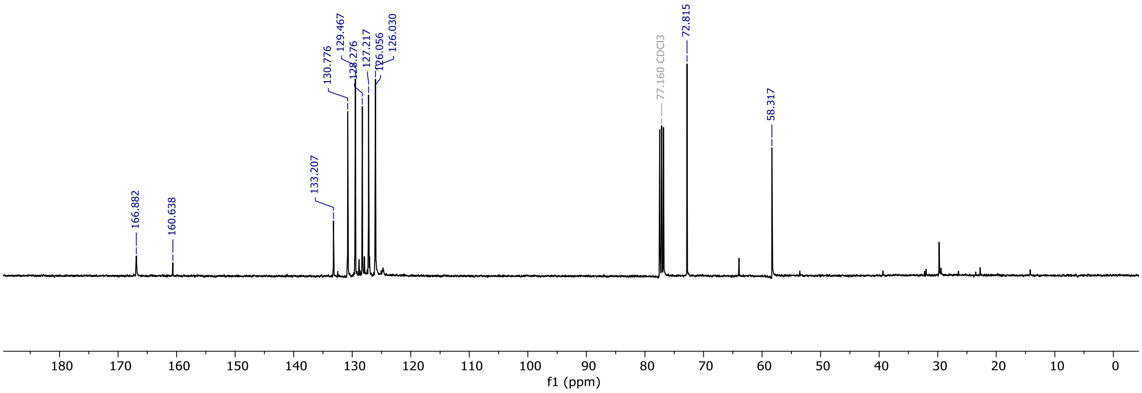

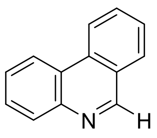

6

1H NMR (400 MHz, CDCl3)

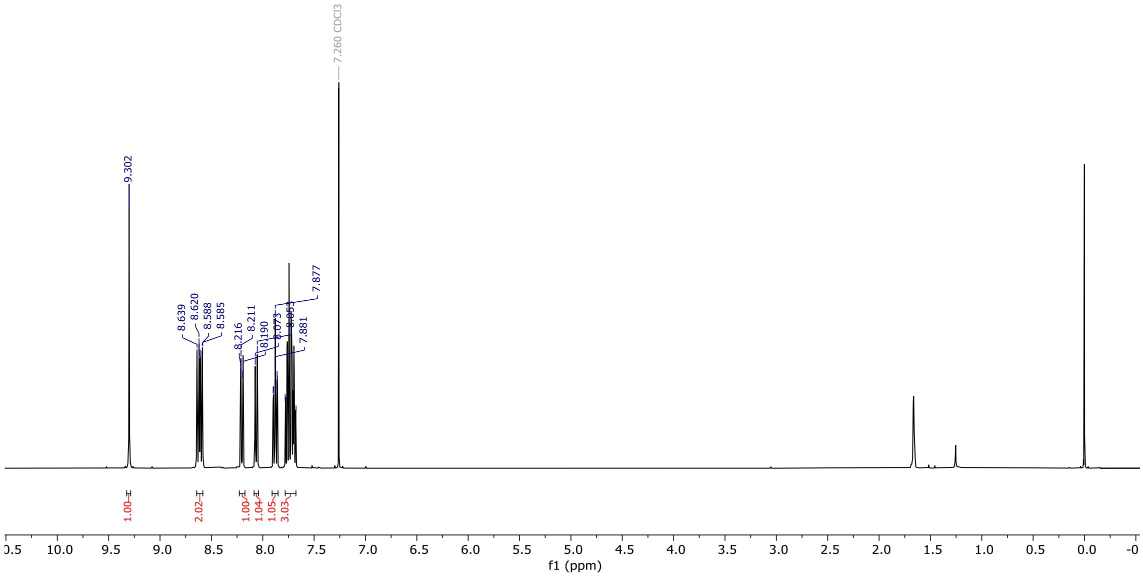

13C NMR (101 MHz, CDCl3)

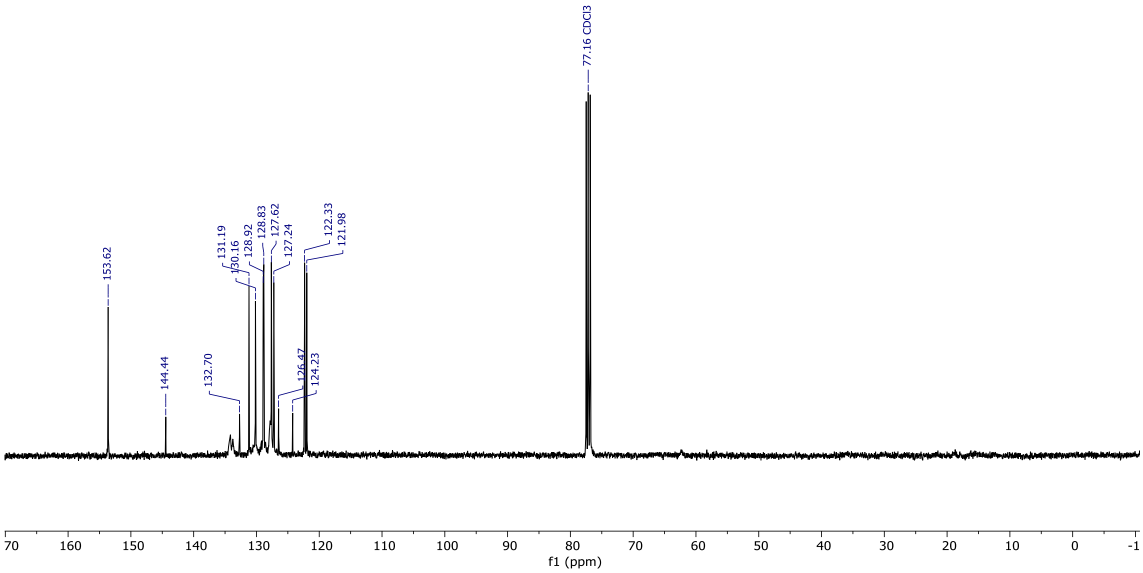

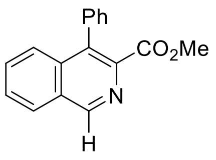

7

1H NMR (400 MHz, CDCl3)

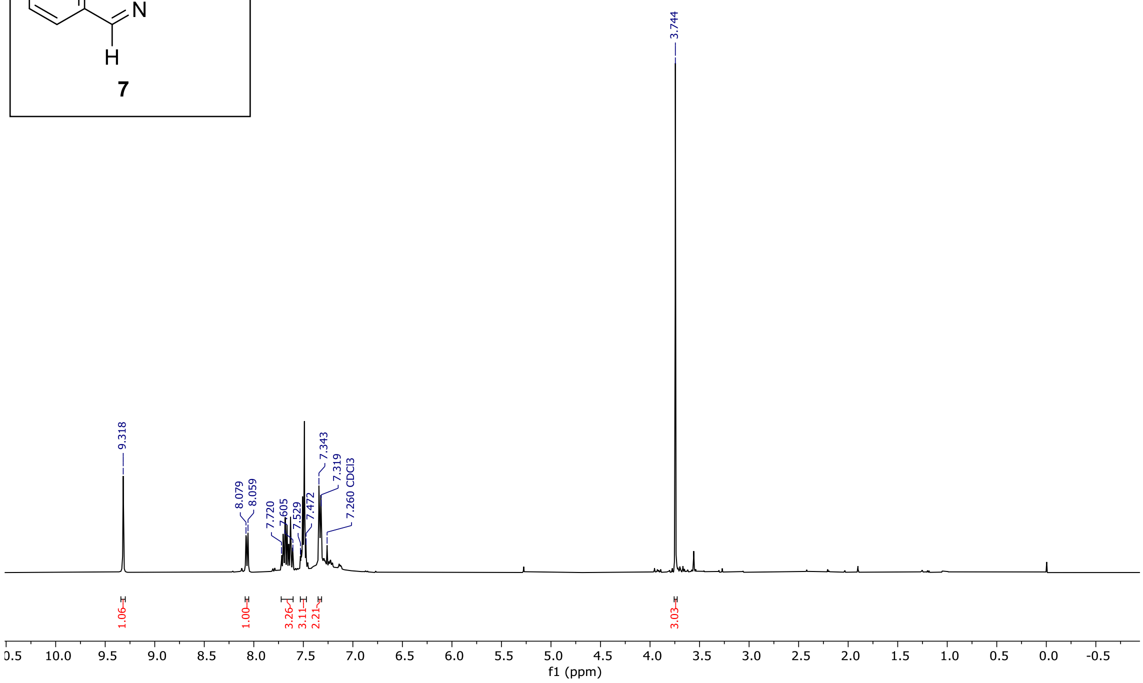

13C NMR (101 MHz, CDCl3)

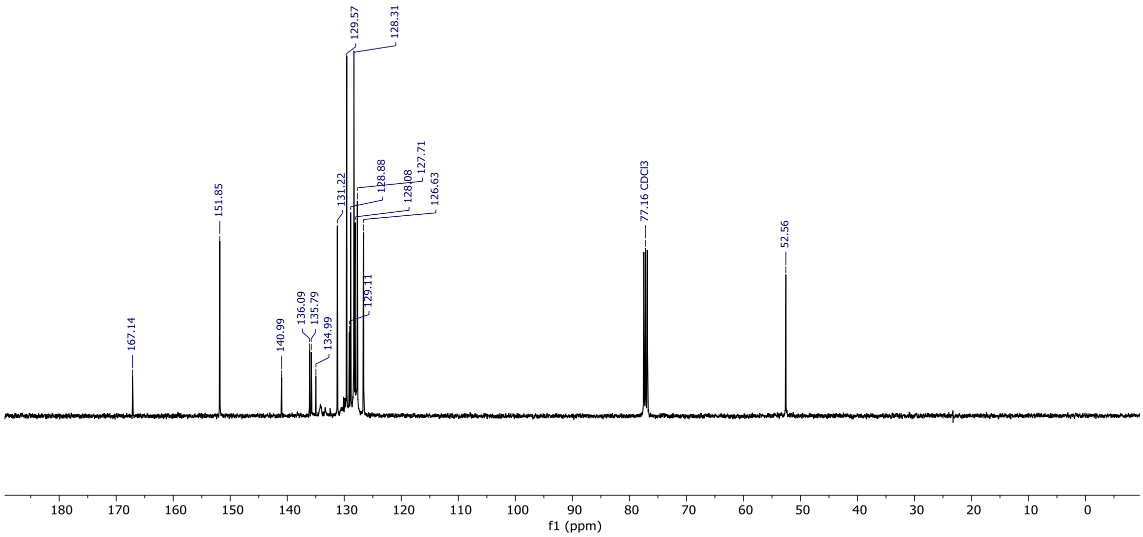

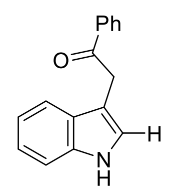
Ph
O
H
N
H

8

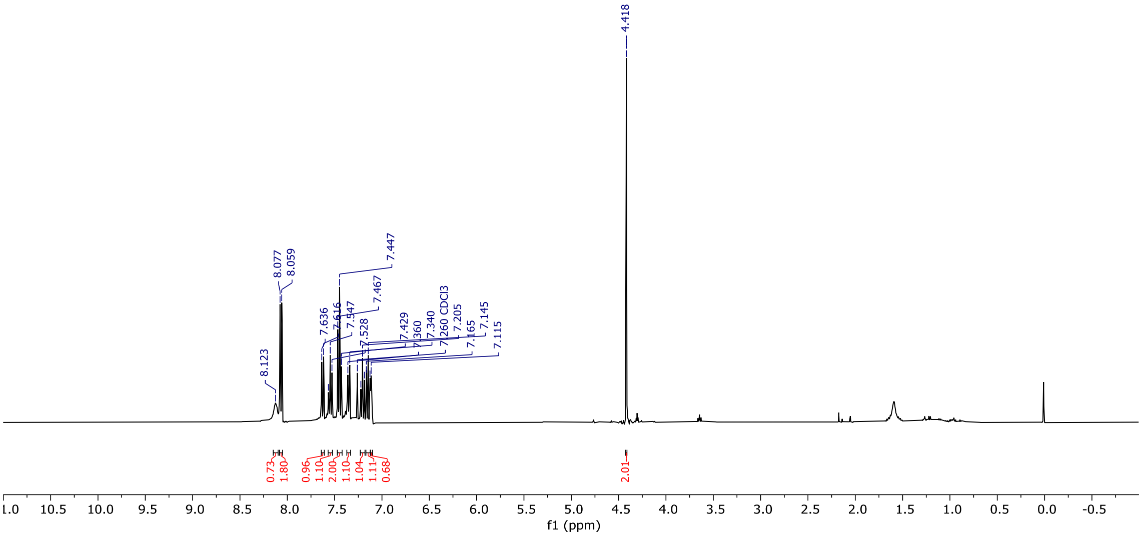
1H NMR (400 MHz, CDCl3)
4.418
f1 (ppm)

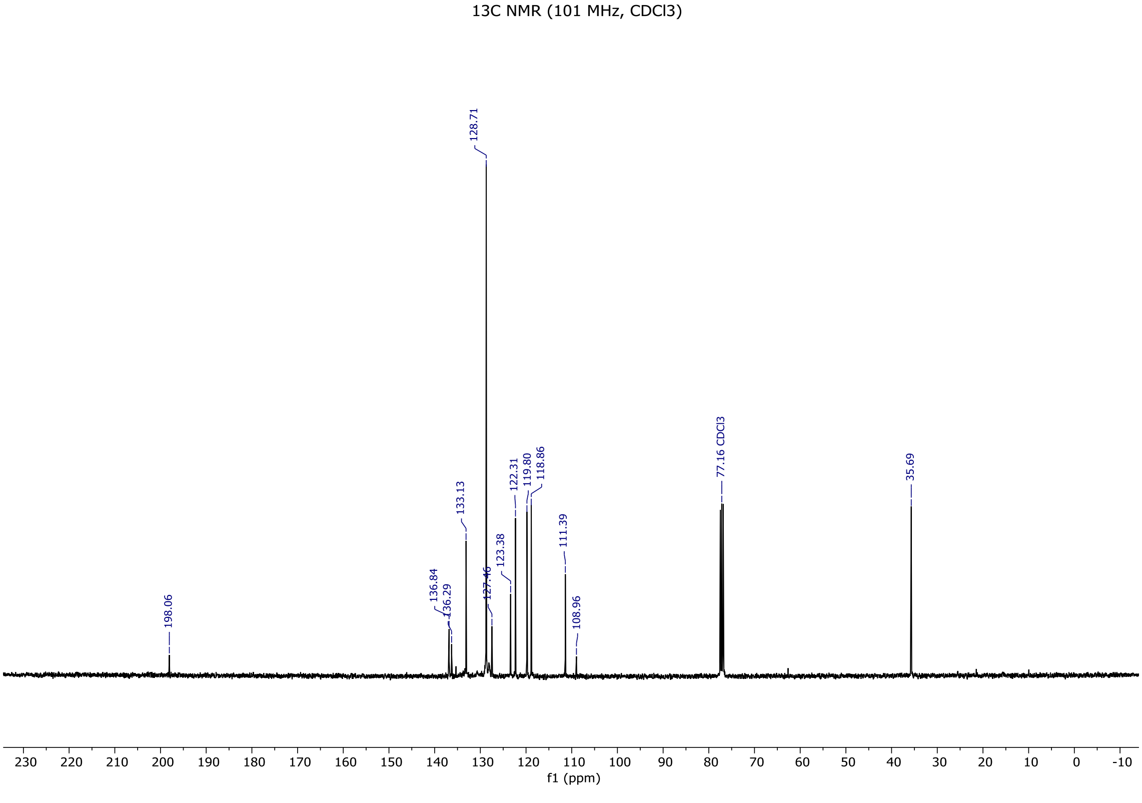
13C NMR (101 MHz, CDCl3)
198.06
136.84
136.29
133.13
128.71
127.46
123.38
122.31
119.80
118.86
111.39
108.96
77.16 CDCl3
35.69
f1 (ppm)

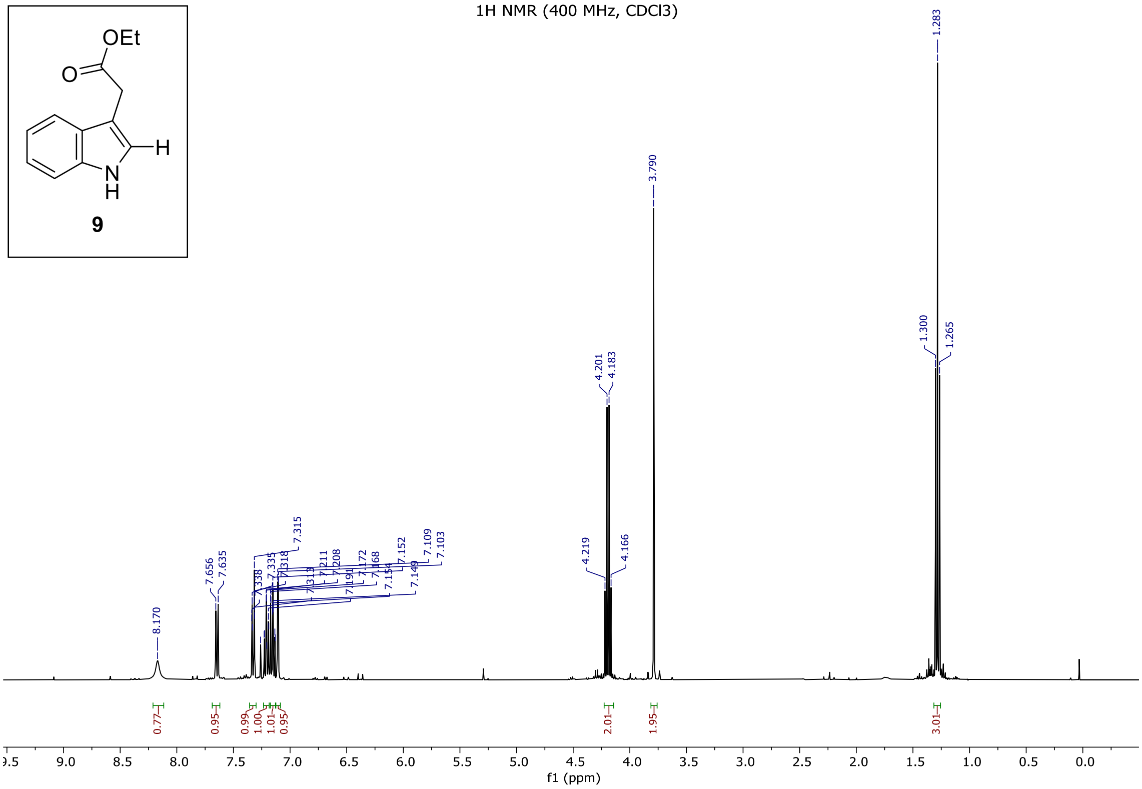

13C NMR (101 MHz, CDCl3)

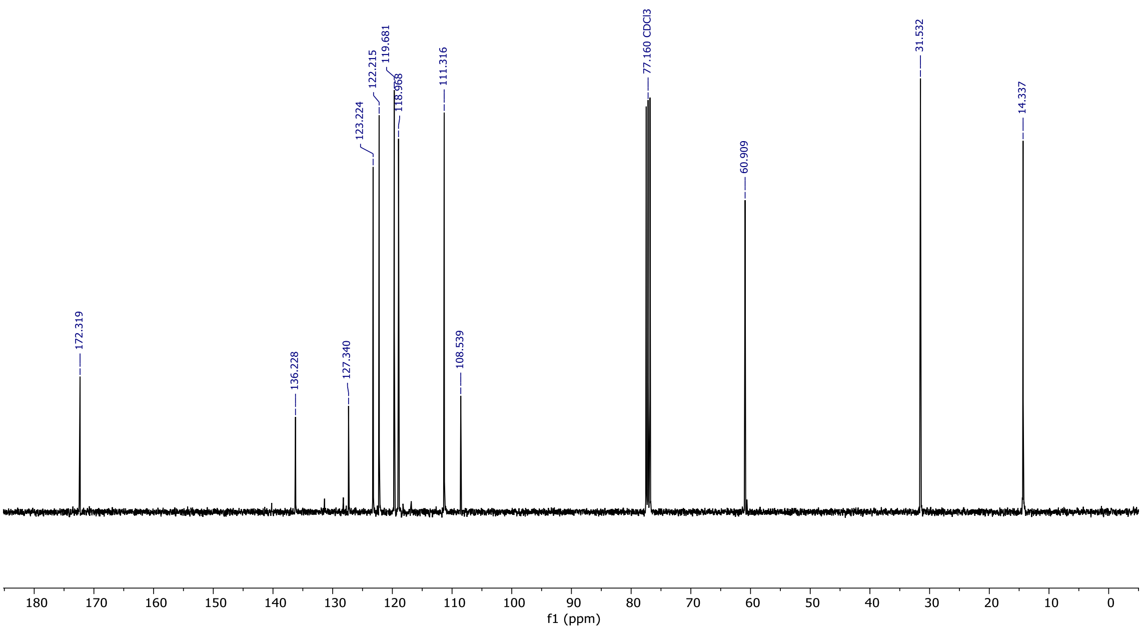

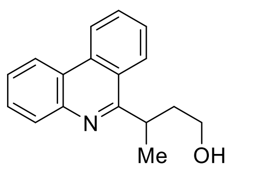

6a

1H NMR (400 MHz, CDCl3)

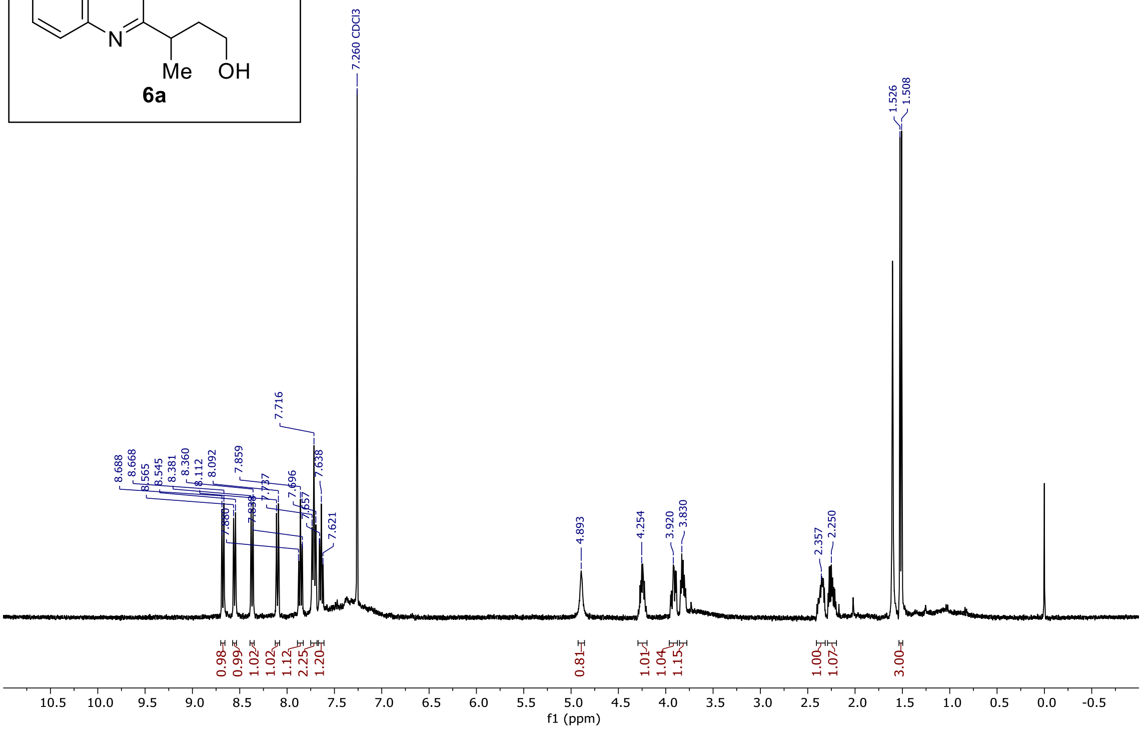

13C NMR (101 MHz, CDCl3)

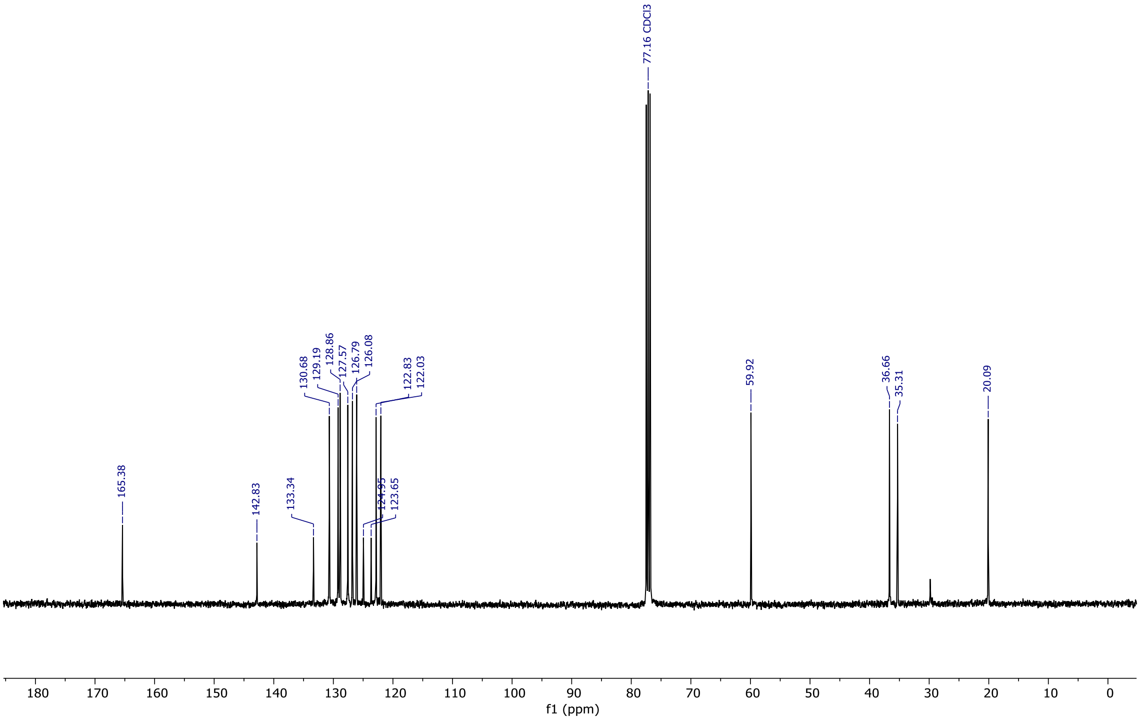

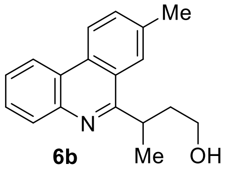

6b

1H NMR (400 MHz, CDCl3)

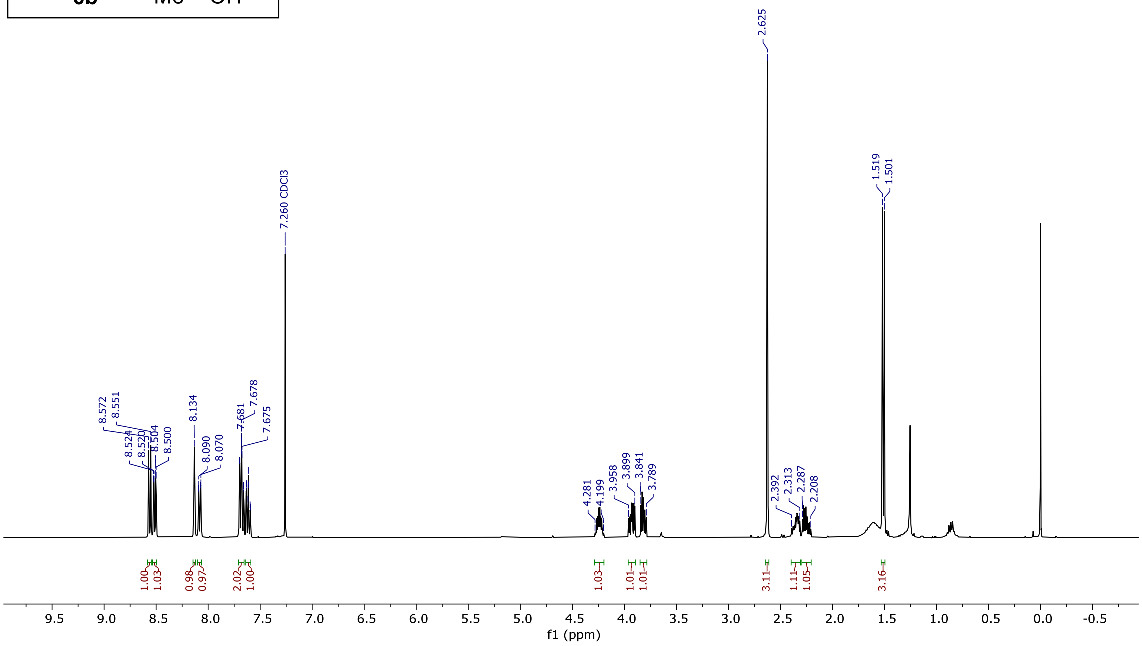

13C NMR (101 MHz, CDCl3)

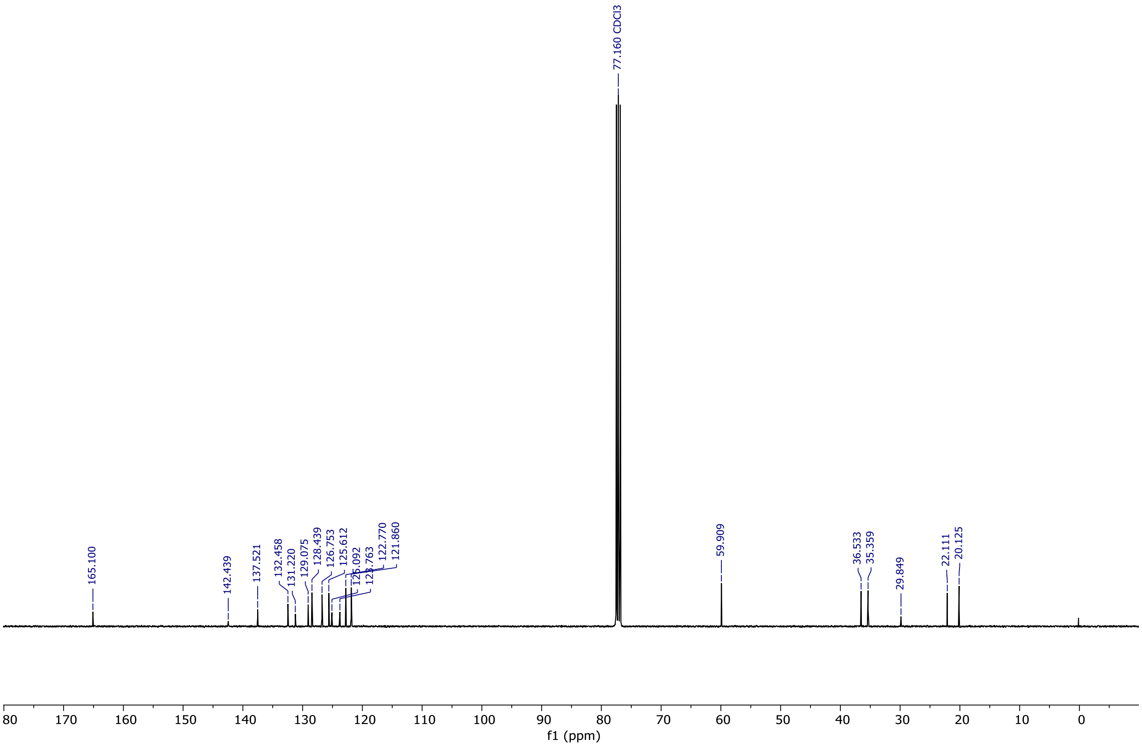

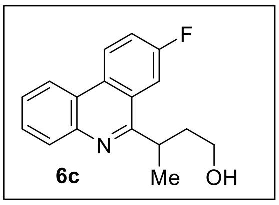

1H NMR (400 MHz, CDCl3)

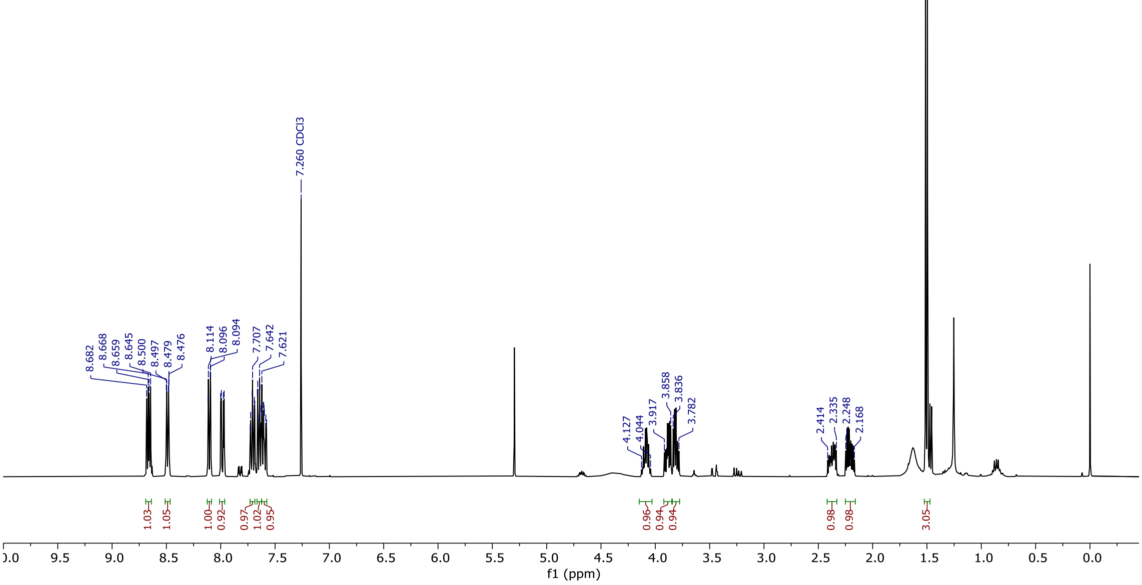

13C NMR (101 MHz, CDCl3)

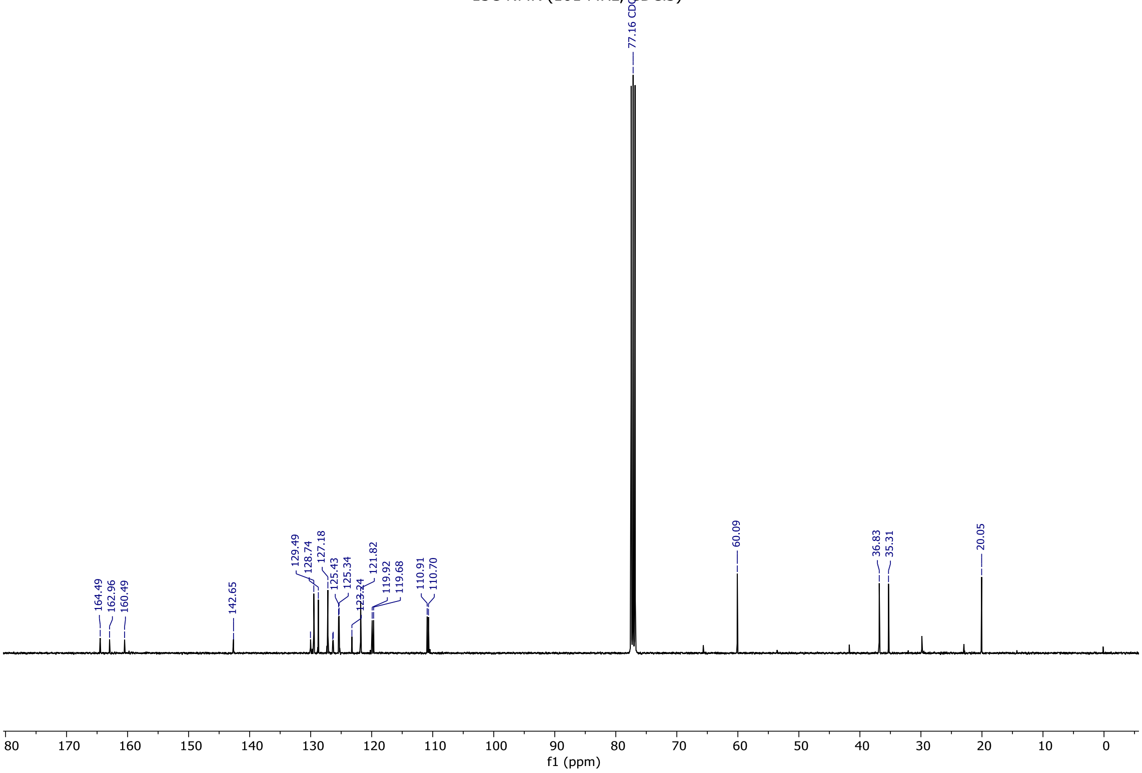

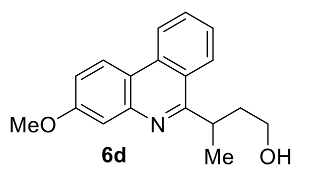

6d

1H NMR (400 MHz, CDCl3)

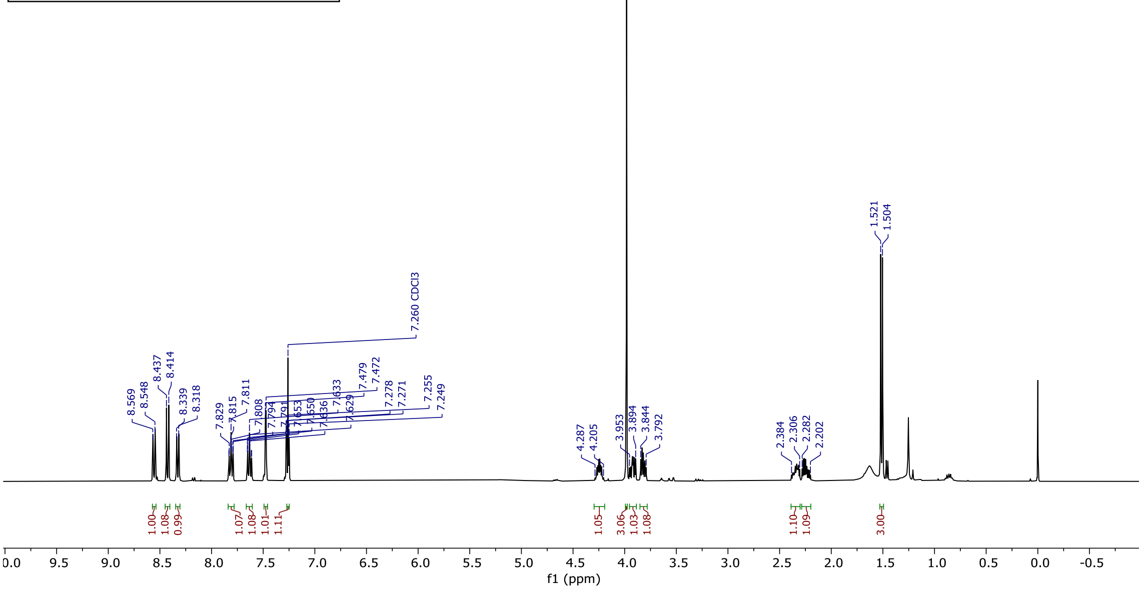

13C NMR (101 MHz, CDCl3)

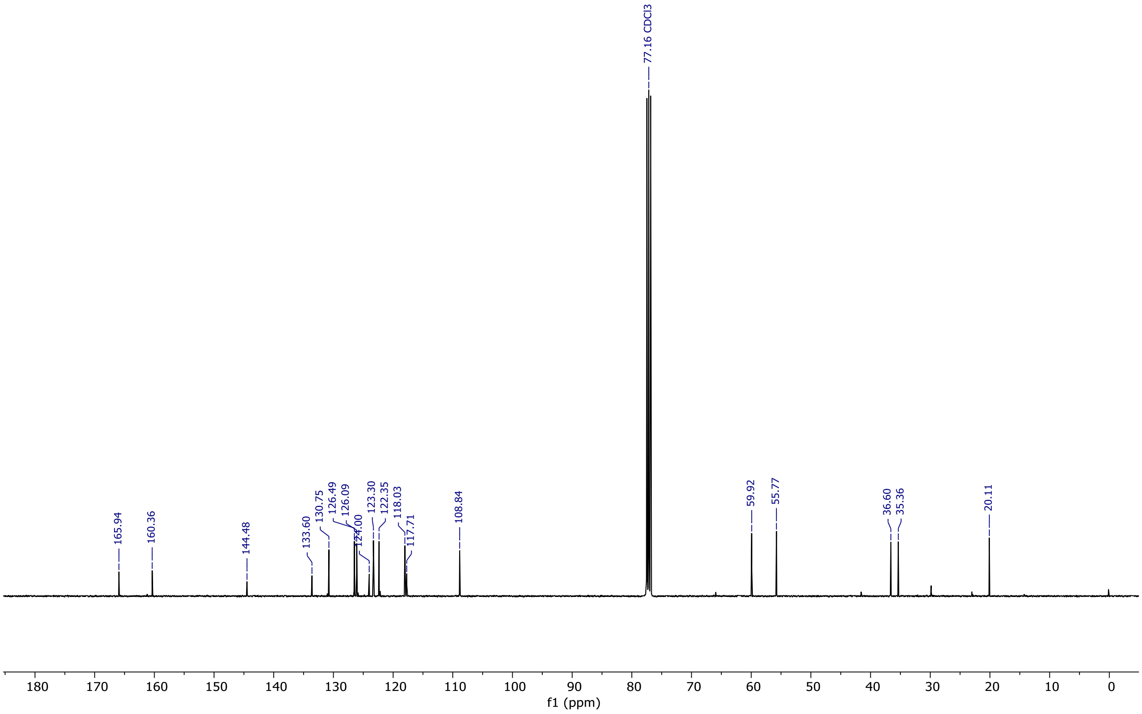

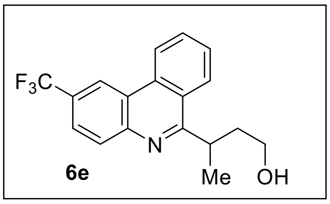
F3C
N
Me
OH
6e

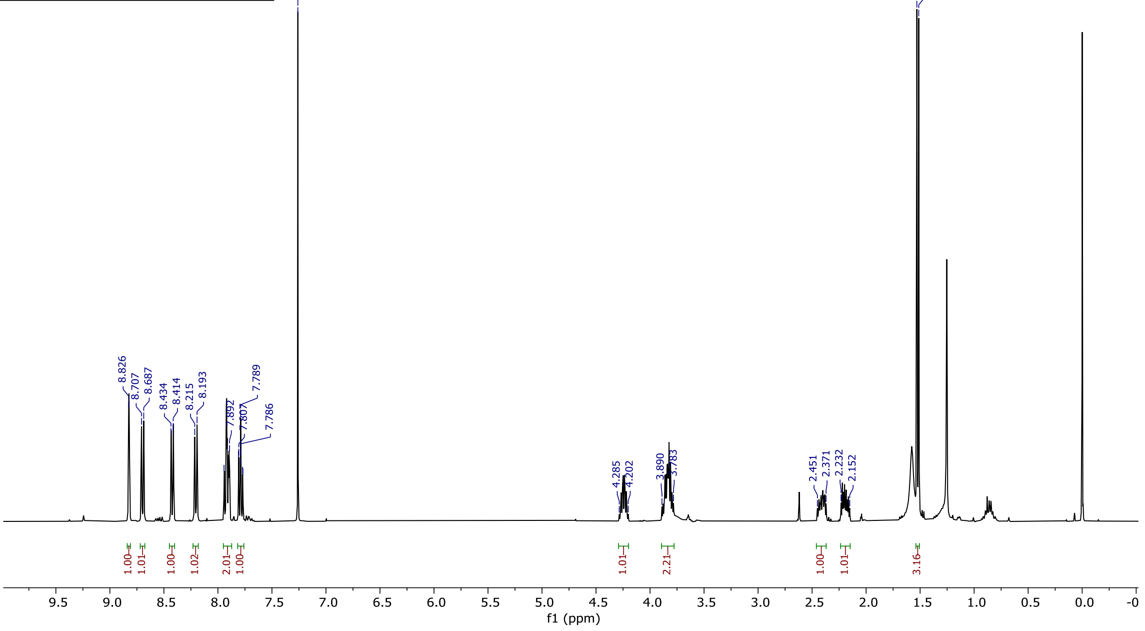
1H NMR (400 MHz, CDCl3)
7.260 CDCl3
1.530
1.513
8.826
8.707
8.687
8.434
8.414
8.215
8.193
7.892
7.807
7.789
7.786
4.285
4.202
3.890
3.783
2.451
2.371
2.232
2.152
1.00
1.01
1.00
1.02
2.01
1.00
1.01
2.21
1.00
1.01
3.16
f1 (ppm)

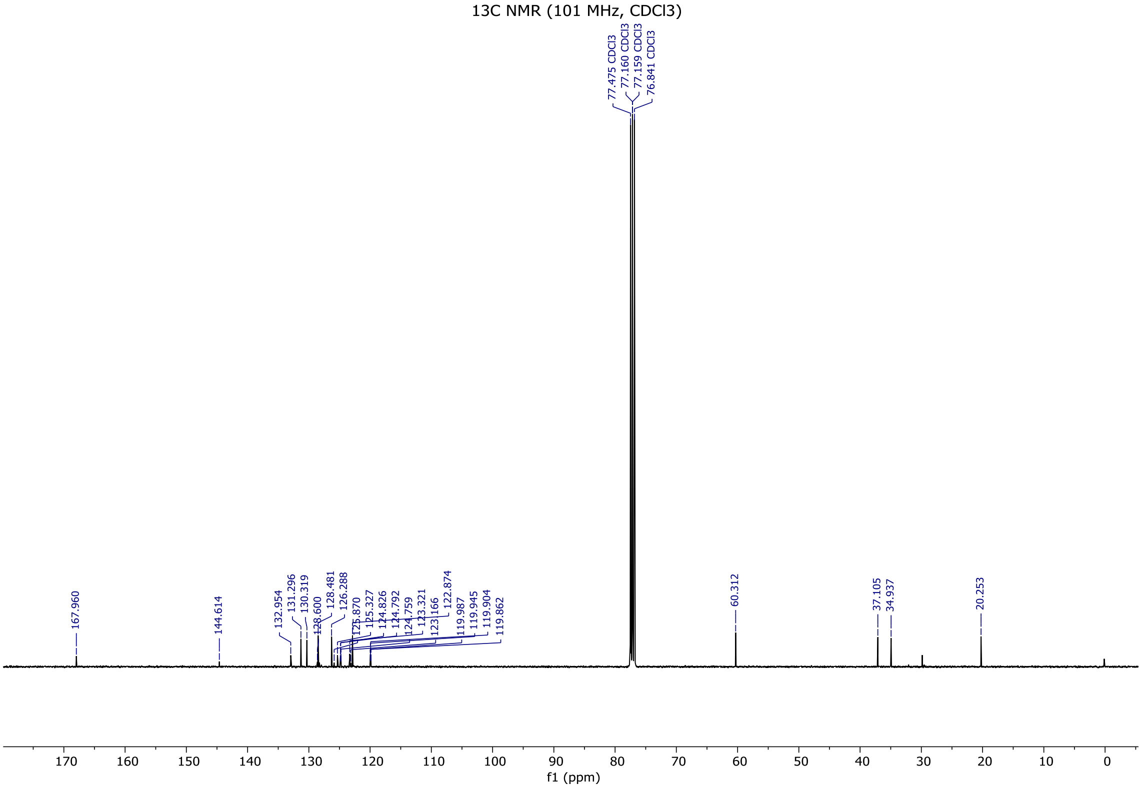
13C NMR (101 MHz, CDCl3)
77.475 CDCl3
77.160 CDCl3
77.159 CDCl3
76.841 CDCl3
167.960
144.614
132.954
131.296
130.319
128.600
128.481
126.288
125.870
125.327
124.826
124.792
124.759
123.321
123.166
122.874
119.987
119.945
119.904
119.862
60.312
37.105
34.937
20.253
f1 (ppm)

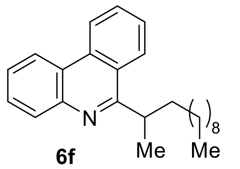

6f

1H NMR (400 MHz, CDCl3)

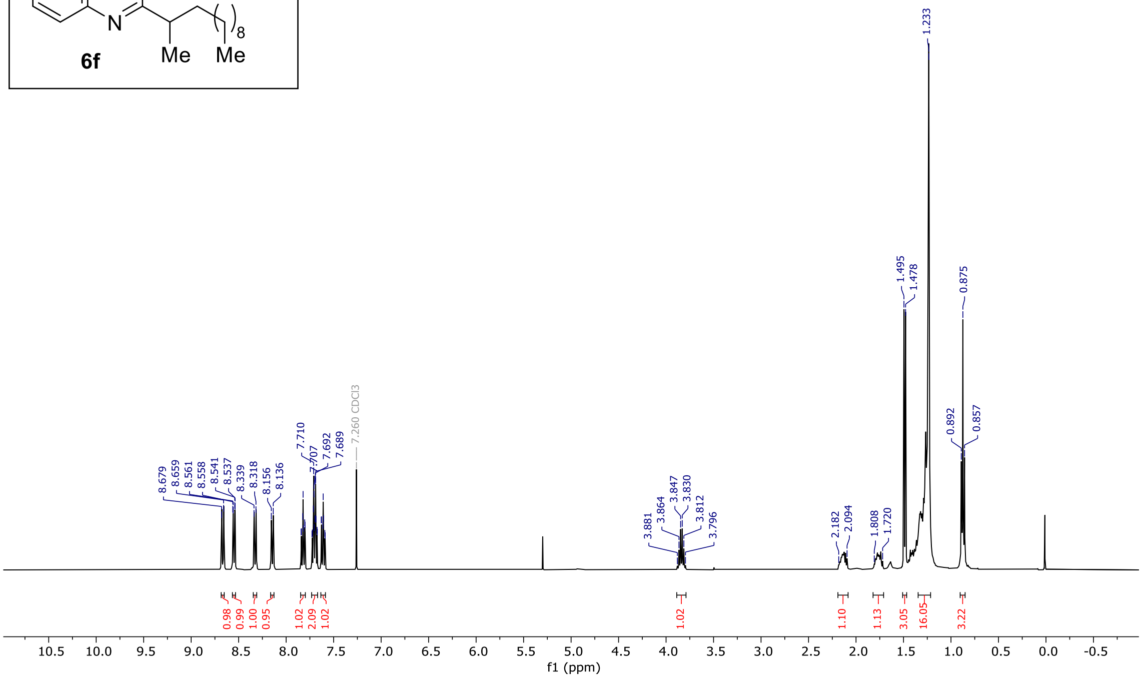

13C NMR (101 MHz, CDCl3)

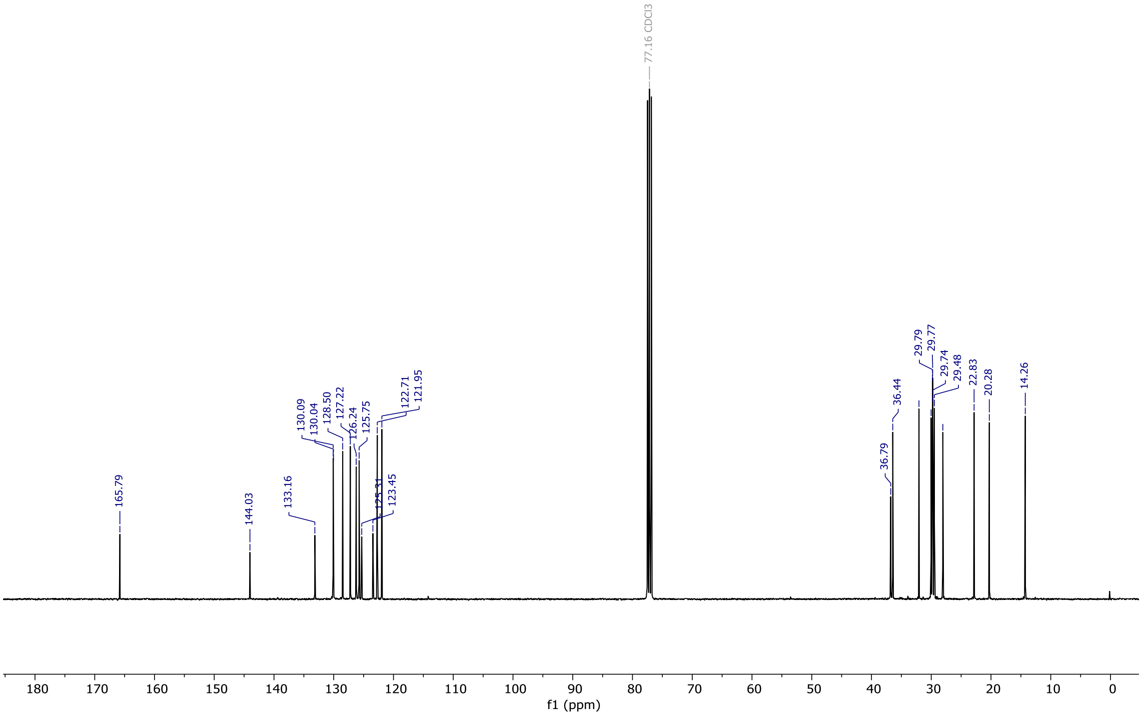

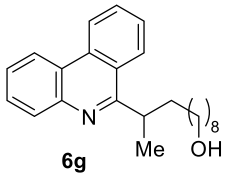

6g

1H NMR (400 MHz, CDCl3)

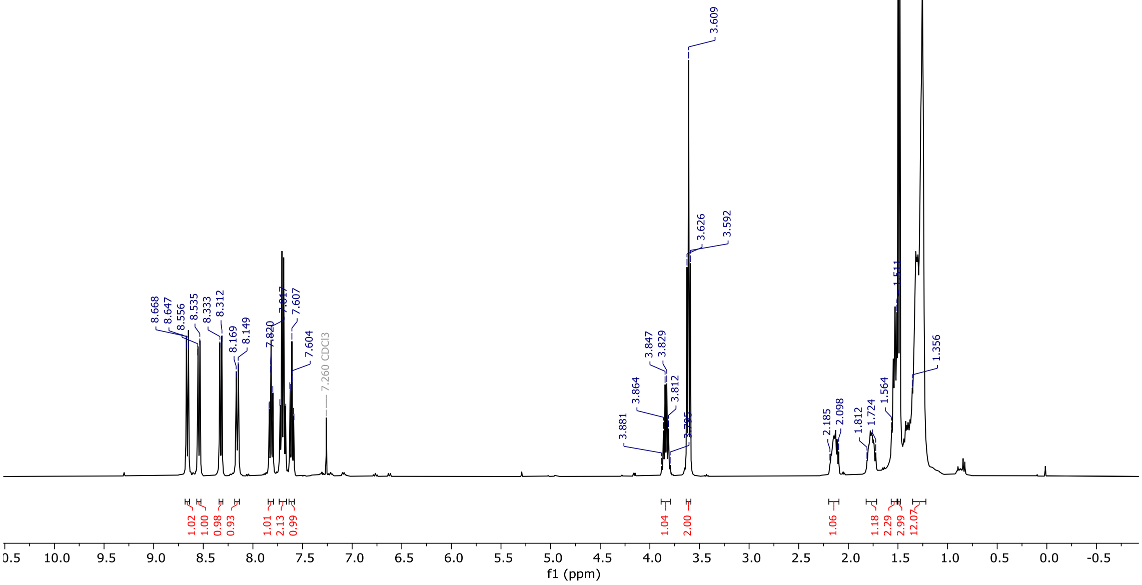

13C NMR (101 MHz, CDCl3)

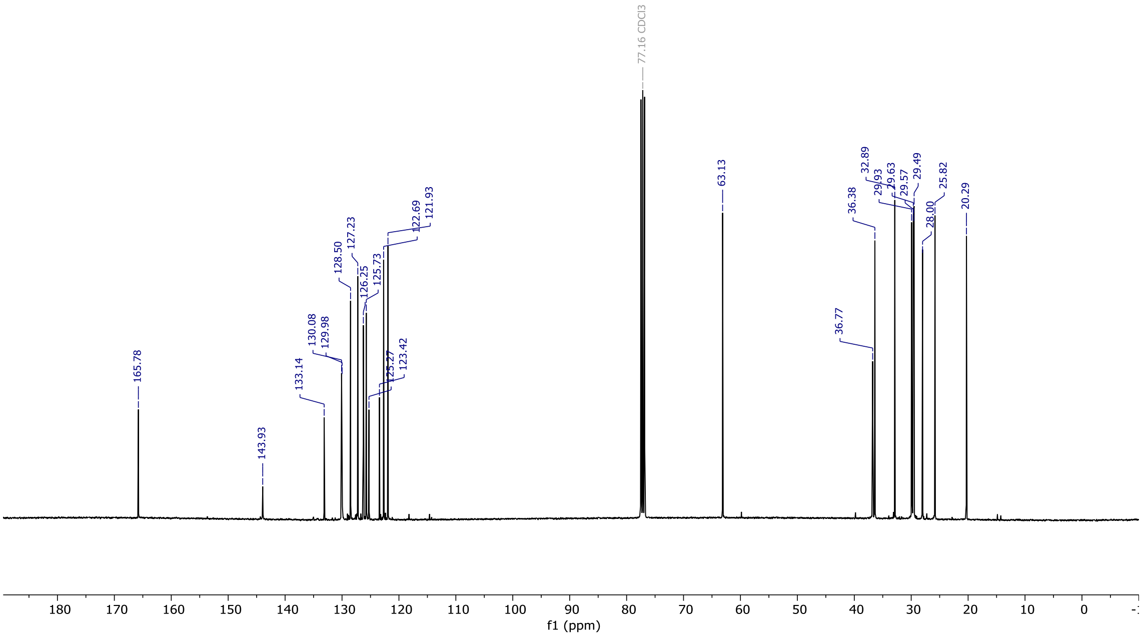

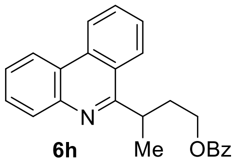

6h

1H NMR (400 MHz, CDCl3)

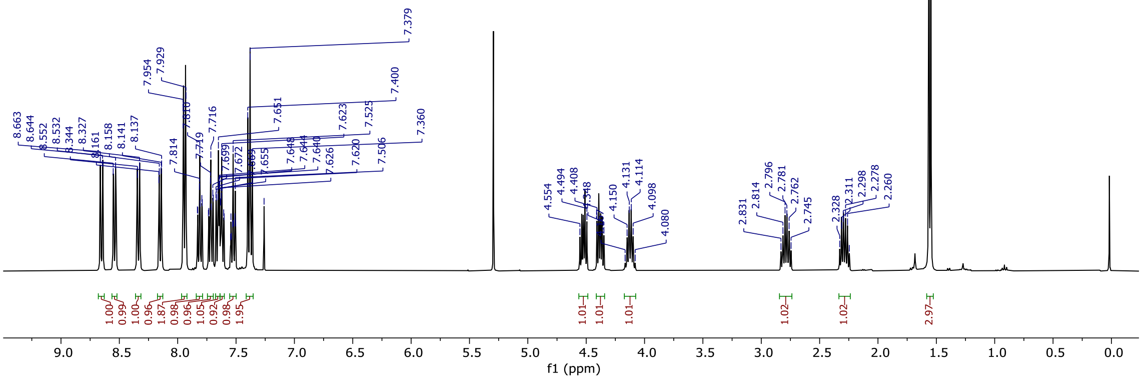

13C NMR (101 MHz, CDCl3)

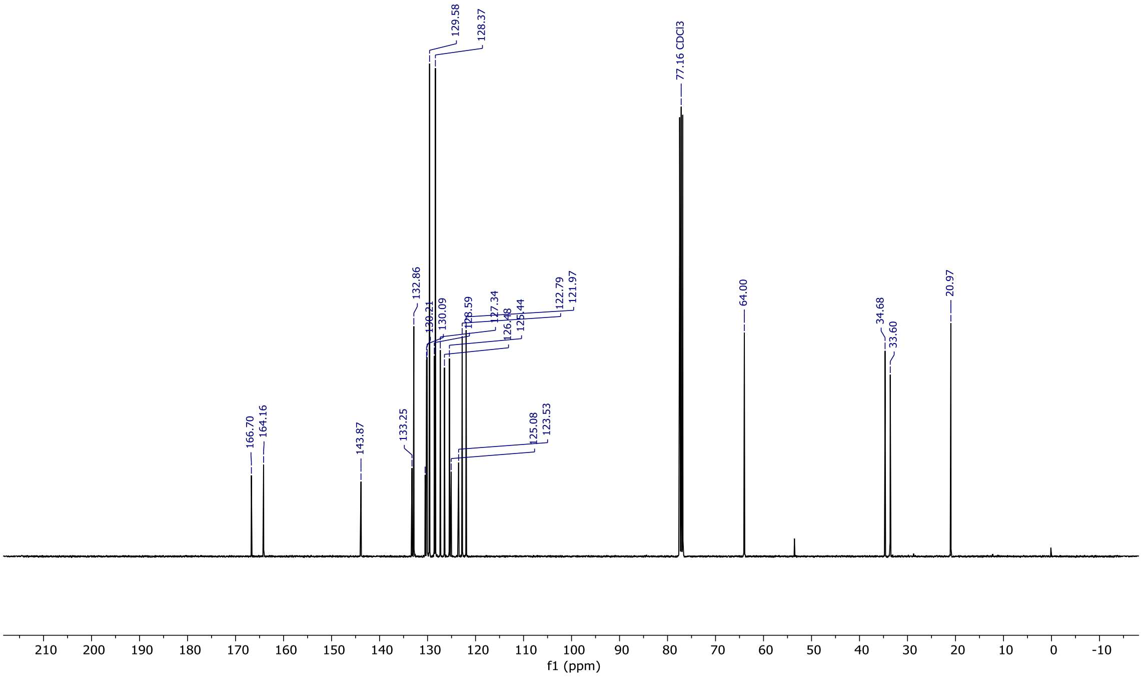

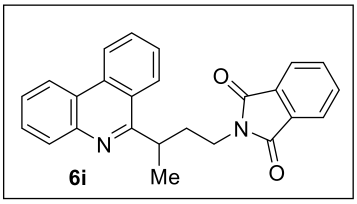

1H NMR (400 MHz, CDCl3)

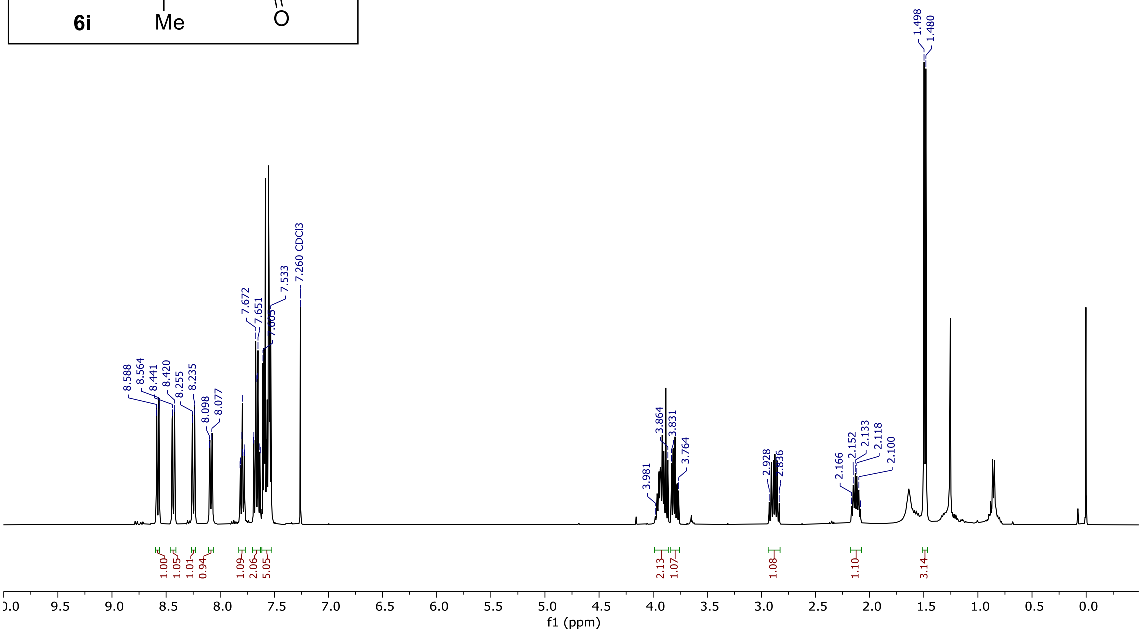

13C NMR (101 MHz, CDCl3)

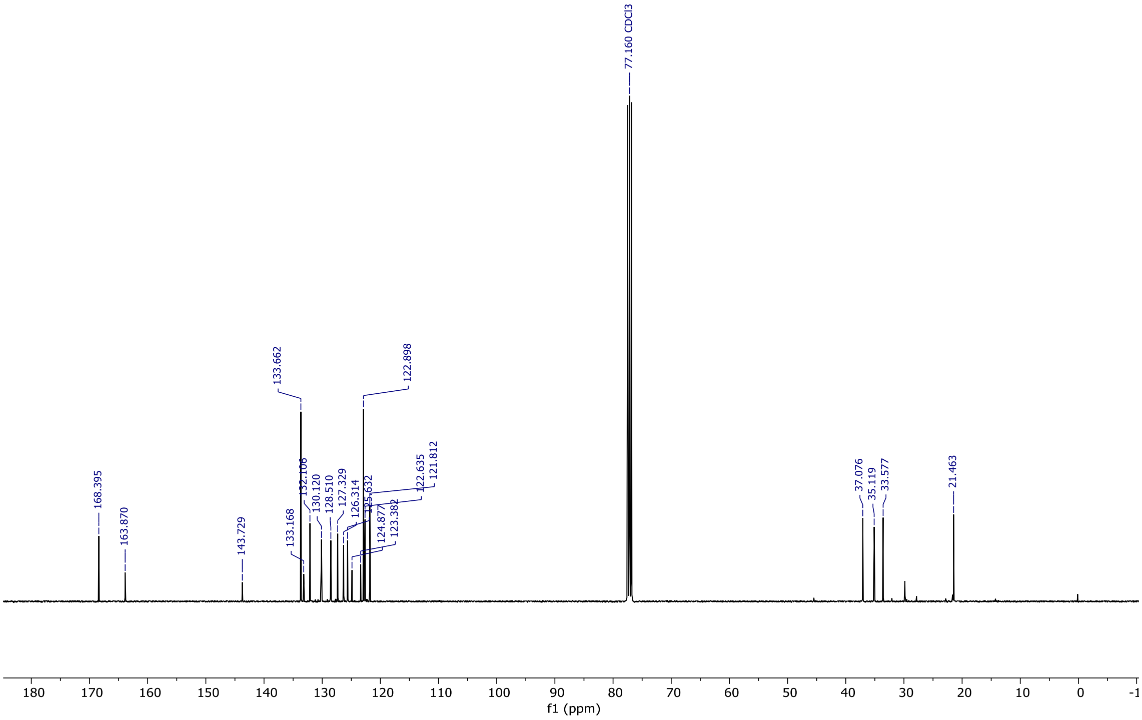

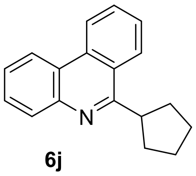

6j

1H NMR (400 MHz, CDCl3)

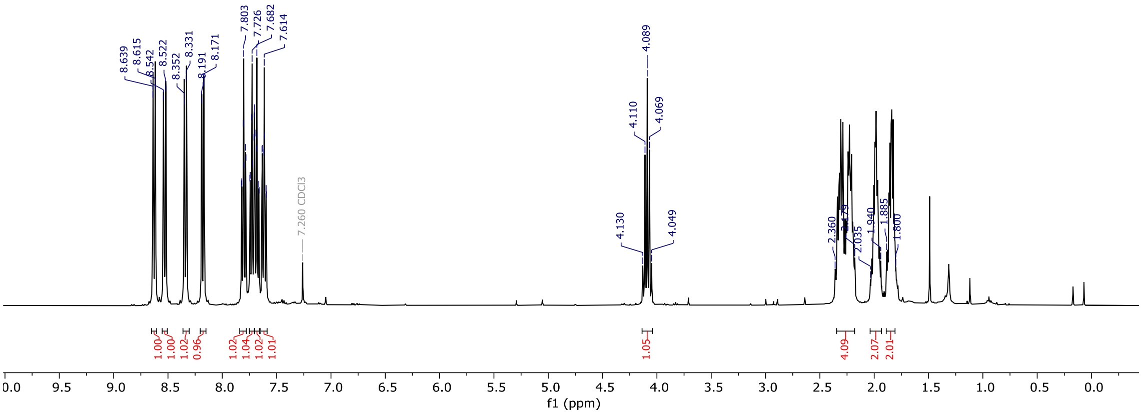

13C NMR (101 MHz, CDCl3)

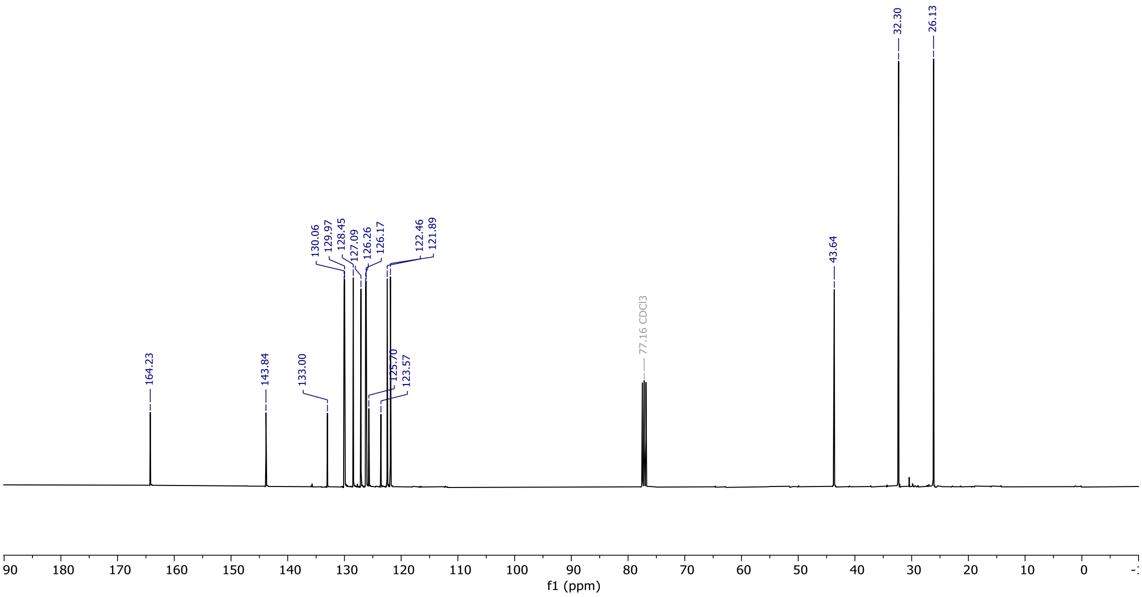

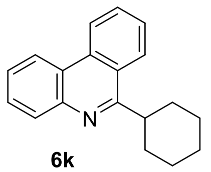

6k

1H NMR (400 MHz, CDCl3)

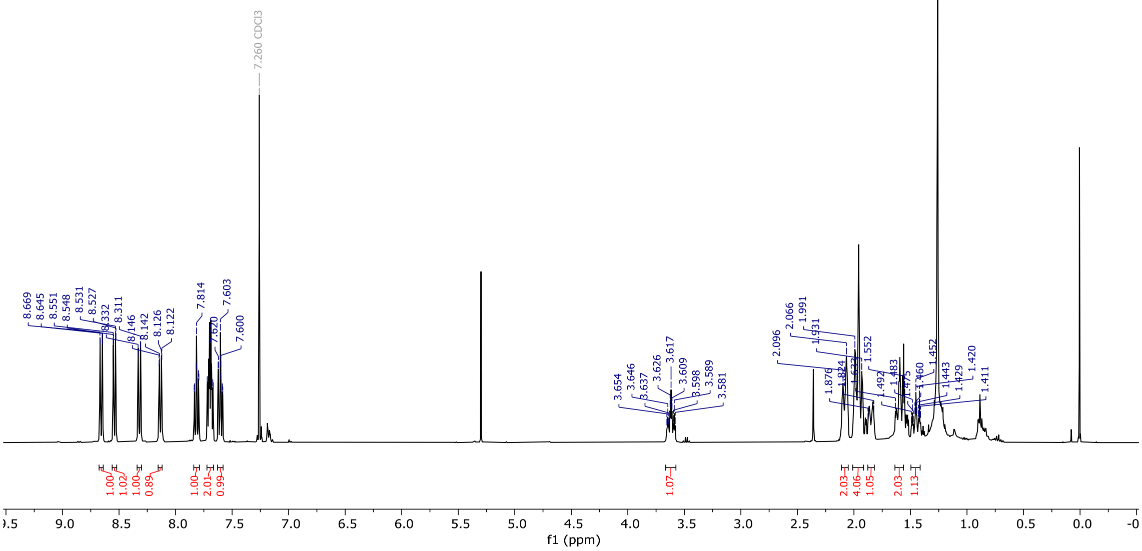

13C NMR (101 MHz, CDCl3)

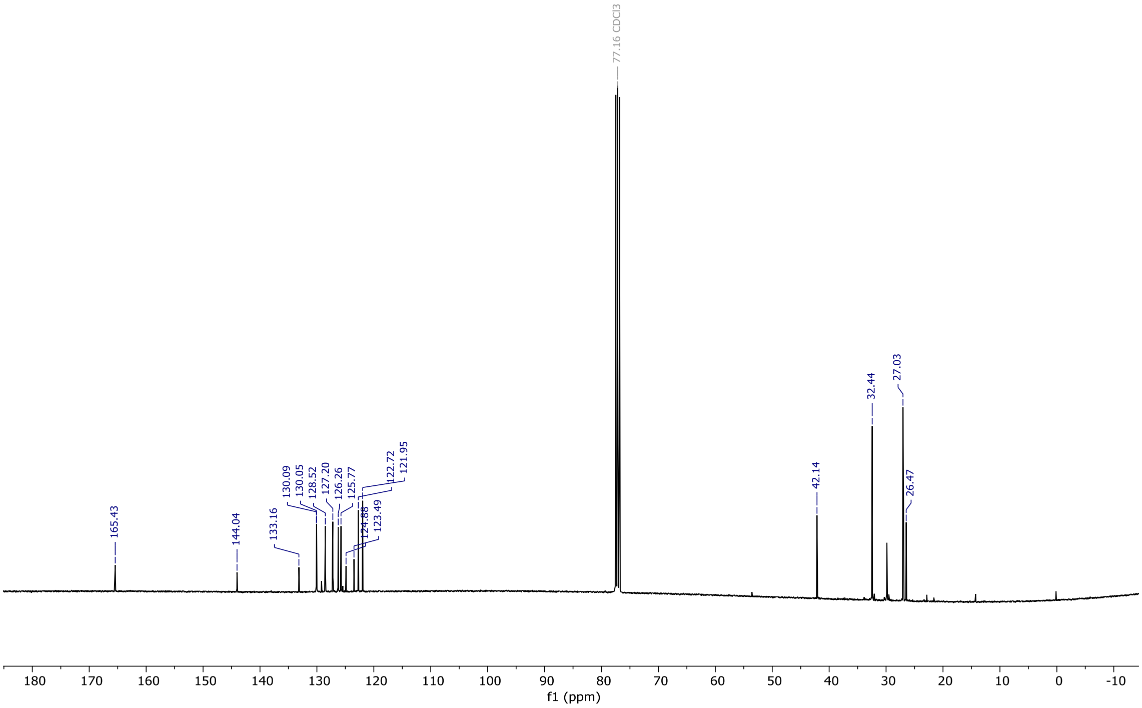

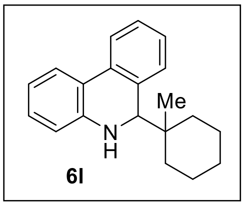

1H NMR (400 MHz, CD3OD)

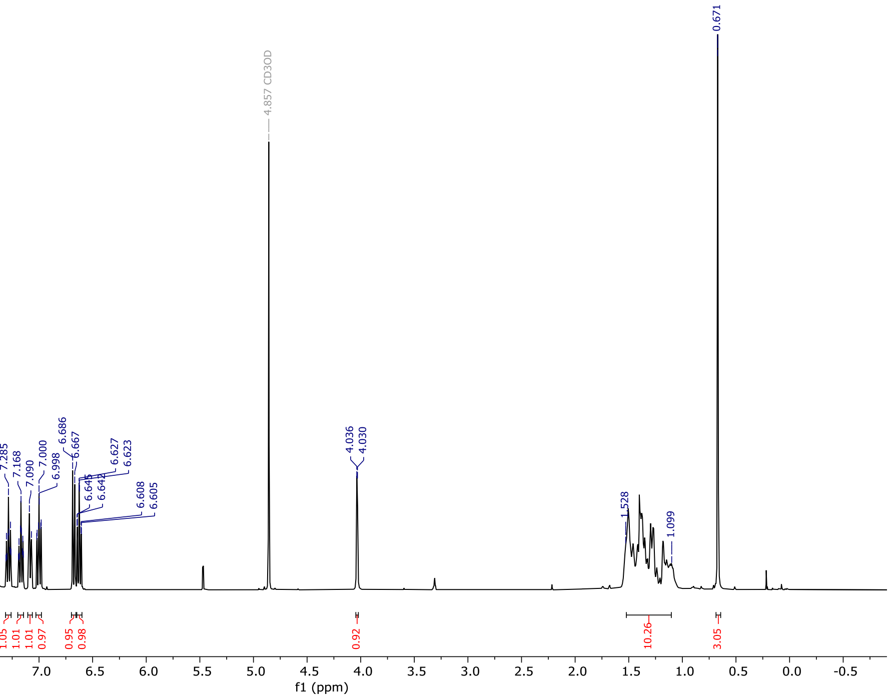

13C NMR (101 MHz, CD3OD

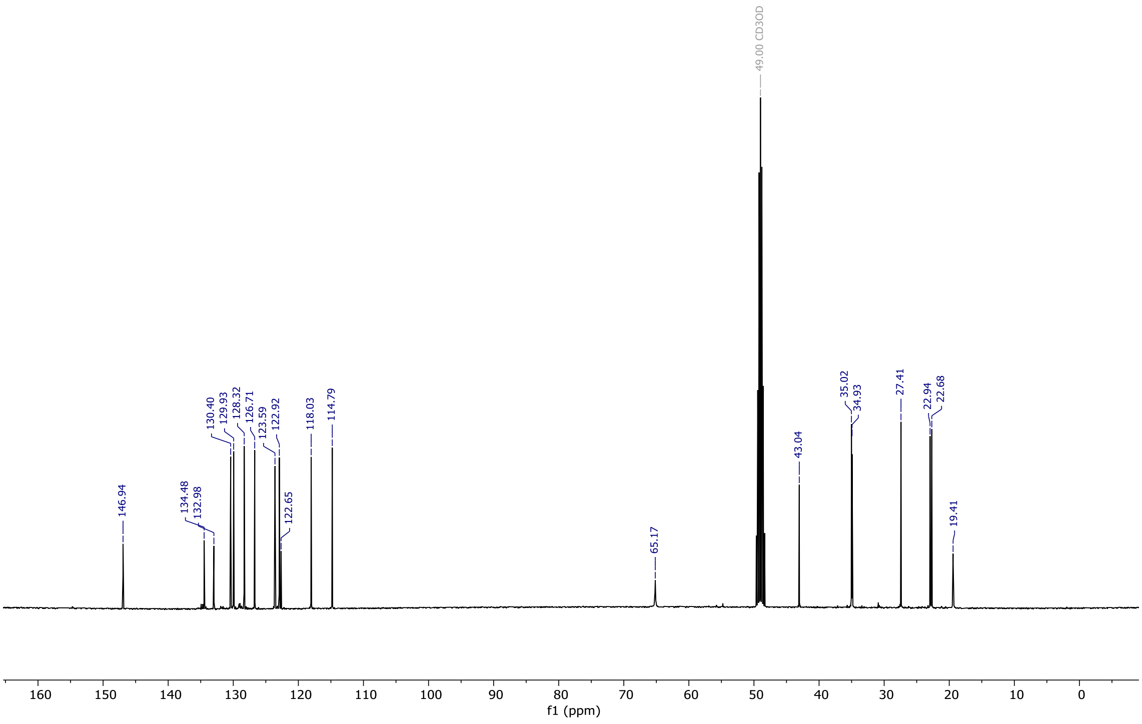

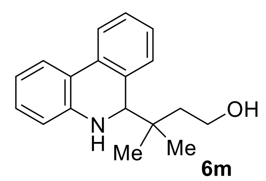

6m

1H NMR (400 MHz, CD3CN)

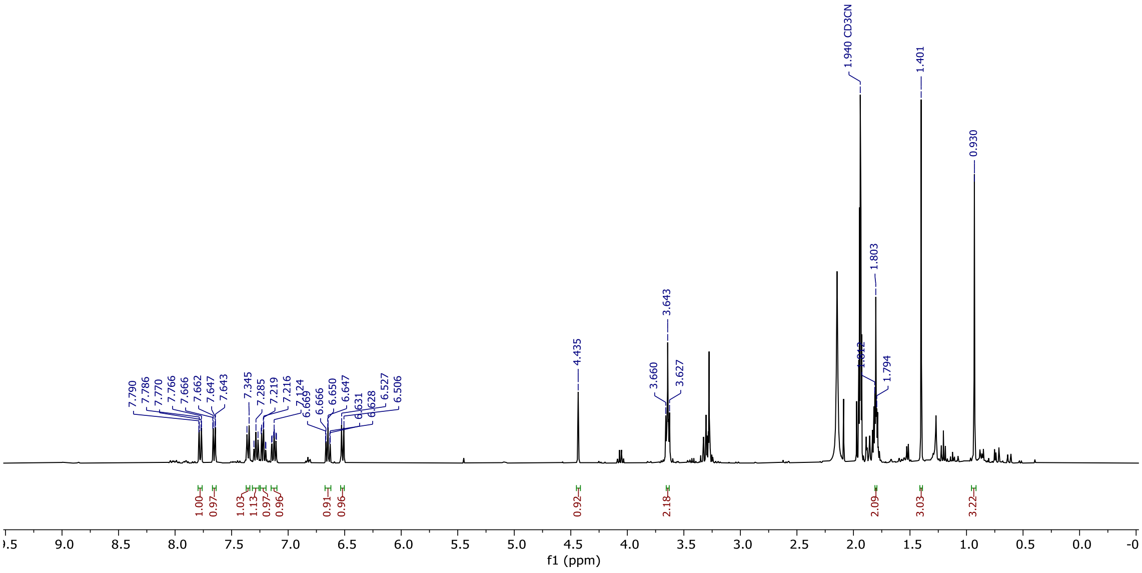

13C NMR (101 MHz, CD3CN)

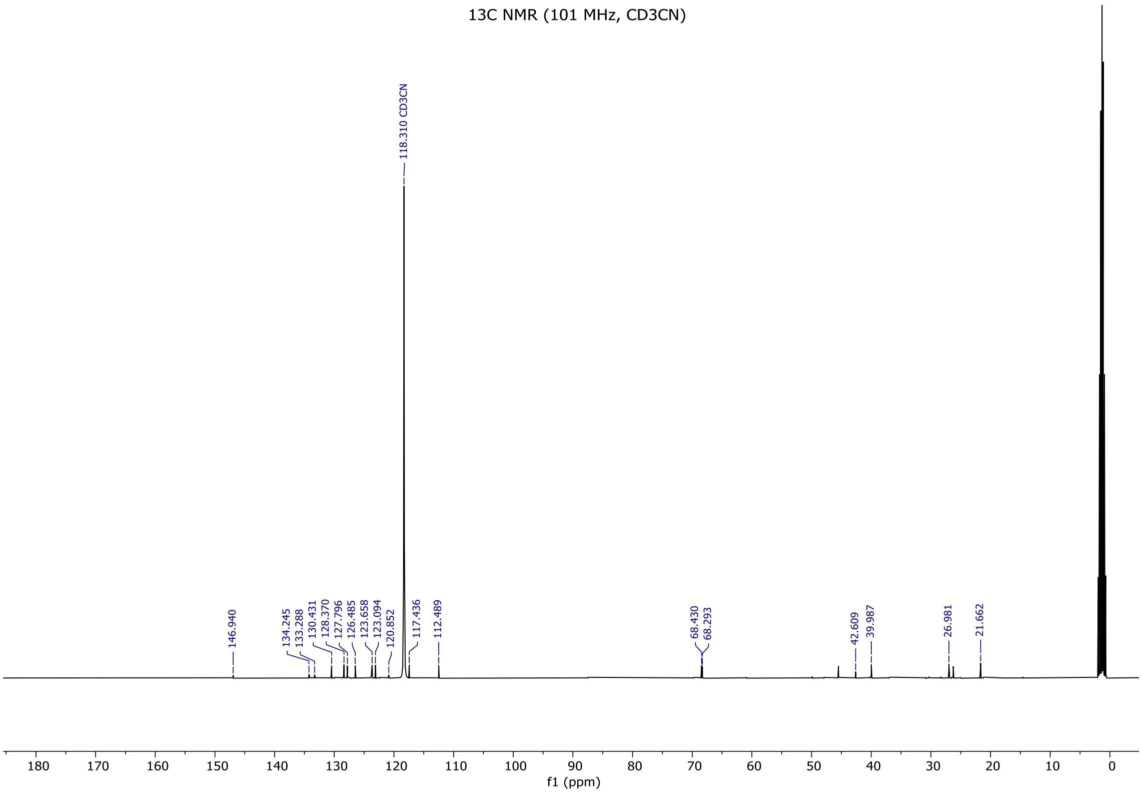

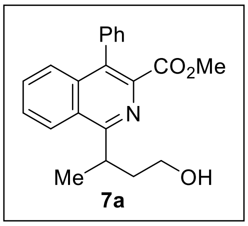

1H NMR (400 MHz, CDCl3)

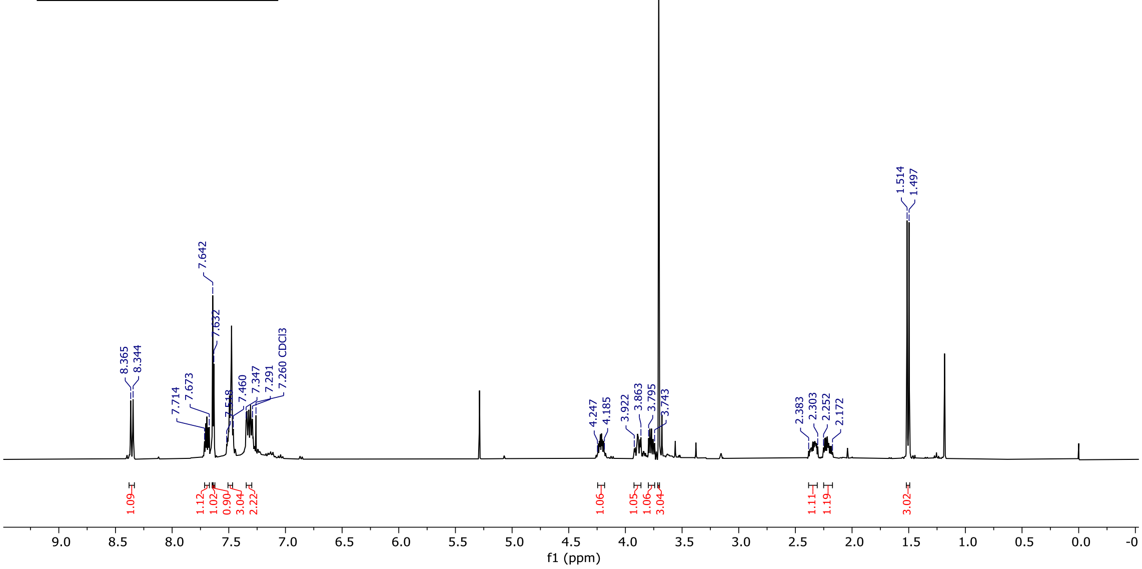

13C NMR (101 MHz, CDCl3)

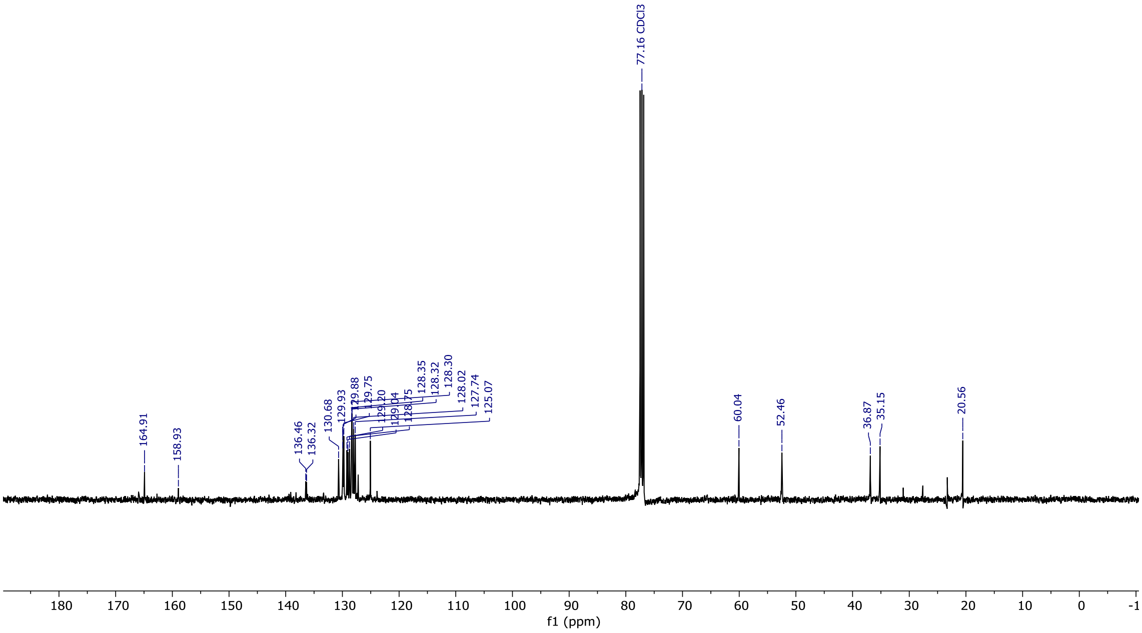

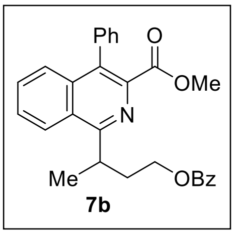

1H NMR (400 MHz, CDCl3)

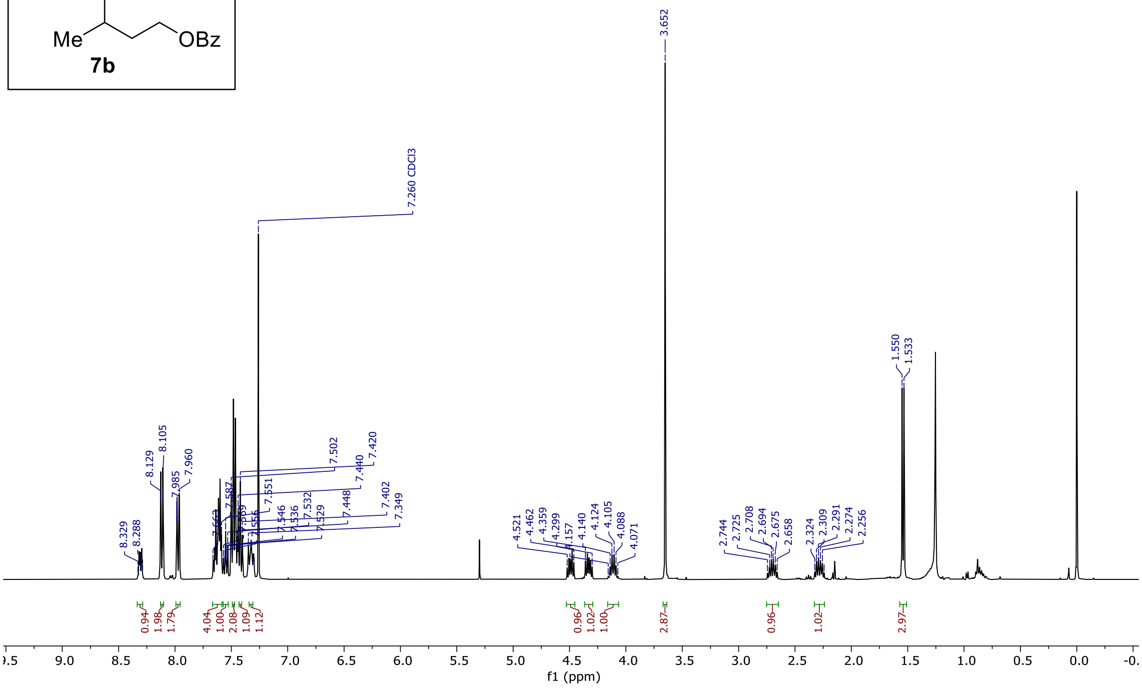

13C NMR (101 MHz, CDCl3)

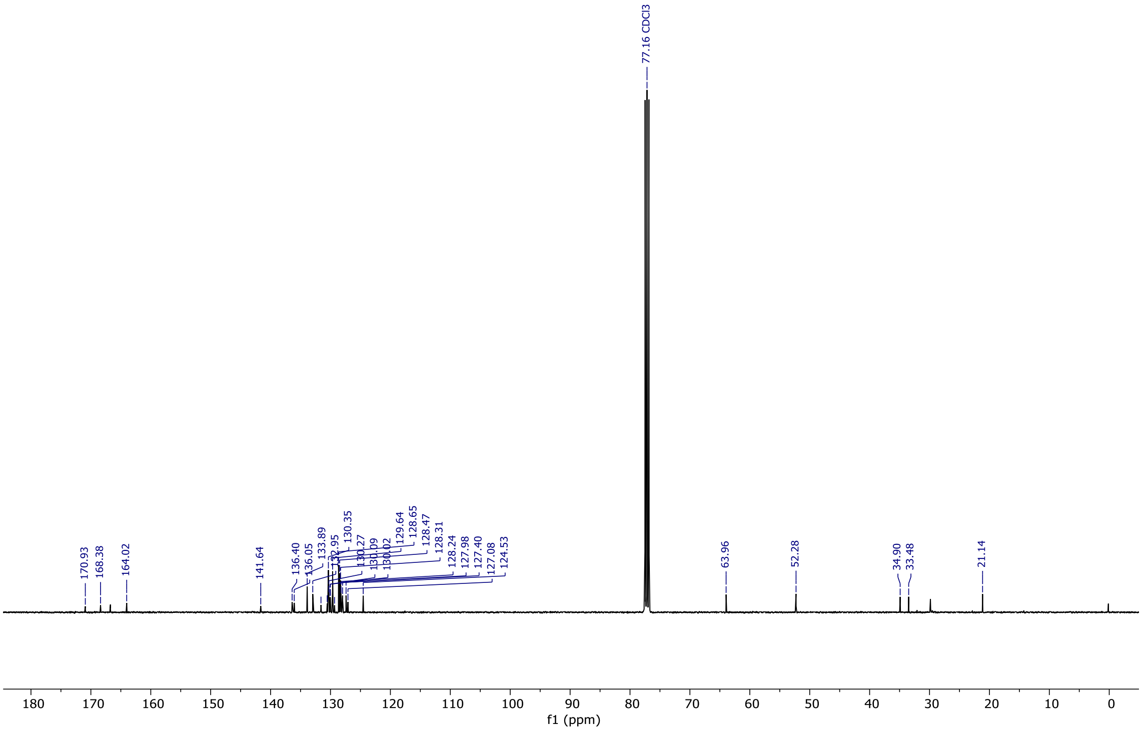

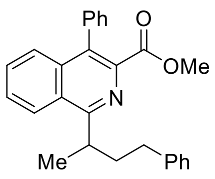

7c

1H NMR (400 MHz, CDCl3)

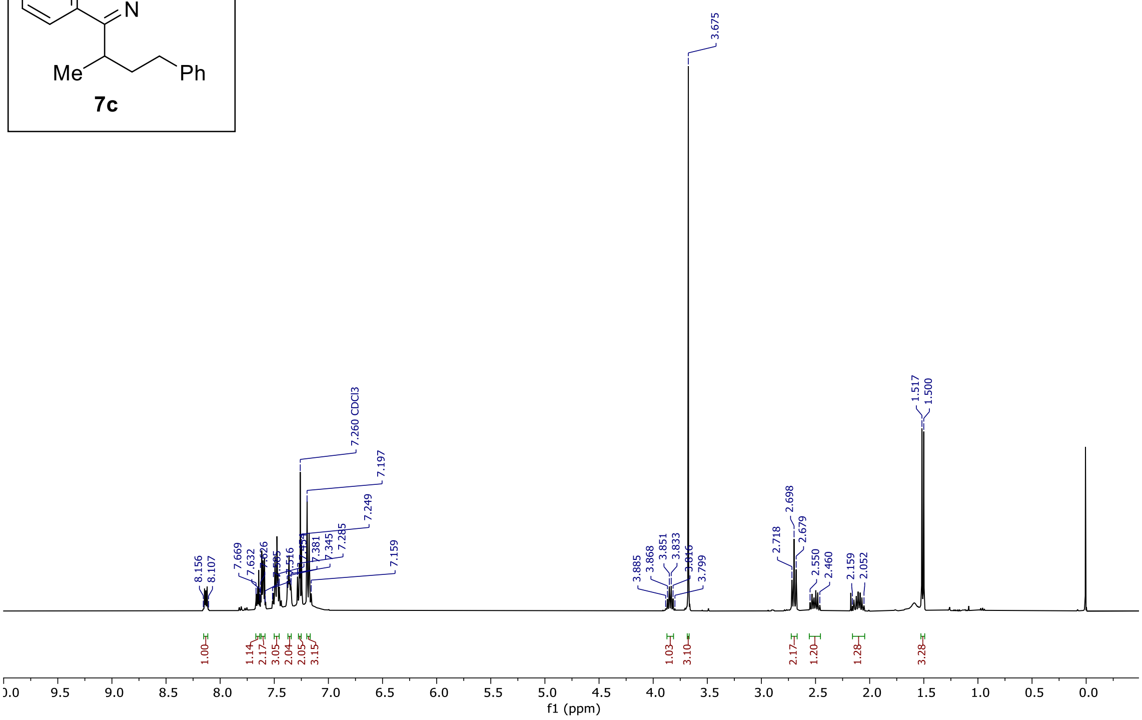

13C NMR (101 MHz, CDCl3)

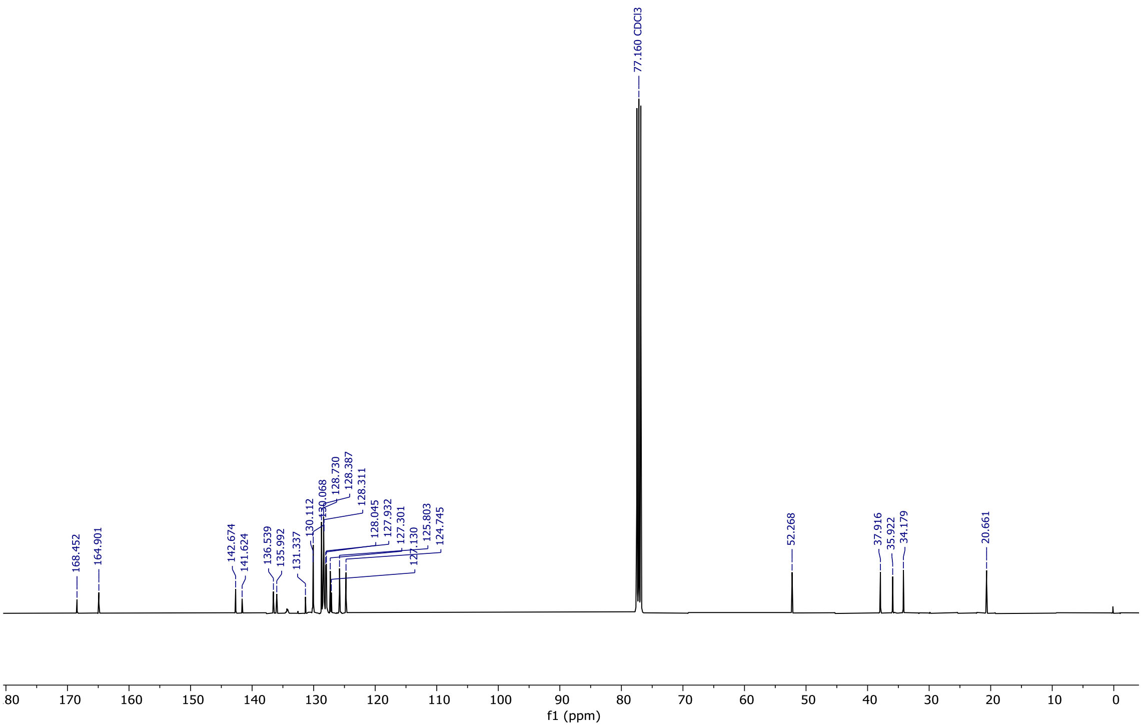

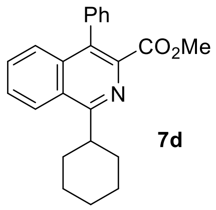

7d

1H NMR (500 MHz, CD3OD)

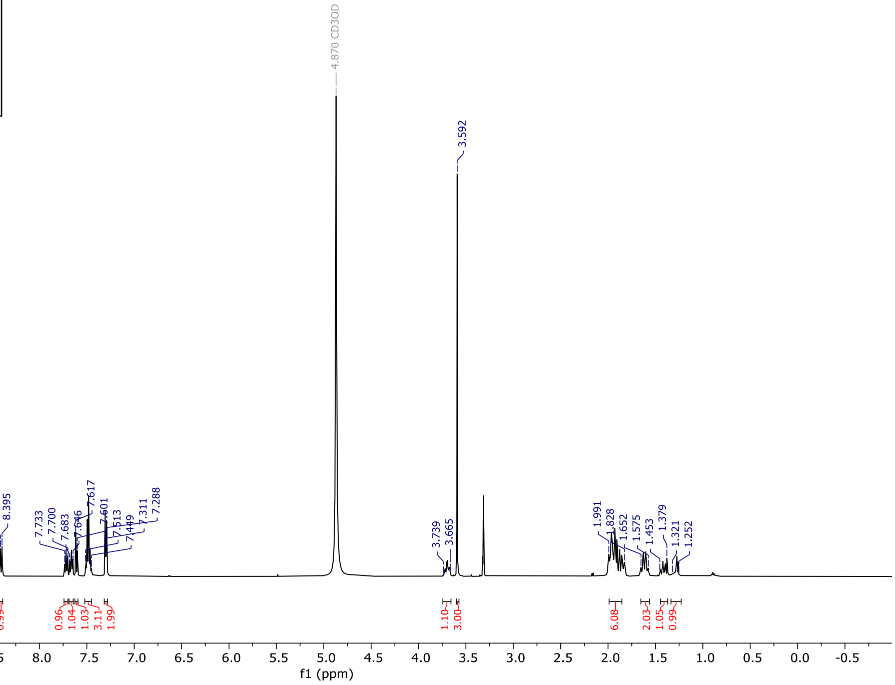

13C NMR (126 MHz, CD3OD)

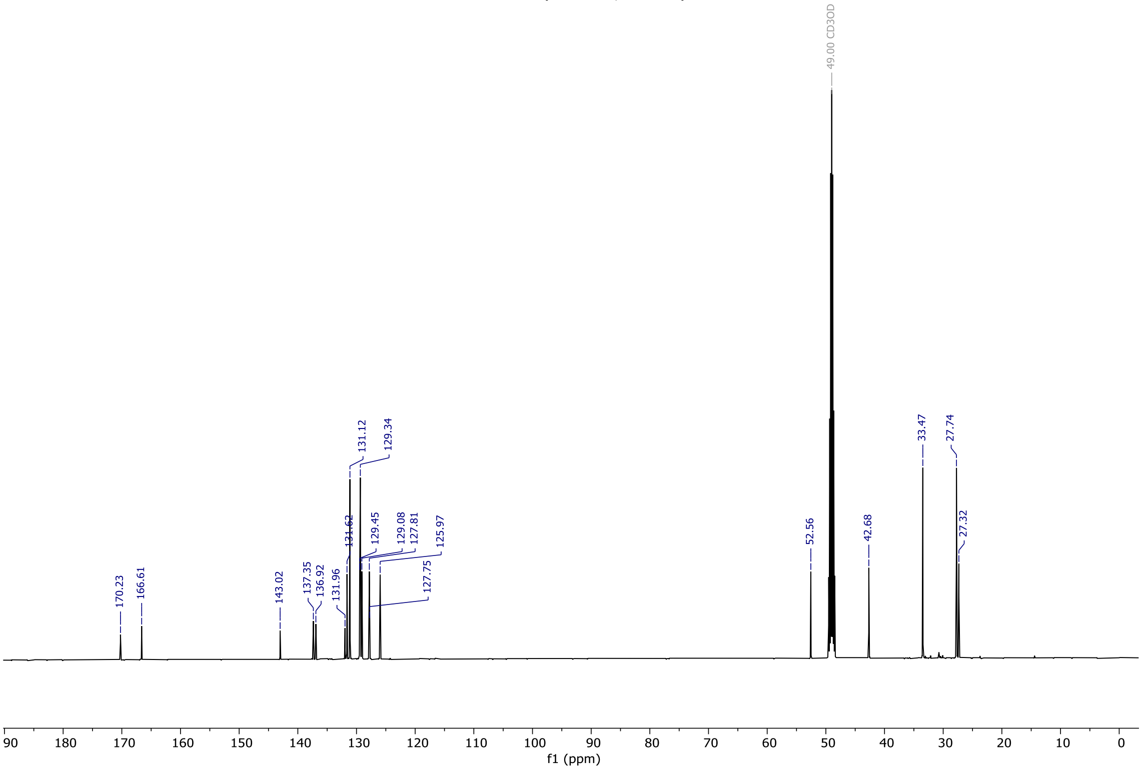

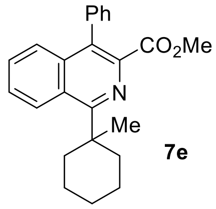

7e

1H NMR (400 MHz, CD2Cl2)

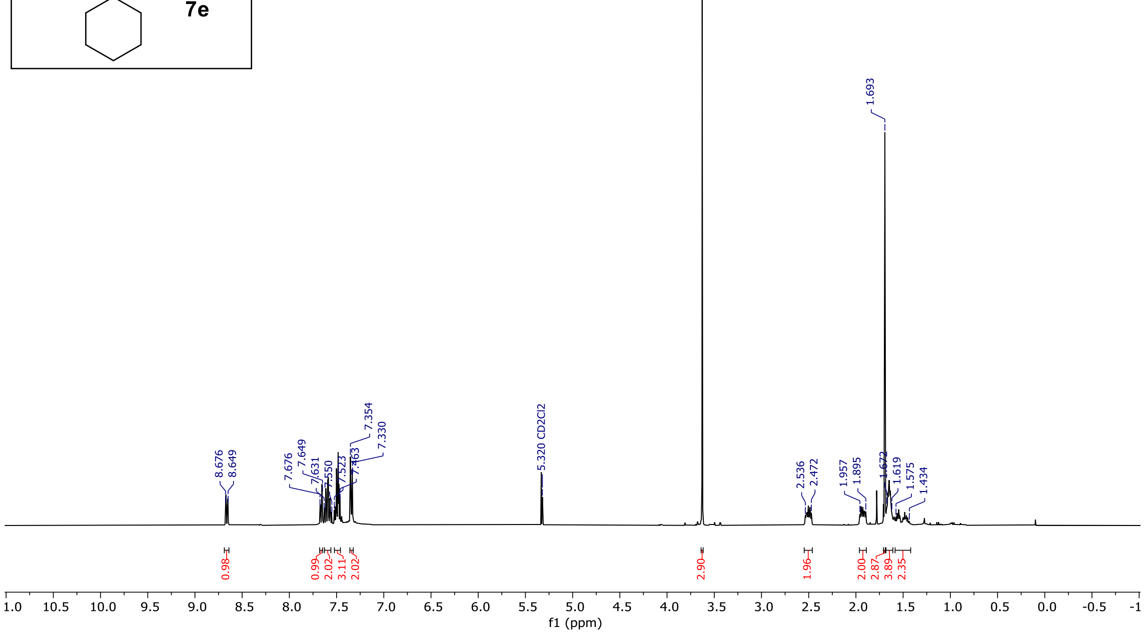

13C NMR (101 MHz, CD2Cl2)

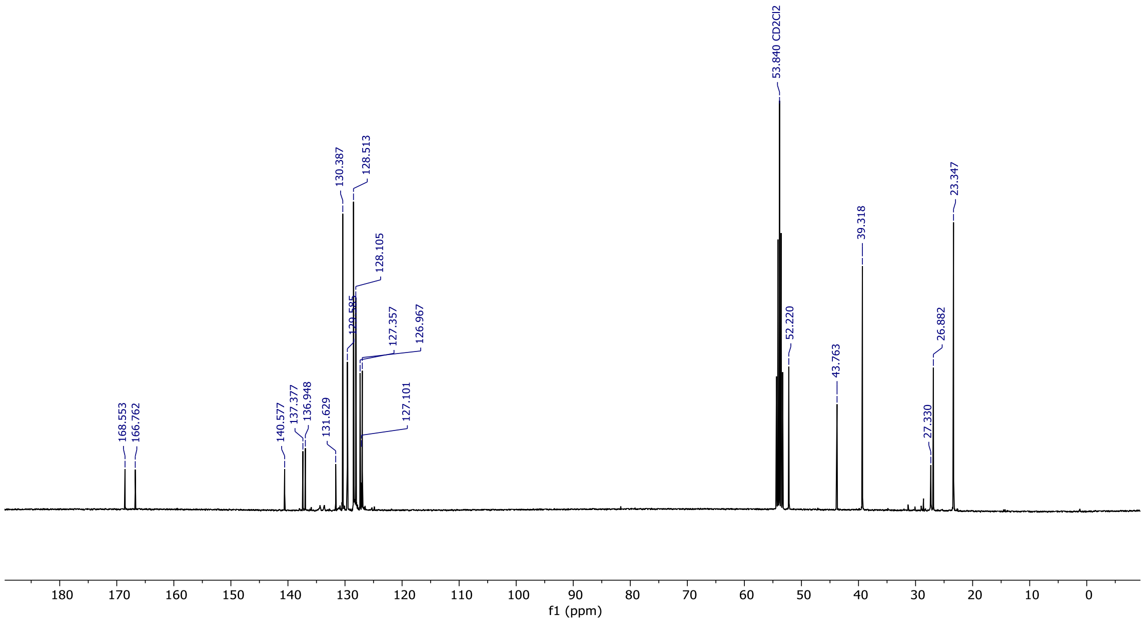

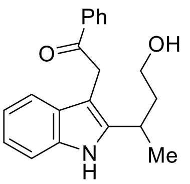

8a

1H NMR (400 MHz, CDCl3)

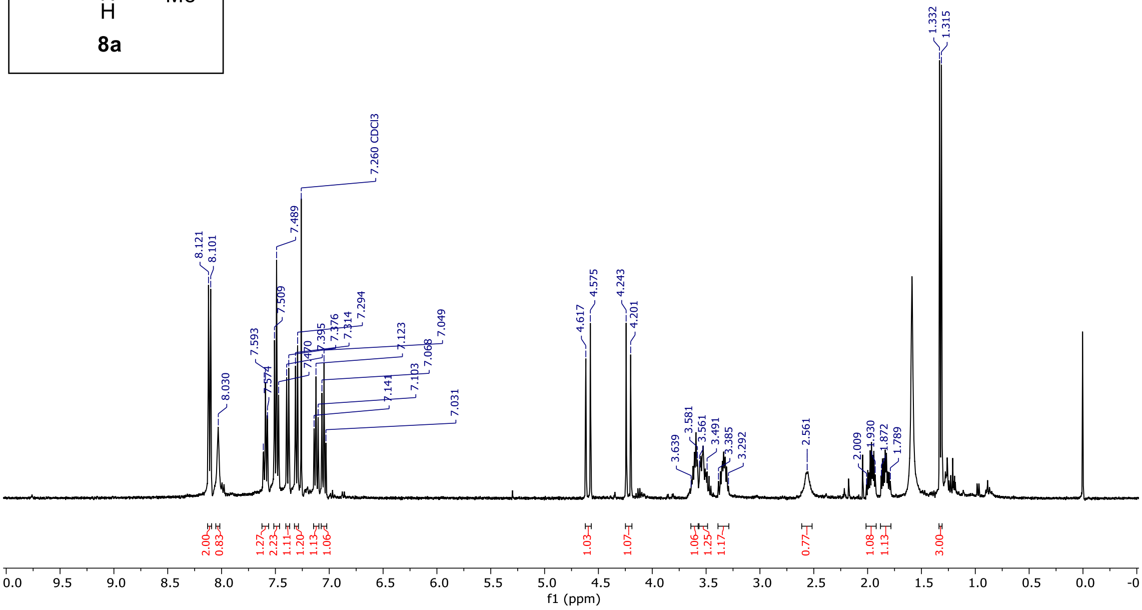

13C NMR (101 MHz, CDCl3)

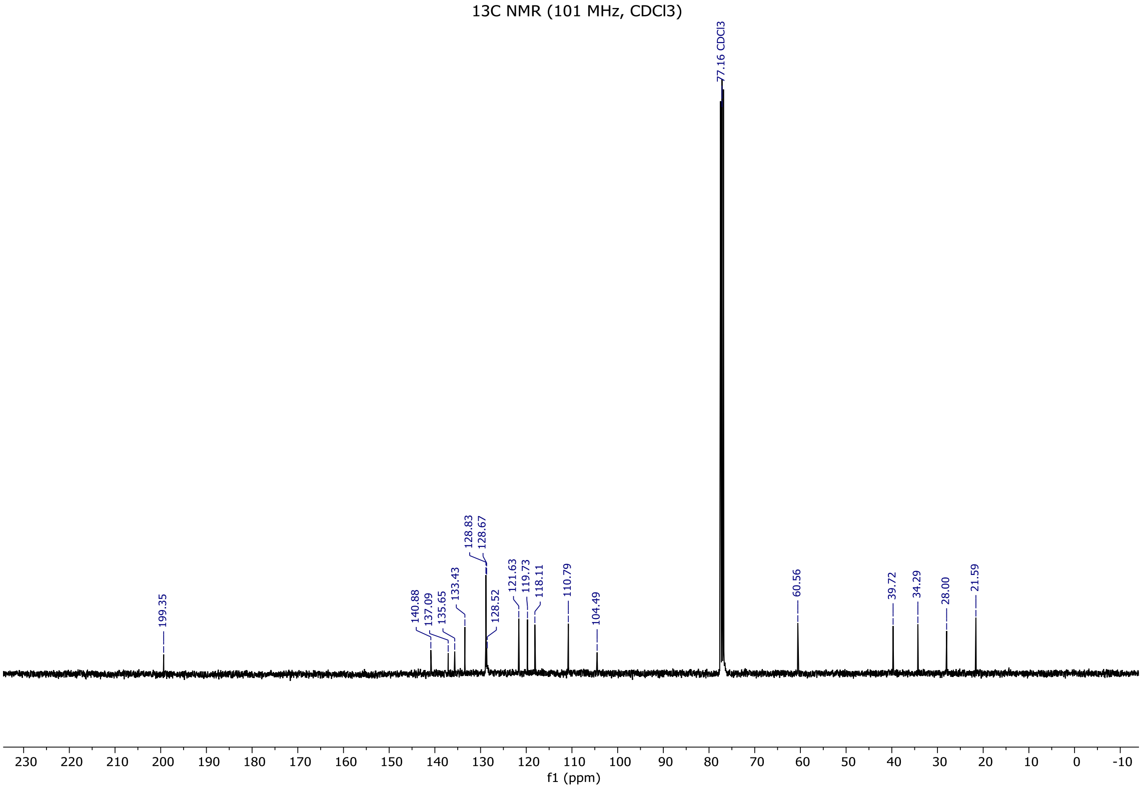

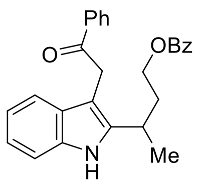

8b

1H NMR (400 MHz, CDCl3)

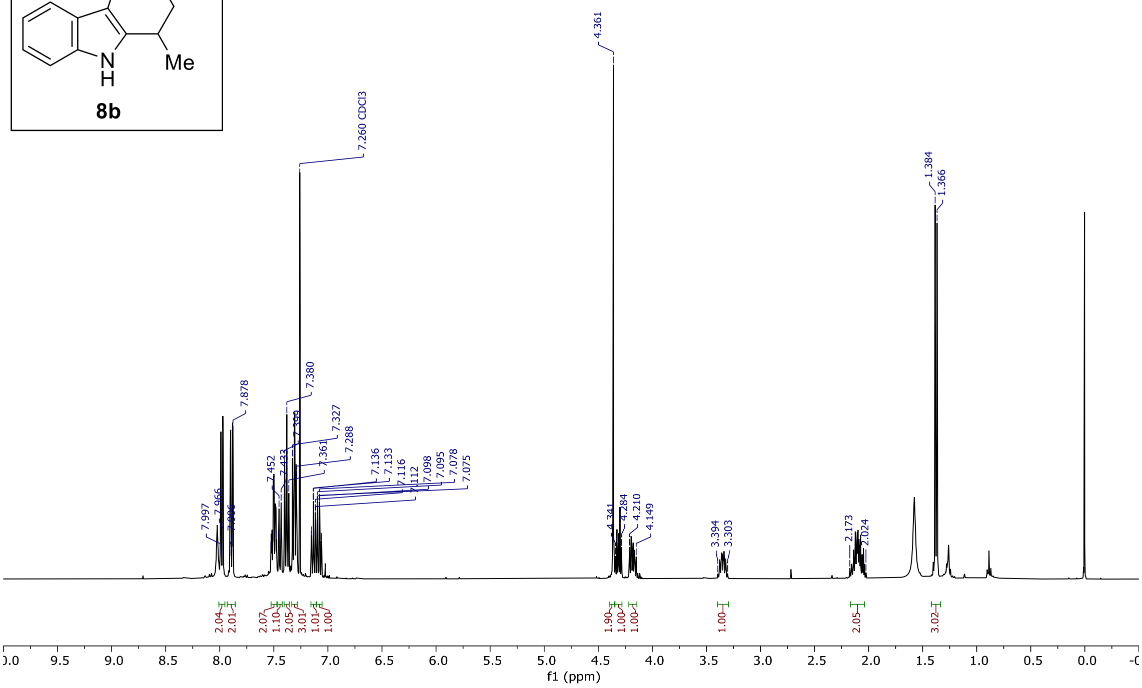

13C NMR (101 MHz, CDCl3)

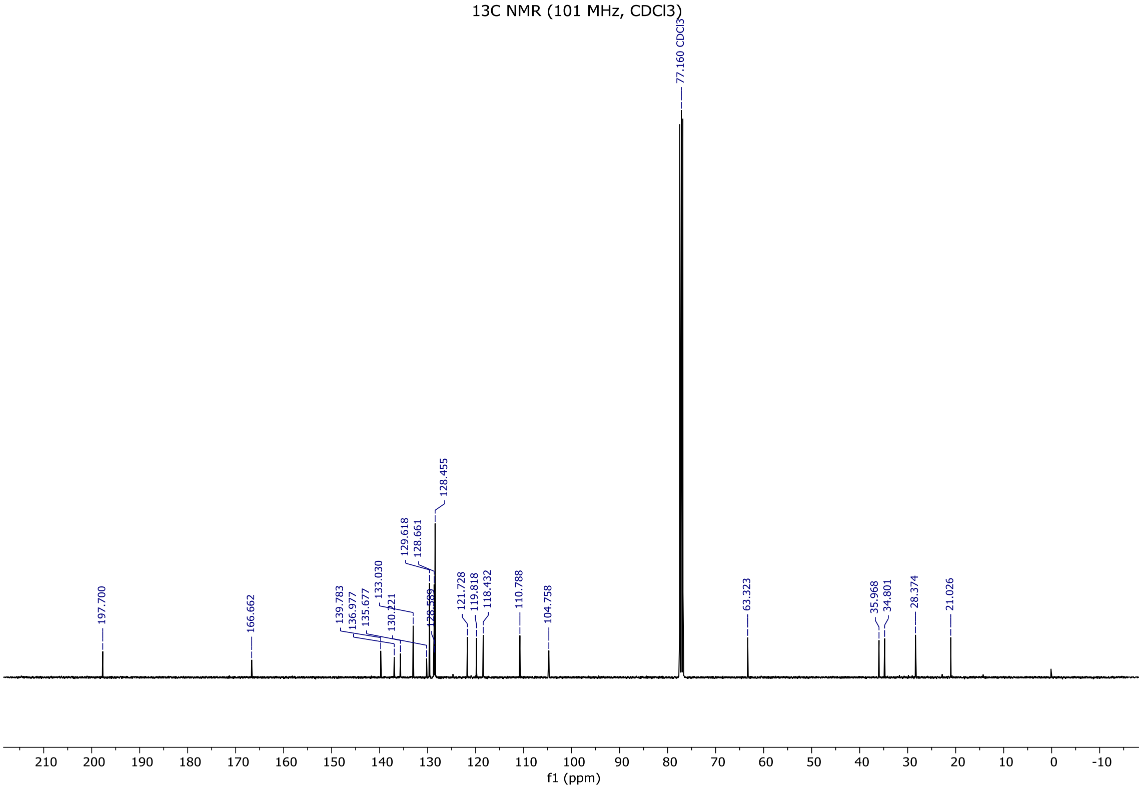

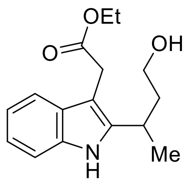

9a

1H NMR (400 MHz, CDCl3)

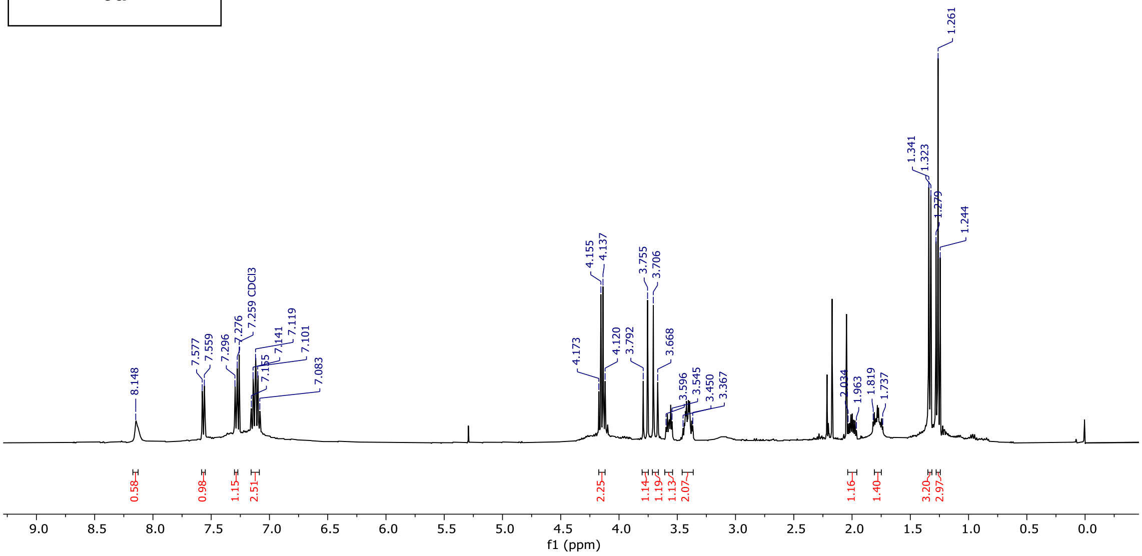

13C NMR (400 MHz, CDCl3)

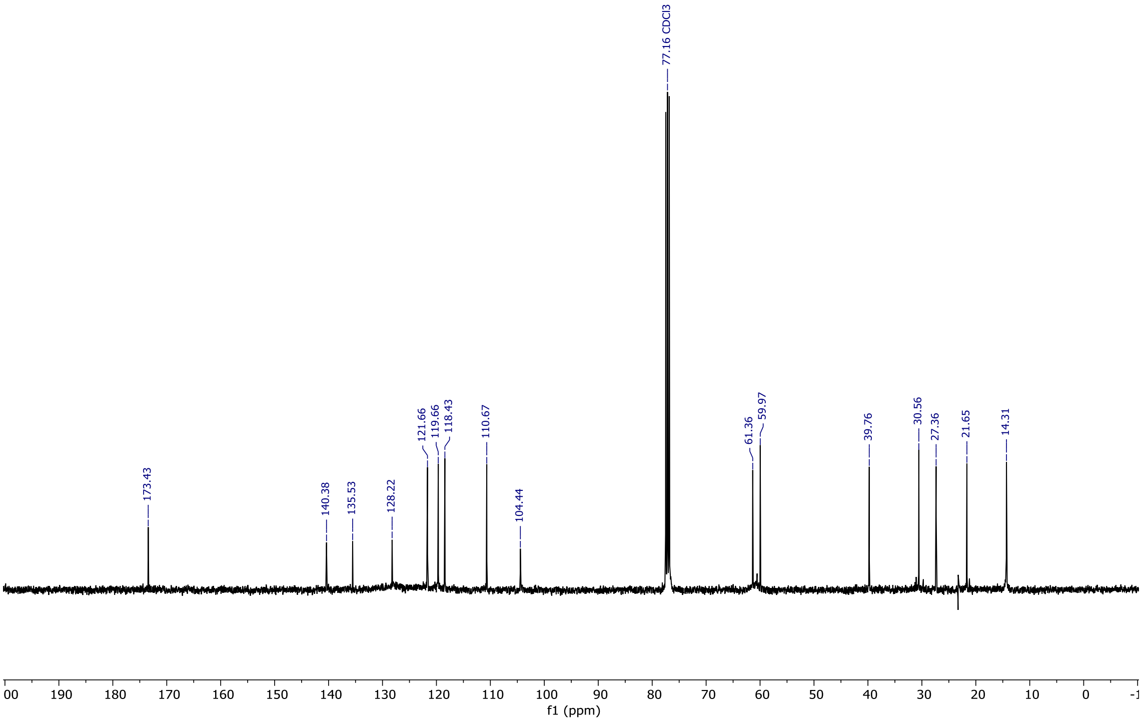

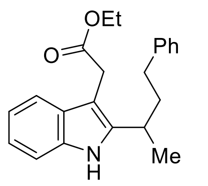

9b

1H NMR (400 MHz, CDCl3)

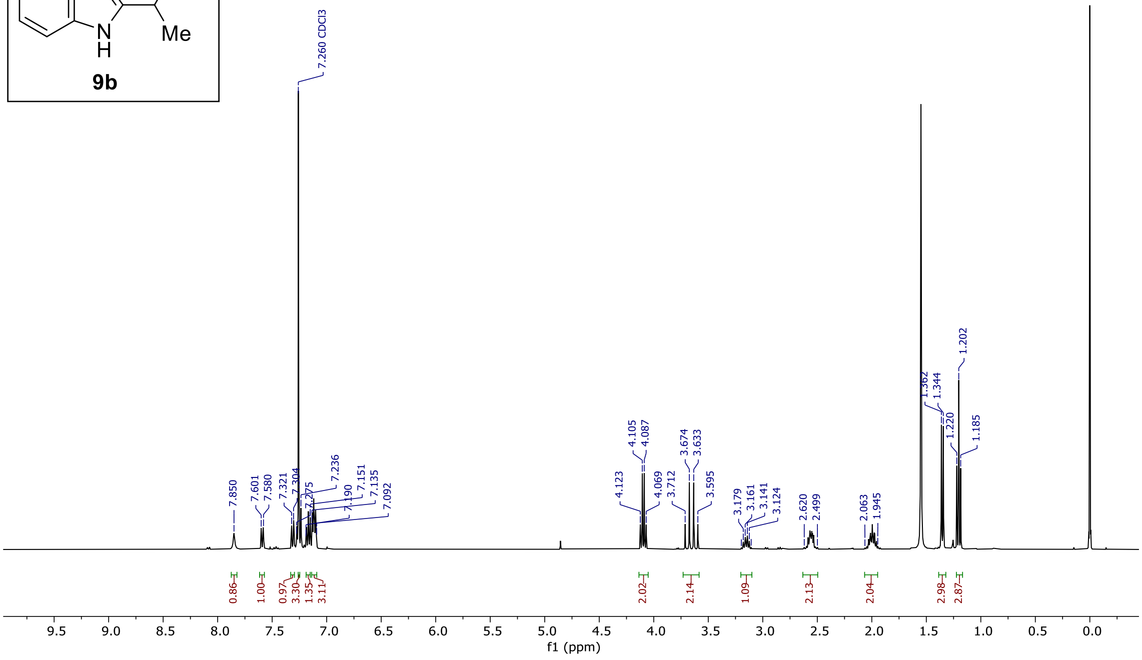

13C NMR (101 MHz, CDCl3)

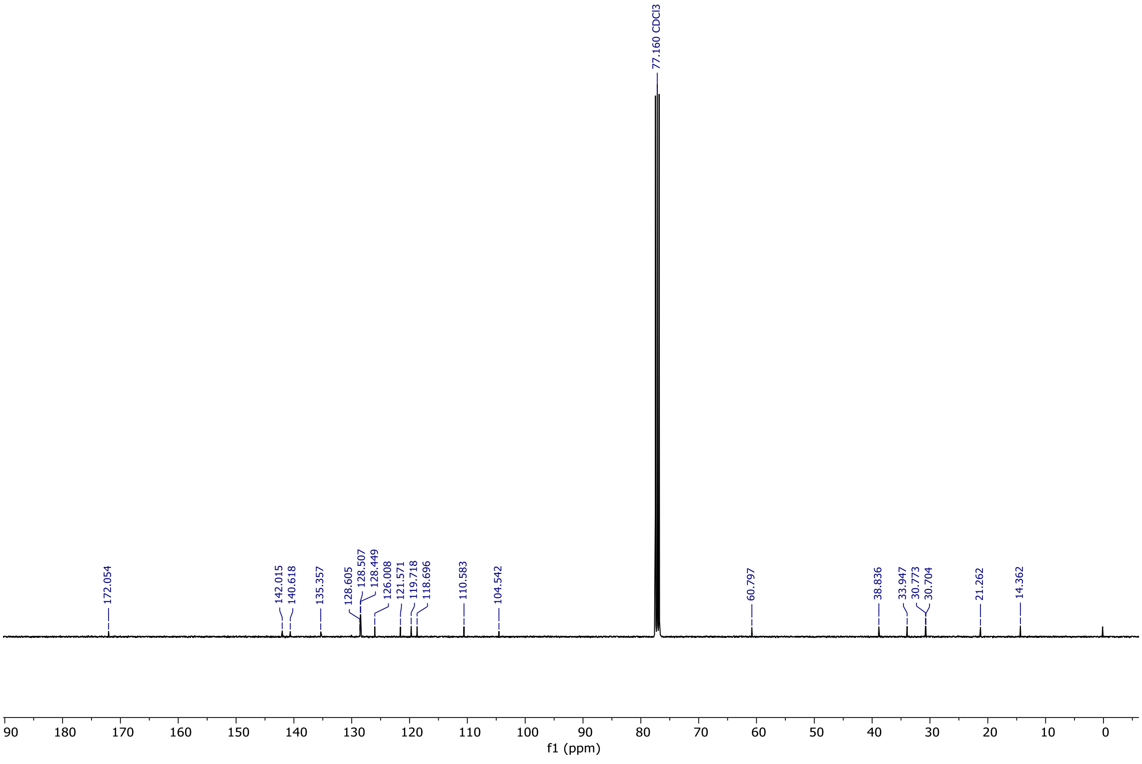

Copies of ^{19}F NMR spectra

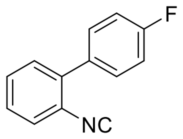

1c

19F NMR (376 MHz, CDCl3)

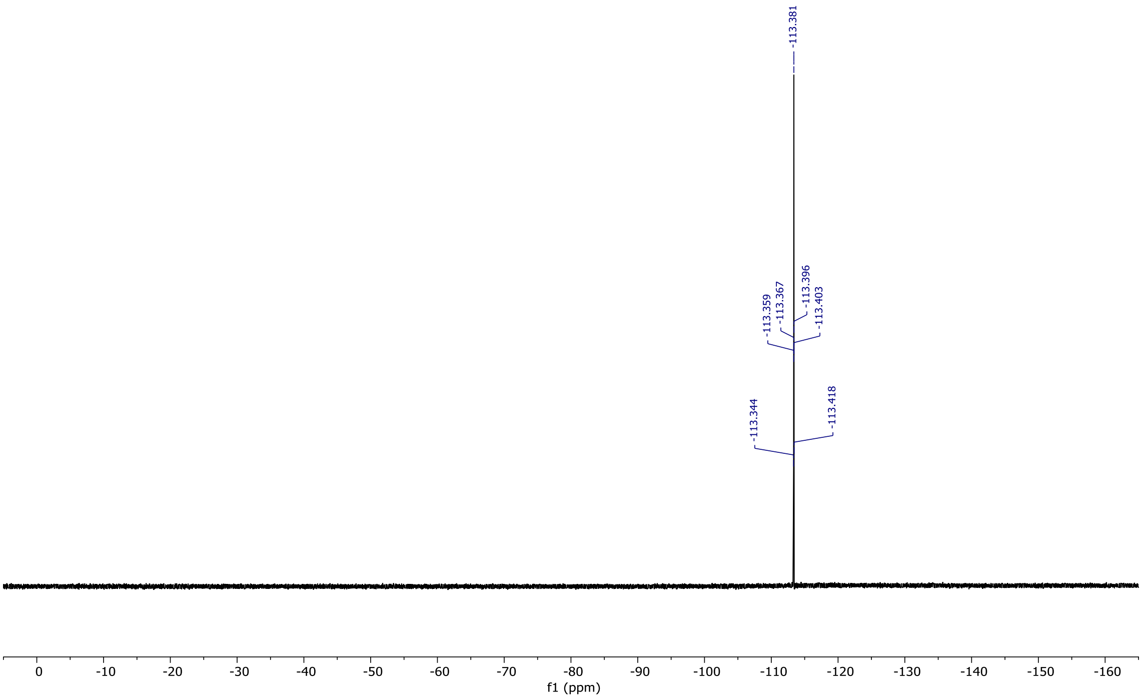

19F NMR (376 MHz, CDCl3)

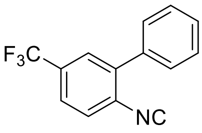

1e

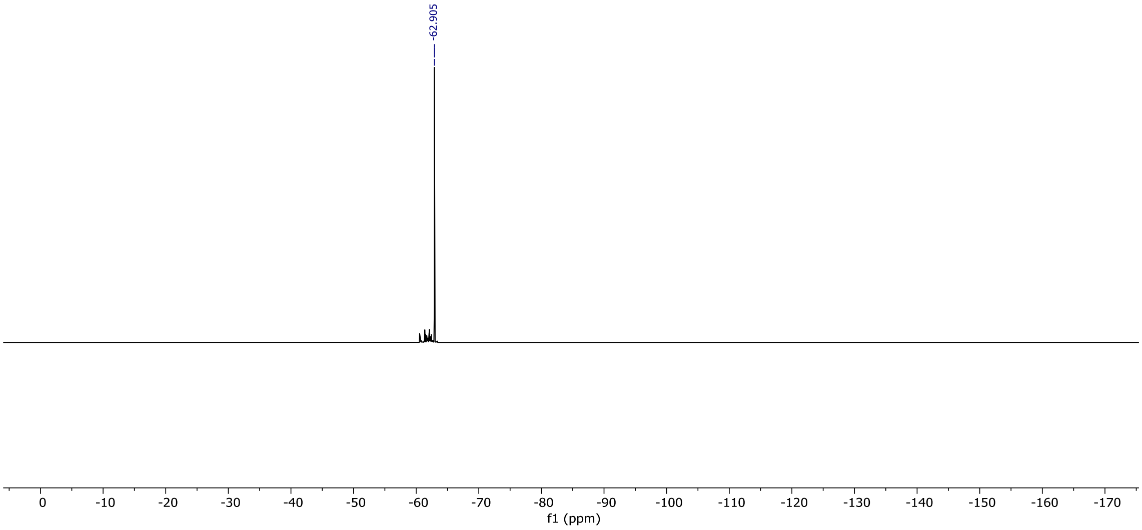

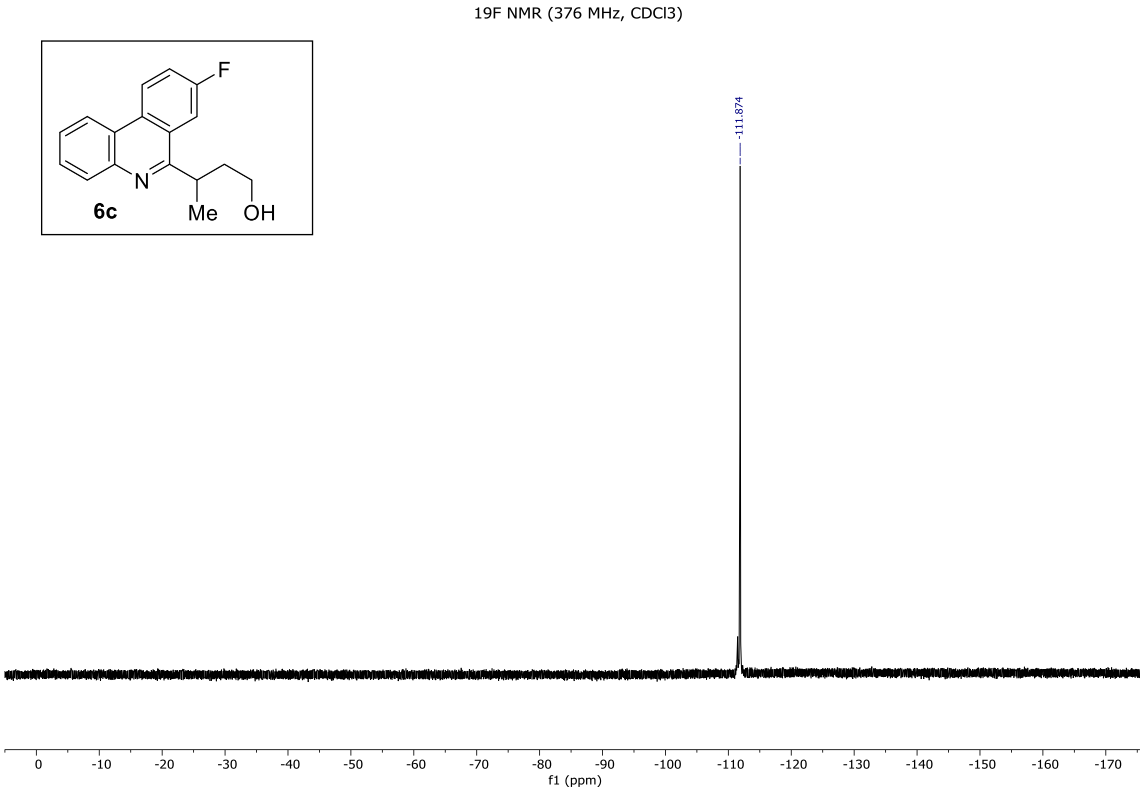
19F NMR (376 MHz, CDCl3)
F
N
6c
Me
OH
-111.874
0 -10 -20 -30 -40 -50 -60 -70 -80 -90 -100 -110 -120 -130 -140 -150 -160 -170
f1 (ppm)

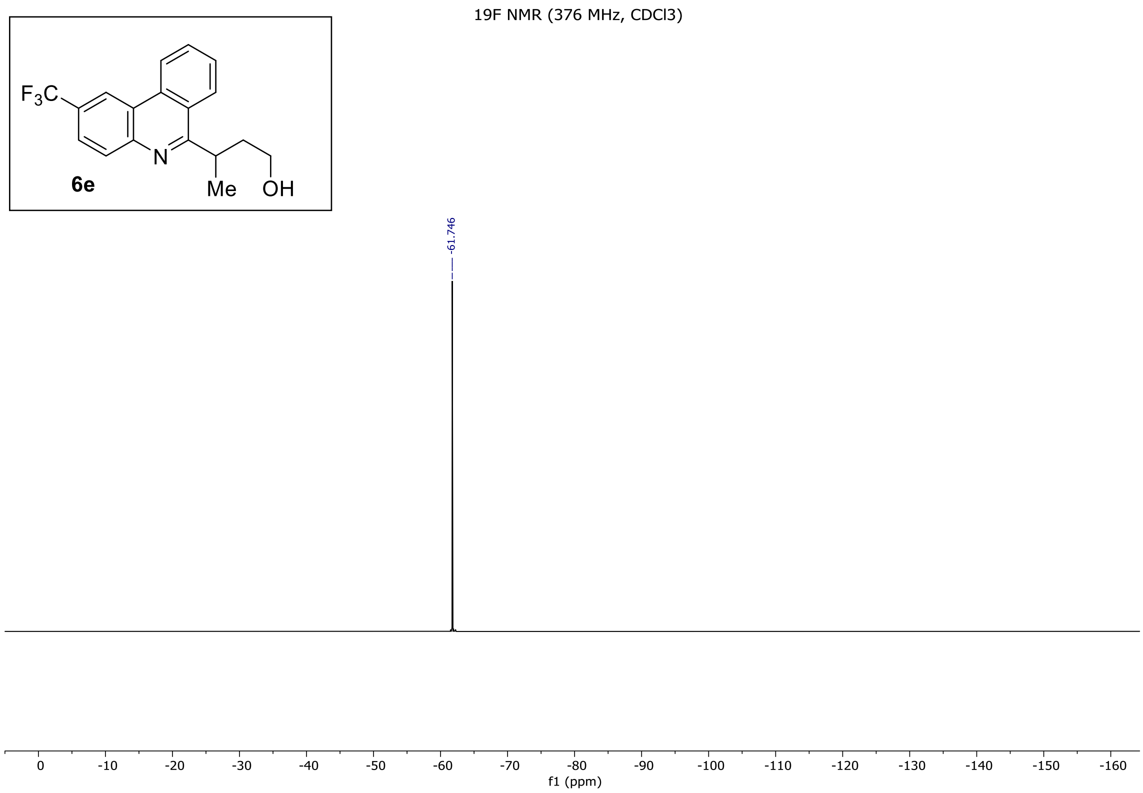
19F NMR (376 MHz, CDCl3)
F3C
N
6e
Me
OH
-61.746
0 -10 -20 -30 -40 -50 -60 -70 -80 -90 -100 -110 -120 -130 -140 -150 -160
f1 (ppm)

References

[1] Rong, J.; Deng, L.; Tan, P.; Ni, C.; Gu, Y.; Hu, J. Radical Fluoroalkylation of Isocyanides with Fluorinated Sulfones by Visible-Light Photoredox Catalysis. *Angew. Chem. Int. Ed.* **2016**, *55*, 2743−2747.

[2] Hu. Z.; Yuan, H.; Men, Y.; Liu, Q.; Zhang, J.; Xu, X. Cross-Cycloaddition of Two Different Isocyanides: Chemoselective Heterodimerization and [3+2]-Cyclization of 1,4-Diazabutatriene. *Angew. Chem. Int. Ed.* **2016**, *55*, 7077-7080.

[3] Compound **SI-6** was prepared by Horner-Wadsworth-Emmons homologation of 2-nitrobenzaldehyde followed by the reduction of the nitro group in analogous manner to **SI-4**.

[4] Evoniuk, C.J.; Gomes, G.D.P.; Ly, M.; White, F.D.; Alabugin, I.V. Coupling Radical Homoallylic Expansions with C–C Fragmentations for the Synthesis of Heteroaromatics: Quinolines from Reactions of o-Alkenylarylisonitriles with Aryl, Alkyl, and Perfluoroalkyl Radicals. *J. Org. Chem.* **2017**, *82*, 4265-4278.

[5] Wu, X.; Gannett, C.N.; Liu, J.; Zeng, R.; Novaes, L.F.T.; Wang, H.; Abruna, H.D.; Lin, S. Intercepting Hydrogen Evolution with Hydrogen-Atom Transfer: Electron-Initiated Hydrofunctionalization of Alkenes. *J.Am.Chem.Soc.* **2022**, *144*, 17783−17791.

[6] Mao, C.L.; Zhao, S.; Zang, Z.L.; Xiao, L.; Zhou, C.H.; He, Y.; Cai G.C. Pd-Catalyzed Remote Site-Selective and Stereoselective C(Alkenyl)−H Alkenylation of Unactivated Cycloalkenes. *J.Org.Chem.* **2020**, *85*, 774−787.

[7] a) Leifert, D.; Daniliuc, C.G.; Studer, A. 6-Aroylated Phenanthridines via Base Promoted Homolytic Aromatic Substitution (BHAS). *Org.Lett.*, **2013**, *15*, 6286. b) Patil, P.; Ahmadian-Moghaddam, M.; Dömling, A. Isocyanide 2.0. *Green Chem.* **2020**, *22*, 6902-6911.

[8] Wang, H.; Yu, Y.; Hong, X.; Xu, B. Mn(II)/O_2-promoted oxidative annulation of vinyl isocyanides with boronic acids: synthesis of multi-substituted isoquinolines. *Chem. Commun.* **2014**, *50*, 13485-13488.

[9] Linsenmeier, A. M.; Williams, C.M.; Bräse, S. Synthesis of Phenanthridine Derivatives via Photolysis. *J. Org. Chem.* **2011**, *76*, 9127-9132.

[10] Qian, P.; Du, B.; Zhou, J.; Mei, H.; Han, J.; Pan, Y. DBU-promoted cyclization of vinyl isocyanides with ethers via the functionalization of a C(sp^3)–H bond for the synthesis of isoquinolines. *RSC Adv.*, **2015**, *5*, 64961-64965.

[11] Wang, P.; Zhao, J.Z.; Li, H.F.; Liang, X.M.; Zhang, Y.L.; Da, C.S. Acid-catalyzed highly diastereoselective and effective synthesis of 1,3-disubstituted tetrahydropyrano[3,4-b]indoles. *Tetrahedron Lett.* **2017**, *58*, 129-133.

[12] Vargas, D.A.; Tinoco, A.; Tyagi, V.; Fasan, R. Myoglobin-Catalyzed C−H Functionalization of Unprotected Indoles. *Angew. Chem. Int. Ed.* **2018**, *57*, 9911-9915.

[13] Sha, W.; Yu, J.T.; Jiang, Y.; Yang, H.; Cheng, J. The benzoyl peroxide-promoted functionalization of simple alkanes with 2-aryl phenyl isonitrile. *Chem. Commun.* **2014**, *50*, 9179-9181.

[14] Mao, H., Gao, M., Liu, B., Xu, B. Manganese(II)-catalyzed modular synthesis of isoquinolines from vinyl isocyanides and hydrazines. *Org. Chem. Front.*, **2016**, *3*, 516-521.
