# Supplementary figures and images for: Isocyanides as Acceptor Groups in MHAT Reactions with Unactivated Alkenes
Source: Org Lett. 2023 Aug 30;25(35):6539–43. doi: 10.1021/acs.orglett.3c02358 (PMC10496133; doi:10.1021/acs.orglett.3c02358)

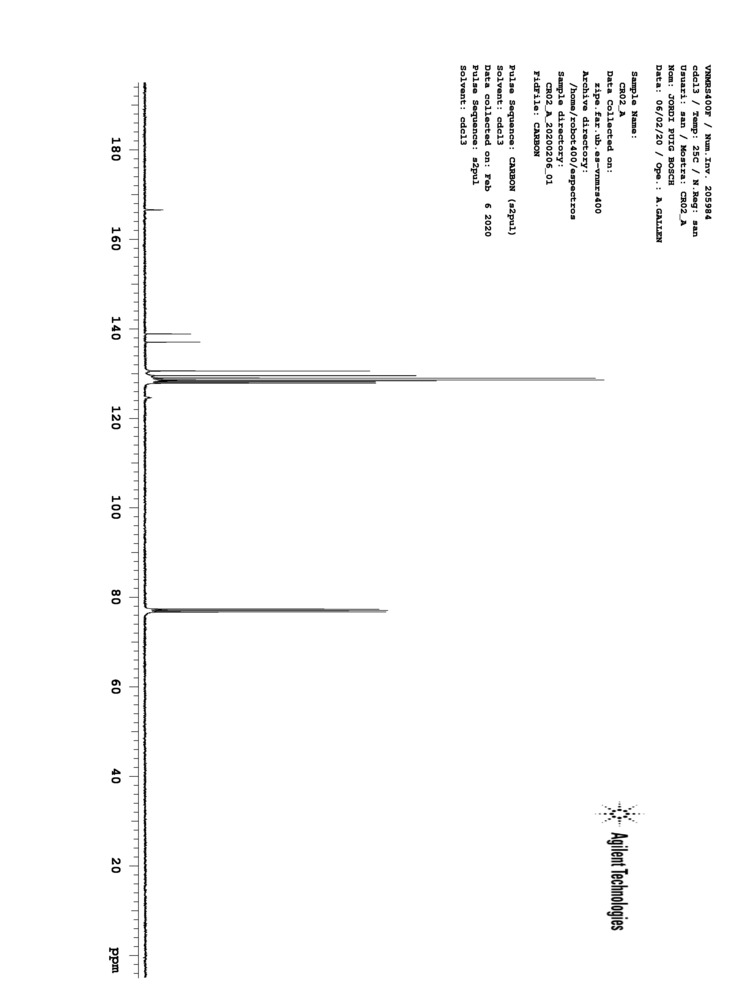

Supplement: Supplementary file 2 — ol3c02358_si_002.zip [file ol3c02358_si_002.zip › FID for publication/FID for publication/Compound 1a/Compound 1a-C13.fid/espectro.jpg]

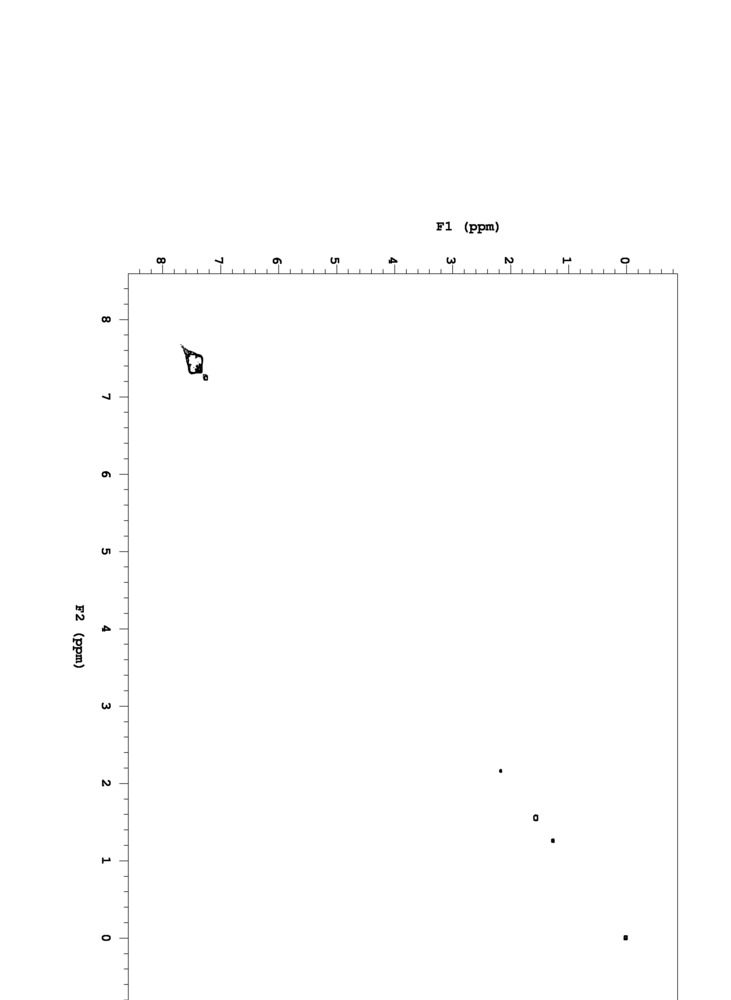

Supplement: Supplementary file 2 — ol3c02358_si_002.zip [file ol3c02358_si_002.zip › FID for publication/FID for publication/Compound 1a/Compound 1a-gCOSY.fid/espectro.jpg]

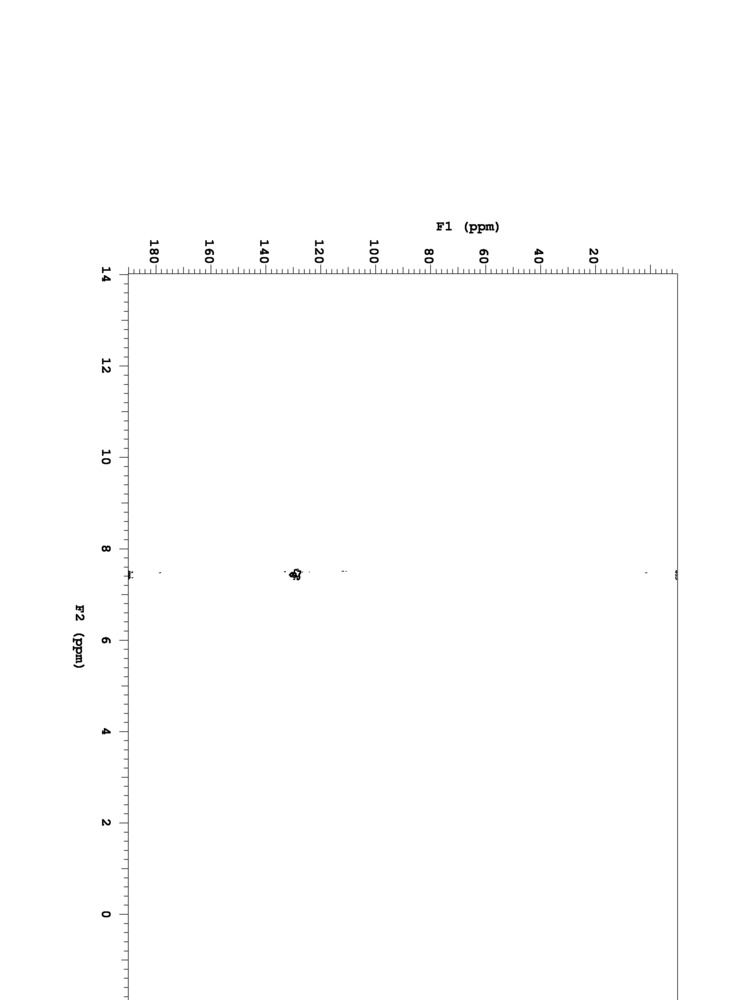

Supplement: Supplementary file 2 — ol3c02358_si_002.zip [file ol3c02358_si_002.zip › FID for publication/FID for publication/Compound 1a/Compound 1a-gHSQCAD.fid/espectro.jpg]

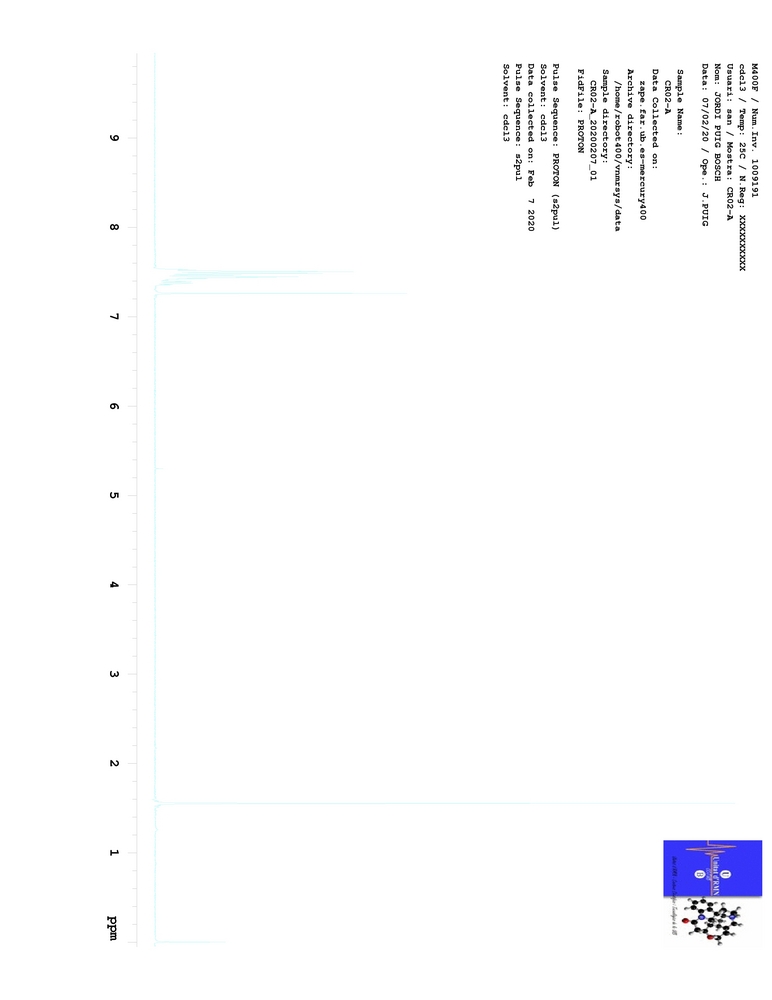

Supplement: Supplementary file 2 — ol3c02358_si_002.zip [file ol3c02358_si_002.zip › FID for publication/FID for publication/Compound 1a/Compound 1a-H1.fid/espectro.jpg]
