# Supplementary material for: Isocyanides as Acceptor Groups in MHAT Reactions with Unactivated Alkenes
Source: Org Lett. 2023 Aug 30;25(35):6539–43. doi: 10.1021/acs.orglett.3c02358 (PMC10496133; doi:10.1021/acs.orglett.3c02358)
Supplement: Supplementary file 2 — ol3c02358_si_002.zip [file ol3c02358_si_002.zip › FID for publication/FID for publication/Compound 7d/Compound 7d-COSY.fid/pdata/1/san-11086-2022_12_1.pdf]

Register 11086/2022
Operator AGUSTIN MARTINEZ

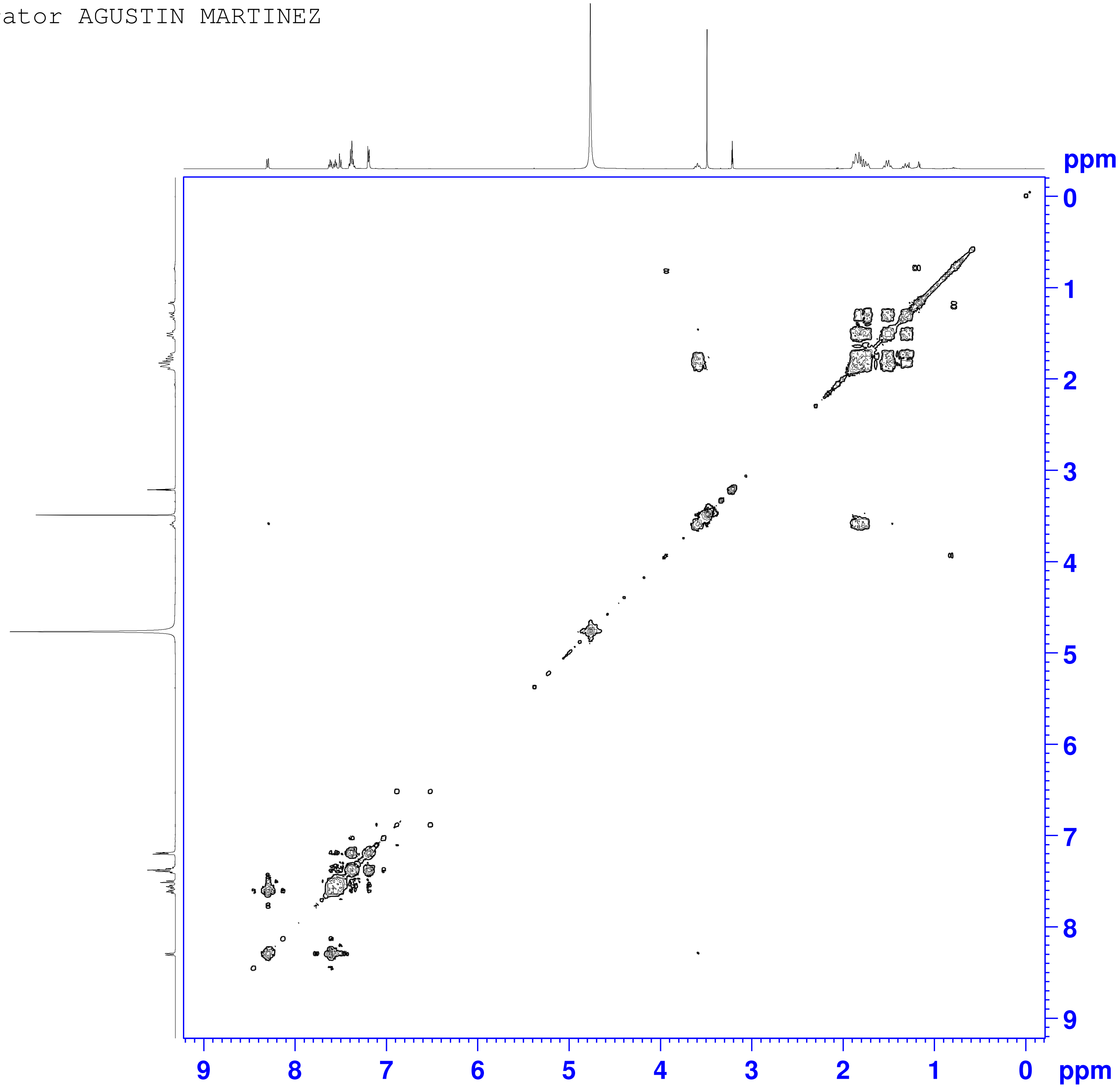


Current Data Parameters
NAME san-11086-2022
EXPNO 12
PROCNO 1

F2 - Acquisition Parameters
Date_ 20221013
Time 5.17 h
INSTRUM Avance Neo
PROBHD Z151574_0054 (
PULPROG cosygpppqf
TD 2048
SOLVENT MeOD
NS 4
DS 16
SWH 4716.981 Hz
FIDRES 4.606427 Hz
AQ 0.2170880 sec
RG 101
DW 106.000 usec
DE 6.50 usec
TE 298.0 K
D0 0.00000300 sec
D1 0.99385548 sec
D11 0.03000000 sec
D12 0.00002000 sec
D13 0.00000400 sec
D16 0.00020000 sec
IN0 0.00021200 sec
TDav 1
SFO1 500.1323116 MHz
NUC1 1H
P0 8.20 usec
P1 8.20 usec
P17 2500.00 usec
PLW1 24.45700073 W
PLW10 1.82720006 W
GPNAM[1] SMSQ10.100
GPZ1 10.00 %
P16 1000.00 usec

F1 - Acquisition parameters
TD 256
SFO1 500.1323 MHz
FIDRES 36.851414 Hz
SW 9.431 ppm
FnMODE QF

F2 - Processing parameters
SI 2048
SF 500.1300584 MHz
WDW QSINE
SSB 0
LB 0 Hz
GB 0
PC 1.40

F1 - Processing parameters
SI 1024
MC2 QF
SF 500.1300584 MHz
WDW QSINE
SSB 0
LB 0 Hz
GB 0
