# Supplementary material for: Isocyanides as Acceptor Groups in MHAT Reactions with Unactivated Alkenes
Source: Org Lett. 2023 Aug 30;25(35):6539–43. doi: 10.1021/acs.orglett.3c02358 (PMC10496133; doi:10.1021/acs.orglett.3c02358)
Supplement: Supplementary file 2 — ol3c02358_si_002.zip [file ol3c02358_si_002.zip › FID for publication/FID for publication/Compound 7d/Compound 7d-HSQC.fid/pdata/1/san-11086-2022_13_1.pdf]

Register 11086/2022
Operator AGUSTIN MARTINEZ

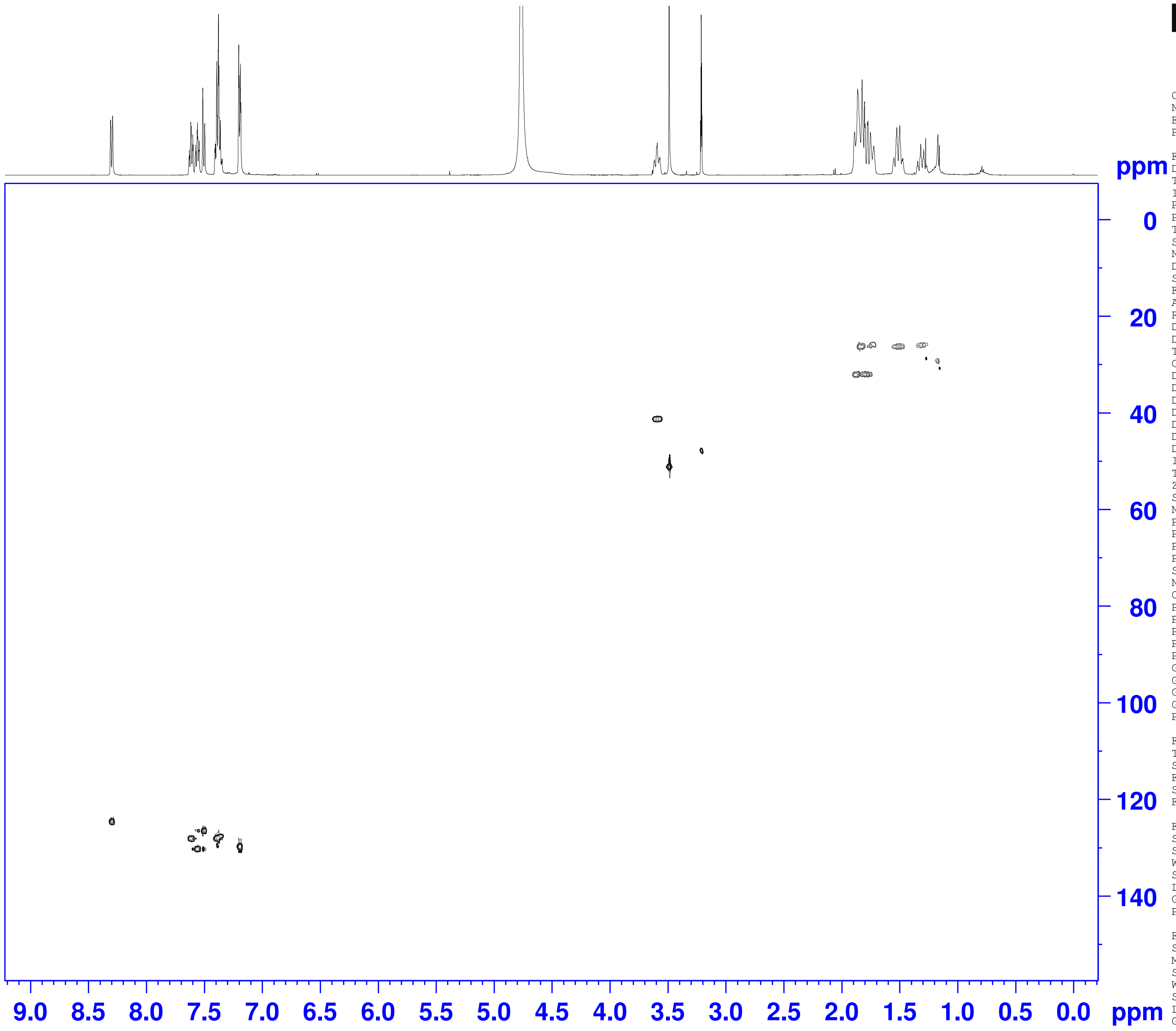

BRUKER

Current Data Parameters

NAME	san-11086-2022	
EXPNO	13	
PROCNO	1	

F2 - Acquisition Parameters

Date_	20221013	
Time	6.22	h
INSTRUM	Avance Neo	
PROBHD	Z151574_0054 (	
PULPROG	hsqcedetgp	
TD	2048	
SOLVENT	MeOD	
NS	8	
DS	32	
SWH	4716.981	Hz
FIDRES	4.606427	Hz
AQ	0.2170880	sec
RG	101	
DW	106.000	usec
DE	6.50	usec
TE	298.1	K
CNST2	145.0000000	
D0	0.00000300	sec
D1	1.50000000	sec
D4	0.00172414	sec
D11	0.03000000	sec
D13	0.00000400	sec
D16	0.00020000	sec
D21	0.00345000	sec
IN0	0.00002410	sec
TDav	1	
ZGOPTNS		
SFO1	500.1323116	MHz
NUC1	1H	
P1	8.20	usec
P2	16.40	usec
P28	0	usec
PLW1	24.45700073	W
SFO2	125.7672204	MHz
NUC2	13C	
CPDPRG[2	garp4	
P3	9.10	usec
P4	18.20	usec
PCPD2	70.00	usec
PLW2	102.19000244	W
PLW12	1.72700000	W
GPNAM[1]	SMSQ10.100	
GPZ1	80.00	%
GPNAM[2]	SMSQ10.100	
GPZ2	20.10	%
P16	1000.00	usec

F1 - Acquisition parameters

TD	256	
SFO1	125.7672	MHz
FIDRES	162.127106	Hz
SW	165.005	ppm
FnMODE	Echo-Antiecho	

F2 - Processing parameters

SI	2048	
SF	500.1300584	MHz
WDW	QSINE	
SSB	2	
LB	0	Hz
GB	0	
PC	1.40	

F1 - Processing parameters

SI	1024	
MC2	echo-antiecho	
SF	125.7577885	MHz
WDW	QSINE	
SSB	2	
LB	0	Hz
GB	0	
