# Supplementary material for: Isocyanides as Acceptor Groups in MHAT Reactions with Unactivated Alkenes
Source: Org Lett. 2023 Aug 30;25(35):6539–43. doi: 10.1021/acs.orglett.3c02358 (PMC10496133; doi:10.1021/acs.orglett.3c02358)
Supplement: Supplementary file 2 — ol3c02358_si_002.zip [file ol3c02358_si_002.zip › FID for publication/StructureFile_BRADSHAW.docx]

**Structure File**

The spectrometers used to collect the data are:

- **Varian VNMRS 400 MHz**
- **Varian Mercury 400 MHz**

The acquisition software used is VnmrJ.

The processing program used to analyze the data is MestreNova.

**2-Isocyano-1,1'-biphenyl (1a)**

SMILES Code: C#[N+]c1ccccc1c2ccccc2

MoleFile:

Actelion Java MolfileCreator 1.0

14 15 0 0 0 0 0 0 0 0999 V2000

2.5981	-3.7500	-0.0000 C	0 0 0 0 0 0 0 0 0 0 0 0

2.5981	-2.2500	-0.0000 N	0 0 0 0 0 0 0 0 0 0 0 0

2.5981	-0.7500	-0.0000 C	0 0 0 0 0 0 0 0 0 0 0 0

3.8971	-0.0000	-0.0000 C	0 0 0 0 0 0 0 0 0 0 0 0

3.8971	1.5000	-0.0000 C	0 0 0 0 0 0 0 0 0 0 0 0

2.5981	2.2500	-0.0000 C	0 0 0 0 0 0 0 0 0 0 0 0

1.2990	1.5000	-0.0000 C	0 0 0 0 0 0 0 0 0 0 0 0

1.2990	-0.0000	-0.0000 C	0 0 0 0 0 0 0 0 0 0 0 0

0.0000	-0.7500	-0.0000 C	0 0 0 0 0 0 0 0 0 0 0 0

-1.2990	0.0000	-0.0000 C	0 0 0 0 0 0 0 0 0 0 0 0

-2.5981	-0.7500	-0.0000 C	0 0 0 0 0 0 0 0 0 0 0 0

-2.5981	-2.2500	-0.0000 C	0 0 0 0 0 0 0 0 0 0 0 0

-1.2990	-3.0000	-0.0000 C	0 0 0 0 0 0 0 0 0 0 0 0

-0.0000	-2.2500	-0.0000 C	0 0 0 0 0 0 0 0 0 0 0 0

2 1 3 0 0 0 0

3 2 1 0 0 0 0

4 3 2 0 0 0 0

5 4 1 0 0 0 0

6 5 2 0 0 0 0

7 6 1 0 0 0 0

8 7 2 0 0 0 0

8 3 1 0 0 0 0

9 8 1 0 0 0 0

10 9 2 0 0 0 0

11 10 1 0 0 0 0

12 11 2 0 0 0 0

13 12 1 0 0 0 0

14 13 2 0 0 0 0

14 9 1 0 0 0 0

M CHG 1	2	1

M END

**2-isocyano-4'-methyl-1,1'-biphenyl (1b)**

SMILES Code: C#[N+]c1ccccc1c2ccc(C)cc2

MoleFile:

15 16 0 0 0 0 0 0 0 0999 V2000

2.4249	0.8082	0.0000 C	0 0 0 0 0 0 0 0 0 0 0 0

2.4249	2.2082	0.0000 C	0 0 0 0 0 0 0 0 0 0 0 0

1.2124	2.9082	0.0000 C	0 0 0 0 0 0 0 0 0 0 0 0

0.0000	2.2082	0.0000 C	0 0 0 0 0 0 0 0 0 0 0 0

0.0000	0.8082	0.0000 C	0 0 0 0 0 0 0 0 0 0 0 0

1.2124	0.1082	0.0000 C	0 0 0 0 0 0 0 0 0 0 0 0

3.6373	2.9082	0.0000 C	0 0 0 0 0 0 0 0 0 0 0 0

4.8497	2.2082	0.0000 C	0 0 0 0 0 0 0 0 0 0 0 0

6.0622	2.9082	0.0000 C	0 0 0 0 0 0 0 0 0 0 0 0

6.0622	4.3082	0.0000 C	0 0 0 0 0 0 0 0 0 0 0 0

4.8497	5.0082	0.0000 C	0 0 0 0 0 0 0 0 0 0 0 0

3.6373	4.3082	0.0000 C	0 0 0 0 0 0 0 0 0 0 0 0

7.2746	5.0082	0.0000 C	0 0 0 0 0 0 0 0 0 0 0 0

3.6373	0.1082	0.0000 N	0 3 0 0 0 0 0 0 0 0 0 0

5.0331	0.0000	0.0000 C	0 0 0 0 0 0 0 0 0 0 0 0

1 2 1 0 0 0 0

2 3 2 0 0 0 0

3 4 1 0 0 0 0

4 5 2 0 0 0 0

5 6 1 0 0 0 0

6 1 2 0 0 0 0

2 7 1 0 0 0 0

8 9 1 0 0 0 0

9 10 2 0 0 0 0

10 11 1 0 0 0 0

11 12 2 0 0 0 0

7 12 1 0 0 0 0

7 8 2 0 0 0 0

10 13 1 0 0 0 0

1 14 1 0 0 0 0

14 15 3 0 0 0 0

M CHG 1 14	1

M END

**4'-fluoro-2-isocyano-1,1'-biphenyl (1c)**

SMILES Code: C#[N+]c1ccccc1c2ccc(F)cc2

MoleFile:

15 16 0 0 0 0 0 0 0 0999 V2000

2.4249	0.8082	0.0000 C	0 0 0 0 0 0 0 0 0 0 0 0

2.4249	2.2082	0.0000 C	0 0 0 0 0 0 0 0 0 0 0 0

1.2124	2.9082	0.0000 C	0 0 0 0 0 0 0 0 0 0 0 0

0.0000	2.2082	0.0000 C	0 0 0 0 0 0 0 0 0 0 0 0

0.0000	0.8082	0.0000 C	0 0 0 0 0 0 0 0 0 0 0 0

1.2124	0.1082	0.0000 C	0 0 0 0 0 0 0 0 0 0 0 0

3.6373	2.9082	0.0000 C	0 0 0 0 0 0 0 0 0 0 0 0

4.8497	2.2082	0.0000 C	0 0 0 0 0 0 0 0 0 0 0 0

6.0622	2.9082	0.0000 C	0 0 0 0 0 0 0 0 0 0 0 0

6.0622	4.3082	0.0000 C	0 0 0 0 0 0 0 0 0 0 0 0

4.8497	5.0082	0.0000 C	0 0 0 0 0 0 0 0 0 0 0 0

3.6373	4.3082	0.0000 C	0 0 0 0 0 0 0 0 0 0 0 0

7.2746	5.0082	0.0000 F	0 0 0 0 0 0 0 0 0 0 0 0

3.6373	0.1082	0.0000 N	0 3 0 0 0 0 0 0 0 0 0 0

5.0331	0.0000	0.0000 C	0 0 0 0 0 0 0 0 0 0 0 0

1 2 1 0 0 0 0

2 3 2 0 0 0 0

3 4 1 0 0 0 0

4 5 2 0 0 0 0

5 6 1 0 0 0 0

6 1 2 0 0 0 0

2 7 1 0 0 0 0

8 9 1 0 0 0 0

9 10 2 0 0 0 0

10 11 1 0 0 0 0

11 12 2 0 0 0 0

7 12 1 0 0 0 0

7 8 2 0 0 0 0

10 13 1 0 0 0 0

1 14 1 0 0 0 0

14 15 3 0 0 0 0

M CHG 1 14	1

M END

**2-isocyano-4-methoxy-1,1'-biphenyl (1d)**

SMILES Code: C#[N+]c1cc(OC)ccc1c2ccccc2

MoleFile:

16 17 0 0 0 0 0 0 0 0999 V2000

4.8497	0.8082	0.0000 C	0 0 0 0 0 0 0 0 0 0 0 0

4.8497	2.2082	0.0000 C	0 0 0 0 0 0 0 0 0 0 0 0

3.6373	2.9082	0.0000 C	0 0 0 0 0 0 0 0 0 0 0 0

2.4248	2.2082	0.0000 C	0 0 0 0 0 0 0 0 0 0 0 0

2.4248	0.8082	0.0000 C	0 0 0 0 0 0 0 0 0 0 0 0

3.6373	0.1082	0.0000 C	0 0 0 0 0 0 0 0 0 0 0 0

6.0621	2.9082	0.0000 C	0 0 0 0 0 0 0 0 0 0 0 0

7.2746	2.2082	0.0000 C	0 0 0 0 0 0 0 0 0 0 0 0

8.4870	2.9082	0.0000 C	0 0 0 0 0 0 0 0 0 0 0 0

8.4870	4.3082	0.0000 C	0 0 0 0 0 0 0 0 0 0 0 0

7.2746	5.0082	0.0000 C	0 0 0 0 0 0 0 0 0 0 0 0

6.0621	4.3082	0.0000 C	0 0 0 0 0 0 0 0 0 0 0 0

6.0621	0.1082	0.0000 N	0 3 0 0 0 0 0 0 0 0 0 0

7.4580	0.0000	0.0000 C	0 0 0 0 0 0 0 0 0 0 0 0

1.2124	0.1082	0.0000 O	0 0 0 0 0 0 0 0 0 0 0 0

0.0000	0.8082	0.0000 C	0 0 0 0 0 0 0 0 0 0 0 0

1 2 1 0 0 0 0

2 3 2 0 0 0 0

3 4 1 0 0 0 0

4 5 2 0 0 0 0

5 6 1 0 0 0 0

6 1 2 0 0 0 0

2 7 1 0 0 0 0

8 9 1 0 0 0 0

9 10 2 0 0 0 0

10 11 1 0 0 0 0

11 12 2 0 0 0 0

7 12 1 0 0 0 0

7 8 2 0 0 0 0

1 13 1 0 0 0 0

13 14 3 0 0 0 0

5 15 1 0 0 0 0

15 16 1 0 0 0 0

M CHG 1 13	1

M END

**2-isocyano-5-(trifluoromethyl)-1,1'-biphenyl (1e)**

SMILES Code: C#[N+]c1ccc(C(F)(F)F)cc1c2ccccc2

MoleFile:

18 19 0 0 0 0 0 0 0 0999 V2000

4.8498	0.8082	0.0000 C	0 0 0 0 0 0 0 0 0 0 0 0

4.8498	2.2082	0.0000 C	0 0 0 0 0 0 0 0 0 0 0 0

3.6374	2.9082	0.0000 C	0 0 0 0 0 0 0 0 0 0 0 0

2.4249	2.2082	0.0000 C	0 0 0 0 0 0 0 0 0 0 0 0

2.4249	0.8082	0.0000 C	0 0 0 0 0 0 0 0 0 0 0 0

3.6374	0.1082	0.0000 C	0 0 0 0 0 0 0 0 0 0 0 0

6.0622	2.9082	0.0000 C	0 0 0 0 0 0 0 0 0 0 0 0

7.2747	2.2082	0.0000 C	0 0 0 0 0 0 0 0 0 0 0 0

8.4871	2.9082	0.0000 C	0 0 0 0 0 0 0 0 0 0 0 0

8.4871	4.3082	0.0000 C	0 0 0 0 0 0 0 0 0 0 0 0

7.2747	5.0082	0.0000 C	0 0 0 0 0 0 0 0 0 0 0 0

6.0622	4.3082	0.0000 C	0 0 0 0 0 0 0 0 0 0 0 0

6.0622	0.1082	0.0000 N	0 3 0 0 0 0 0 0 0 0 0 0

7.4581	0.0000	0.0000 C	0 0 0 0 0 0 0 0 0 0 0 0

1.2125	2.9082	0.0000 C	0 0 0 0 0 0 0 0 0 0 0 0

1.2124	4.3082	0.0000 F	0 0 0 0 0 0 0 0 0 0 0 0

0.0000	2.2081	0.0000 F	0 0 0 0 0 0 0 0 0 0 0 0

0.0000	3.6081	0.0000 F	0 0 0 0 0 0 0 0 0 0 0 0

1 2 1 0 0 0 0

2 3 2 0 0 0 0

3 4 1 0 0 0 0

4 5 2 0 0 0 0

5 6 1 0 0 0 0

6 1 2 0 0 0 0

2 7 1 0 0 0 0

8 9 1 0 0 0 0

9 10 2 0 0 0 0

10 11 1 0 0 0 0

11 12 2 0 0 0 0

7 12 1 0 0 0 0

7 8 2 0 0 0 0

1 13 1 0 0 0 0

13 14 3 0 0 0 0

4 15 1 0 0 0 0

15 16 1 0 0 0 0

15 17 1 0 0 0 0

15 18 1 0 0 0 0

M CHG 1 13	1

M END

**Methyl 2-isocyano-3,3-diphenylacrylate (2)**

SMILES Code: C#[N+]/C(C(=O)OC)=C(c1ccccc1)\c2ccccc2

MoleFille:

Actelion Java MolfileCreator 1.0

20 21 0 0 0 0 0 0 0 0999 V2000

0.0000	-2.5000	-0.0000 C	0 0 0 0 0 0 0 0 0 0 0 0

0.8660	-2.0000	-0.0000 N	0 0 0 0 0 0 0 0 0 0 0 0

1.7321	-1.5000	-0.0000 C	0 0 0 0 0 0 0 0 0 0 0 0

1.7321	-0.5000	-0.0000 C	0 0 0 0 0 0 0 0 0 0 0 0

2.5981	-0.0000	-0.0000 O	0 0 0 0 0 0 0 0 0 0 0 0

0.8660	-0.0000	-0.0000 O	0 0 0 0 0 0 0 0 0 0 0 0

0.0000	-0.5000	-0.0000 C	0 0 0 0 0 0 0 0 0 0 0 0

2.5981	-2.0000	-0.0000 C	0 0 0 0 0 0 0 0 0 0 0 0

3.4641	-1.5000	-0.0000 C	0 0 0 0 0 0 0 0 0 0 0 0

4.3301	-2.0000	-0.0000 C	0 0 0 0 0 0 0 0 0 0 0 0

5.1962	-1.5000	-0.0000 C	0 0 0 0 0 0 0 0 0 0 0 0

5.1962	-0.5000	-0.0000 C	0 0 0 0 0 0 0 0 0 0 0 0

4.3301	0.0000	-0.0000 C	0 0 0 0 0 0 0 0 0 0 0 0

3.4641	-0.5000	-0.0000 C	0 0 0 0 0 0 0 0 0 0 0 0

2.5981	-3.0000	-0.0000 C	0 0 0 0 0 0 0 0 0 0 0 0

1.7321	-3.5000	-0.0000 C	0 0 0 0 0 0 0 0 0 0 0 0

1.7321	-4.5000	-0.0000 C	0 0 0 0 0 0 0 0 0 0 0 0

2.5981	-5.0000	-0.0000 C	0 0 0 0 0 0 0 0 0 0 0 0

3.4641	-4.5000	-0.0000 C	0 0 0 0 0 0 0 0 0 0 0 0

3.4641	-3.5000	-0.0000 C	0 0 0 0 0 0 0 0 0 0 0 0

2 1 3 0 0 0 0

3 2 1 0 0 0 0

4 3 1 0 0 0 0

5 4 2 0 0 0 0

6 4 1 0 0 0 0

7 6 1 0 0 0 0

8 3 2 0 0 0 0

9 8 1 0 0 0 0

10 9 2 0 0 0 0

11 10 1 0 0 0 0

12 11 2 0 0 0 0

13 12 1 0 0 0 0

14 13 2 0 0 0 0

14 9 1 0 0 0 0

15 8 1 0 0 0 0

16 15 2 0 0 0 0

17 16 1 0 0 0 0

18 17 2 0 0 0 0

19 18 1 0 0 0 0

20 19 2 0 0 0 0

20 15 1 0 0 0 0

M CHG 1	2	1

M END

**3-(2-Isocyanophenyl)-1-phenylprop-2-en-1-one (3)**

SMILES Code: C#[N+]c1ccccc1/C=C/C(=O)c2ccccc2

MoleFile:

Actelion Java MolfileCreator 1.0

18 19 0 0 0 0 0 0 0 0999 V2000

2.5981	-3.7500	-0.0000 C	0 0 0 0 0 0 0 0 0 0 0 0

3.8971	-3.0000	-0.0000 N	0 0 0 0 0 0 0 0 0 0 0 0

5.1962	-2.2500	-0.0000 C	0 0 0 0 0 0 0 0 0 0 0 0

6.4952	-3.0000	-0.0000 C	0 0 0 0 0 0 0 0 0 0 0 0

7.7942	-2.2500	-0.0000 C	0 0 0 0 0 0 0 0 0 0 0 0

7.7942	-0.7500	-0.0000 C	0 0 0 0 0 0 0 0 0 0 0 0

6.4952	0.0000	-0.0000 C	0 0 0 0 0 0 0 0 0 0 0 0

5.1962	-0.7500	-0.0000 C	0 0 0 0 0 0 0 0 0 0 0 0

3.8971	-0.0000	-0.0000 C	0 0 0 0 0 0 0 0 0 0 0 0

2.5981	-0.7500	-0.0000 C	0 0 0 0 0 0 0 0 0 0 0 0

1.2990	-0.0000	-0.0000 C	0 0 0 0 0 0 0 0 0 0 0 0

1.2990	1.5000	-0.0000 O	0 0 0 0 0 0 0 0 0 0 0 0

0.0000	-0.7500	-0.0000 C	0 0 0 0 0 0 0 0 0 0 0 0

-1.2990	0.0000	-0.0000 C	0 0 0 0 0 0 0 0 0 0 0 0

-2.5981	-0.7500	-0.0000 C	0 0 0 0 0 0 0 0 0 0 0 0

-2.5981	-2.2500	-0.0000 C	0 0 0 0 0 0 0 0 0 0 0 0

-1.2990	-3.0000	-0.0000 C	0 0 0 0 0 0 0 0 0 0 0 0

-0.0000	-2.2500	-0.0000 C	0 0 0 0 0 0 0 0 0 0 0 0

2 1 3 0 0 0 0

3 2 1 0 0 0 0

4 3 2 0 0 0 0

5 4 1 0 0 0 0

6 5 2 0 0 0 0

7 6 1 0 0 0 0

8 7 2 0 0 0 0

8 3 1 0 0 0 0

9 8 1 0 0 0 0

10 9 2 0 0 0 0

11 10 1 0 0 0 0

12 11 2 0 0 0 0

13 11 1 0 0 0 0

14 13 2 0 0 0 0

15 14 1 0 0 0 0

16 15 2 0 0 0 0

17 16 1 0 0 0 0

18 17 2 0 0 0 0

18 13 1 0 0 0 0

M CHG 1	2	1

M END

**Ethyl 3-(2-isocyanophenyl)acrylate (4)**

SMILES Code: C#[N+]c1ccccc1/C=C/C(=O)OCC

MoleFile:

Actelion Java MolfileCreator 1.0

15 15 0 0 0 0 0 0 0 0999 V2000

5.1962	-3.7500	-0.0000 C	0 0 0 0 0 0 0 0 0 0 0 0

6.4952	-3.0000	-0.0000 N	0 0 0 0 0 0 0 0 0 0 0 0

7.7942	-2.2500	-0.0000 C	0 0 0 0 0 0 0 0 0 0 0 0

9.0933	-3.0000	-0.0000 C	0 0 0 0 0 0 0 0 0 0 0 0

10.3923	-2.2500	-0.0000 C	0 0 0 0 0 0 0 0 0 0 0 0

10.3923	-0.7500	-0.0000 C	0 0 0 0 0 0 0 0 0 0 0 0

9.0933	-0.0000	-0.0000 C	0 0 0 0 0 0 0 0 0 0 0 0

7.7942	-0.7500	-0.0000 C	0 0 0 0 0 0 0 0 0 0 0 0

6.4952	-0.0000	-0.0000 C	0 0 0 0 0 0 0 0 0 0 0 0

5.1962	-0.7500	-0.0000 C	0 0 0 0 0 0 0 0 0 0 0 0

3.8971	-0.0000	-0.0000 C	0 0 0 0 0 0 0 0 0 0 0 0

3.8971	1.5000	-0.0000 O	0 0 0 0 0 0 0 0 0 0 0 0

2.5981	-0.7500	-0.0000 O	0 0 0 0 0 0 0 0 0 0 0 0

1.2990	-0.0000	-0.0000 C	0 0 0 0 0 0 0 0 0 0 0 0

0.0000	-0.7500	-0.0000 C	0 0 0 0 0 0 0 0 0 0 0 0

2 1 3 0 0 0 0

3 2 1 0 0 0 0

4 3 2 0 0 0 0

5 4 1 0 0 0 0

6 5 2 0 0 0 0

7 6 1 0 0 0 0

8 7 2 0 0 0 0

8 3 1 0 0 0 0

9 8 1 0 0 0 0

10 9 2 0 0 0 0

11 10 1 0 0 0 0

12 11 2 0 0 0 0

13 11 1 0 0 0 0

14 13 1 0 0 0 0

15 14 1 0 0 0 0

M CHG 1	2	1

M END

**1-Isocyano-2-(3-methoxyprop-1-en-1-yl)benzene (5)**

SMILES Code: C#[N+]c1ccccc1/C=C/COC

MoleFile:

Actelion Java MolfileCreator 1.0

13 13 0 0 0 0 0 0 0 0999 V2000

7.7942	-3.7500	-0.0000 C	0 0 0 0 0 0 0 0 0 0 0 0

7.7942	-2.2500	-0.0000 N	0 0 0 0 0 0 0 0 0 0 0 0

7.7942	-0.7500	-0.0000 C	0 0 0 0 0 0 0 0 0 0 0 0

9.0933	-0.0000	-0.0000 C	0 0 0 0 0 0 0 0 0 0 0 0

9.0933	1.5000	-0.0000 C	0 0 0 0 0 0 0 0 0 0 0 0

7.7942	2.2500	-0.0000 C	0 0 0 0 0 0 0 0 0 0 0 0

6.4952	1.5000	-0.0000 C	0 0 0 0 0 0 0 0 0 0 0 0

6.4952	-0.0000	-0.0000 C	0 0 0 0 0 0 0 0 0 0 0 0

5.1962	-0.7500	-0.0000 C	0 0 0 0 0 0 0 0 0 0 0 0

3.8971	-0.0000	-0.0000 C	0 0 0 0 0 0 0 0 0 0 0 0

2.5981	-0.7500	-0.0000 C	0 0 0 0 0 0 0 0 0 0 0 0

1.2990	-0.0000	-0.0000 O	0 0 0 0 0 0 0 0 0 0 0 0

0.0000	-0.7500	-0.0000 C	0 0 0 0 0 0 0 0 0 0 0 0

2 1 3 0 0 0 0

3 2 1 0 0 0 0

4 3 2 0 0 0 0

5 4 1 0 0 0 0

6 5 2 0 0 0 0

7 6 1 0 0 0 0

8 7 2 0 0 0 0

8 3 1 0 0 0 0

9 8 1 0 0 0 0

10 9 2 0 0 0 0

11 10 1 0 0 0 0

12 11 1 0 0 0 0

13 12 1 0 0 0 0

M CHG 1	2	1

M END

**Phenanthridine (6)**

SMILES Code: c1ccc3c(c1)cnc2ccccc23

MoleFile:

Actelion Java MolfileCreator 1.0

14 16 0 0 0 0 0 0 0 0999 V2000

0.0000	-0.0000	-0.0000 C	0 0 0 0 0 0 0 0 0 0 0 0

0.0000	-1.5000	-0.0000 C	0 0 0 0 0 0 0 0 0 0 0 0

1.2990	-2.2500	-0.0000 C	0 0 0 0 0 0 0 0 0 0 0 0

2.5981	-1.5000	-0.0000 C	0 0 0 0 0 0 0 0 0 0 0 0

2.5981	0.0000	-0.0000 C	0 0 0 0 0 0 0 0 0 0 0 0

1.2990	0.7500	-0.0000 C	0 0 0 0 0 0 0 0 0 0 0 0

3.8971	0.7500	-0.0000 C	0 0 0 0 0 0 0 0 0 0 0 0

5.1962	-0.0000	-0.0000 N	0 0 0 0 0 0 0 0 0 0 0 0

5.1962	-1.5000	-0.0000 C	0 0 0 0 0 0 0 0 0 0 0 0

6.4952	-2.2500	-0.0000 C	0 0 0 0 0 0 0 0 0 0 0 0

6.4952	-3.7500	-0.0000 C	0 0 0 0 0 0 0 0 0 0 0 0

5.1962	-4.5000	-0.0000 C	0 0 0 0 0 0 0 0 0 0 0 0

3.8971	-3.7500	-0.0000 C	0 0 0 0 0 0 0 0 0 0 0 0

3.8971	-2.2500	-0.0000 C	0 0 0 0 0 0 0 0 0 0 0 0

2 1 2 0 0 0 0

3 2 1 0 0 0 0

4 3 2 0 0 0 0

5 4 1 0 0 0 0

6 5 2 0 0 0 0

6 1 1 0 0 0 0

7 5 1 0 0 0 0

8 7 2 0 0 0 0

9 8 1 0 0 0 0

10 9 2 0 0 0 0

11 10 1 0 0 0 0

12 11 2 0 0 0 0

13 12 1 0 0 0 0

14 13 2 0 0 0 0

14 9 1 0 0 0 0

14 4 1 0 0 0 0

M END

**3-Methoxycarbonyl-4-phenylisoquinoline (7)**

SMILES Code: COC(=O)c2ncc1ccccc1c2c3ccccc3

MoleFile:

Actelion Java MolfileCreator 1.0

20 22 0 0 0 0 0 0 0 0999 V2000

3.8971	-0.0000	-0.0000 C	0 0 0 0 0 0 0 0 0 0 0 0

2.5981	-0.7500	-0.0000 O	0 0 0 0 0 0 0 0 0 0 0 0

1.2990	-0.0000	-0.0000 C	0 0 0 0 0 0 0 0 0 0 0 0

1.2990	1.5000	-0.0000 O	0 0 0 0 0 0 0 0 0 0 0 0

0.0000	-0.7500	-0.0000 C	0 0 0 0 0 0 0 0 0 0 0 0

-1.2990	0.0000	-0.0000 N	0 0 0 0 0 0 0 0 0 0 0 0

-2.5981	-0.7500	-0.0000 C	0 0 0 0 0 0 0 0 0 0 0 0

-2.5981	-2.2500	-0.0000 C	0 0 0 0 0 0 0 0 0 0 0 0

-3.8971	-3.0000	-0.0000 C	0 0 0 0 0 0 0 0 0 0 0 0

-3.8971	-4.5000	-0.0000 C	0 0 0 0 0 0 0 0 0 0 0 0

-2.5981	-5.2500	-0.0000 C	0 0 0 0 0 0 0 0 0 0 0 0

-1.2990	-4.5000	-0.0000 C	0 0 0 0 0 0 0 0 0 0 0 0

-1.2990	-3.0000	-0.0000 C	0 0 0 0 0 0 0 0 0 0 0 0

-0.0000	-2.2500	-0.0000 C	0 0 0 0 0 0 0 0 0 0 0 0

1.2990	-3.0000	-0.0000 C	0 0 0 0 0 0 0 0 0 0 0 0

1.2990	-4.5000	-0.0000 C	0 0 0 0 0 0 0 0 0 0 0 0

2.5981	-5.2500	-0.0000 C	0 0 0 0 0 0 0 0 0 0 0 0

3.8971	-4.5000	-0.0000 C	0 0 0 0 0 0 0 0 0 0 0 0

3.8971	-3.0000	-0.0000 C	0 0 0 0 0 0 0 0 0 0 0 0

2.5981	-2.2500	-0.0000 C	0 0 0 0 0 0 0 0 0 0 0 0

2 1 1 0 0 0 0

3 2 1 0 0 0 0

4 3 2 0 0 0 0

5 3 1 0 0 0 0

6 5 2 0 0 0 0

7 6 1 0 0 0 0

8 7 2 0 0 0 0

9 8 1 0 0 0 0

10 9 2 0 0 0 0

11 10 1 0 0 0 0

12 11 2 0 0 0 0

13 12 1 0 0 0 0

13 8 1 0 0 0 0

14 13 2 0 0 0 0

14 5 1 0 0 0 0

15 14 1 0 0 0 0

16 15 2 0 0 0 0

17 16 1 0 0 0 0

18 17 2 0 0 0 0

19 18 1 0 0 0 0

20 19 2 0 0 0 0

20 15 1 0 0 0 0

M END

´

**3-(2-Oxo-2-phenylethyl)indole (8)**

SMILES Code: O=C(Cc1c[nH]c2ccccc12)c3ccccc3

MoleFile:

Actelion Java MolfileCreator 1.0

18 20 0 0 0 0 0 0 0 0999 V2000

2.5981	-0.0000	-0.0000 O	0 0 0 0 0 0 0 0 0 0 0 0

1.7321	-0.5000	-0.0000 C	0 0 0 0 0 0 0 0 0 0 0 0

0.8660	-0.0000	-0.0000 C	0 0 0 0 0 0 0 0 0 0 0 0

0.0000	-0.5000	-0.0000 C	0 0 0 0 0 0 0 0 0 0 0 0

-0.1045	-1.4945	-0.0000 C	0 0 0 0 0 0 0 0 0 0 0 0

-1.0827	-1.7024	-0.0000 N	0 0 0 0 0 0 0 0 0 0 0 0

-1.5827	-0.8364	-0.0000 C	0 0 0 0 0 0 0 0 0 0 0 0

-2.5608	-0.6285	-0.0000 C	0 0 0 0 0 0 0 0 0 0 0 0

-2.8698	0.3226	-0.0000 C	0 0 0 0 0 0 0 0 0 0 0 0

-2.2007	1.0657	-0.0000 C	0 0 0 0 0 0 0 0 0 0 0 0

-1.2226	0.8578	-0.0000 C	0 0 0 0 0 0 0 0 0 0 0 0

-0.9135	-0.0933	-0.0000 C	0 0 0 0 0 0 0 0 0 0 0 0

1.7321	-1.5000	-0.0000 C	0 0 0 0 0 0 0 0 0 0 0 0

0.8660	-2.0000	-0.0000 C	0 0 0 0 0 0 0 0 0 0 0 0

0.8660	-3.0000	-0.0000 C	0 0 0 0 0 0 0 0 0 0 0 0

1.7321	-3.5000	-0.0000 C	0 0 0 0 0 0 0 0 0 0 0 0

2.5981	-3.0000	-0.0000 C	0 0 0 0 0 0 0 0 0 0 0 0

2.5981	-2.0000	-0.0000 C	0 0 0 0 0 0 0 0 0 0 0 0

2 1 2 0 0 0 0

3 2 1 0 0 0 0

4 3 1 0 0 0 0

5 4 2 0 0 0 0

6 5 1 0 0 0 0

7 6 1 0 0 0 0

8 7 2 0 0 0 0

9 8 1 0 0 0 0

10 9 2 0 0 0 0

11 10 1 0 0 0 0

12 11 2 0 0 0 0

12 4 1 0 0 0 0

12 7 1 0 0 0 0

13 2 1 0 0 0 0

14 13 2 0 0 0 0

15 14 1 0 0 0 0

16 15 2 0 0 0 0

17 16 1 0 0 0 0

18 17 2 0 0 0 0

18 13 1 0 0 0 0

M END

**Ethyl 3-indoleacetate (9)**

SMILES Code: CCOC(=O)Cc1c[nH]c2ccccc12

MoleFile:

Actelion Java MolfileCreator 1.0

15 16 0 0 0 0 0 0 0 0999 V2000

4.3301	-0.0000	-0.0000 C	0 0 0 0 0 0 0 0 0 0 0 0

3.4641	-0.5000	-0.0000 C	0 0 0 0 0 0 0 0 0 0 0 0

2.5981	-0.0000	-0.0000 O	0 0 0 0 0 0 0 0 0 0 0 0

1.7321	-0.5000	-0.0000 C	0 0 0 0 0 0 0 0 0 0 0 0

1.7321	-1.5000	-0.0000 O	0 0 0 0 0 0 0 0 0 0 0 0

0.8660	-0.0000	-0.0000 C	0 0 0 0 0 0 0 0 0 0 0 0

0.0000	-0.5000	-0.0000 C	0 0 0 0 0 0 0 0 0 0 0 0

-0.9135	-0.0933	-0.0000 C	0 0 0 0 0 0 0 0 0 0 0 0

-1.5827	-0.8364	-0.0000 N	0 0 0 0 0 0 0 0 0 0 0 0

-1.0827	-1.7024	-0.0000 C	0 0 0 0 0 0 0 0 0 0 0 0

-1.3917	-2.6535	-0.0000 C	0 0 0 0 0 0 0 0 0 0 0 0

-0.7226	-3.3966	-0.0000 C	0 0 0 0 0 0 0 0 0 0 0 0

0.2556	-3.1887	-0.0000 C	0 0 0 0 0 0 0 0 0 0 0 0

0.5646	-2.2377	-0.0000 C	0 0 0 0 0 0 0 0 0 0 0 0

-0.1045	-1.4945	-0.0000 C	0 0 0 0 0 0 0 0 0 0 0 0

2 1 1 0 0 0 0

3 2 1 0 0 0 0

4 3 1 0 0 0 0

5 4 2 0 0 0 0

6 4 1 0 0 0 0

7 6 1 0 0 0 0

8 7 2 0 0 0 0

9 8 1 0 0 0 0

10 9 1 0 0 0 0

11 10 2 0 0 0 0

12 11 1 0 0 0 0

13 12 2 0 0 0 0

14 13 1 0 0 0 0

15 14 2 0 0 0 0

15 7 1 0 0 0 0

15 10 1 0 0 0 0

M END

**6-(4-hydroxybutan-2-yl)phenanthridine (6a)**

SMILES Code: CC(CCO)c2nc1ccccc1c3ccccc23

MoleFile:

Actelion Java MolfileCreator 1.0

19 21 0 0 0 0 0 0 0 0999 V2000

3.4641	-0.5000	-0.0000 C	0 0 0 0 0 0 0 0 0 0 0 0

2.5981	-0.0000	-0.0000 C	0 0 0 0 0 0 0 0 0 0 0 0

1.7321	-0.5000	-0.0000 C	0 0 0 0 0 0 0 0 0 0 0 0

0.8660	-0.0000	-0.0000 C	0 0 0 0 0 0 0 0 0 0 0 0

0.0000	-0.5000	-0.0000 O	0 0 0 0 0 0 0 0 0 0 0 0

2.5981	1.0000	-0.0000 C	0 0 0 0 0 0 0 0 0 0 0 0

3.4641	1.5000	-0.0000 N	0 0 0 0 0 0 0 0 0 0 0 0

3.4641	2.5000	-0.0000 C	0 0 0 0 0 0 0 0 0 0 0 0

4.3301	3.0000	-0.0000 C	0 0 0 0 0 0 0 0 0 0 0 0

4.3301	4.0000	-0.0000 C	0 0 0 0 0 0 0 0 0 0 0 0

3.4641	4.5000	-0.0000 C	0 0 0 0 0 0 0 0 0 0 0 0

2.5981	4.0000	-0.0000 C	0 0 0 0 0 0 0 0 0 0 0 0

2.5981	3.0000	-0.0000 C	0 0 0 0 0 0 0 0 0 0 0 0

1.7321	2.5000	-0.0000 C	0 0 0 0 0 0 0 0 0 0 0 0

0.8660	3.0000	-0.0000 C	0 0 0 0 0 0 0 0 0 0 0 0

-0.0000	2.5000	-0.0000 C	0 0 0 0 0 0 0 0 0 0 0 0

0.0000	1.5000	-0.0000 C	0 0 0 0 0 0 0 0 0 0 0 0

0.8660	1.0000	-0.0000 C	0 0 0 0 0 0 0 0 0 0 0 0

1.7321	1.5000	-0.0000 C	0 0 0 0 0 0 0 0 0 0 0 0

2 1 1 0 0 0 0

3 2 1 0 0 0 0

4 3 1 0 0 0 0

5 4 1 0 0 0 0

6 2 1 0 0 0 0

7 6 2 0 0 0 0

8 7 1 0 0 0 0

9 8 1 0 0 0 0

10 9 2 0 0 0 0

11 10 1 0 0 0 0

12 11 2 0 0 0 0

13 12 1 0 0 0 0

13 8 2 0 0 0 0

14 13 1 0 0 0 0

15 14 1 0 0 0 0

16 15 2 0 0 0 0

17 16 1 0 0 0 0

18 17 2 0 0 0 0

19 18 1 0 0 0 0

19 6 1 0 0 0 0

19 14 2 0 0 0 0

M END

**6-(4-hydroxybutan-2-yl)-8-methylphenanthridine (6b)**

SMILES Code: Cc1ccc3c(c1)c(C(C)CCO)nc2ccccc23

MoleFile:

20 22 0 0 0 0 0 0 0 0999 V2000

2.4249	2.1000	0.0000 C	0 0 0 0 0 0 0 0 0 0 0 0

2.4249	3.5000	0.0000 C	0 0 0 0 0 0 0 0 0 0 0 0

1.2124	4.2000	0.0000 C	0 0 0 0 0 0 0 0 0 0 0 0

0.0000	3.5000	0.0000 C	0 0 0 0 0 0 0 0 0 0 0 0

0.0000	2.1000	0.0000 C	0 0 0 0 0 0 0 0 0 0 0 0

1.2124	1.4000	0.0000 C	0 0 0 0 0 0 0 0 0 0 0 0

3.6373	1.4000	0.0000 N	0 0 0 0 0 0 0 0 0 0 0 0

4.8497	2.1000	0.0000 C	0 0 0 0 0 0 0 0 0 0 0 0

4.8497	3.5000	0.0000 C	0 0 0 0 0 0 0 0 0 0 0 0

3.6373	4.2000	0.0000 C	0 0 0 0 0 0 0 0 0 0 0 0

6.0622	4.2000	0.0000 C	0 0 0 0 0 0 0 0 0 0 0 0

6.0622	5.6000	0.0000 C	0 0 0 0 0 0 0 0 0 0 0 0

4.8497	6.3000	0.0000 C	0 0 0 0 0 0 0 0 0 0 0 0

3.6373	5.6000	0.0000 C	0 0 0 0 0 0 0 0 0 0 0 0

7.2746	6.3000	0.0000 C	0 0 0 0 0 0 0 0 0 0 0 0

6.0622	1.4000	0.0000 C	0 0 0 0 0 0 0 0 0 0 0 0

6.0622	0.0000	0.0000 C	0 0 0 0 0 0 0 0 0 0 0 0

7.2746	2.1000	0.0000 C	0 0 0 0 0 0 0 0 0 0 0 0

8.4870	1.4000	0.0000 C	0 0 0 0 0 0 0 0 0 0 0 0

9.6995	2.1000	0.0000 O	0 0 0 0 0 0 0 0 0 0 0 0

1 2 1 0 0 0 0

2 3 2 0 0 0 0

3 4 1 0 0 0 0

4 5 2 0 0 0 0

5 6 1 0 0 0 0

6 1 2 0 0 0 0

7 8 2 0 0 0 0

8 9 1 0 0 0 0

9 10 2 0 0 0 0

2 10 1 0 0 0 0

1 7 1 0 0 0 0

11 12 2 0 0 0 0

12 13 1 0 0 0 0

13 14 2 0 0 0 0

10 14 1 0 0 0 0

9 11 1 0 0 0 0

12 15 1 0 0 0 0

8 16 1 0 0 0 0

16 17 1 0 0 0 0

16 18 1 0 0 0 0

18 19 1 0 0 0 0

19 20 1 0 0 0 0

M END

**6-(4-hydroxybutan-2-yl)-8-fluorophenanthridine (6c)**

SMILES Code: CC(CCO)c2nc1ccccc1c3ccc(F)cc23

MoleFile:

20 22 0 0 0 0 0 0 0 0999 V2000

2.4249	2.1000	0.0000 C	0 0 0 0 0 0 0 0 0 0 0 0

2.4249	3.5000	0.0000 C	0 0 0 0 0 0 0 0 0 0 0 0

1.2124	4.2000	0.0000 C	0 0 0 0 0 0 0 0 0 0 0 0

0.0000	3.5000	0.0000 C	0 0 0 0 0 0 0 0 0 0 0 0

0.0000	2.1000	0.0000 C	0 0 0 0 0 0 0 0 0 0 0 0

1.2124	1.4000	0.0000 C	0 0 0 0 0 0 0 0 0 0 0 0

3.6373	1.4000	0.0000 N	0 0 0 0 0 0 0 0 0 0 0 0

4.8497	2.1000	0.0000 C	0 0 0 0 0 0 0 0 0 0 0 0

4.8497	3.5000	0.0000 C	0 0 0 0 0 0 0 0 0 0 0 0

3.6373	4.2000	0.0000 C	0 0 0 0 0 0 0 0 0 0 0 0

6.0622	4.2000	0.0000 C	0 0 0 0 0 0 0 0 0 0 0 0

6.0622	5.6000	0.0000 C	0 0 0 0 0 0 0 0 0 0 0 0

4.8497	6.3000	0.0000 C	0 0 0 0 0 0 0 0 0 0 0 0

3.6373	5.6000	0.0000 C	0 0 0 0 0 0 0 0 0 0 0 0

7.2746	6.3000	0.0000 F	0 0 0 0 0 0 0 0 0 0 0 0

6.0622	1.4000	0.0000 C	0 0 0 0 0 0 0 0 0 0 0 0

6.0622	0.0000	0.0000 C	0 0 0 0 0 0 0 0 0 0 0 0

7.2746	2.1000	0.0000 C	0 0 0 0 0 0 0 0 0 0 0 0

8.4870	1.4000	0.0000 C	0 0 0 0 0 0 0 0 0 0 0 0

9.6995	2.1000	0.0000 O	0 0 0 0 0 0 0 0 0 0 0 0

1 2 1 0 0 0 0

2 3 2 0 0 0 0

3 4 1 0 0 0 0

4 5 2 0 0 0 0

5 6 1 0 0 0 0

6 1 2 0 0 0 0

7 8 2 0 0 0 0

8 9 1 0 0 0 0

9 10 2 0 0 0 0

2 10 1 0 0 0 0

1 7 1 0 0 0 0

11 12 2 0 0 0 0

12 13 1 0 0 0 0

13 14 2 0 0 0 0

10 14 1 0 0 0 0

9 11 1 0 0 0 0

12 15 1 0 0 0 0

8 16 1 0 0 0 0

16 17 1 0 0 0 0

16 18 1 0 0 0 0

18 19 1 0 0 0 0

19 20 1 0 0 0 0

M END

**6-(4-hydroxybutan-2-yl)-3-methoxyphenanthridine (6d)**

SMILES Code: COc1ccc3c(c1)nc(C(C)CCO)c2ccccc23

MoleFile:

21 23 0 0 0 0 0 0 0 0999 V2000

4.8497	2.1000	0.0000 C	0 0 0 0 0 0 0 0 0 0 0 0

4.8497	3.5000	0.0000 C	0 0 0 0 0 0 0 0 0 0 0 0

3.6373	4.2000	0.0000 C	0 0 0 0 0 0 0 0 0 0 0 0

2.4249	3.5000	0.0000 C	0 0 0 0 0 0 0 0 0 0 0 0

2.4249	2.1000	0.0000 C	0 0 0 0 0 0 0 0 0 0 0 0

3.6373	1.4000	0.0000 C	0 0 0 0 0 0 0 0 0 0 0 0

6.0622	1.4000	0.0000 N	0 0 0 0 0 0 0 0 0 0 0 0

7.2746	2.1000	0.0000 C	0 0 0 0 0 0 0 0 0 0 0 0

7.2746	3.5000	0.0000 C	0 0 0 0 0 0 0 0 0 0 0 0

6.0622	4.2000	0.0000 C	0 0 0 0 0 0 0 0 0 0 0 0

8.4870	4.2000	0.0000 C	0 0 0 0 0 0 0 0 0 0 0 0

8.4870	5.6000	0.0000 C	0 0 0 0 0 0 0 0 0 0 0 0

7.2746	6.3000	0.0000 C	0 0 0 0 0 0 0 0 0 0 0 0

6.0622	5.6000	0.0000 C	0 0 0 0 0 0 0 0 0 0 0 0

8.4870	1.4000	0.0000 C	0 0 0 0 0 0 0 0 0 0 0 0

8.4870	0.0000	0.0000 C	0 0 0 0 0 0 0 0 0 0 0 0

9.6995	2.1000	0.0000 C	0 0 0 0 0 0 0 0 0 0 0 0

10.9119	1.4000	0.0000 C	0 0 0 0 0 0 0 0 0 0 0 0

12.1244	2.1000	0.0000 O	0 0 0 0 0 0 0 0 0 0 0 0

1.2124	1.4000	0.0000 O	0 0 0 0 0 0 0 0 0 0 0 0

0.0000	2.1000	0.0000 C	0 0 0 0 0 0 0 0 0 0 0 0

1 2 1 0 0 0 0

2 3 2 0 0 0 0

3 4 1 0 0 0 0

4 5 2 0 0 0 0

5 6 1 0 0 0 0

6 1 2 0 0 0 0

7 8 2 0 0 0 0

8 9 1 0 0 0 0

9 10 2 0 0 0 0

2 10 1 0 0 0 0

1 7 1 0 0 0 0

11 12 2 0 0 0 0

12 13 1 0 0 0 0

13 14 2 0 0 0 0

10 14 1 0 0 0 0

9 11 1 0 0 0 0

8 15 1 0 0 0 0

15 16 1 0 0 0 0

15 17 1 0 0 0 0

17 18 1 0 0 0 0

18 19 1 0 0 0 0

5 20 1 0 0 0 0

20 21 1 0 0 0 0

M END

**6-(4-hydroxybutan-2-yl)-2-(trifluoromethyl)phenanthridine (6e)**

SMILES Code: CC(CCO)c2nc1ccc(C(F)(F)F)cc1c3ccccc23

MoleFile:

23 25 0 0 0 0 0 0 0 0999 V2000

4.8497	2.1000	0.0000 C	0 0 0 0 0 0 0 0 0 0 0 0

4.8497	3.5000	0.0000 C	0 0 0 0 0 0 0 0 0 0 0 0

3.6373	4.2000	0.0000 C	0 0 0 0 0 0 0 0 0 0 0 0

2.4249	3.5000	0.0000 C	0 0 0 0 0 0 0 0 0 0 0 0

2.4249	2.1000	0.0000 C	0 0 0 0 0 0 0 0 0 0 0 0

3.6373	1.4000	0.0000 C	0 0 0 0 0 0 0 0 0 0 0 0

6.0622	1.4000	0.0000 N	0 0 0 0 0 0 0 0 0 0 0 0

7.2746	2.1000	0.0000 C	0 0 0 0 0 0 0 0 0 0 0 0

7.2746	3.5000	0.0000 C	0 0 0 0 0 0 0 0 0 0 0 0

6.0622	4.2000	0.0000 C	0 0 0 0 0 0 0 0 0 0 0 0

8.4870	4.2000	0.0000 C	0 0 0 0 0 0 0 0 0 0 0 0

8.4870	5.6000	0.0000 C	0 0 0 0 0 0 0 0 0 0 0 0

7.2746	6.3000	0.0000 C	0 0 0 0 0 0 0 0 0 0 0 0

6.0622	5.6000	0.0000 C	0 0 0 0 0 0 0 0 0 0 0 0

8.4870	1.4000	0.0000 C	0 0 0 0 0 0 0 0 0 0 0 0

8.4870	0.0000	0.0000 C	0 0 0 0 0 0 0 0 0 0 0 0

9.6995	2.1000	0.0000 C	0 0 0 0 0 0 0 0 0 0 0 0

10.9119	1.4000	0.0000 C	0 0 0 0 0 0 0 0 0 0 0 0

12.1244	2.1000	0.0000 O	0 0 0 0 0 0 0 0 0 0 0 0

1.2124	4.2000	0.0000 C	0 0 0 0 0 0 0 0 0 0 0 0

1.2124	5.6000	0.0000 F	0 0 0 0 0 0 0 0 0 0 0 0

0.0000	3.5000	0.0000 F	0 0 0 0 0 0 0 0 0 0 0 0

0.0000	4.9000	0.0000 F	0 0 0 0 0 0 0 0 0 0 0 0

1 2 1 0 0 0 0

2 3 2 0 0 0 0

3 4 1 0 0 0 0

4 5 2 0 0 0 0

5 6 1 0 0 0 0

6 1 2 0 0 0 0

7 8 2 0 0 0 0

8 9 1 0 0 0 0

9 10 2 0 0 0 0

2 10 1 0 0 0 0

1 7 1 0 0 0 0

11 12 2 0 0 0 0

12 13 1 0 0 0 0

13 14 2 0 0 0 0

10 14 1 0 0 0 0

9 11 1 0 0 0 0

8 15 1 0 0 0 0

15 16 1 0 0 0 0

15 17 1 0 0 0 0

17 18 1 0 0 0 0

18 19 1 0 0 0 0

4 20 1 0 0 0 0

20 21 1 0 0 0 0

20 22 1 0 0 0 0

20 23 1 0 0 0 0

M END

**6-(dodecan-2-yl)phenanthridine** (**6f**)

SMILES Code: CCCCCCCCCCC(C)c2nc1ccccc1c3ccccc23

MoleFile:

Actelion Java MolfileCreator 1.0

26 28 0 0 0 0 0 0 0 0999 V2000

9.5263	-0.0000	-0.0000 C	0 0 0 0 0 0 0 0 0 0 0 0

8.6603	-0.5000	-0.0000 C	0 0 0 0 0 0 0 0 0 0 0 0

7.7942	-0.0000	-0.0000 C	0 0 0 0 0 0 0 0 0 0 0 0

6.9282	-0.5000	-0.0000 C	0 0 0 0 0 0 0 0 0 0 0 0

6.0622	-0.0000	-0.0000 C	0 0 0 0 0 0 0 0 0 0 0 0

5.1962	-0.5000	-0.0000 C	0 0 0 0 0 0 0 0 0 0 0 0

4.3301	-0.0000	-0.0000 C	0 0 0 0 0 0 0 0 0 0 0 0

3.4641	-0.5000	-0.0000 C	0 0 0 0 0 0 0 0 0 0 0 0

2.5981	-0.0000	-0.0000 C	0 0 0 0 0 0 0 0 0 0 0 0

1.7321	-0.5000	-0.0000 C	0 0 0 0 0 0 0 0 0 0 0 0

0.8660	-0.0000	-0.0000 C	0 0 0 0 0 0 0 0 0 0 0 0

0.8660	1.0000	-0.0000 C	0 0 0 0 0 0 0 0 0 0 0 0

0.0000	-0.5000	-0.0000 C	0 0 0 0 0 0 0 0 0 0 0 0

-0.8660	0.0000	-0.0000 N	0 0 0 0 0 0 0 0 0 0 0 0

-1.7321	-0.5000	-0.0000 C	0 0 0 0 0 0 0 0 0 0 0 0

-2.5981	0.0000	-0.0000 C	0 0 0 0 0 0 0 0 0 0 0 0

-3.4641	-0.5000	-0.0000 C	0 0 0 0 0 0 0 0 0 0 0 0

-3.4641	-1.5000	-0.0000 C	0 0 0 0 0 0 0 0 0 0 0 0

-2.5981	-2.0000	-0.0000 C	0 0 0 0 0 0 0 0 0 0 0 0

-1.7321	-1.5000	-0.0000 C	0 0 0 0 0 0 0 0 0 0 0 0

-0.8660	-2.0000	-0.0000 C	0 0 0 0 0 0 0 0 0 0 0 0

-0.8660	-3.0000	-0.0000 C	0 0 0 0 0 0 0 0 0 0 0 0

-0.0000	-3.5000	-0.0000 C	0 0 0 0 0 0 0 0 0 0 0 0

0.8660	-3.0000	-0.0000 C	0 0 0 0 0 0 0 0 0 0 0 0

0.8660	-2.0000	-0.0000 C	0 0 0 0 0 0 0 0 0 0 0 0

-0.0000	-1.5000	-0.0000 C	0 0 0 0 0 0 0 0 0 0 0 0

2 1 1 0 0 0 0

3 2 1 0 0 0 0

4 3 1 0 0 0 0

5 4 1 0 0 0 0

6 5 1 0 0 0 0

7 6 1 0 0 0 0

8 7 1 0 0 0 0

9 8 1 0 0 0 0

10 9 1 0 0 0 0

11 10 1 0 0 0 0

12 11 1 0 0 0 0

13 11 1 0 0 0 0

14 13 2 0 0 0 0

15 14 1 0 0 0 0

16 15 1 0 0 0 0

17 16 2 0 0 0 0

18 17 1 0 0 0 0

19 18 2 0 0 0 0

20 19 1 0 0 0 0

20 15 2 0 0 0 0

21 20 1 0 0 0 0

22 21 1 0 0 0 0

23 22 2 0 0 0 0

24 23 1 0 0 0 0

25 24 2 0 0 0 0

26 25 1 0 0 0 0

26 13 1 0 0 0 0

26 21 2 0 0 0 0

M END

**6-(11-hydroxyundecan-2-yl)phenanthridine (6g)**

SMILES Code: CC(CCCCCCCCCO)c2nc1ccccc1c3ccccc23

MoleFile:

Actelion Java MolfileCreator 1.0

26 28 0 0 0 0 0 0 0 0999 V2000

9.5263	-0.0000	-0.0000 C	0 0 0 0 0 0 0 0 0 0 0 0

8.6603	-0.5000	-0.0000 C	0 0 0 0 0 0 0 0 0 0 0 0

7.7942	-0.0000	-0.0000 C	0 0 0 0 0 0 0 0 0 0 0 0

6.9282	-0.5000	-0.0000 C	0 0 0 0 0 0 0 0 0 0 0 0

6.0622	-0.0000	-0.0000 C	0 0 0 0 0 0 0 0 0 0 0 0

5.1962	-0.5000	-0.0000 C	0 0 0 0 0 0 0 0 0 0 0 0

4.3301	-0.0000	-0.0000 C	0 0 0 0 0 0 0 0 0 0 0 0

3.4641	-0.5000	-0.0000 C	0 0 0 0 0 0 0 0 0 0 0 0

2.5981	-0.0000	-0.0000 C	0 0 0 0 0 0 0 0 0 0 0 0

1.7321	-0.5000	-0.0000 C	0 0 0 0 0 0 0 0 0 0 0 0

0.8660	-0.0000	-0.0000 C	0 0 0 0 0 0 0 0 0 0 0 0

0.0000	-0.5000	-0.0000 O	0 0 0 0 0 0 0 0 0 0 0 0

8.6603	-1.5000	-0.0000 C	0 0 0 0 0 0 0 0 0 0 0 0

7.7942	-2.0000	-0.0000 N	0 0 0 0 0 0 0 0 0 0 0 0

7.7942	-3.0000	-0.0000 C	0 0 0 0 0 0 0 0 0 0 0 0

6.9282	-3.5000	-0.0000 C	0 0 0 0 0 0 0 0 0 0 0 0

6.9282	-4.5000	-0.0000 C	0 0 0 0 0 0 0 0 0 0 0 0

7.7942	-5.0000	-0.0000 C	0 0 0 0 0 0 0 0 0 0 0 0

8.6603	-4.5000	-0.0000 C	0 0 0 0 0 0 0 0 0 0 0 0

8.6603	-3.5000	-0.0000 C	0 0 0 0 0 0 0 0 0 0 0 0

9.5263	-3.0000	-0.0000 C	0 0 0 0 0 0 0 0 0 0 0 0

10.3923	-3.5000	-0.0000 C	0 0 0 0 0 0 0 0 0 0 0 0

11.2583	-3.0000	-0.0000 C	0 0 0 0 0 0 0 0 0 0 0 0

11.2583	-2.0000	-0.0000 C	0 0 0 0 0 0 0 0 0 0 0 0

10.3923	-1.5000	-0.0000 C	0 0 0 0 0 0 0 0 0 0 0 0

9.5263	-2.0000	-0.0000 C	0 0 0 0 0 0 0 0 0 0 0 0

2 1 1 0 0 0 0

3 2 1 0 0 0 0

4 3 1 0 0 0 0

5 4 1 0 0 0 0

6 5 1 0 0 0 0

7 6 1 0 0 0 0

8 7 1 0 0 0 0

9 8 1 0 0 0 0

10 9 1 0 0 0 0

11 10 1 0 0 0 0

12 11 1 0 0 0 0

13 2 1 0 0 0 0

14 13 2 0 0 0 0

15 14 1 0 0 0 0

16 15 1 0 0 0 0

17 16 2 0 0 0 0

18 17 1 0 0 0 0

19 18 2 0 0 0 0

20 19 1 0 0 0 0

20 15 2 0 0 0 0

21 20 1 0 0 0 0

22 21 1 0 0 0 0

23 22 2 0 0 0 0

24 23 1 0 0 0 0

25 24 2 0 0 0 0

26 25 1 0 0 0 0

26 13 1 0 0 0 0

26 21 2 0 0 0 0

M END

**6-(4-(benzoyloxy)butan-2-yl)phenanthridine (6h)**

SMILES Code: CC(CCOC(=O)c1ccccc1)c3nc2ccccc2c4ccccc34

MoleFile:

27 30 0 0 0 0 0 0 0 0999 V2000

2.4249	2.1000	0.0000 C	0 0 0 0 0 0 0 0 0 0 0 0

2.4249	3.5000	0.0000 C	0 0 0 0 0 0 0 0 0 0 0 0

1.2124	4.2000	0.0000 C	0 0 0 0 0 0 0 0 0 0 0 0

0.0000	3.5000	0.0000 C	0 0 0 0 0 0 0 0 0 0 0 0

0.0000	2.1000	0.0000 C	0 0 0 0 0 0 0 0 0 0 0 0

1.2124	1.4000	0.0000 C	0 0 0 0 0 0 0 0 0 0 0 0

3.6373	1.4000	0.0000 N	0 0 0 0 0 0 0 0 0 0 0 0

4.8497	2.1000	0.0000 C	0 0 0 0 0 0 0 0 0 0 0 0

4.8497	3.5000	0.0000 C	0 0 0 0 0 0 0 0 0 0 0 0

3.6373	4.2000	0.0000 C	0 0 0 0 0 0 0 0 0 0 0 0

6.0622	4.2000	0.0000 C	0 0 0 0 0 0 0 0 0 0 0 0

6.0622	5.6000	0.0000 C	0 0 0 0 0 0 0 0 0 0 0 0

4.8497	6.3000	0.0000 C	0 0 0 0 0 0 0 0 0 0 0 0

3.6373	5.6000	0.0000 C	0 0 0 0 0 0 0 0 0 0 0 0

6.0622	1.4000	0.0000 C	0 0 0 0 0 0 0 0 0 0 0 0

6.0622	0.0000	0.0000 C	0 0 0 0 0 0 0 0 0 0 0 0

7.2746	2.1000	0.0000 C	0 0 0 0 0 0 0 0 0 0 0 0

8.4870	1.4000	0.0000 C	0 0 0 0 0 0 0 0 0 0 0 0

9.6995	2.1000	0.0000 O	0 0 0 0 0 0 0 0 0 0 0 0

10.9119	1.4000	0.0000 C	0 0 0 0 0 0 0 0 0 0 0 0

12.1244	2.1000	0.0000 C	0 0 0 0 0 0 0 0 0 0 0 0

13.3368	1.4000	0.0000 C	0 0 0 0 0 0 0 0 0 0 0 0

14.5492	2.1000	0.0000 C	0 0 0 0 0 0 0 0 0 0 0 0

14.5492	3.5000	0.0000 C	0 0 0 0 0 0 0 0 0 0 0 0

13.3368	4.2000	0.0000 C	0 0 0 0 0 0 0 0 0 0 0 0

12.1244	3.5000	0.0000 C	0 0 0 0 0 0 0 0 0 0 0 0

10.9119	0.0000	0.0000 O	0 0 0 0 0 0 0 0 0 0 0 0

1 2 1 0 0 0 0

2 3 2 0 0 0 0

3 4 1 0 0 0 0

4 5 2 0 0 0 0

5 6 1 0 0 0 0

6 1 2 0 0 0 0

7 8 2 0 0 0 0

8 9 1 0 0 0 0

9 10 2 0 0 0 0

2 10 1 0 0 0 0

1 7 1 0 0 0 0

11 12 2 0 0 0 0

12 13 1 0 0 0 0

13 14 2 0 0 0 0

10 14 1 0 0 0 0

9 11 1 0 0 0 0

8 15 1 0 0 0 0

15 16 1 0 0 0 0

15 17 1 0 0 0 0

17 18 1 0 0 0 0

18 19 1 0 0 0 0

19 20 1 0 0 0 0

20 21 1 0 0 0 0

22 23 1 0 0 0 0

23 24 2 0 0 0 0

24 25 1 0 0 0 0

25 26 2 0 0 0 0

21 26 1 0 0 0 0

21 22 2 0 0 0 0

20 27 2 0 0 0 0

M END

**6-(4-(1,3-dioxoisoindolin-2-yl)butan-2-yl)phenanthridine (6i)**

SMILES Code: CC(CCn2c(=O)c1ccccc1c2=O)c4nc3ccccc3c5ccccc45

MoleFile:

29 33 0 0 0 0 0 0 0 0999 V2000

2.4249	2.1000	0.0000 C	0 0 0 0 0 0 0 0 0 0 0 0

2.4249	3.5000	0.0000 C	0 0 0 0 0 0 0 0 0 0 0 0

1.2124	4.2000	0.0000 C	0 0 0 0 0 0 0 0 0 0 0 0

0.0000	3.5000	0.0000 C	0 0 0 0 0 0 0 0 0 0 0 0

0.0000	2.1000	0.0000 C	0 0 0 0 0 0 0 0 0 0 0 0

1.2124	1.4000	0.0000 C	0 0 0 0 0 0 0 0 0 0 0 0

3.6373	1.4000	0.0000 N	0 0 0 0 0 0 0 0 0 0 0 0

4.8497	2.1000	0.0000 C	0 0 0 0 0 0 0 0 0 0 0 0

4.8497	3.5000	0.0000 C	0 0 0 0 0 0 0 0 0 0 0 0

3.6373	4.2000	0.0000 C	0 0 0 0 0 0 0 0 0 0 0 0

6.0622	4.2000	0.0000 C	0 0 0 0 0 0 0 0 0 0 0 0

6.0622	5.6000	0.0000 C	0 0 0 0 0 0 0 0 0 0 0 0

4.8497	6.3000	0.0000 C	0 0 0 0 0 0 0 0 0 0 0 0

3.6373	5.6000	0.0000 C	0 0 0 0 0 0 0 0 0 0 0 0

6.0622	1.4000	0.0000 C	0 0 0 0 0 0 0 0 0 0 0 0

6.0622	0.0000	0.0000 C	0 0 0 0 0 0 0 0 0 0 0 0

7.2746	2.1000	0.0000 C	0 0 0 0 0 0 0 0 0 0 0 0

8.4870	1.4000	0.0000 C	0 0 0 0 0 0 0 0 0 0 0 0

9.6995	2.1000	0.0000 N	0 0 0 0 0 0 0 0 0 0 0 0

10.9784	1.5306	0.0000 C	0 0 0 0 0 0 0 0 0 0 0 0

11.9152	2.5710	0.0000 C	0 0 0 0 0 0 0 0 0 0 0 0

11.2152	3.7834	0.0000 C	0 0 0 0 0 0 0 0 0 0 0 0

9.8458	3.4923	0.0000 C	0 0 0 0 0 0 0 0 0 0 0 0

13.3152	2.5710	0.0000 C	0 0 0 0 0 0 0 0 0 0 0 0

14.0152	3.7834	0.0000 C	0 0 0 0 0 0 0 0 0 0 0 0

13.3152	4.9958	0.0000 C	0 0 0 0 0 0 0 0 0 0 0 0

11.9152	4.9958	0.0000 C	0 0 0 0 0 0 0 0 0 0 0 0

8.8054	4.4291	0.0000 O	0 0 0 0 0 0 0 0 0 0 0 0

11.2695	0.1612	0.0000 O	0 0 0 0 0 0 0 0 0 0 0 0

1 2 1 0 0 0 0

2 3 2 0 0 0 0

3 4 1 0 0 0 0

4 5 2 0 0 0 0

5 6 1 0 0 0 0

6 1 2 0 0 0 0

7 8 2 0 0 0 0

8 9 1 0 0 0 0

9 10 2 0 0 0 0

2 10 1 0 0 0 0

1 7 1 0 0 0 0

11 12 2 0 0 0 0

12 13 1 0 0 0 0

13 14 2 0 0 0 0

10 14 1 0 0 0 0

9 11 1 0 0 0 0

8 15 1 0 0 0 0

15 16 1 0 0 0 0

15 17 1 0 0 0 0

17 18 1 0 0 0 0

18 19 1 0 0 0 0

20 21 1 0 0 0 0

21 22 1 0 0 0 0

22 23 1 0 0 0 0

19 23 1 0 0 0 0

19 20 1 0 0 0 0

24 25 1 0 0 0 0

25 26 2 0 0 0 0

26 27 1 0 0 0 0

22 27 2 0 0 0 0

21 24 2 0 0 0 0

23 28 2 0 0 0 0

20 29 2 0 0 0 0

M END

**6-cyclopentylphenanthridine (6j)**

SMILES Code: c1ccc4c(c1)nc(C2CCCC2)c3ccccc34

MoleFile:

Actelion Java MolfileCreator 1.0

19 22 0 0 0 0 0 0 0 0999 V2000

4.3301	-0.0000	-0.0000 C	0 0 0 0 0 0 0 0 0 0 0 0

4.3301	1.0000	-0.0000 C	0 0 0 0 0 0 0 0 0 0 0 0

3.4641	1.5000	-0.0000 C	0 0 0 0 0 0 0 0 0 0 0 0

2.5981	1.0000	-0.0000 C	0 0 0 0 0 0 0 0 0 0 0 0

2.5981	-0.0000	-0.0000 C	0 0 0 0 0 0 0 0 0 0 0 0

3.4641	-0.5000	-0.0000 C	0 0 0 0 0 0 0 0 0 0 0 0

1.7321	-0.5000	-0.0000 N	0 0 0 0 0 0 0 0 0 0 0 0

0.8660	-0.0000	-0.0000 C	0 0 0 0 0 0 0 0 0 0 0 0

0.0000	-0.5000	-0.0000 C	0 0 0 0 0 0 0 0 0 0 0 0

-0.9135	-0.0933	-0.0000 C	0 0 0 0 0 0 0 0 0 0 0 0

-1.5827	-0.8364	-0.0000 C	0 0 0 0 0 0 0 0 0 0 0 0

-1.0827	-1.7024	-0.0000 C	0 0 0 0 0 0 0 0 0 0 0 0

-0.1045	-1.4945	-0.0000 C	0 0 0 0 0 0 0 0 0 0 0 0

0.8660	1.0000	-0.0000 C	0 0 0 0 0 0 0 0 0 0 0 0

0.0000	1.5000	-0.0000 C	0 0 0 0 0 0 0 0 0 0 0 0

0.0000	2.5000	-0.0000 C	0 0 0 0 0 0 0 0 0 0 0 0

0.8660	3.0000	-0.0000 C	0 0 0 0 0 0 0 0 0 0 0 0

1.7321	2.5000	-0.0000 C	0 0 0 0 0 0 0 0 0 0 0 0

1.7321	1.5000	-0.0000 C	0 0 0 0 0 0 0 0 0 0 0 0

2 1 2 0 0 0 0

3 2 1 0 0 0 0

4 3 2 0 0 0 0

5 4 1 0 0 0 0

6 5 2 0 0 0 0

6 1 1 0 0 0 0

7 5 1 0 0 0 0

8 7 2 0 0 0 0

9 8 1 0 0 0 0

10 9 1 0 0 0 0

11 10 1 0 0 0 0

12 11 1 0 0 0 0

13 12 1 0 0 0 0

13 9 1 0 0 0 0

14 8 1 0 0 0 0

15 14 2 0 0 0 0

16 15 1 0 0 0 0

17 16 2 0 0 0 0

18 17 1 0 0 0 0

19 18 2 0 0 0 0

19 14 1 0 0 0 0

19 4 1 0 0 0 0

M END

**6-cyclohexylphenanthridine (6k)**

SMILES Code: c1ccc4c(c1)nc(C2CCCCC2)c3ccccc34

MoleFile:

Actelion Java MolfileCreator 1.0

20 23 0 0 0 0 0 0 0 0999 V2000

4.3301	-0.0000	-0.0000 C	0 0 0 0 0 0 0 0 0 0 0 0

4.3301	1.0000	-0.0000 C	0 0 0 0 0 0 0 0 0 0 0 0

3.4641	1.5000	-0.0000 C	0 0 0 0 0 0 0 0 0 0 0 0

2.5981	1.0000	-0.0000 C	0 0 0 0 0 0 0 0 0 0 0 0

2.5981	-0.0000	-0.0000 C	0 0 0 0 0 0 0 0 0 0 0 0

3.4641	-0.5000	-0.0000 C	0 0 0 0 0 0 0 0 0 0 0 0

1.7321	-0.5000	-0.0000 N	0 0 0 0 0 0 0 0 0 0 0 0

0.8660	-0.0000	-0.0000 C	0 0 0 0 0 0 0 0 0 0 0 0

0.0000	-0.5000	-0.0000 C	0 0 0 0 0 0 0 0 0 0 0 0

-0.8660	0.0000	-0.0000 C	0 0 0 0 0 0 0 0 0 0 0 0

-1.7321	-0.5000	-0.0000 C	0 0 0 0 0 0 0 0 0 0 0 0

-1.7321	-1.5000	-0.0000 C	0 0 0 0 0 0 0 0 0 0 0 0

-0.8660	-2.0000	-0.0000 C	0 0 0 0 0 0 0 0 0 0 0 0

-0.0000	-1.5000	-0.0000 C	0 0 0 0 0 0 0 0 0 0 0 0

0.8660	1.0000	-0.0000 C	0 0 0 0 0 0 0 0 0 0 0 0

0.0000	1.5000	-0.0000 C	0 0 0 0 0 0 0 0 0 0 0 0

0.0000	2.5000	-0.0000 C	0 0 0 0 0 0 0 0 0 0 0 0

0.8660	3.0000	-0.0000 C	0 0 0 0 0 0 0 0 0 0 0 0

1.7321	2.5000	-0.0000 C	0 0 0 0 0 0 0 0 0 0 0 0

1.7321	1.5000	-0.0000 C	0 0 0 0 0 0 0 0 0 0 0 0

2 1 2 0 0 0 0

3 2 1 0 0 0 0

4 3 2 0 0 0 0

5 4 1 0 0 0 0

6 5 2 0 0 0 0

6 1 1 0 0 0 0

7 5 1 0 0 0 0

8 7 2 0 0 0 0

9 8 1 0 0 0 0

10 9 1 0 0 0 0

11 10 1 0 0 0 0

12 11 1 0 0 0 0

13 12 1 0 0 0 0

14 13 1 0 0 0 0

14 9 1 0 0 0 0

15 8 1 0 0 0 0

16 15 2 0 0 0 0

17 16 1 0 0 0 0

18 17 2 0 0 0 0

19 18 1 0 0 0 0

20 19 2 0 0 0 0

20 15 1 0 0 0 0

20 4 1 0 0 0 0

M END

**6-(1-methylcyclohexyl)-5,6-dihydrophenanthridine (6l)**

SMILES Code: CC4(C3Nc1ccccc1c2ccccc23)CCCCC4

MoleFile:

Actelion Java MolfileCreator 1.0

21 24 0 0 0 0 0 0 0 0999 V2000

0.8660	-0.0000	-0.0000 C	0 0 0 0 0 0 0 0 0 0 0 0

0.0000	-0.5000	-0.0000 C	0 0 0 0 0 0 0 0 0 0 0 0

0.8660	-1.0000	-0.0000 C	0 0 0 0 0 0 0 0 0 0 0 0

0.8660	-2.0000	-0.0000 N	0 0 0 0 0 0 0 0 0 0 0 0

1.7321	-2.5000	-0.0000 C	0 0 0 0 0 0 0 0 0 0 0 0

1.7321	-3.5000	-0.0000 C	0 0 0 0 0 0 0 0 0 0 0 0

2.5981	-4.0000	-0.0000 C	0 0 0 0 0 0 0 0 0 0 0 0

3.4641	-3.5000	-0.0000 C	0 0 0 0 0 0 0 0 0 0 0 0

3.4641	-2.5000	-0.0000 C	0 0 0 0 0 0 0 0 0 0 0 0

2.5981	-2.0000	-0.0000 C	0 0 0 0 0 0 0 0 0 0 0 0

2.5981	-1.0000	-0.0000 C	0 0 0 0 0 0 0 0 0 0 0 0

3.4641	-0.5000	-0.0000 C	0 0 0 0 0 0 0 0 0 0 0 0

3.4641	0.5000	-0.0000 C	0 0 0 0 0 0 0 0 0 0 0 0

2.5981	1.0000	-0.0000 C	0 0 0 0 0 0 0 0 0 0 0 0

1.7321	0.5000	-0.0000 C	0 0 0 0 0 0 0 0 0 0 0 0

1.7321	-0.5000	-0.0000 C	0 0 0 0 0 0 0 0 0 0 0 0

-0.8660	0.0000	-0.0000 C	0 0 0 0 0 0 0 0 0 0 0 0

-1.7321	-0.5000	-0.0000 C	0 0 0 0 0 0 0 0 0 0 0 0

-1.7321	-1.5000	-0.0000 C	0 0 0 0 0 0 0 0 0 0 0 0

-0.8660	-2.0000	-0.0000 C	0 0 0 0 0 0 0 0 0 0 0 0

-0.0000	-1.5000	-0.0000 C	0 0 0 0 0 0 0 0 0 0 0 0

2 1 1 0 0 0 0

3 2 1 0 0 0 0

4 3 1 0 0 0 0

5 4 1 0 0 0 0

6 5 2 0 0 0 0

7 6 1 0 0 0 0

8 7 2 0 0 0 0

9 8 1 0 0 0 0

10 9 2 0 0 0 0

10 5 1 0 0 0 0

11 10 1 0 0 0 0

12 11 2 0 0 0 0

13 12 1 0 0 0 0

14 13 2 0 0 0 0

15 14 1 0 0 0 0

16 15 2 0 0 0 0

16 11 1 0 0 0 0

16 3 1 0 0 0 0

17 2 1 0 0 0 0

18 17 1 0 0 0 0

19 18 1 0 0 0 0

20 19 1 0 0 0 0

21 20 1 0 0 0 0

21 2 1 0 0 0 0

M END

**6-(4-hydroxy-2-methylbutan-2-yl)-5,6-dihydrophenanthridine (6m)**

SMILES Code: CC(C)(CCO)C3Nc1ccccc1c2ccccc23

MoleFile:

20 22 0 0 0 0 0 0 0 0999 V2000

2.4249	2.1000	0.0000 C	0 0 0 0 0 0 0 0 0 0 0 0

2.4249	3.5000	0.0000 C	0 0 0 0 0 0 0 0 0 0 0 0

1.2124	4.2000	0.0000 C	0 0 0 0 0 0 0 0 0 0 0 0

0.0000	3.5000	0.0000 C	0 0 0 0 0 0 0 0 0 0 0 0

0.0000	2.1000	0.0000 C	0 0 0 0 0 0 0 0 0 0 0 0

1.2124	1.4000	0.0000 C	0 0 0 0 0 0 0 0 0 0 0 0

3.6373	1.4000	0.0000 N	0 0 0 0 0 0 0 0 0 0 0 0

4.8497	2.1000	0.0000 C	0 0 0 0 0 0 0 0 0 0 0 0

4.8497	3.5000	0.0000 C	0 0 0 0 0 0 0 0 0 0 0 0

3.6373	4.2000	0.0000 C	0 0 0 0 0 0 0 0 0 0 0 0

6.0622	4.2000	0.0000 C	0 0 0 0 0 0 0 0 0 0 0 0

6.0622	5.6000	0.0000 C	0 0 0 0 0 0 0 0 0 0 0 0

4.8497	6.3000	0.0000 C	0 0 0 0 0 0 0 0 0 0 0 0

3.6373	5.6000	0.0000 C	0 0 0 0 0 0 0 0 0 0 0 0

6.0622	1.4000	0.0000 C	0 0 0 0 0 0 0 0 0 0 0 0

6.0622	0.0000	0.0000 C	0 0 0 0 0 0 0 0 0 0 0 0

7.2746	2.1000	0.0000 C	0 0 0 0 0 0 0 0 0 0 0 0

8.4870	1.4000	0.0000 C	0 0 0 0 0 0 0 0 0 0 0 0

9.6995	2.1000	0.0000 O	0 0 0 0 0 0 0 0 0 0 0 0

7.2014	0.5862	0.0000 C	0 0 0 0 0 0 0 0 0 0 0 0

1 2 1 0 0 0 0

2 3 2 0 0 0 0

3 4 1 0 0 0 0

4 5 2 0 0 0 0

5 6 1 0 0 0 0

6 1 2 0 0 0 0

8 9 1 0 0 0 0

9 10 2 0 0 0 0

2 10 1 0 0 0 0

1 7 1 0 0 0 0

11 12 2 0 0 0 0

12 13 1 0 0 0 0

13 14 2 0 0 0 0

10 14 1 0 0 0 0

9 11 1 0 0 0 0

8 15 1 0 0 0 0

15 16 1 0 0 0 0

15 17 1 0 0 0 0

17 18 1 0 0 0 0

18 19 1 0 0 0 0

15 20 1 0 0 0 0

7 8 1 0 0 0 0

M END

**1-(4-Hydroxybutan-2-yl)-3-methoxycarbonyl-4-phenylisoquinoline (7a)**

SMILES Code: COC(=O)c2cc(C(C)CCO)c1ccccc1c2c3ccccc3

MoleFile:

Actelion Java MolfileCreator 1.0

25 27 0 0 0 0 0 0 0 0999 V2000

7.7942	-0.0000	-0.0000 C	0 0 0 0 0 0 0 0 0 0 0 0

6.9282	-0.5000	-0.0000 O	0 0 0 0 0 0 0 0 0 0 0 0

6.0622	-0.0000	-0.0000 C	0 0 0 0 0 0 0 0 0 0 0 0

6.0622	1.0000	-0.0000 O	0 0 0 0 0 0 0 0 0 0 0 0

5.1962	-0.5000	-0.0000 C	0 0 0 0 0 0 0 0 0 0 0 0

4.3301	-0.0000	-0.0000 C	0 0 0 0 0 0 0 0 0 0 0 0

3.4641	-0.5000	-0.0000 C	0 0 0 0 0 0 0 0 0 0 0 0

2.5981	-0.0000	-0.0000 C	0 0 0 0 0 0 0 0 0 0 0 0

2.5981	1.0000	-0.0000 C	0 0 0 0 0 0 0 0 0 0 0 0

1.7321	-0.5000	-0.0000 C	0 0 0 0 0 0 0 0 0 0 0 0

0.8660	-0.0000	-0.0000 C	0 0 0 0 0 0 0 0 0 0 0 0

0.0000	-0.5000	-0.0000 O	0 0 0 0 0 0 0 0 0 0 0 0

3.4641	-1.5000	-0.0000 C	0 0 0 0 0 0 0 0 0 0 0 0

2.5981	-2.0000	-0.0000 C	0 0 0 0 0 0 0 0 0 0 0 0

2.5981	-3.0000	-0.0000 C	0 0 0 0 0 0 0 0 0 0 0 0

3.4641	-3.5000	-0.0000 C	0 0 0 0 0 0 0 0 0 0 0 0

4.3301	-3.0000	-0.0000 C	0 0 0 0 0 0 0 0 0 0 0 0

4.3301	-2.0000	-0.0000 C	0 0 0 0 0 0 0 0 0 0 0 0

5.1962	-1.5000	-0.0000 C	0 0 0 0 0 0 0 0 0 0 0 0

6.0622	-2.0000	-0.0000 C	0 0 0 0 0 0 0 0 0 0 0 0

6.0622	-3.0000	-0.0000 C	0 0 0 0 0 0 0 0 0 0 0 0

6.9282	-3.5000	-0.0000 C	0 0 0 0 0 0 0 0 0 0 0 0

7.7942	-3.0000	-0.0000 C	0 0 0 0 0 0 0 0 0 0 0 0

7.7942	-2.0000	-0.0000 C	0 0 0 0 0 0 0 0 0 0 0 0

6.9282	-1.5000	-0.0000 C	0 0 0 0 0 0 0 0 0 0 0 0

2 1 1 0 0 0 0

3 2 1 0 0 0 0

4 3 2 0 0 0 0

5 3 1 0 0 0 0

6 5 2 0 0 0 0

7 6 1 0 0 0 0

8 7 1 0 0 0 0

9 8 1 0 0 0 0

10 8 1 0 0 0 0

11 10 1 0 0 0 0

12 11 1 0 0 0 0

13 7 2 0 0 0 0

14 13 1 0 0 0 0

15 14 2 0 0 0 0

16 15 1 0 0 0 0

17 16 2 0 0 0 0

18 17 1 0 0 0 0

18 13 1 0 0 0 0

19 18 2 0 0 0 0

19 5 1 0 0 0 0

20 19 1 0 0 0 0

21 20 2 0 0 0 0

22 21 1 0 0 0 0

23 22 2 0 0 0 0

24 23 1 0 0 0 0

25 24 2 0 0 0 0

25 20 1 0 0 0 0

M END

**Methyl 1-(4-(benzoyloxy)butan-2-yl)-4-phenylisoquinoline-3-carboxylate (7b)**

SMILES Code: COC(=O)c3cc(C(C)CCOC(=O)c1ccccc1)c2ccccc2c3c4ccccc4

MoleFile:

33 36 0 0 0 0 0 0 0 0999 V2000

2.4249	9.1000	0.0000 C	0 0 0 0 0 0 0 0 0 0 0 0

2.4249	10.5000	0.0000 C	0 0 0 0 0 0 0 0 0 0 0 0

1.2124	11.2000	0.0000 C	0 0 0 0 0 0 0 0 0 0 0 0

0.0000	10.5000	0.0000 C	0 0 0 0 0 0 0 0 0 0 0 0

0.0000	9.1000	0.0000 C	0 0 0 0 0 0 0 0 0 0 0 0

1.2124	8.4000	0.0000 C	0 0 0 0 0 0 0 0 0 0 0 0

3.6373	8.4000	0.0000 C	0 0 0 0 0 0 0 0 0 0 0 0

4.8497	9.1000	0.0000 C	0 0 0 0 0 0 0 0 0 0 0 0

4.8497	10.5000	0.0000 C	0 0 0 0 0 0 0 0 0 0 0 0

3.6373	11.2000	0.0000 C	0 0 0 0 0 0 0 0 0 0 0 0

3.6373	12.6000	0.0000 C	0 0 0 0 0 0 0 0 0 0 0 0

6.0622	11.2000	0.0000 C	0 0 0 0 0 0 0 0 0 0 0 0

7.2746	10.5000	0.0000 O	0 0 0 0 0 0 0 0 0 0 0 0

6.0622	12.6000	0.0000 O	0 0 0 0 0 0 0 0 0 0 0 0

7.2746	9.1000	0.0000 C	0 0 0 0 0 0 0 0 0 0 0 0

3.6373	7.0000	0.0000 C	0 0 0 0 0 0 0 0 0 0 0 0

2.4249	6.3000	0.0000 C	0 0 0 0 0 0 0 0 0 0 0 0

4.8497	6.3000	0.0000 C	0 0 0 0 0 0 0 0 0 0 0 0

4.8497	4.9000	0.0000 C	0 0 0 0 0 0 0 0 0 0 0 0

6.0622	4.2000	0.0000 O	0 0 0 0 0 0 0 0 0 0 0 0

6.0622	2.8000	0.0000 C	0 0 0 0 0 0 0 0 0 0 0 0

7.2746	2.1000	0.0000 C	0 0 0 0 0 0 0 0 0 0 0 0

4.8497	2.1000	0.0000 O	0 0 0 0 0 0 0 0 0 0 0 0

7.2746	0.7000	0.0000 C	0 0 0 0 0 0 0 0 0 0 0 0

8.4870	0.0000	0.0000 C	0 0 0 0 0 0 0 0 0 0 0 0

9.6995	0.7000	0.0000 C	0 0 0 0 0 0 0 0 0 0 0 0

9.6995	2.1000	0.0000 C	0 0 0 0 0 0 0 0 0 0 0 0

8.4870	2.8000	0.0000 C	0 0 0 0 0 0 0 0 0 0 0 0

4.8497	13.3000	0.0000 C	0 0 0 0 0 0 0 0 0 0 0 0

4.8497	14.7000	0.0000 C	0 0 0 0 0 0 0 0 0 0 0 0

3.6373	15.4000	0.0000 C	0 0 0 0 0 0 0 0 0 0 0 0

2.4249	14.7000	0.0000 C	0 0 0 0 0 0 0 0 0 0 0 0

2.4249	13.3000	0.0000 C	0 0 0 0 0 0 0 0 0 0 0 0

1 2 1 0 0 0 0

2 3 2 0 0 0 0

3 4 1 0 0 0 0

4 5 2 0 0 0 0

5 6 1 0 0 0 0

6 1 2 0 0 0 0

7 8 2 0 0 0 0

8 9 1 0 0 0 0

9 10 2 0 0 0 0

2 10 1 0 0 0 0

1 7 1 0 0 0 0

10 11 1 0 0 0 0

9 12 1 0 0 0 0

12 13 1 0 0 0 0

12 14 2 0 0 0 0

13 15 1 0 0 0 0

7 16 1 0 0 0 0

16 17 1 0 0 0 0

16 18 1 0 0 0 0

18 19 1 0 0 0 0

19 20 1 0 0 0 0

20 21 1 0 0 0 0

21 22 1 0 0 0 0

21 23 2 0 0 0 0

24 25 1 0 0 0 0

25 26 2 0 0 0 0

26 27 1 0 0 0 0

27 28 2 0 0 0 0

22 28 1 0 0 0 0

22 24 2 0 0 0 0

29 30 1 0 0 0 0

30 31 2 0 0 0 0

31 32 1 0 0 0 0

32 33 2 0 0 0 0

11 33 1 0 0 0 0

11 29 2 0 0 0 0

M END

**Methyl 4-phenyl-1-(4-phenylbutan-2-yl)isoquinoline-3-carboxylate (7c)**

SMILES Code: COC(=O)c3cc(C(C)CCc1ccccc1)c2ccccc2c3c4ccccc4

MoleFile:

30 33 0 0 0 0 0 0 0 0999 V2000

2.4249	7.0000	0.0000 C	0 0 0 0 0 0 0 0 0 0 0 0

2.4249	8.4000	0.0000 C	0 0 0 0 0 0 0 0 0 0 0 0

1.2124	9.1000	0.0000 C	0 0 0 0 0 0 0 0 0 0 0 0

0.0000	8.4000	0.0000 C	0 0 0 0 0 0 0 0 0 0 0 0

0.0000	7.0000	0.0000 C	0 0 0 0 0 0 0 0 0 0 0 0

1.2124	6.3000	0.0000 C	0 0 0 0 0 0 0 0 0 0 0 0

3.6373	6.3000	0.0000 C	0 0 0 0 0 0 0 0 0 0 0 0

4.8497	7.0000	0.0000 C	0 0 0 0 0 0 0 0 0 0 0 0

4.8497	8.4000	0.0000 C	0 0 0 0 0 0 0 0 0 0 0 0

3.6373	9.1000	0.0000 C	0 0 0 0 0 0 0 0 0 0 0 0

3.6373	10.5000	0.0000 C	0 0 0 0 0 0 0 0 0 0 0 0

6.0622	9.1000	0.0000 C	0 0 0 0 0 0 0 0 0 0 0 0

7.2746	8.4000	0.0000 O	0 0 0 0 0 0 0 0 0 0 0 0

6.0622	10.5000	0.0000 O	0 0 0 0 0 0 0 0 0 0 0 0

7.2746	7.0000	0.0000 C	0 0 0 0 0 0 0 0 0 0 0 0

3.6373	4.9000	0.0000 C	0 0 0 0 0 0 0 0 0 0 0 0

2.4249	4.2000	0.0000 C	0 0 0 0 0 0 0 0 0 0 0 0

4.8497	4.2000	0.0000 C	0 0 0 0 0 0 0 0 0 0 0 0

4.8497	2.8000	0.0000 C	0 0 0 0 0 0 0 0 0 0 0 0

4.8497	11.2000	0.0000 C	0 0 0 0 0 0 0 0 0 0 0 0

4.8497	12.6000	0.0000 C	0 0 0 0 0 0 0 0 0 0 0 0

3.6373	13.3000	0.0000 C	0 0 0 0 0 0 0 0 0 0 0 0

2.4249	12.6000	0.0000 C	0 0 0 0 0 0 0 0 0 0 0 0

2.4249	11.2000	0.0000 C	0 0 0 0 0 0 0 0 0 0 0 0

6.0622	2.1000	0.0000 C	0 0 0 0 0 0 0 0 0 0 0 0

6.0622	0.7000	0.0000 C	0 0 0 0 0 0 0 0 0 0 0 0

7.2746	0.0000	0.0000 C	0 0 0 0 0 0 0 0 0 0 0 0

8.4870	0.7000	0.0000 C	0 0 0 0 0 0 0 0 0 0 0 0

8.4870	2.1000	0.0000 C	0 0 0 0 0 0 0 0 0 0 0 0

7.2746	2.8000	0.0000 C	0 0 0 0 0 0 0 0 0 0 0 0

1 2 1 0 0 0 0

2 3 2 0 0 0 0

3 4 1 0 0 0 0

4 5 2 0 0 0 0

5 6 1 0 0 0 0

6 1 2 0 0 0 0

7 8 2 0 0 0 0

8 9 1 0 0 0 0

9 10 2 0 0 0 0

2 10 1 0 0 0 0

1 7 1 0 0 0 0

10 11 1 0 0 0 0

9 12 1 0 0 0 0

12 13 1 0 0 0 0

12 14 2 0 0 0 0

13 15 1 0 0 0 0

7 16 1 0 0 0 0

16 17 1 0 0 0 0

16 18 1 0 0 0 0

18 19 1 0 0 0 0

20 21 1 0 0 0 0

21 22 2 0 0 0 0

22 23 1 0 0 0 0

23 24 2 0 0 0 0

11 24 1 0 0 0 0

11 20 2 0 0 0 0

19 25 1 0 0 0 0

26 27 1 0 0 0 0

27 28 2 0 0 0 0

28 29 1 0 0 0 0

29 30 2 0 0 0 0

25 30 1 0 0 0 0

25 26 2 0 0 0 0

M END

**1-cyclohexyl-3-methoxycarbonyl-4-phenylisoquinoline (7d)**

SMILES Code: COC(=O)c3cc(C1CCCCC1)c2ccccc2c3c4ccccc4

MoleFile:

Actelion Java MolfileCreator 1.0

26 29 0 0 0 0 0 0 0 0999 V2000

2.5981	-0.0000	-0.0000 C	0 0 0 0 0 0 0 0 0 0 0 0

1.7321	-0.5000	-0.0000 O	0 0 0 0 0 0 0 0 0 0 0 0

0.8660	-0.0000	-0.0000 C	0 0 0 0 0 0 0 0 0 0 0 0

0.8660	1.0000	-0.0000 O	0 0 0 0 0 0 0 0 0 0 0 0

0.0000	-0.5000	-0.0000 C	0 0 0 0 0 0 0 0 0 0 0 0

-0.8660	0.0000	-0.0000 C	0 0 0 0 0 0 0 0 0 0 0 0

-1.7321	-0.5000	-0.0000 C	0 0 0 0 0 0 0 0 0 0 0 0

-2.5981	0.0000	-0.0000 C	0 0 0 0 0 0 0 0 0 0 0 0

-2.5981	1.0000	-0.0000 C	0 0 0 0 0 0 0 0 0 0 0 0

-3.4641	1.5000	-0.0000 C	0 0 0 0 0 0 0 0 0 0 0 0

-4.3301	1.0000	-0.0000 C	0 0 0 0 0 0 0 0 0 0 0 0

-4.3301	0.0000	-0.0000 C	0 0 0 0 0 0 0 0 0 0 0 0

-3.4641	-0.5000	-0.0000 C	0 0 0 0 0 0 0 0 0 0 0 0

-1.7321	-1.5000	-0.0000 C	0 0 0 0 0 0 0 0 0 0 0 0

-2.5981	-2.0000	-0.0000 C	0 0 0 0 0 0 0 0 0 0 0 0

-2.5981	-3.0000	-0.0000 C	0 0 0 0 0 0 0 0 0 0 0 0

-1.7321	-3.5000	-0.0000 C	0 0 0 0 0 0 0 0 0 0 0 0

-0.8660	-3.0000	-0.0000 C	0 0 0 0 0 0 0 0 0 0 0 0

-0.8660	-2.0000	-0.0000 C	0 0 0 0 0 0 0 0 0 0 0 0

-0.0000	-1.5000	-0.0000 C	0 0 0 0 0 0 0 0 0 0 0 0

0.8660	-2.0000	-0.0000 C	0 0 0 0 0 0 0 0 0 0 0 0

0.8660	-3.0000	-0.0000 C	0 0 0 0 0 0 0 0 0 0 0 0

1.7321	-3.5000	-0.0000 C	0 0 0 0 0 0 0 0 0 0 0 0

2.5981	-3.0000	-0.0000 C	0 0 0 0 0 0 0 0 0 0 0 0

2.5981	-2.0000	-0.0000 C	0 0 0 0 0 0 0 0 0 0 0 0

1.7321	-1.5000	-0.0000 C	0 0 0 0 0 0 0 0 0 0 0 0

2 1 1 0 0 0 0

3 2 1 0 0 0 0

4 3 2 0 0 0 0

5 3 1 0 0 0 0

6 5 2 0 0 0 0

7 6 1 0 0 0 0

8 7 1 0 0 0 0

9 8 1 0 0 0 0

10 9 1 0 0 0 0

11 10 1 0 0 0 0

12 11 1 0 0 0 0

13 12 1 0 0 0 0

13 8 1 0 0 0 0

14 7 2 0 0 0 0

15 14 1 0 0 0 0

16 15 2 0 0 0 0

17 16 1 0 0 0 0

18 17 2 0 0 0 0

19 18 1 0 0 0 0

19 14 1 0 0 0 0

20 19 2 0 0 0 0

20 5 1 0 0 0 0

21 20 1 0 0 0 0

22 21 2 0 0 0 0

23 22 1 0 0 0 0

24 23 2 0 0 0 0

25 24 1 0 0 0 0

26 25 2 0 0 0 0

26 21 1 0 0 0 0

M END

**1-(1-methylcyclohexyl)-3-methoxycarbonyl-4-phenylisoquinoline (7e)**

SMILES Code: COC(=O)c3cc(C1(C)CCCCC1)c2ccccc2c3c4ccccc4

Mole File:

Actelion Java MolfileCreator 1.0

27 30 0 0 0 0 0 0 0 0999 V2000

2.5981	-0.0000	-0.0000 C	0 0 0 0 0 0 0 0 0 0 0 0

1.7321	-0.5000	-0.0000 O	0 0 0 0 0 0 0 0 0 0 0 0

0.8660	-0.0000	-0.0000 C	0 0 0 0 0 0 0 0 0 0 0 0

0.8660	1.0000	-0.0000 O	0 0 0 0 0 0 0 0 0 0 0 0

0.0000	-0.5000	-0.0000 C	0 0 0 0 0 0 0 0 0 0 0 0

-0.8660	0.0000	-0.0000 C	0 0 0 0 0 0 0 0 0 0 0 0

-1.7321	-0.5000	-0.0000 C	0 0 0 0 0 0 0 0 0 0 0 0

-2.5981	0.0000	-0.0000 C	0 0 0 0 0 0 0 0 0 0 0 0

-2.5981	-1.0000	-0.0000 C	0 0 0 0 0 0 0 0 0 0 0 0

-2.5981	1.0000	-0.0000 C	0 0 0 0 0 0 0 0 0 0 0 0

-3.4641	1.5000	-0.0000 C	0 0 0 0 0 0 0 0 0 0 0 0

-4.3301	1.0000	-0.0000 C	0 0 0 0 0 0 0 0 0 0 0 0

-4.3301	0.0000	-0.0000 C	0 0 0 0 0 0 0 0 0 0 0 0

-3.4641	-0.5000	-0.0000 C	0 0 0 0 0 0 0 0 0 0 0 0

-1.7321	-1.5000	-0.0000 C	0 0 0 0 0 0 0 0 0 0 0 0

-2.5981	-2.0000	-0.0000 C	0 0 0 0 0 0 0 0 0 0 0 0

-2.5981	-3.0000	-0.0000 C	0 0 0 0 0 0 0 0 0 0 0 0

-1.7321	-3.5000	-0.0000 C	0 0 0 0 0 0 0 0 0 0 0 0

-0.8660	-3.0000	-0.0000 C	0 0 0 0 0 0 0 0 0 0 0 0

-0.8660	-2.0000	-0.0000 C	0 0 0 0 0 0 0 0 0 0 0 0

-0.0000	-1.5000	-0.0000 C	0 0 0 0 0 0 0 0 0 0 0 0

0.8660	-2.0000	-0.0000 C	0 0 0 0 0 0 0 0 0 0 0 0

0.8660	-3.0000	-0.0000 C	0 0 0 0 0 0 0 0 0 0 0 0

1.7321	-3.5000	-0.0000 C	0 0 0 0 0 0 0 0 0 0 0 0

2.5981	-3.0000	-0.0000 C	0 0 0 0 0 0 0 0 0 0 0 0

2.5981	-2.0000	-0.0000 C	0 0 0 0 0 0 0 0 0 0 0 0

1.7321	-1.5000	-0.0000 C	0 0 0 0 0 0 0 0 0 0 0 0

2 1 1 0 0 0 0

3 2 1 0 0 0 0

4 3 2 0 0 0 0

5 3 1 0 0 0 0

6 5 2 0 0 0 0

7 6 1 0 0 0 0

8 7 1 0 0 0 0

9 8 1 0 0 0 0

10 8 1 0 0 0 0

11 10 1 0 0 0 0

12 11 1 0 0 0 0

13 12 1 0 0 0 0

14 13 1 0 0 0 0

14 8 1 0 0 0 0

15 7 2 0 0 0 0

16 15 1 0 0 0 0

17 16 2 0 0 0 0

18 17 1 0 0 0 0

19 18 2 0 0 0 0

20 19 1 0 0 0 0

20 15 1 0 0 0 0

21 20 2 0 0 0 0

21 5 1 0 0 0 0

22 21 1 0 0 0 0

23 22 2 0 0 0 0

24 23 1 0 0 0 0

25 24 2 0 0 0 0

26 25 1 0 0 0 0

27 26 2 0 0 0 0

27 22 1 0 0 0 0

M END

**2-(4-Hydroxybutan-2-yl)-3-(2-oxo-2-phenylethyl)indole (8a)**

SMILES Code: CC(CCO)c2[nH]c1ccccc1c2CC(=O)c3ccccc3

MoleFile:

Actelion Java MolfileCreator 1.0

23 25 0 0 0 0 0 0 0 0999 V2000

3.4641	-0.5000	-0.0000 C	0 0 0 0 0 0 0 0 0 0 0 0

2.5981	-0.0000	-0.0000 C	0 0 0 0 0 0 0 0 0 0 0 0

1.7321	-0.5000	-0.0000 C	0 0 0 0 0 0 0 0 0 0 0 0

0.8660	-0.0000	-0.0000 C	0 0 0 0 0 0 0 0 0 0 0 0

0.0000	-0.5000	-0.0000 O	0 0 0 0 0 0 0 0 0 0 0 0

2.5981	1.0000	-0.0000 C	0 0 0 0 0 0 0 0 0 0 0 0

3.4071	1.5878	-0.0000 N	0 0 0 0 0 0 0 0 0 0 0 0

3.0981	2.5388	-0.0000 C	0 0 0 0 0 0 0 0 0 0 0 0

3.5981	3.4049	-0.0000 C	0 0 0 0 0 0 0 0 0 0 0 0

3.0981	4.2709	-0.0000 C	0 0 0 0 0 0 0 0 0 0 0 0

2.0981	4.2709	-0.0000 C	0 0 0 0 0 0 0 0 0 0 0 0

1.5981	3.4049	-0.0000 C	0 0 0 0 0 0 0 0 0 0 0 0

2.0981	2.5388	-0.0000 C	0 0 0 0 0 0 0 0 0 0 0 0

1.7891	1.5878	-0.0000 C	0 0 0 0 0 0 0 0 0 0 0 0

0.8380	1.2788	-0.0000 C	0 0 0 0 0 0 0 0 0 0 0 0

0.0949	1.9479	-0.0000 C	0 0 0 0 0 0 0 0 0 0 0 0

0.3028	2.9260	-0.0000 O	0 0 0 0 0 0 0 0 0 0 0 0

-0.8562	1.6389	-0.0000 C	0 0 0 0 0 0 0 0 0 0 0 0

-1.5993	2.3080	-0.0000 C	0 0 0 0 0 0 0 0 0 0 0 0

-2.5504	1.9990	-0.0000 C	0 0 0 0 0 0 0 0 0 0 0 0

-2.7583	1.0208	-0.0000 C	0 0 0 0 0 0 0 0 0 0 0 0

-2.0152	0.3517	-0.0000 C	0 0 0 0 0 0 0 0 0 0 0 0

-1.0641	0.6607	-0.0000 C	0 0 0 0 0 0 0 0 0 0 0 0

2 1 1 0 0 0 0

3 2 1 0 0 0 0

4 3 1 0 0 0 0

5 4 1 0 0 0 0

6 2 1 0 0 0 0

7 6 1 0 0 0 0

8 7 1 0 0 0 0

9 8 2 0 0 0 0

10 9 1 0 0 0 0

11 10 2 0 0 0 0

12 11 1 0 0 0 0

13 12 2 0 0 0 0

13 8 1 0 0 0 0

14 13 1 0 0 0 0

14 6 2 0 0 0 0

15 14 1 0 0 0 0

16 15 1 0 0 0 0

17 16 2 0 0 0 0

18 16 1 0 0 0 0

19 18 2 0 0 0 0

20 19 1 0 0 0 0

21 20 2 0 0 0 0

22 21 1 0 0 0 0

23 22 2 0 0 0 0

23 18 1 0 0 0 0

M END

**2-(4-(benzoyloxy)butan-2-yl)-3-(2-oxo-2-phenylethyl)-1H-indole (8b)**

SMILES Code: CC(CCOC(=O)c1ccccc1)c3[nH]c2ccccc2c3CC(=O)c4ccccc4

MoleFile:

31 34 0 0 0 0 0 0 0 0999 V2000

2.4249	2.6714	0.0000 C	0 0 0 0 0 0 0 0 0 0 0 0

2.4249	4.0714	0.0000 C	0 0 0 0 0 0 0 0 0 0 0 0

1.2124	4.7714	0.0000 C	0 0 0 0 0 0 0 0 0 0 0 0

0.0000	4.0714	0.0000 C	0 0 0 0 0 0 0 0 0 0 0 0

0.0000	2.6714	0.0000 C	0 0 0 0 0 0 0 0 0 0 0 0

1.2124	1.9714	0.0000 C	0 0 0 0 0 0 0 0 0 0 0 0

3.7564	2.2388	0.0000 N	0 0 0 0 0 0 0 0 0 0 0 0

4.5792	3.3714	0.0000 C	0 0 0 0 0 0 0 0 0 0 0 0

3.7564	4.5040	0.0000 C	0 0 0 0 0 0 0 0 0 0 0 0

4.1890	5.8355	0.0000 C	0 0 0 0 0 0 0 0 0 0 0 0

5.5584	6.1266	0.0000 C	0 0 0 0 0 0 0 0 0 0 0 0

5.9792	3.3714	0.0000 C	0 0 0 0 0 0 0 0 0 0 0 0

5.9910	7.4580	0.0000 C	0 0 0 0 0 0 0 0 0 0 0 0

6.4952	5.0862	0.0000 O	0 0 0 0 0 0 0 0 0 0 0 0

7.3604	7.7491	0.0000 C	0 0 0 0 0 0 0 0 0 0 0 0

7.7930	9.0806	0.0000 C	0 0 0 0 0 0 0 0 0 0 0 0

6.8563	10.1210	0.0000 C	0 0 0 0 0 0 0 0 0 0 0 0

5.4868	9.8299	0.0000 C	0 0 0 0 0 0 0 0 0 0 0 0

5.0542	8.4984	0.0000 C	0 0 0 0 0 0 0 0 0 0 0 0

5.7328	1.9932	0.0000 C	0 0 0 0 0 0 0 0 0 0 0 0

7.3755	3.4738	0.0000 C	0 0 0 0 0 0 0 0 0 0 0 0

8.1624	2.3159	0.0000 C	0 0 0 0 0 0 0 0 0 0 0 0

9.5586	2.4184	0.0000 O	0 0 0 0 0 0 0 0 0 0 0 0

10.3455	1.2604	0.0000 C	0 0 0 0 0 0 0 0 0 0 0 0

11.7417	1.3629	0.0000 C	0 0 0 0 0 0 0 0 0 0 0 0

9.7361	0.0000	0.0000 O	0 0 0 0 0 0 0 0 0 0 0 0

12.5286	0.2049	0.0000 C	0 0 0 0 0 0 0 0 0 0 0 0

13.9248	0.3074	0.0000 C	0 0 0 0 0 0 0 0 0 0 0 0

14.5342	1.5678	0.0000 C	0 0 0 0 0 0 0 0 0 0 0 0

13.7473	2.7258	0.0000 C	0 0 0 0 0 0 0 0 0 0 0 0

12.3511	2.6233	0.0000 C	0 0 0 0 0 0 0 0 0 0 0 0

1 2 1 0 0 0 0

2 3 2 0 0 0 0

3 4 1 0 0 0 0

4 5 2 0 0 0 0

5 6 1 0 0 0 0

6 1 2 0 0 0 0

7 8 1 0 0 0 0

8 9 2 0 0 0 0

2 9 1 0 0 0 0

1 7 1 0 0 0 0

9 10 1 0 0 0 0

10 11 1 0 0 0 0

8 12 1 0 0 0 0

11 13 1 0 0 0 0

11 14 2 0 0 0 0

15 16 1 0 0 0 0

16 17 2 0 0 0 0

17 18 1 0 0 0 0

18 19 2 0 0 0 0

13 19 1 0 0 0 0

13 15 2 0 0 0 0

12 20 1 0 0 0 0

12 21 1 0 0 0 0

21 22 1 0 0 0 0

22 23 1 0 0 0 0

23 24 1 0 0 0 0

24 25 1 0 0 0 0

24 26 2 0 0 0 0

27 28 1 0 0 0 0

28 29 2 0 0 0 0

29 30 1 0 0 0 0

30 31 2 0 0 0 0

25 31 1 0 0 0 0

25 27 2 0 0 0 0

M END

**Ethyl 2-(4-hydroxybutan-2-yl)-3-indoleacetate (9a)**

SMILES Code: CCOC(=O)Cc1c(C(C)CCO)[nH]c2ccccc12

MoleFile:

Actelion Java MolfileCreator 1.0

20 21 0 0 0 0 0 0 0 0999 V2000

4.3301	-0.0000	-0.0000 C	0 0 0 0 0 0 0 0 0 0 0 0

3.4641	-0.5000	-0.0000 C	0 0 0 0 0 0 0 0 0 0 0 0

2.5981	-0.0000	-0.0000 O	0 0 0 0 0 0 0 0 0 0 0 0

1.7321	-0.5000	-0.0000 C	0 0 0 0 0 0 0 0 0 0 0 0

1.7321	-1.5000	-0.0000 O	0 0 0 0 0 0 0 0 0 0 0 0

0.8660	-0.0000	-0.0000 C	0 0 0 0 0 0 0 0 0 0 0 0

0.0000	-0.5000	-0.0000 C	0 0 0 0 0 0 0 0 0 0 0 0

-0.9135	-0.0933	-0.0000 C	0 0 0 0 0 0 0 0 0 0 0 0

-1.1215	0.8849	-0.0000 C	0 0 0 0 0 0 0 0 0 0 0 0

-0.3783	1.5540	-0.0000 C	0 0 0 0 0 0 0 0 0 0 0 0

-2.0725	1.1939	-0.0000 C	0 0 0 0 0 0 0 0 0 0 0 0

-2.2804	2.1720	-0.0000 C	0 0 0 0 0 0 0 0 0 0 0 0

-3.2315	2.4811	-0.0000 O	0 0 0 0 0 0 0 0 0 0 0 0

-1.5827	-0.8364	-0.0000 N	0 0 0 0 0 0 0 0 0 0 0 0

-1.0827	-1.7024	-0.0000 C	0 0 0 0 0 0 0 0 0 0 0 0

-1.3917	-2.6535	-0.0000 C	0 0 0 0 0 0 0 0 0 0 0 0

-0.7226	-3.3966	-0.0000 C	0 0 0 0 0 0 0 0 0 0 0 0

0.2556	-3.1887	-0.0000 C	0 0 0 0 0 0 0 0 0 0 0 0

0.5646	-2.2377	-0.0000 C	0 0 0 0 0 0 0 0 0 0 0 0

-0.1045	-1.4945	-0.0000 C	0 0 0 0 0 0 0 0 0 0 0 0

2 1 1 0 0 0 0

3 2 1 0 0 0 0

4 3 1 0 0 0 0

5 4 2 0 0 0 0

6 4 1 0 0 0 0

7 6 1 0 0 0 0

8 7 2 0 0 0 0

9 8 1 0 0 0 0

10 9 1 0 0 0 0

11 9 1 0 0 0 0

12 11 1 0 0 0 0

13 12 1 0 0 0 0

14 8 1 0 0 0 0

15 14 1 0 0 0 0

16 15 2 0 0 0 0

17 16 1 0 0 0 0

18 17 2 0 0 0 0

19 18 1 0 0 0 0

20 19 2 0 0 0 0

20 7 1 0 0 0 0

20 15 1 0 0 0 0

M END

**3-(2-ethoxy-2-oxoethyl)-2-(4-phenylbutan-2-yl)-1H-indole (9b)**

SMILES Code: CCOC(=O)Cc2c(C(C)CCc1ccccc1)[nH]c3ccccc23

MoleFile:

25 27 0 0 0 0 0 0 0 0999 V2000

2.4249	1.4110	0.0000 C	0 0 0 0 0 0 0 0 0 0 0 0

2.4249	2.8110	0.0000 C	0 0 0 0 0 0 0 0 0 0 0 0

1.2124	3.5110	0.0000 C	0 0 0 0 0 0 0 0 0 0 0 0

0.0000	2.8110	0.0000 C	0 0 0 0 0 0 0 0 0 0 0 0

0.0000	1.4110	0.0000 C	0 0 0 0 0 0 0 0 0 0 0 0

1.2124	0.7110	0.0000 C	0 0 0 0 0 0 0 0 0 0 0 0

3.7564	0.9783	0.0000 N	0 0 0 0 0 0 0 0 0 0 0 0

4.5792	2.1110	0.0000 C	0 0 0 0 0 0 0 0 0 0 0 0

3.7564	3.2436	0.0000 C	0 0 0 0 0 0 0 0 0 0 0 0

4.1890	4.5751	0.0000 C	0 0 0 0 0 0 0 0 0 0 0 0

5.5584	4.8661	0.0000 C	0 0 0 0 0 0 0 0 0 0 0 0

5.9792	2.1110	0.0000 C	0 0 0 0 0 0 0 0 0 0 0 0

5.9910	6.1976	0.0000 O	0 0 0 0 0 0 0 0 0 0 0 0

6.4952	3.8257	0.0000 O	0 0 0 0 0 0 0 0 0 0 0 0

5.7328	0.7328	0.0000 C	0 0 0 0 0 0 0 0 0 0 0 0

7.3755	2.2134	0.0000 C	0 0 0 0 0 0 0 0 0 0 0 0

8.1624	1.0555	0.0000 C	0 0 0 0 0 0 0 0 0 0 0 0

9.5586	1.1579	0.0000 C	0 0 0 0 0 0 0 0 0 0 0 0

10.3455	0.0000	0.0000 C	0 0 0 0 0 0 0 0 0 0 0 0

11.7417	0.1025	0.0000 C	0 0 0 0 0 0 0 0 0 0 0 0

12.3511	1.3629	0.0000 C	0 0 0 0 0 0 0 0 0 0 0 0

11.5642	2.5208	0.0000 C	0 0 0 0 0 0 0 0 0 0 0 0

10.1680	2.4184	0.0000 C	0 0 0 0 0 0 0 0 0 0 0 0

7.3604	6.4887	0.0000 C	0 0 0 0 0 0 0 0 0 0 0 0

7.7930	7.8202	0.0000 C	0 0 0 0 0 0 0 0 0 0 0 0

1 2 1 0 0 0 0

2 3 2 0 0 0 0

3 4 1 0 0 0 0

4 5 2 0 0 0 0

5 6 1 0 0 0 0

6 1 2 0 0 0 0

7 8 1 0 0 0 0

8 9 2 0 0 0 0

2 9 1 0 0 0 0

1 7 1 0 0 0 0

9 10 1 0 0 0 0

10 11 1 0 0 0 0

8 12 1 0 0 0 0

11 13 1 0 0 0 0

11 14 2 0 0 0 0

12 15 1 0 0 0 0

12 16 1 0 0 0 0

16 17 1 0 0 0 0

17 18 1 0 0 0 0

19 20 1 0 0 0 0

20 21 2 0 0 0 0

21 22 1 0 0 0 0

22 23 2 0 0 0 0

18 23 1 0 0 0 0

18 19 2 0 0 0 0

13 24 1 0 0 0 0

24 25 1 0 0 0 0

M END
